# Mechanochemical Defluorinative Arylation of Trifluoroacetamides: An Entry to Aromatic Amides

**DOI:** 10.1021/acs.joc.2c02197

**Published:** 2023-01-09

**Authors:** Satenik Mkrtchyan, Mohanad Shkoor, Mandalaparthi Phanindrudu, Miroslav Medved′, Olena Sevastyanova, Viktor O. Iaroshenko

**Affiliations:** † Department of Chemistry, Faculty of Natural Sciences, Matej Bel University, Tajovského 40, 97401 Banská Bystrica, Slovakia; ‡ Department of Chemistry and Earth Sciences, Qatar University, P.O. Box 2713, Doha, Qatar; § Inorganic and Physical Chemistry Division, CSIR-Indian Institute of Chemical Technology, Uppal Road, Tarnaka, Hyderabad 500607, India; ∥ Regional Centre of Advanced Technologies and Materials, Czech Advanced Technology and Research Institute, Palacky University Olomouc, Křížkovského 511/8, 77900 Olomouc, Czech Republic; ⊥ Wallenberg Wood Science Center, Department of Fibre and Polymer Technology, KTH Royal Institute of Technology, Teknikringen 56-58, SE-10044 Stockholm, Sweden; # Division of Wood Chemistry and Pulp Technology, Department of Fiber and Polymer Technology, School of Chemistry, Biotechnology and Health, KTH Royal Institute of Technology, Teknikringen 56-58, 100 44 Stockholm, Sweden; ∇ Department of Chemistry, University of Helsinki, A.I. Virtasen aukio 1, 00014 Helsinki, Finland; ○ Department of Biology/Chemistry, Center for Cellular Nanoanalytics (CellNanOs), Universität Osnabrück, Barbarastr. 7, D-49076 Osnabrück, Germany

## Abstract

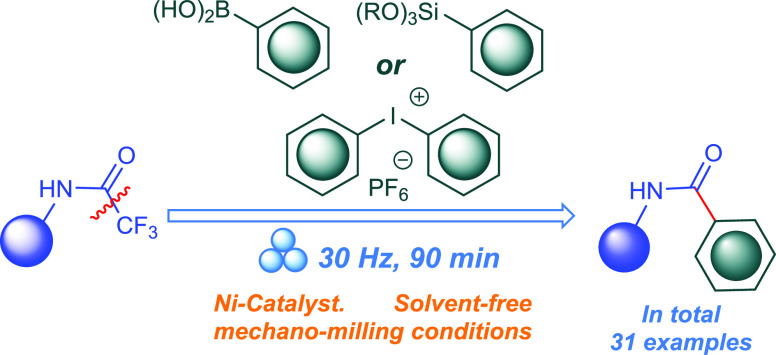

The amide bond is prominent in natural and synthetic organic molecules endowed with activity in various fields. Among a wide array of amide synthetic methods, substitution on a pre-existing (O)C–N moiety is an underexplored strategy for the synthesis of amides. In this work, we disclose a new protocol for the defluorinative arylation of aliphatic and aromatic trifluoroacetamides yielding aromatic amides. The mechanochemically induced reaction of either arylboronic acids, trimethoxyphenylsilanes, diaryliodonium salts, or dimethyl(phenyl)sulfonium salts with trifluoroacetamides affords substituted aromatic amides in good to excellent yields. These nickel-catalyzed reactions are enabled by C–CF_3_ bond activation using Dy_2_O_3_ as an additive. The current protocol provides versatile and scalable routes for accessing a wide variety of substituted aromatic amides. Moreover, the protocol described in this work overcomes the drawbacks and limitations in the previously reported methods.

## Introduction

The amide bond is a privileged and ubiquitous structural scaffold that comprises the backbone for peptides and a vast number of natural products, bioactive molecules, and marketed pharmaceuticals. Approximately a quarter of the current marketed drugs and drug lead molecules encompass amide linkage; hence, the reactions involving the amide bond creation and functionalization are the most performed processes in the drug industry, accounting for nearly 16% of total reported chemical synthesis reactions.^1−5^ Moreover, the amide bond is routinely encountered in molecules serving as lubricants, pesticides, perfumes, and agrochemicals.^4,6−11^ From a synthetic organic chemistry point of view, amides play a pivotal role as catalysts^12,13^ and building blocks to access a wide variety of valuable molecules.^14−19^ As a consequence, amide bond formation and derivatization continue to attract the attention of synthetic organic and process chemists.

A vast number of protocols for the synthesis and derivatization of amides has been reported.^19−22^ Nevertheless, the substitution onto a pre-existing (O)C–N structural motif has emerged as an elegant protocol to access various substituted amides, overcoming the drawbacks associated with the dehydrative condensation protocol such as the stoichiometric use of high molecular mass carboxylic acid activators and the poor atom economy. In this context, carbamoylation reactions represent a straightforward tool toward the functionalization of (O)C–N moieties. Although a variety of carbamoyl surrogates and catalytic systems were developed and provided elegant routes to amides, most of these methods are limited by harsh conditions to generate the carbamoyl radical, especially when formamides are used as the carbamoyl source, excess use of oxidants, elevated reactions temperatures, poor atom economy, and lack of tolerance of many functional groups.^23−27^

**Scheme 1:**
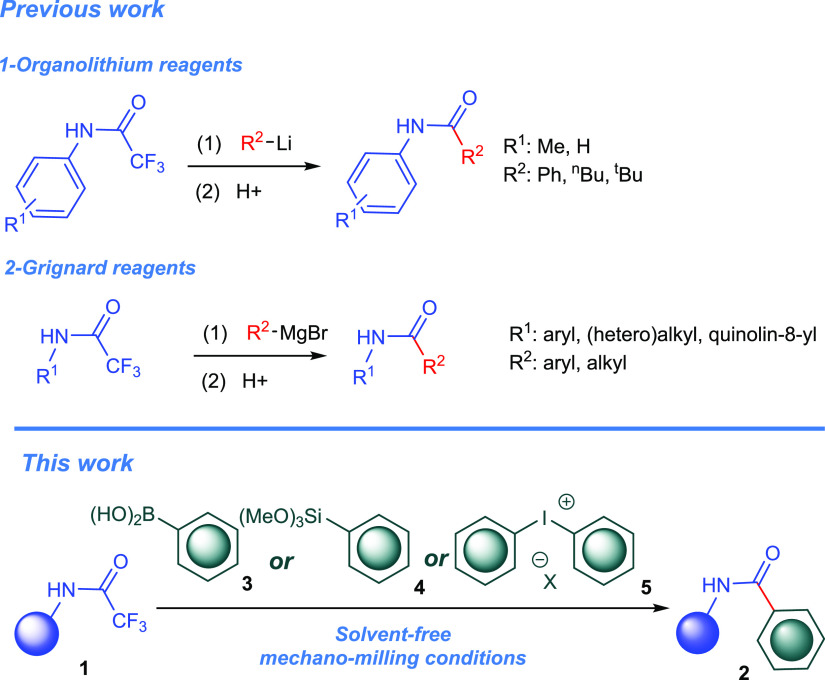
Previous Works and Our Synthetic Scenario

Carbamoylation reactions employing trihaloacetamides^28−31^ as carbamoyl surrogates, via loss of CX_3_, have been scarcely studied. In particular, trifluoroacetamides are preferred over the other trihaloacetamides for their bench stability and preparation feasibility. Despite the inertness of the N(O)C–CF_3_ bond, the trifluoroacetamido functionality has served as a carbamoyl surrogate via the cleavage of the N(O)C–CF_3_ fragment, which has been utilized to create C(O)–N^32,33^ and C(O)–O^34,35^ bonds. However, the formation of the N(O)C–C bond as an approach for the synthesis of substituted amides has been underexplored so far. Smith and co-workers reported the formation of anilides by the defluorinative arylation of trifluoroacetamides with organolithium compounds (Scheme 1).^36^ Due to the high reactivity of organolithium reagents, this protocol lacks tolerance of many functional groups, in particular halogenated precursors, and therefore suffers from a narrow synthetic scope. Recently, Kambe’s group employed Grignard reagents as the organometallic coupling partners in the defluorinative arylation and alkylation of trifluoroacetamides (Scheme 1).^37^ The reaction involved inherent drawbacks such as prolonged heating time, large excess of the Grignard reagent, moderate to low yields when alkyl Grignard reagents were employed, lack of the scope diversity, and practical and safety concerns associated with the use of Grignard reagents. As a consequence, the discovery of facile procedures for defluorinative arylation of trifluoroacetamides is still sought after.

## Results and Discussion

To circumvent the need for highly reactive C-nucleophiles to achieve the cleavage of N(O)C-CF_3_ bond of trifluoroacetamides, the C-F bonds need to be activated.^38,39^ It is known that trivalent lanthanides are capable of polarizing C(sp^3^)–F bonds and thus activate them for further cleavage and subsequent functionalization.^40,41^ Since lanthanides on the right side of the periodic table are stronger activator of C(sp^3^)–F bonds, we envisioned that dysprosium(III) oxide can be a candidate to activate the C(sp^3^)–F bonds of trifluoroacetamides.

**Scheme 2:**
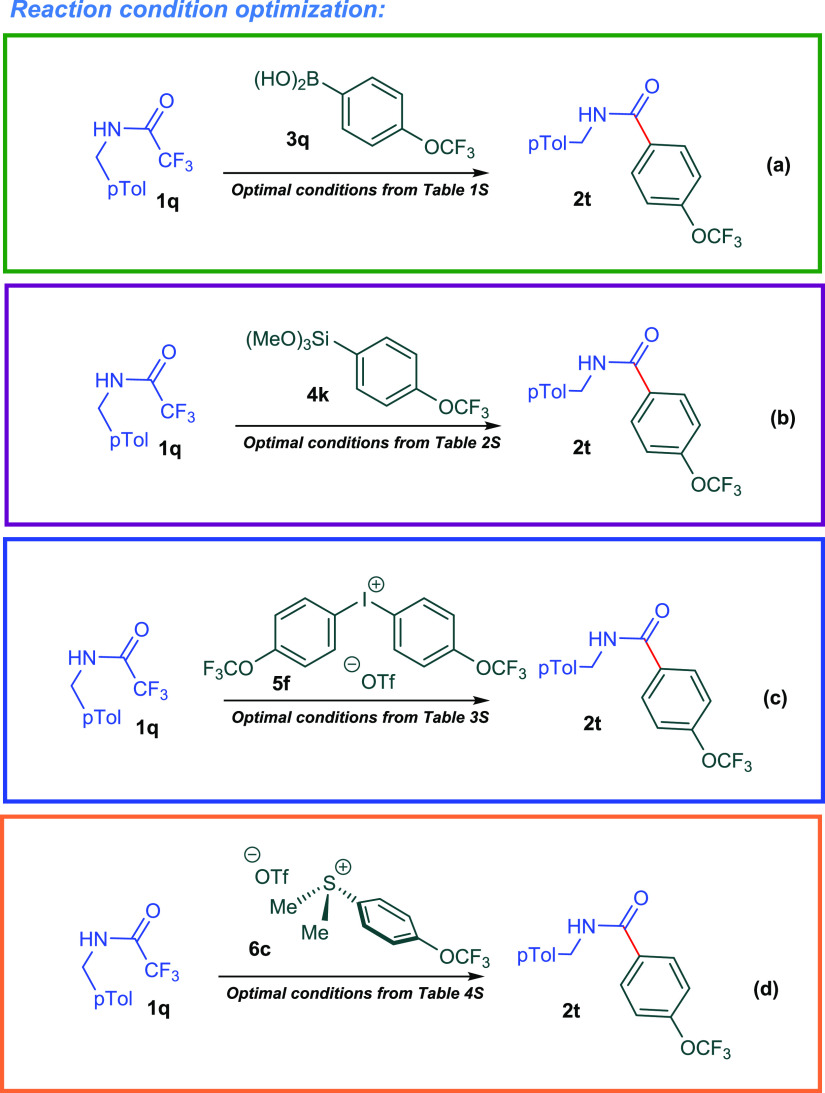
Model Reactions for Reaction Condition Optimization

There is a growing emphasis on the environmental impact of new synthetic procedures. In this perspective, mechanochemically induced transformations as solvent-free and energy-efficient processes have gained substantial interest.^42−45^ Of note, the employment of trifluoroacetamides in carbamoylation reactions can be considered as an effort toward environment remedy as organofluorine compounds are long-lived and persistent in the environment. In view of the above and in pursuit of our research efforts in the chemistry of fluorinated organic compounds,^46,47^ herein, we report a facile protocol for defluorinative coupling of trifluoroacetamides with four different arylating agents, namely, arylboronic acids, trimethoxyphenylsilanes, diaryliodonium salts, and dimethyl(phenyl)sulfonium salts (Scheme 2), yielding a wide variety of substituted amides.

We commenced our endeavor by seeking the optimal reaction conditions for the reaction of trifluoroacetanilide **1q** with 4-(trifluoromethoxy)phenyl boronic acid **3q**. Our preliminary exploratory experiments revealed that the reaction proceeded only in the presence of the 0.1 equivalent of cucurbit[6]uril, which is a pumpkin-shaped supramolecular framework that features both electronegative carbonyl portals and hydrophobic void. We assume that the cucurbit[6]uril acts as a molecular container by encapsulation of reacting molecules in its cavity, thus increasing their effective concentrations and pre-organizing them to achieve a specific conformation to facilitate the titled reactions.^48−51^

A variety of palladium salts including Pd(OAc)_2_, PdCl_2_, and PdCl_2_(PPh_3_)_2_ was screened for their ability to catalyze the model reaction (Scheme 2a, Table S1) with 10 mol % catalyst loading. Among those palladium salts, PdCl_2_ provided the highest isolated yield of 72%. In the course of these experiments, we found that DABCO surpassed K_2_CO_3_ in enhancing the reaction yield. In contrast to palladium salts, CoCl_2_ failed to catalyze the reaction. The reaction yield decreased dramatically when 10 mol % of CuCl or CuCl_2_ was utilized. Upon screening of a variety of Ni(II) salts, we found that NiBr_2_ in combination with DABCO provided the highest isolated reaction yield of 87%. Moreover, our initial studies established that Dy_2_O_3_ was the best additive when compared to other lanthanide oxides such as La_2_O_3_, Ce_2_O_3_, Sm_2_O, Eu_2_O_3_, and Yb_2_O_3_. To validate the demand for a mechanochemical protocol, we studied the model reaction in a variety of solvents under conventional heating conditions. Most of solvent-dependent reactions failed to produce the target amide under these optimized conditions.

Inspired by this success, we then directed our efforts toward optimizing reaction conditions for our second route for the defluorinative arylation of trifluoroacetamides (Scheme 2b, Table S2). The reaction of trifluoroacetanilide **1q** with trimethoxy(4-(trifluoromethoxy)phenyl)silane **4k** was nominated as the model reaction. We embarked on testing the previous optimal conditions (mechano-milling, NiBr_2_, DABCO, Dy_2_O_3_). To our delight, the target amide was produced in 86% yield, which was the highest reaction yield we can obtain. Interestingly, replacing DABCO with K_2_CO_3_ failed to produce the target amide. In addition to that, no product was detected when CuCl_2_ was employed to catalyze the reaction. However, a replacement of NiBr_2_ with PdCl_2_ afforded the product in 80% reaction yield. Under the optimal reaction conditions for this transformation, replacing mechano-milling technique with a variety of solvents failed to yield the target product.

**Scheme 3:**
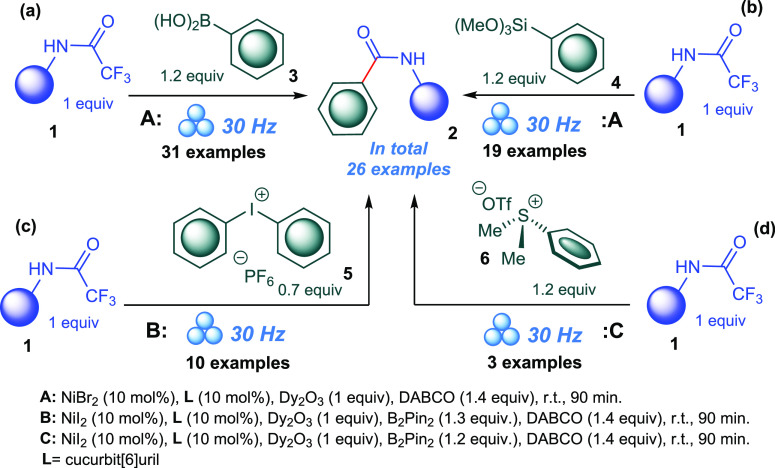
The Optimum Reaction Conditions for the Arylating Agents

The reaction of trifluoroacetanilide **1q** with bis(4-(trifluoro methoxy)phenyl)iodonium triflate **5f** represented our model reaction for the third route of defluorinative arylation of trifluoroacetamides (Scheme 2c, Table S3). In our initial trials, the previous optimal conditions failed to yield the target amide **2t**. Upon addition of bis(pinacolato)diboron to the reaction mixture, the target product **2t** was formed in 71%. Furthermore, replacement of NiBr_2_ with NiI_2_ gave the product in 83% yield as the highest isolated in our efforts to optimize this route. As expected, the use of the inorganic base Na_2_CO_3_ drastically decreased the yield to 10%. In other trials, both PdBr_2_ and PdCl_2_ catalyzed the transformations and afforded the target product in 63 and 55%, respectively. On the other hand, both CuCl_2_ and CoCl_2_ failed to catalyze the reaction. To our delight, under these optimum conditions, the reaction of trifluoroacetanilide **1q** with dimethyl(4-(trifluoromethoxy)phenyl)sulfonium triflate **6c** afforded the desired amide **2t** in 86% yield (Scheme 2d, Table S4). The reaction yields sharply decreased when DABCO was replaced by Na_2_CO_3_. It is important to note that in both cases, the additional of pinacol diborane was indispensable. We believe that it reacts in situ with iodonium and sulphonium salts, respectively, giving rise to the corresponding pinacol esters, which in turn enter the reaction. The latter reaction represents the model for our fourth route to achieve the defluorinative arylation of trifluoroacetamides. The optimized reaction conditions for the four routes are depicted in Scheme 3.

**Scheme 4:**
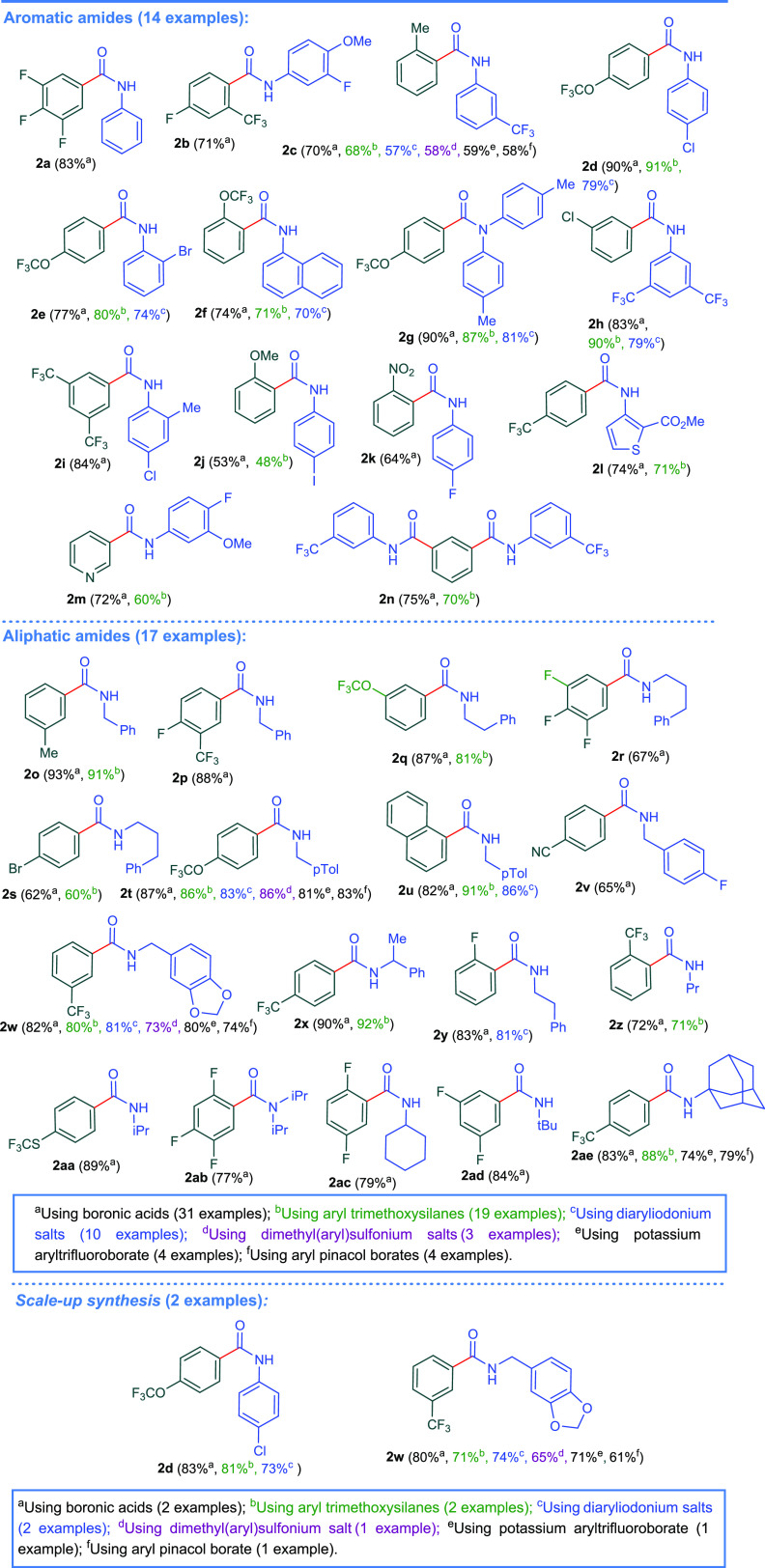
Synthetic Scope of the Current Protocol.

With the established optimized reaction condition for the four pathways in hands, we set out to scrutinize the synthetic scope of these reactions (Scheme 4) to determine the generality and limitations for our protocols. Under the optimized conditions, selective cleavage of the N(O)C–CF_3_ bond was observed as CF_3_–O, Ph–CF_3_, and Ph–F bonds in starting substrates remained intact. Consequently, we were successful in preparing a series of fluorinated aromatic amides in good to excellent yields. Furthermore, chlorinated aromatic amides were also prepared in very good yields; thereby, the current protocol overcomes the limitations of the previous protocols for halogenated substrates. We did not observe a general trend between the reaction yield and the electronic nature of the substituents on the arylating agent. To afford the scientific community with different choices, we synthesized the library of amides starting from the different arylating agents utilized in this study. In most cases, we noticed no significant influence of the arylating agent type on the reaction yield. For instance, compound **2e** was prepared in 77% yield starting from boronic acid, in 80% yield from aryl trimethoxysilane, and in 74% yield when diaryliodonium salt was used. A variety of aromatic and aliphatic reagents reacted smoothly under optimized conditions and afforded the corresponding amides in good to excellent yields. Of note, potassium aryltrifluoroborates and pinacol borates were also successful substrates and afforded the corresponding amides in very good yields.

Furthermore, primary and secondary trifluoroacetamides underwent defluorinative arylation to yield the target amides. To demonstrate the synthetic validity of our protocol, scale-up reactions successfully yielded the desired amides in high yields employing the various arylating agents utilized in the current work. Thus, compounds **2d** and **2w** were prepared in gram quantities (Scheme 4).

**Figure 1:**
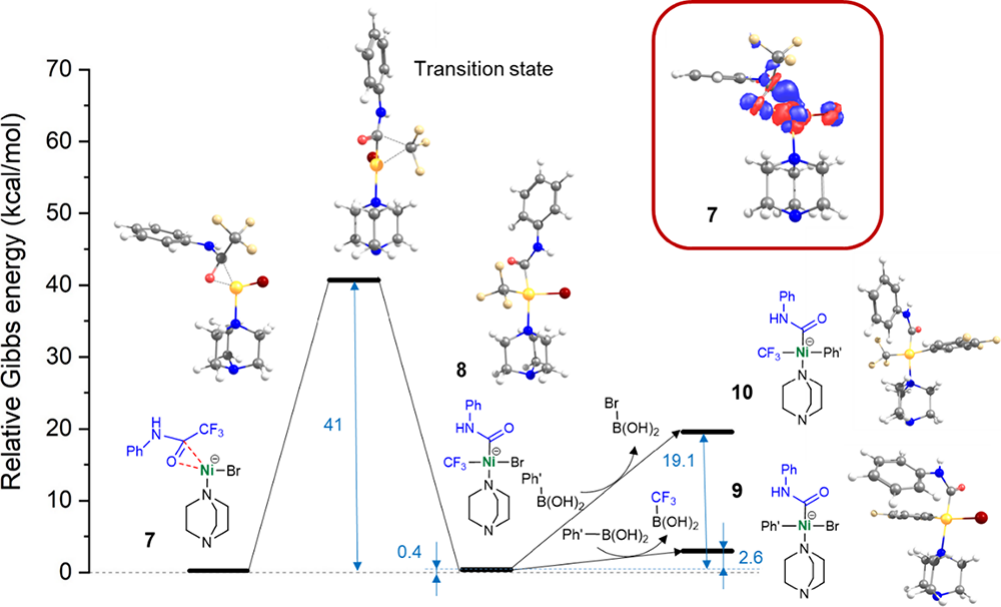
Energy diagram (ΔG° in kcal/mol; *T* = 298.15 K) demonstrating the C–CF_3_ bond activation by a reduced Ni^0^ site coordinated by DABCO and Br^–^ anion and a subsequent reaction of the complex with 3,4,5-trifluoro-phenyl-boronic acid (Ph–B(OH)_2_) as an arylation agent. The displayed structures were optimized at the ωB97X-D/def2-SVP level of theory (color coding: H/C/N/O/F/Ni/Br—white/ gray/blue/red/beige/yellow/dark red). Inset: EDD plot indicating the changes in electron density distribution upon the binding of an amide to the Ni^0^ site (red/blue indicates decrease/increase of the electron density; isovalue = 0.015 au).

The observed sensitivity of the catalytic activity on utilized chemical ingredients in our mechanochemical protocol indicates their intricate interplay that can hardly be fully rationalized at the atomistic level. Nevertheless, to address the feasibility of the C–CF_3_ bond activation in the presence of Ni species, we performed density functional theory (DFT) calculations employing the ωB97X-D functional^52^ in combination with the def2-SVP atomic basis set^53^ for several model systems using the Gaussian 16 program.^54^ In particular, we considered Ni species in oxidation states +2 and 0, the latter being highly probable under the applied conditions,^55^ and we assumed that they prefer to be tetra-coordinated with a reagent amide, DABCO, and bromide anions as strong electron donors (Figure 1). We found that the Ni^2+^ species preferably coordinate with the amide via a carbonyl oxygen atom in the presence of either one or two bromide anions (Figures S1 and S2). Importantly, in both cases the structure with a split C–CF_3_ bond is energetically highly unfavorable (by ∼45–50 kcal/mol compared to the initial structure), thus diminishing the possibility of this oxidation state being catalytically active. On the other hand, the zero-valent nickel provides notably different interactions with the ligands. As in the case of Ni^2+^, in the presence of two bromide anions, it preferentially coordinates the amide via an oxygen atom on the carbonyl group (Figure S3). However, a spontaneous release of a Br^–^ anion indicates the preference for the formation of mono-bromo complexes. It is also worth noting that the di-bromo complex with a cleaved C–CF_3_ bond is again energetically unfavorable (Figure S3, structure C). In the most stable mono-bromo complex **7** (Figure S4, structure A), the central Ni^0^ atom interacts with π orbitals on the carbonyl group (see the electron density difference (EDD) plot in the inset of Figure S5). Interestingly, the structure **8** with a cleaved C–CF_3_ bond (Figure S5 and Figure S4, structure D) is in this case energetically comparable with **7**, and the activation barrier (∼41 kcal/mol) for the transformation is reachable assuming the mechano-milling conditions. In the next step, the reaction of **8** with an arylation agent (phenyl–boronic acid, Ph–B(OH)_2_) leads to a ligand exchange with a preferential release of the CF_3_ group (via the formation of CF_3_–B(OH)_2_) producing complex **9** with a convenient arrangement of the ligands for the final coupling reaction resulting in required products (Figure 1).

## Summary

In conclusion, a new mechanochemical protocol for the synthesis of a wide variety of aromatic amides is disclosed. The current protocol adopts a tactic based on the functionalization of a pre-existing (O)C–N fragment. The nickel-catalyzed, dysprosium(III) oxide-mediated defluorinative coupling of substituted trifluoroacetanilide with either arylboronic acids, trimethoxyphenylsilanes, diaryliodonium salts, or dimethyl(phenyl)sulfonium proceeded smoothly to afford the target amides. The employment of various arylating reagents can potentially afford the synthetic community with many synthetic options. DFT calculations were performed to elucidate the reaction mechanism and to corroborate the active role of Ni species in the current reaction. In particular, the coordination sphere and the electron donor character of Ni(0) in the presence of DABCO and bromide anions facilitated the cleavage of the N(O)C–CF_3_ bond opening a reaction pathway for the final coupling of the amide with arylation agents.

## Supporting Information Available

General information; Cartesian coordinates; HRMS and MG MS data; spectral data; copies of ^1^H and ^13^C{^1^H} NMR spectra for all compounds prepared (PDF)
