# Supplementary material for: Mechanochemical Defluorinative Arylation of Trifluoroacetamides: An Entry to Aromatic Amides
Source: J Org Chem. 2023 Jan 9;88(2):863–70. doi: 10.1021/acs.joc.2c02197 (PMC9872087; doi:10.1021/acs.joc.2c02197)
Supplement: Supplementary file 1 — jo2c02197_si_001.pdf [file jo2c02197_si_001.pdf]

Supporting Information

Mechanochemical Defluorinative Arylation of Trifluoroacetamides: An entry to Aromatic Amides.

Satenik Mkrtchyan,[a]* Mohanad Shkoor,[b] Mandalaparthi Phanindrudu,[c] Miroslav Medveď,[a,d] Olena Sevastyanova,[e,f] Viktor O. Iaroshenko.[a,e,f,g,h] *

[a]Department of Chemistry, Faculty of Natural Sciences, Matej Bel University, Tajovského 40, 97401 Banská Bystrica (Slovakia).
E-mail: mkrtchyan.satenik@umb.sk E-mail: viktor.iaroshenko@umb.sk
[b]Department of Chemistry and Earth Sciences, Qatar University, P.O. Box 2713, Doha (Qatar).
[c]Inorganic and Physical Chemistry Division, CSIR-Indian Institute of Chemical Technology, Uppal road, Tarnaka, Hyderabad 500607 (India).
[d]Regional Centre of Advanced Technologies and Materials, Czech Advanced Technology and Research Institute, Palacky University Olomouc, Křížkovského 511/8, 77900 Olomouc (Czech Republic).
[e]Wallenberg Wood Science Center, Department of Fibre and Polymer Technology, KTH Royal Institute of Technology, Teknikringen 56-58, SE-10044, Stockholm (Sweden).
[f]Division of Wood Chemistry and Pulp Technology, Department of Fiber and Polymer Technology, School of Chemistry, Biotechnology and Health, KTH Royal Institute of Technology, Teknikringen 56-58, 100 44 Stockholm (Sweden). E-mail: viktiaro@kth.se
[g]Department of Chemistry, University of Helsinki, A.I. Virtasen aukio 1, 00014 Helsinki (Finland). E-mail: iva108@gmail.com
[h]Department of Biology/Chemistry, Center for Cellular Nanoanalytics (CellNanOs), Universität Osnabrück, Barbarastr. 7, D-49076 Osnabrück (Germany). E-Mail: viktor.iaroshenko@uni-osnabrueck.de

Table of Contents

(A) Experimental Section S3

(B) Characterization of products S13

(C) Copies ^{1}H and ^{13}C NMR spectra S42

(D) DFT Studies S105

(A) Experimental Section.

Commercially available starting materials, reagents, catalysts, anhydrous and degassed solvents were used without further purification. Flash column chromatography was performed with Merck Silica gel 60 (230-400 mesh). The solvents for column chromatography were distilled before the use. Thin layer chromatography was carried out using Merck TLC Silica gel 60 F_{254} and visualized by short-wavelength ultraviolet light or by treatment with potassium permanganate ($KMnO_4$) stain. ^{1}H, ^{13}C and ^{19}F NMR spectra were recorded on a Bruker 250, 400 and 500 MHz at 20ºC. All ^{1}H NMR spectra are reported in parts per million (ppm) downfield of TMS and were measured relative to the signals for $CHCl_3$ (7.26 ppm) and DMSO (2.50 ppm). All $^{13}C\{^{1}H\}$ NMR spectra were reported in ppm relative to residual $CHCl_3$ (77.00 ppm) or DMSO (39.70 ppm) and were obtained with ^{1}H decoupling. Coupling constants, *J*, are reported in Hertz (Hz). Gas chromatographic analyses was performed on Gas Chromatograph Mass Spectrometer GCMS-QP2010 Ultra instrument. Mechanochemical synthesis was performed using the Retsch MM400 mill using the standard kit. Liquid chemicals were dosed using gas tight micro syringes. Isolation of obtained compounds was achieved by column chromatography on Silica gel. All commercially available compounds were purchased from appropriate vendors.

A-1. Scope of the reagents used.

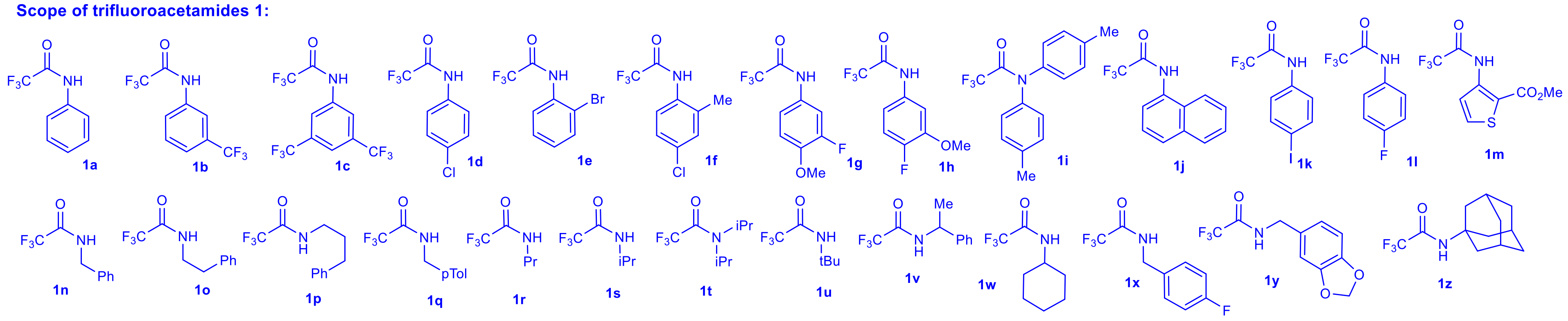

Scheme S1. List of trifluoroacetamids **1**.

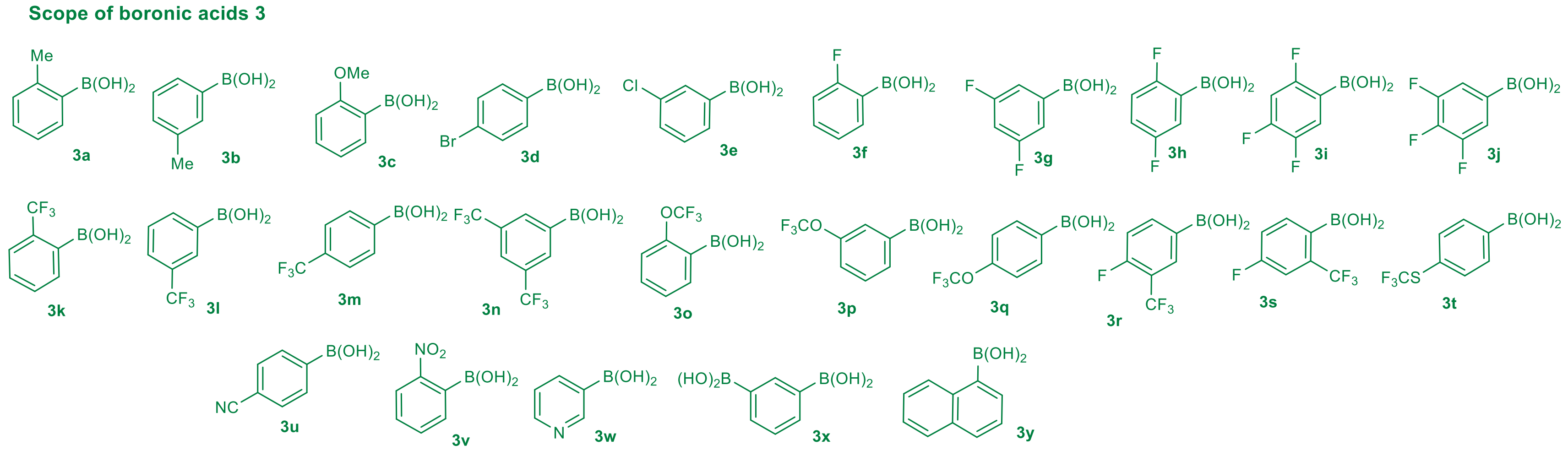

Scheme S2. List of boronic acids **3**.

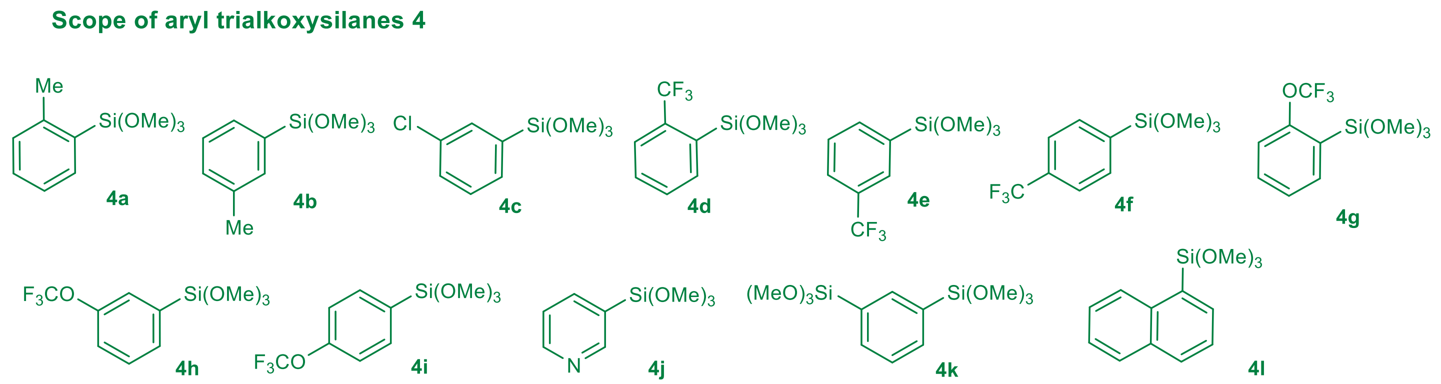

Scheme S3. List aryl trialkoxysilanes **4**.

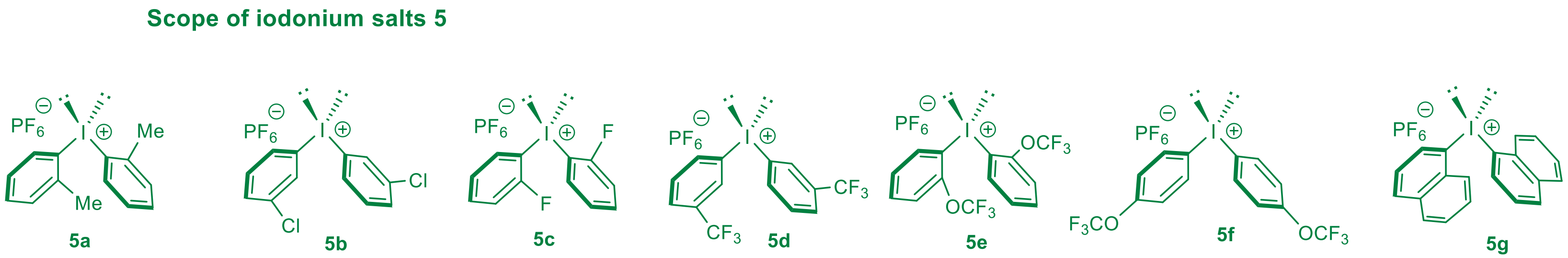

Scheme S4. List iodonium salts **5**.

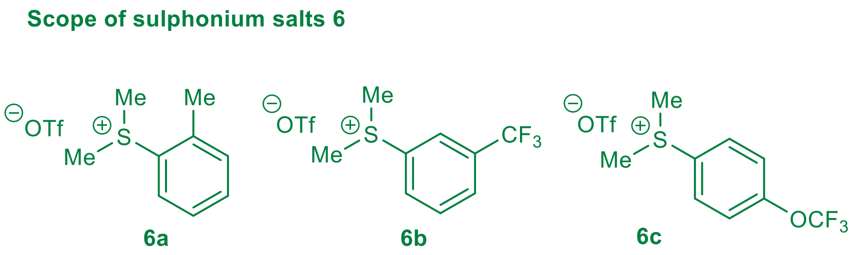

Scheme S5. List sulphonium salts **6**.

A-2. Reaction condition screening.

Table S1. Optimization of the reaction conditions.

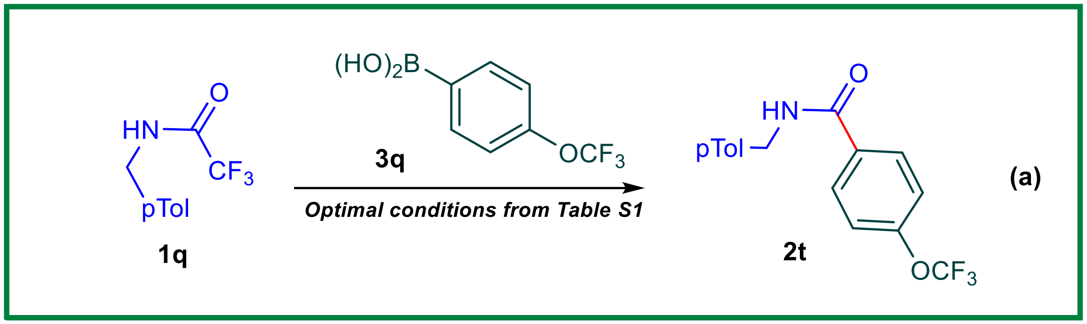

Reaction (a)			
entry	reaction components	frequency/time	yield (%) **2t**[a]
1	$Pd(OAc)_2$ (10 mol%), L (10 mol%), Dy_2O_3 (1 equiv.), K_2CO_3 (1.8 equiv.), r.t.	30Hz/90min	38
2	$PdCl_2$ (10 mol%), L (10 mol%), Dy_2O_3 (1 equiv.), K_2CO_3 (1.8 equiv.), r.t.	30Hz/90min	47
3	$Pd(OAc)_2$ (10 mol%), L (10 mol%), Dy_2O_3 (1 equiv.), DABCO (1.4 equiv.), r.t.	30Hz/90min	64
4	$PdCl_2$ (10 mol%), L (10 mol%), Dy_2O_3 (1 equiv.), DABCO (1.4 equiv.), r.t.	30Hz/90min	72
5	$PdCl_2(PPh_3)_2$ (10 mol%), L (10 mol%), Dy_2O_3 (1 equiv.), DABCO (1.4 equiv.), r.t.	30Hz/90min	60
6	$CoCl_2$ (10 mol%), L (10 mol%), Dy_2O_3 (1 equiv.), DABCO (1.4 equiv.), r.t.	30Hz/90min	0
7	CuI (10 mol%), L (10 mol%), Dy_2O_3 (1 equiv.), DABCO (1.4 equiv.), r.t.	30Hz/90min	22
8	$CuCl_2$ (0.1 equiv.), L (0.1 equiv.), Dy_2O_3 (1 equiv.), DABCO (1.4 equiv.), r.t.	30Hz/90min	17
9	$NiCl_2$ (10 mol%), L (10 mol%), Dy_2O_3 (1 equiv.), DABCO (1.4 equiv.), r.t.	30Hz/90min	44

10	$Ni(OAc)_2$ (10 mol%), L (10 mol%), Dy_2O_3 (1 equiv.), DABCO (1.4 equiv.), r.t.	30Hz/90min	0
**11**	**$NiBr_2$ (10 mol%), L (10 mol%), Dy_2O_3 (1 equiv.), DABCO (1.4 equiv.), r.t.**	**30Hz/90min**	**87**
12	NiI_2 (10 mol%), L (10 mol%), Dy_2O_3 (1 equiv.), DABCO (1.4 equiv.), r.t.	30Hz/90min	49
13	$NiBr_2$ (10 mol%), L (10 mol%), Dy_2O_3 (1 equiv.), K_2CO_3 (1.8 equiv.), r.t.	30Hz/90min	21
14	$NiBr_2$ (10 mol%), L (10 mol%), La_2O_3 (1 equiv.), DABCO (1.4 equiv.), r.t.	30Hz/90min	0
15	$NiBr_2$ (10 mol%), L (10 mol%), Ce_2O_3 (1 equiv.), DABCO (1.4 equiv.), r.t.	30Hz/90min	traces
16	$NiBr_2$ (10 mol%), L (10 mol%), Sm_2O_3 (1 equiv.), DABCO (1.4 equiv.), r.t.	30Hz/90min	11
17	$NiBr_2$ (10 mol%), L (10 mol%), Eu_2O_3 (1 equiv.), DABCO (1.4 equiv.), r.t.	30Hz/90min	42
18	$NiBr_2$ (10 mol%), L (10 mol%), Yb_2O_3 (1 equiv.), DABCO (1.4 equiv.), r.t.	30Hz/90min	25
**Reactions in solution**			
19	$NiBr_2$ (10 mol%), L (10 mol%), Dy_2O_3 (1 equiv.), DABCO (1.4 equiv.), reflux, in benzol.	------/18h	0
20	$NiBr_2$ (10 mol%), L (10 mol%), Dy_2O_3 (1 equiv.), DABCO (1.4 equiv.), reflux, in 1,4-dioxane.	------/18h	0
21	$NiBr_2$ (10 mol%), L (10 mol%), Dy_2O_3 (1 equiv.), DABCO (1.4 equiv.), 120 ^oC, in DMF.	------/18h	0
22	$NiBr_2$ (10 mol%), L (10 mol%), Dy_2O_3 (1 equiv.), DABCO (1.4 equiv.), 120 ^oC, in DMA.	------/18h	17
23	$NiBr_2$ (10 mol%), L (10 mol%), Dy_2O_3 (1 equiv.), DABCO (1.4 equiv.), reflux, in EtOH.	------/18h	0

[a] Isolated yield.

Table S2. Optimization of the reaction conditions.

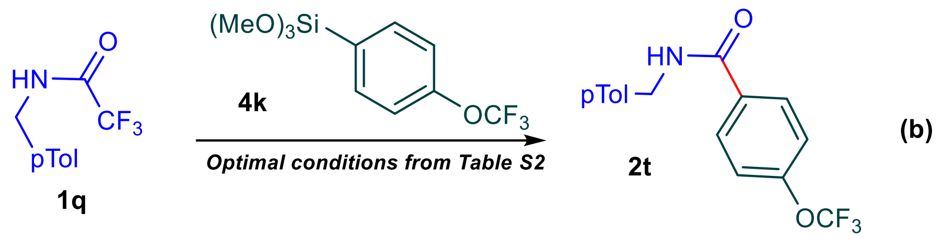

Reaction (b)			
entry	reaction components	frequency/time	yield (%) **2t**[a]
**1**	**$NiBr_2$ (10 mol%), L (10 mol%), Dy_2O_3 (1 equiv.), DABCO (1.4 equiv.), r.t.**	**30Hz/90min**	**86**
2	$NiBr_2$ (10 mol%), L (10 mol%), Dy_2O_3 (1 equiv.), K_2CO_3 (1.8 equiv.), r.t.	30Hz/90min	0
3	CuO (10 mol%), L (10 mol%), Dy_2O_3 (1 equiv.), DABCO (1.4 equiv.), r.t.	30Hz/90min	0
4	$CuCl_2$ (10 mol%), L (10 mol%), Dy_2O_3 (1 equiv.), DABCO (1.4 equiv.), r.t.	30Hz/90min	0
5	$PdCl_2$ (10 mol%), L (10 mol%), Dy_2O_3 (1 equiv.), DABCO (1.4 equiv.), r.t.	30Hz/90min	80
**Reactions in solution**			
6	$NiBr_2$ (10 mol%), L (10 mol%), Dy_2O_3 (1 equiv.), DABCO (1.4 equiv.), reflux, in benzol.	------/18h	0
7	$NiBr_2$ (10 mol%), L (10 mol%), Dy_2O_3 (1 equiv.), DABCO (1.4 equiv.), reflux, in 1,4-dioxane.	------/18h	0
8	$NiBr_2$ (10 mol%), L (10 mol%), Dy_2O_3 (1 equiv.), DABCO (1.4 equiv.), 120 ^oC, in DMF.	------/18h	0
9	$NiBr_2$ (10 mol%), L (10 mol%), Dy_2O_3 (1 equiv.), DABCO (1.4 equiv.), 120 ^oC, in DMA.	------/18h	0
10	$NiBr_2$ (10 mol%), L (10 mol%), Dy_2O_3 (1 equiv.), DABCO (1.4 equiv.), reflux, in EtOH.	------/18h	0

[a] Isolated yield.

Table S3. Optimization of the reaction conditions.

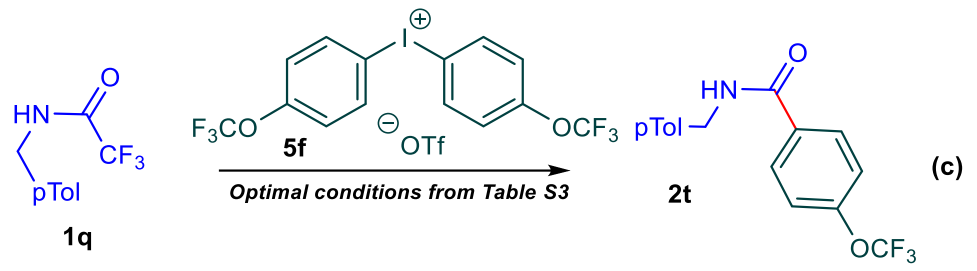

Reaction (c)			
entry	reaction components	milling frequency/time	yield (%) **2t**[a]
1	$NiBr_2$ (10 mol%), L (10 mol%), Dy_2O_3 (1 equiv.), DABCO (1.4 equiv.), r.t.	30Hz/90min	0
2	$PdBr_2$ (10 mol%), L (10 mol%), Dy_2O_3 (1 equiv.), B_2Pin_2 (1.3 equiv.), DABCO (1.4 equiv.), r.t.	30Hz/90min	63
3	$PdCl_2$ (10 mol%), L (10 mol%), Dy_2O_3 (1 equiv.), B_2Pin_2 (1.3 equiv.), DABCO (1.4 equiv.), r.t.	30Hz/90min	55
4	$CuCl_2$ (10 mol%), L (10 mol%), Dy_2O_3 (1 equiv.), B_2Pin_2 (1.3 equiv.), DABCO (1.4 equiv.), r.t.	30Hz/90min	0
5	$CoCl_2$ (10 mol%), L (10 mol%), Dy_2O_3 (1 equiv.), B_2Pin_2 (1.3 equiv.), DABCO (1.4 equiv.), r.t.	30Hz/90min	0
6	$NiBr_2$ (10 mol%), L (10 mol%), Dy_2O_3 (1 equiv.), B_2Pin_2 (1.3 equiv.), DABCO (1.4 equiv.), r.t.	30Hz/90min	71
**7**	**NiI_2 (10 mol%), L (10 mol%), Dy_2O_3 (1 equiv.), B_2Pin_2 (1.3 equiv.), DABCO (1.4 equiv.), r.t.**	**30Hz/90min**	**83**
8	NiI_2 (10 mol%), L (10 mol%), Dy_2O_3 (1 equiv.), B_2Pin_2 (1.3 equiv.), Na_2CO_3 (1.4 equiv.), r.t.	30Hz/90min	10

[a] Isolated yield.

Table S4. Optimization of the reaction conditions.

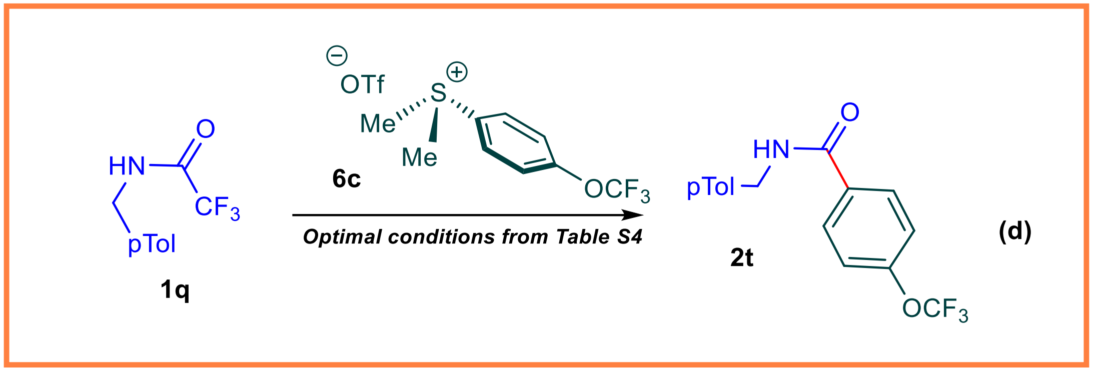

Reaction (d)			
entry	reaction components	milling frequency/time	yield (%) **2t**[a]
1	NiI_2 (10 mol%), L (10 mol%), Dy_2O_3 (1 equiv.), B_2Pin_2 (1.3 equiv.), DABCO (1.4 equiv.), r.t.	30Hz/90min	86
**2**	**NiI_2 (10 mol%), L (10 mol%), Dy_2O_3 (1 equiv.), B_2Pin_2 (1.2 equiv.), DABCO (1.4 equiv.), r.t.**	**30Hz/90min**	**86**
3	NiI_2 (10 mol%), L (10 mol%), Dy_2O_3 (1 equiv.), B_2Pin_2 (1.2 equiv.), Na_2CO_3 (1.4 equiv.), r.t.	30Hz/90min	10

[a] Isolated yield.

Reaction procedures with optimised reaction conditions.

General procedure for the synthesis of amides 2 starting from trifluoroacetamides 1 and boronic acids 3.

In dry box, to 5 mL grinding vessel (made of stainless) equipped with two balls (made of stainless, diameter: 5 mm) was placed consequently $NiBr_2$ (22 mg, 0.1 mmol, 10 mol%), cucurbit[6]uril (**L**) (100 mg, 0.1 mmol, 10 mol%), Dy_2O_3 (373 mg, 1.0 mmol, 1.0 equiv.), DABCO (157 mg, 1.4 mmol, 1.4 equiv.); then appropriate boronic acid **3** (1.2 mmol, 1.2 equiv.) and appropriate trifluoroacetamide **1** (1.0 mmol, 1.0 equiv.) were added and the reaction vessel was properly capped. Finally, the vessel was installed on the mill and subjected to milling at 30Hz for 90 minutes. After completion of the reaction, the content of the vessel was generously treated with distilled water, filtrated and finally properly dried in vacuum. The resulted crude was directly subjected to gradient flash chromatography on silica gel to isolate the desired amide derivative.

The gram scale synthesis was performed on 10 mmol of the starting amide in 25 mL grinding vessel using three 10 mm balls.

General procedure for the synthesis of amides 2 starting from trifluoroacetamides 1 and potassium aryltrifluoroborates.

In dry box, to 5 mL grinding vessel (made of stainless) equipped with two balls (made of stainless, diameter: 5 mm) was placed consequently $NiBr_2$ (22 mg, 0.1 mmol, 10 mol%), cucurbit[6]uril (**L**) (100 mg, 0.1 mmol, 10 mol%), Dy_2O_3 (373 mg, 1.0 mmol, 1.0 equiv.), DABCO (157 mg, 1.4 mmol, 1.4 equiv.); then potassium aryltrifluoroborate (1.2 mmol, 1.2 equiv.) and appropriate trifluoroacetamide **1** (1.0 mmol, 1.0 equiv.) were added and the reaction vessel was properly capped. Finally, the vessel was installed on the mill and subjected to milling at 30Hz for 90 minutes. After completion of the reaction, the content of the vessel was generously treated with distilled water, filtrated and finally properly dried in vacuum. The resulted crude was directly subjected to gradient flash chromatography on silica gel to isolate the desired amide derivative.

The gram scale synthesis was performed on 10 mmol of the starting amide in 25 mL grinding vessel using three 10 mm balls.

General procedure for the synthesis of amides 2 starting from trifluoroacetamides 1 aryl pinacol borates.

In dry box, to 5 mL grinding vessel (made of stainless) equipped with two balls (made of stainless, diameter: 5 mm) was placed consequently $NiBr_2$ (22 mg, 0.1 mmol, 10 mol%), cucurbit[6]uril (**L**) (100 mg, 0.1 mmol, 10 mol%), Dy_2O_3 (373 mg, 1.0 mmol, 1.0 equiv.), DABCO (157 mg, 1.4 mmol, 1.4 equiv.); then appropriate aryl pinacol borate (1.2 mmol, 1.2 equiv.) and appropriate trifluoroacetamide **1** (1.0 mmol, 1.0 equiv.) were added and the reaction vessel was properly capped. Finally, the vessel was installed on the mill and subjected to milling at 30Hz for 90 minutes. After completion of the reaction, the content of the vessel was generously treated with distilled water, filtrated and finally properly dried in vacuum. The resulted crude was directly subjected to gradient flash chromatography on silica gel to isolate the desired amide derivative.

The gram scale synthesis was performed on 10 mmol of the starting amide in 25 mL grinding vessel using three 10 mm balls.

General procedure for the synthesis of amides 2 starting from trifluoroacetamides 1 and aryl trialkoxysilanes 4.

In dry box, to 5 mL grinding vessel (made of stainless) equipped with two balls (made of stainless, diameter: 5 mm) was placed consequently $NiBr_2$ (22 mg, 0.1 mmol, 10 mol%), cucurbit[6]uril (**L**) (100 mg, 0.1 mmol, 10 mol%), Dy_2O_3 (373 mg, 1.0 mmol, 1.0 equiv.), DABCO (157 mg, 1.4 mmol, 1.4 equiv.); then appropriate trialkoxysilane **4** (1.2 mmol, 1.2 equiv.) and appropriate trifluoroacetamide **1** (1.0 mmol, 1.0 equiv.) were added and the reaction vessel was properly capped. Finally, the vessel was installed on the mill and subjected to milling at 30Hz for 90 minutes. After completion of the reaction, the content of the vessel was generously treated with distilled water, filtrated and finally properly dried in vacuum. The resulted crude was directly subjected to gradient flash chromatography on silica gel to isolate the desired amide derivative.

The gram scale synthesis was performed on 10 mmol of the starting amide in 25 mL grinding vessel using three 10 mm balls.

General procedure for the synthesis of amides 2 starting from trifluoroacetamides 1 and iodonium salts 5.

In dry box, to 5 mL grinding vessel (made of stainless) equipped with two balls (made of stainless, diameter: 5 mm) was placed consequently NiI_2 (31 mg, 0.1 mmol, 10 mol%), cucurbit[6]uril (**L**) (100 mg, 0.1 mmol, 10 mol%), Dy_2O_3 (373 mg, 1.0 mmol, 1.0 equiv.), DABCO (157 mg, 1.4 mmol, 1.4 equiv.), bis(pinacolato)diborane (330 mg, 1.3 mmol, 1.3 equiv.); then appropriate iodonium salt **5** (0.7 mmol, 0.7 equiv.) and appropriate trifluoroacetamide **1** (1.0 mmol, 1.0 equiv.) were added and the reaction vessel was properly capped. Finally, the vessel was installed on the mill and subjected to milling at 30Hz for 90 minutes. After completion of the reaction, the content of the vessel was generously treated with distilled water, filtrated and finally properly dried in vacuum. The resulted crude was directly subjected to gradient flash chromatography on silica gel to isolate the desired amide derivative.

The gram scale synthesis was performed on 10 mmol of the starting amide in 25 mL grinding vessel using three 10 mm balls.

General procedure for the synthesis of amides 2 starting from trifluoroacetamides 1 and sulphonium salts 6.

In dry box, to 5 mL grinding vessel (made of stainless) equipped with two balls (made of stainless, diameter: 5 mm) was placed consequently NiI_2 (31 mg, 0.1 mmol, 10 mol%), cucurbit[6]uril (**L**) (100 mg, 0.1 mmol, 10 mol%), Dy_2O_3 (373 mg, 1.0 mmol, 1.0 equiv.), DABCO (157 mg, 1.4 mmol, 1.4 equiv.), bis(pinacolato)diborane (305 mg, 1.2 mmol, 1.2 equiv.); then appropriate sulphonium salt **6** (1.2 mmol, 1.2 equiv.) and appropriate trifluoroacetamide **1** (1.0 mmol, 1.0 equiv.) were added and the reaction vessel was properly capped. Finally, the vessel was installed on the mill and subjected to milling at 30Hz for 90 minutes. After completion of the reaction, the content of the vessel was generously treated with distilled water, filtrated and finally properly dried in vacuum. The resulted crude was directly subjected to gradient flash chromatography on silica gel to isolate the desired amide derivative.

The gram scale synthesis was performed on 10 mmol of the starting amide in 25 mL grinding vessel using three 10 mm balls.

(B) Characterization of products.

3,4,5-trifluoro-N-phenylbenzamide (2a).

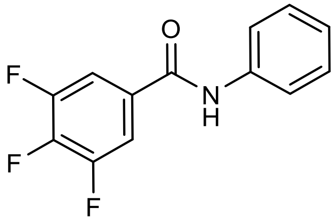

The title compound was prepared starting from $NiBr_2$ (22 mg, 0.1 mmol, 10 mol%), cucurbit[6]uril (**L**) (100 mg, 0.1 mmol, 10 mol%), Dy_2O_3 (373 mg, 1.0 mmol, 1.0 equiv.), DABCO (157 mg, 1.4 mmol, 1.4 equiv.), boronic acid **3j** (211 mg, 1.2 mmol, 1.2 equiv.) and trifluoroacetamide **1a** (189 mg, 1.0 mmol, 1.0 equiv.). The purification was accomplished by column chromatography on silica gel with Hexane/Ethyl acetate 2:1 as eluent to provide the desired amide **2a** (208 mg, 0.83 mmol, 83%).

White solid, mp 161 - 162 ^oC. **^{1}H NMR** (500 MHz, DMSO-*d*$_6$): δ = 10.29 (s, 1H), 7.88 (dd, ^3J=8.6 Hz, ^4J=6.9 Hz, 2H), 7.71 (d, ^3J=7.7 Hz, 2H), 7.32 (t, ^3J=7.9 Hz, 2H), 7.09 (t, ^3J=7.4 Hz, 1H).

^{13}C{^{1}H} NMR (126 MHz, DMSO-*d*$_6$): δ= 162.0, 150.0 (ddd, $^1J_{CF}$ = 247.1 Hz, J_{CF} =9.6, J_{CF} = 2.8), 140.8 (dt, $^1J_{CF}$ = 252.7 Hz, J_{CF} = 16.5), 138.6, 131.5 (m), 128.7, 124.2, 120.5, 112.9 (dd, J_{CF} = 17.5 Hz, J_{CF} = 4.9).

MS (GC, 70eV): m/z (%) = 251 (M^+, 3.7), 159 (100), 131 (36).

Anal. calcd. for $C_{13}H_8NOF_3$: C, 62.16; H, 3.21; N, 5.58. Found: C, 62.29; H, 3.19, N, 5.52.

4-fluoro-N-(3-fluoro-4-methoxyphenyl)-2-(trifluoromethyl)benzamide (2b).

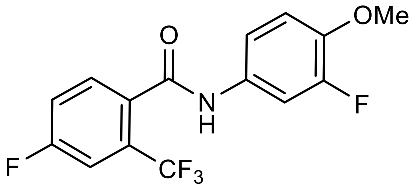

The title compound was prepared starting from $NiBr_2$ (22 mg, 0.1 mmol, 10 mol%), cucurbit[6]uril (**L**) (100 mg, 0.1 mmol, 10 mol%), Dy_2O_3 (373 mg, 1.0 mmol, 1.0 equiv.), DABCO (157 mg, 1.4 mmol, 1.4 equiv.), boronic acid **3s** (250 mg, 1.2 mmol, 1.2 equiv.) and trifluoroacetamide **1g** (237 mg, 1.0 mmol, 1.0 equiv.). The purification was accomplished by column chromatography on silica gel with Hexane/Ethyl acetate 2:1 as eluent to provide the desired amide **2b** (164 mg, 0.71 mmol, 71%).

White solid, mp 140 - 141 °C. **^{1}H NMR** (500 MHz, DMSO-d_6): δ = 10.61 (s, 1H), 7.84 – 7.77 (m, 2H), 7.71 – 7.61 (m, 2H), 7.38 (d, ^3J =8.9 Hz, 1H), 7.16 (t, ^3J =9.3 Hz, 1H), 3.82 (s, 3H).

^{13}C{^{1}H} NMR (126 MHz, DMSO-d_6): δ = 164.5, 162.0 (d, $^1J_{CF}$ = 248.7 Hz), 150.9 (d, $^1J_{CF}$ = 242.6 Hz), 143.4 (d, J_{CF} = 10.4 Hz), 132.7, 132.1 (d, J_{CF} = 8.9 Hz), 131.4 (d, J_{CF} = 8.5 Hz), 128.3 (m), 122.9 (q, $^1J_{CF}$ = 273.2 Hz), 119.5 (d, J_{CF} = 19.9 Hz), 115.6, 114.3 (m), 114.1, 108.0 (d, J_{CF} = 22.9 Hz), 56.2.

HRMS (TOF MS ES+) m/z: $[M + H]^+$: Calcd for $C_{15}H_{11}NO_2F_5$ (M+H) 332.0715. Found 332.0710.

2-methyl-N-(3-(trifluoromethyl)phenyl)benzamide (2c).

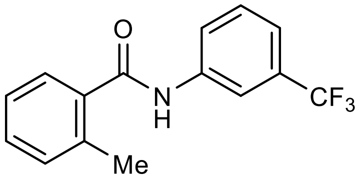

The title compound was prepared starting from $NiBr_2$ (22 mg, 0.1 mmol, 10 mol%), cucurbit[6]uril (**L**) (100 mg, 0.1 mmol, 10 mol%), Dy_2O_3 (373 mg, 1.0 mmol, 1.0 equiv.), DABCO (157 mg, 1.4 mmol, 1.4 equiv.), boronic acid **3a** (163 mg, 1.2 mmol, 1.2 equiv.) and trifluoroacetamide **1b** (257 mg, 1.0 mmol, 1.0 equiv.). The purification was accomplished by column chromatography on silica gel with Hexane/Ethyl acetate 3:1 as eluent to provide the desired amide **2c** (195 mg, 0.70 mmol, 70%).

Alternatively, the title compound was prepared starting from $NiBr_2$ (22 mg, 0.1 mmol, 10 mol%), cucurbit[6]uril (**L**) (100 mg, 0.1 mmol, 10 mol%), Dy_2O_3 (373 mg, 1.0 mmol, 1.0 equiv.), DABCO (157 mg, 1.4 mmol, 1.4 equiv.), trialkoxysilane **4a** (254 mg, 1.2 mmol, 1.2 equiv.) and trifluoroacetamide **1b** (257 mg, 1.0 mmol, 1.0 equiv.). The purification was accomplished by column chromatography on silica gel with Hexane/Ethyl acetate 3:1 as eluent to provide the desired amide **2c** (190 mg, 0.68 mmol, 68%).

Alternatively, the title compound was prepared starting from NiI_2 (31 mg, 0.1 mmol, 10 mol%), cucurbit[6]uril (**L**) (100 mg, 0.1 mmol, 10 mol%), Dy_2O_3 (373 mg, 1.0 mmol, 1.0 equiv.), DABCO (157 mg, 1.4 mmol, 1.4 equiv.), bis(pinacolato)diborane (330 mg, 1.3 mmol, 1.3 equiv.), iodonium salt **5a** (338 mg, 0.7 mmol, 0.7 equiv.) and trifluoroacetamide **1b** (257 mg, 1.0 mmol, 1.0 equiv.). The purification was accomplished by column chromatography on silica gel with Hexane/Ethyl acetate 3:1 as eluent to provide the desired amide **2c** (159 mg, 0.57 mmol, 57%).

Alternatively, the title compound was prepared starting from NiI_2 (31 mg, 0.1 mmol, 10 mol%), cucurbit[6]uril (**L**) (100 mg, 0.1 mmol, 10 mol%), Dy_2O_3 (373 mg, 1.0 mmol, 1.0 equiv.), DABCO (157 mg, 1.4 mmol, 1.4 equiv.), bis(pinacolato)diborane (305 mg, 1.2 mmol, 1.2 equiv.); then appropriate sulphonium salt **6a** (362 mg, 1.2 mmol, 1.2 equiv.) and trifluoroacetamide **1b** (257 mg, 1.0 mmol, 1.0 equiv.). The purification was accomplished by column chromatography on silica gel with Hexane/Ethyl acetate 3:1 as eluent to provide the desired amide **2c** (162 mg, 0.58 mmol, 58%).

Alternatively, the title compound was prepared starting from $NiBr_2$ (22 mg, 0.1 mmol, 10 mol%), cucurbit[6]uril (**L**) (100 mg, 0.1 mmol, 10 mol%), Dy_2O_3 (373 mg, 1.0 mmol, 1.0 equiv.), DABCO (157 mg, 1.4 mmol, 1.4 equiv.), potassium trifluoro(o-tolyl)borate (238 mg, 1.2 mmol, 1.2 equiv.) and trifluoroacetamide **1b** (257 mg, 1.0 mmol, 1.0 equiv.). The purification was accomplished by column chromatography on silica gel with Hexane/Ethyl acetate 3:1 as eluent to provide the desired amide **2c** (165 mg, 0.59 mmol, 59%).

Alternatively, the title compound was prepared starting from $NiBr_2$ (22 mg, 0.1 mmol, 10 mol%), cucurbit[6]uril (**L**) (100 mg, 0.1 mmol, 10 mol%), Dy_2O_3 (373 mg, 1.0 mmol, 1.0 equiv.), DABCO (157 mg, 1.4 mmol, 1.4 equiv.), 4,4,5,5-tetramethyl-2-(o-tolyl)-1,3,2-dioxaborolane (262 mg, 1.2 mmol, 1.2 equiv.) and trifluoroacetamide **1b** (257 mg, 1.0 mmol, 1.0 equiv.). The purification was accomplished by column chromatography on silica gel with Hexane/Ethyl acetate 3:1 as eluent to provide the desired amide **2c** (162 mg, 0.59 mmol, 58%).

White solid, mp 129 - 131 °C. **^{1}H NMR** (500 MHz, $CDCl_3$): δ = 7.99 (s, 1H), 7.90 (s, 1H), 7.78 (d, ^3J=7.6 Hz, 1H), 7.44 (t, ^3J=7.9 Hz, 1H), 7.39 (d, ^3J=7.8 Hz, 2H), 7.33 (dt, ^3J=7.5 Hz, ^4J=3.8 Hz, 1H), 7.21 (dd, ^3J=13.3 Hz, ^4J=7.6 Hz, 2H), 2.43 (s, 3H).

^{13}C{^{1}H} NMR (126 MHz, $CDCl_3$): δ= 168.4, 138.5, 136.4, 135.7, 131.3, 131.2 (q, $^2J_{CF}$ = 32.9 Hz), 130.5, 129.62, 126.5, 125.9, 123.8 (q, $^1J_{CF}$ = 272.9 Hz), 123.0, 121.0 (m), 116.6, 19.7.

HRMS (TOF MS ES+) m/z: $[M + H]^+$: Calcd for $C_{15}H_{13}NOF_3$ (M+H) 280.0948. Found 280.0938.

N-(4-chlorophenyl)-4-(trifluoromethoxy)benzamide (2d).

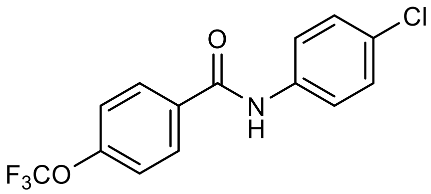

The title compound was prepared starting from $NiBr_2$ (22 mg, 0.1 mmol, 10 mol%), cucurbit[6]uril (**L**) (100 mg, 0.1 mmol, 10 mol%), Dy_2O_3 (373 mg, 1.0 mmol, 1.0 equiv.), DABCO (157 mg, 1.4 mmol, 1.4 equiv.), boronic acid **3q** (247 mg, 1.2 mmol, 1.2 equiv.) and trifluoroacetamide **1d** (223 mg, 1.0 mmol, 1.0 equiv.). The purification was accomplished by column chromatography on silica gel with Hexane/Ethyl acetate 3:1 as eluent to provide the desired amide **2d** (284 mg, 0.90 mmol, 90%). The gram scale synthesis was performed on 10 mmol of the starting **1d** and the aromatic Amide **2d** was prepared in 83% yield (2.61 g, 8.3 mmol).

Alternatively, the title compound was prepared starting from $NiBr_2$ (22 mg, 0.1 mmol, 10 mol%), cucurbit[6]uril (**L**) (100 mg, 0.1 mmol, 10 mol%), Dy_2O_3 (373 mg, 1.0 mmol, 1.0 equiv.), DABCO (157 mg, 1.4 mmol, 1.4 equiv.), trialkoxysilane **4k** (338 mg, 1.2 mmol, 1.2 equiv.) and trifluoroacetamide **1d** (223 mg, 1.0 mmol, 1.0 equiv.). The purification was accomplished by column chromatography on silica gel with Hexane/Ethyl acetate 3:1 as eluent to provide the desired amide **2d** (288 mg, 0.91 mmol, 91%). The gram scale synthesis was performed on 10 mmol of the starting **1d** and the aromatic Amide **2d** was prepared in 81% yield (2.55 g, 8.1 mmol).

Alternatively, the title compound was prepared starting from NiI_2 (31 mg, 0.1 mmol, 10 mol%), cucurbit[6]uril (**L**) (100 mg, 0.1 mmol, 10 mol%), Dy_2O_3 (373 mg, 1.0 mmol, 1.0 equiv.), DABCO (157 mg, 1.4 mmol, 1.4 equiv.), bis(pinacolato)diborane (330 mg, 1.3 mmol, 1.3 equiv.), iodonium salt **5f** (437 mg, 0.7 mmol, 0.7 equiv.) and trifluoroacetamide **1d** (223 mg, 1.0 mmol, 1.0 equiv.). The purification was accomplished by column chromatography on silica gel with Hexane/Ethyl acetate 3:1 as eluent to provide the desired amide **2d** (249 mg, 0.79 mmol, 79%). The gram scale synthesis was performed on 10 mmol of the starting **1d** and the aromatic Amide **2d** was prepared in 73% yield (2.30 g, 7.3 mmol).

White solid, mp 189 - 191 ^oC. ^{1}**H NMR** (500 MHz, $CDCl_3$): δ = 7.89 (d, ^3J=8.6 Hz, 1H), 7.85 (s, 1H), 7.58 (d, ^3J=8.8 Hz, 1H), 7.32 (dd, ^3J=11.0 Hz, ^4J=8.7 Hz, 2H).

^{13}C{^{1}H} NMR (126 MHz, $CDCl_3$): δ= 164.4, 151.8, 136.1, 133.0, 129.9, 129.2, 129.0, 121.5, 120.9, 120.4 (q, $^1J_{CF}$ = 288.0 Hz).

HRMS (TOF MS ES+) m/z: $[M + H]^+$: Calcd for $C_{14}H_{10}NO_2F_3Cl$ (M+H) 316.0340. Found 316.0352.

N-(2-bromophenyl)-4-(trifluoromethoxy)benzamide (2e).

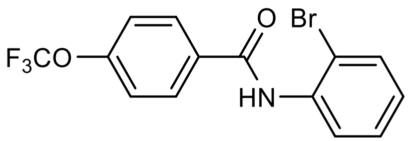

The title compound was prepared starting from $NiBr_2$ (22 mg, 0.1 mmol, 10 mol%), cucurbit[6]uril (**L**) (100 mg, 0.1 mmol, 10 mol%), Dy_2O_3 (373 mg, 1.0 mmol, 1.0 equiv.), DABCO (157 mg, 1.4 mmol, 1.4 equiv.), boronic acid **3q** (247 mg, 1.2 mmol, 1.2 equiv.) and trifluoroacetamide **1e** (267 mg, 1.0 mmol, 1.0 equiv.). The purification was accomplished by column chromatography on silica gel with Hexane/Ethyl acetate 3:1 as eluent to provide the desired amide **2e** (277 mg, 0.77 mmol, 77%).

Alternatively, the title compound was prepared starting from $NiBr_2$ (22 mg, 0.1 mmol, 10 mol%), cucurbit[6]uril (**L**) (100 mg, 0.1 mmol, 10 mol%), Dy_2O_3 (373 mg, 1.0 mmol, 1.0 equiv.), DABCO (157 mg, 1.4 mmol, 1.4 equiv.), trialkoxysilane **4k** (338 mg, 1.2 mmol, 1.2 equiv.) and trifluoroacetamide **1e** (267 mg, 1.0 mmol, 1.0 equiv.). The purification was accomplished by column chromatography on silica gel with Hexane/Ethyl acetate 3:1 as eluent to provide the desired amide **2e** (288 mg, 0.80 mmol, 80%).

Alternatively, the title compound was prepared starting from NiI_2 (31 mg, 0.1 mmol, 10 mol%), cucurbit[6]uril (**L**) (100 mg, 0.1 mmol, 10 mol%), Dy_2O_3 (373 mg, 1.0 mmol, 1.0 equiv.), DABCO (157 mg, 1.4 mmol, 1.4 equiv.), bis(pinacolato)diborane (330 mg, 1.3 mmol, 1.3 equiv.), iodonium salt **5f** (437 mg, 0.7 mmol, 0.7 equiv.) and trifluoroacetamide **1e** (267 mg, 1.0 mmol, 1.0 equiv.). The purification was accomplished by column chromatography on silica gel with Hexane/Ethyl acetate 3:1 as eluent to provide the desired amide **2e** (266 mg, 0.74 mmol, 74%).

White solid, mp 121 - 122 °C. **^{1}H NMR** (500 MHz, $CDCl_3$): δ= 8.51 (d, ^3J = 8.2 Hz, 1H), 8.41 (s, 1H), 7.98 (d, ^3J = 8.6 Hz, 2H), 7.58 (d, ^3J = 8.0 Hz, 1H), 7.37 (dd, ^3J = 15.4 Hz, ^3J = 8.1 Hz, 3H), 7.07 – 7.00 (m, 1H).

^{13}C{^{1}H} NMR (126 MHz, $CDCl_3$): δ= 163.9, 152.0, 135.5, 132.9, 132.3, 129.1, 128.6, 125.6, 121.8, 120.9, 120.3 (q, $^1J_{CF}$ = 258.6 Hz), 119.3, 113.8.

HRMS (TOF MS ES+) m/z: $[M + H]^+$: Calcd for $C_{14}H_{10}NO_2F_3Br$ (M+H) 359.9866. Found 359.9847.

***N*-(naphthalen-1-yl)-2-(trifluoromethoxy)benzamide (2f).**

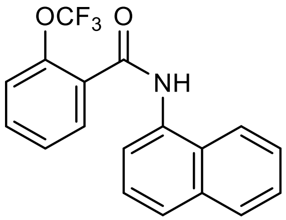

The title compound was prepared starting from $NiBr_2$ (22 mg, 0.1 mmol, 10 mol%), cucurbit[6]uril (**L**) (100 mg, 0.1 mmol, 10 mol%), Dy_2O_3 (373 mg, 1.0 mmol, 1.0 equiv.), DABCO (157 mg, 1.4 mmol, 1.4 equiv.), boronic acid **3o** (247 mg, 1.2 mmol, 1.2 equiv.) and trifluoroacetamide **1j** (239 mg, 1.0 mmol, 1.0 equiv.). The purification was accomplished by column chromatography on silica gel with Hexane/Ethyl acetate 3:1 as eluent to provide the desired amide **2f** (245 mg, 0.74 mmol, 74%).

Alternatively, the title compound was prepared starting from $NiBr_2$ (22 mg, 0.1 mmol, 10 mol%), cucurbit[6]uril (**L**) (100 mg, 0.1 mmol, 10 mol%), Dy_2O_3 (373 mg, 1.0 mmol, 1.0 equiv.), DABCO (157 mg, 1.4 mmol, 1.4 equiv.), trialkoxysilane **4i** (338 mg, 1.2 mmol, 1.2 equiv.) and trifluoroacetamide **1j** (239 mg, 1.0 mmol, 1.0 equiv.). The purification was accomplished by column chromatography on silica gel with Hexane/Ethyl acetate 3:1 as eluent to provide the desired amide **2f** (235 mg, 0.71 mmol, 71%).

Alternatively, the title compound was prepared starting from NiI_2 (31 mg, 0.1 mmol, 10 mol%), cucurbit[6]uril (**L**) (100 mg, 0.1 mmol, 10 mol%), Dy_2O_3 (373 mg, 1.0 mmol, 1.0 equiv.), DABCO (157 mg, 1.4 mmol, 1.4 equiv.), bis(pinacolato)diborane (330 mg, 1.3 mmol, 1.3 equiv.), iodonium salt **5f** (437 mg, 0.7 mmol, 0.7 equiv.) and trifluoroacetamide **1j** (239 mg, 1.0 mmol, 1.0 equiv.). The purification was accomplished by column chromatography on silica gel with Hexane/Ethyl acetate 3:1 as eluent to provide the desired amide **2f** (232 mg, 0.70 mmol, 70%).

White solid, mp 154 - 155 ^oC. **^{1}H NMR** (500 MHz, $CDCl_3$): δ = 8.69 (s, 1H), 8.21 – 8.09 (m, 2H), 7.98 – 7.87 (m, 2H), 7.76 (d, ^3J=8.2 Hz, 1H), 7.61 – 7.51 (m, 4H), 7.48 (t, ^3J=7.5 Hz, 1H), 7.42 (d, ^3J=8.2 Hz, 1H).

^{13}C{^{1}H} NMR (126 MHz, $CDCl_3$): δ= 162.9, 145.9, 134.1, 132.8, 132.2, 132.1, 128.8, 128.1, 127.6, 127.1, 126.5, 126.2, 126.1, 125.8, 120.4 (q, $^1J_{CF}$ = 260.5 Hz), 121.0, 120.9, 120.5.

HRMS (TOF MS ES+) m/z: $[M + H]^+$: Calcd for $C_{18}H_{13}NO_2F_3$ (M+H) 332.0906. Found 332.0898.

N,N-di-p-tolyl-4-(trifluoromethoxy)benzamide (2g).

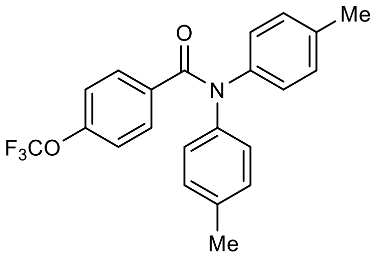

The title compound was prepared starting from $NiBr_2$ (22 mg, 0.1 mmol, 10 mol%), cucurbit[6]uril (**L**) (100 mg, 0.1 mmol, 10 mol%), Dy_2O_3 (373 mg, 1.0 mmol, 1.0 equiv.), DABCO (157 mg, 1.4 mmol, 1.4 equiv.), boronic acid **3q** (247 mg, 1.2 mmol, 1.2 equiv.) and trifluoroacetamide **1i** (293 mg, 1.0 mmol, 1.0 equiv.). The purification was accomplished by column chromatography on silica gel with Hexane/Ethyl acetate 3:1 as eluent to provide the desired amide **2g** (346 mg, 0.90 mmol, 90%).

Alternatively, the title compound was prepared starting from $NiBr_2$ (22 mg, 0.1 mmol, 10 mol%), cucurbit[6]uril (**L**) (100 mg, 0.1 mmol, 10 mol%), Dy_2O_3 (373 mg, 1.0 mmol, 1.0 equiv.), DABCO (157 mg, 1.4 mmol, 1.4 equiv.), trialkoxysilane **4l** (338 mg, 1.2 mmol, 1.2 equiv.) and trifluoroacetamide **1i** (293 mg, 1.0 mmol, 1.0 equiv.). The purification was accomplished by column chromatography on silica gel with Hexane/Ethyl acetate 3:1 as eluent to provide the desired amide **2f** (235 mg, 0.87 mmol, 87%).

Alternatively, the title compound was prepared starting from NiI_2 (31 mg, 0.1 mmol, 10 mol%), cucurbit[6]uril (**L**) (100 mg, 0.1 mmol, 10 mol%), Dy_2O_3 (373 mg, 1.0 mmol, 1.0 equiv.), DABCO (157 mg, 1.4 mmol, 1.4 equiv.), bis(pinacolato)diborane (330 mg, 1.3 mmol, 1.3 equiv.), iodonium salt **5e** (437 mg, 0.7 mmol, 0.7 equiv.) and trifluoroacetamide **1j** (239 mg, 1.0 mmol, 1.0 equiv.). The purification was accomplished by column chromatography on silica gel with Hexane/Ethyl acetate 3:1 as eluent to provide the desired amide **2g** (312 mg, 0.81 mmol, 81%).

White solid, mp 94 - 95 °C. **^{1}H NMR** (500 MHz, $CDCl_3$): δ = 7.53 – 7.47 (m, 2H), 7.10 (d, *J*=7.7 Hz, 4H), 7.05 (d, *J*=8.7 Hz, 6H), 2.32 (s, 6H).

^{13}C{^{1}H} NMR (126 MHz, $CDCl_3$): δ= 169.1, 150.0, 141.1, 136.4, 134.7, 130.9, 129.8, 127.1, 120.2 (q, $^1J_{CF}$ = 257.4 Hz), 119.9, 21.0.

HRMS (TOF MS ES+) m/z: $[M + H]^+$: Calcd for $C_{22}H_{19}NO_2F_3$ (M+H) 386.1363. Found 386.1368.

N-(3,5-bis(trifluoromethyl)phenyl)-3-chlorobenzamide (2h).

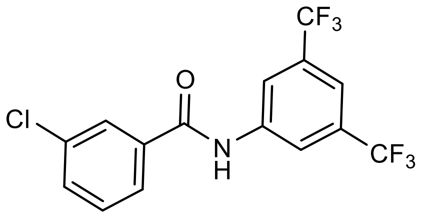

The title compound was prepared starting from $NiBr_2$ (22 mg, 0.1 mmol, 10 mol%), cucurbit[6]uril (**L**) (100 mg, 0.1 mmol, 10 mol%), Dy_2O_3 (373 mg, 1.0 mmol, 1.0 equiv.), DABCO (157 mg, 1.4 mmol, 1.4 equiv.), boronic acid **3e** (187 mg, 1.2 mmol, 1.2 equiv.) and trifluoroacetamide **1c** (325 mg, 1.0 mmol, 1.0 equiv.). The purification was accomplished by column chromatography on silica gel with Hexane/Ethyl acetate 3:1 as eluent to provide the desired amide **2h** (305 mg, 0.83 mmol, 83%).

Alternatively, the title compound was prepared starting from $NiBr_2$ (22 mg, 0.1 mmol, 10 mol%), cucurbit[6]uril (**L**) (100 mg, 0.1 mmol, 10 mol%), Dy_2O_3 (373 mg, 1.0 mmol, 1.0 equiv.), DABCO (157 mg, 1.4 mmol, 1.4 equiv.), trialkoxysilane **4e** (278 mg, 1.2 mmol, 1.2 equiv.) and trifluoroacetamide **1c** (325 mg, 1.0 mmol, 1.0 equiv.). The purification was accomplished by column chromatography on silica gel with Hexane/Ethyl acetate 3:1 as eluent to provide the desired amide **2h** (330 mg, 0.90 mmol, 90%).

Alternatively, the title compound was prepared starting from NiI_2 (31 mg, 0.1 mmol, 10 mol%), cucurbit[6]uril (**L**) (100 mg, 0.1 mmol, 10 mol%),Dy_2O_3 (373 mg, 1.0 mmol, 1.0 equiv.), DABCO (157 mg, 1.4 mmol, 1.4 equiv.), bis(pinacolato)diborane (330 mg, 1.3 mmol, 1.3 equiv.), iodonium salt **5b** (375 mg, 0.7 mmol, 0.7 equiv.) and trifluoroacetamide **1c** (325 mg, 1.0 mmol, 1.0 equiv.). The purification was accomplished by column chromatography on silica gel with Hexane/Ethyl acetate 3:1 as eluent to provide the desired amide **2h** (291 mg, 0.79 mmol, 79%).

White solid, mp 179 - 180 ^{o}C. **^{1}H NMR** (500 MHz, DMSO-*d$_6$*): δ= 10.83 (s, 1H), 8.45 (s, 2H), 8.00 (s, 1H), 7.90 (d, ^{3}J = 7.8 Hz, 1H), 7.73 (s, 1H), 7.64 (dd, ^{3}J = 8.0 Hz, ^{4}J = 1.0 Hz, 1H), 7.54 (t, ^{3}J = 7.9 Hz, 1H).

^{13}C{^{1}H} NMR (126 MHz, DMSO-*d$_6$*): δ= 164.7, 140.9, 135.8, 133.4, 132.0, 130.7 (q, $^{2}J_{CF}$ = 31.2 Hz), 130.5, 127.5, 126.6, 123.3 (q, $^{1}J_{CF}$ = 273.4 Hz), 119.9, 116.5 (m).

MS (GC, 70eV): m/z (%) = 367 (M^+, 12), 139 (100), 111 (41).

Anal. calcd. for $C_{15}H_8NOF_6Cl$: C, 49.00; H, 2.19; N, 3.81. Found: C, 49.08; H, 2.21, N, 3.79.

N-(4-chloro-2-methylphenyl)-3,5-bis(trifluoromethyl)benzamide (2i).

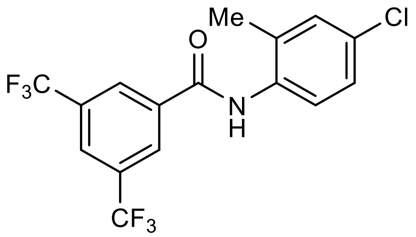

The title compound was prepared starting from $NiBr_2$ (22 mg, 0.1 mmol, 10 mol%), cucurbit[6]uril (**L**) (100 mg, 0.1 mmol, 10 mol%), Dy_2O_3 (373 mg, 1.0 mmol, 1.0 equiv.), DABCO (157 mg, 1.4 mmol, 1.4 equiv.), boronic acid **3n** (310 mg, 1.2 mmol, 1.2 equiv.) and trifluoroacetamide **1f** (237 mg, 1.0 mmol, 1.0 equiv.). The purification was accomplished by column chromatography on silica gel with Hexane/Ethyl acetate 3:1 as eluent to provide the desired amide **2i** (320 mg, 0.84 mmol, 84%).

White solid, mp 187 - 188 °C. **^{1}H NMR** (500 MHz, DMSO-d$_6$): δ= 10.40 (s, 1H), 8.59 (s, 2H), 8.30 (s, 1H), 7.34 (dd, ^3J=5.4 Hz, ^4J=2.7 Hz, 2H), 7.25 (dd, ^3J=8.5 Hz, ^4J=2.2 Hz, 1H), 2.21 (s, 3H).

^{13}C{^{1}H} NMR (126 MHz, DMSO-d$_6$): δ= 162.6, 136.6, 136.4, 134.8, 130.6 (q, $^2J_{CF}$ = 34.1 Hz), 130.5, 130.1, 128.6, 128.3, 126.0, 125.1, 123.1 (q, J_{CF} = 273.7 Hz), 17.7.

HRMS (TOF MS ES+) m/z: $[M + H]^+$: Calcd for $C_{16}H_{11}NOF_6Cl$ (M+H) 382.0432. Found 382.0433.

N-(4-iodophenyl)-2-methoxybenzamide (2j).

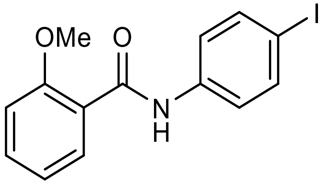

The title compound was prepared starting from $NiBr_2$ (22 mg, 0.1 mmol, 10 mol%), cucurbit[6]uril (**L**) (100 mg, 0.1 mmol, 10 mol%), Dy_2O_3 (373 mg, 1.0 mmol, 1.0 equiv.), DABCO (157 mg, 1.4 mmol, 1.4 equiv.), boronic acid **3c** (182 mg, 1.2 mmol, 1.2 equiv.) and trifluoroacetamide **1k** (315 mg, 1.0

mmol, 1.0 equiv.). The purification was accomplished by column chromatography on silica gel with Hexane/Ethyl acetate 3:1 as eluent to provide the desired amide **2j** (187 mg, 0.53 mmol, 53%).

Alternatively, the title compound was prepared starting from $NiBr_2$ (22 mg, 0.1 mmol, 10 mol%), cucurbit[6]uril (**L**) (100 mg, 0.1 mmol, 10 mol%), Dy_2O_3 (373 mg, 1.0 mmol, 1.0 equiv.), DABCO (157 mg, 1.4 mmol, 1.4 equiv.), trialkoxysilane **4c** (274 mg, 1.2 mmol, 1.2 equiv.) and trifluoroacetamide **1k** (315 mg, 1.0 mmol, 1.0 equiv.). The purification was accomplished by column chromatography on silica gel with Hexane/Ethyl acetate 3:1 as eluent to provide the desired amide **2j** (169 mg, 0.48 mmol, 48%).

White solid, mp 125 - 126 °C. **^{1}H NMR** (500 MHz, $CDCl_3$): δ= 9.80 (s, 1H), 8.45 (s, 2H), 8.23 (dd, ^3J = 8.0 Hz, ^4J = 1.4 Hz, 1H), 7.61 (d, ^3J = 8.6 Hz, 2H), 7.47 – 7.44 (m, 3H), 7.10 (t, ^3J = 7.0 Hz, 1H), 6.98 (d, ^3J = 8.0 Hz, 1H).

^{13}C{^{1}H} NMR (126 MHz, $CDCl_3$): δ= 163.2, 157.0, 138.1, 137.7, 133.4, 132.3, 122.1, 121.5, 121.2 111.4, 87.1, 56.2.

HRMS (TOF MS ES+) m/z: $[M + H]^+$: Calcd for $C_{14}H_{13}NO_2I$ (M+H) 353.9998. Found 353.9991.

N-(4-fluorophenyl)-2-nitrobenzamide (2k).

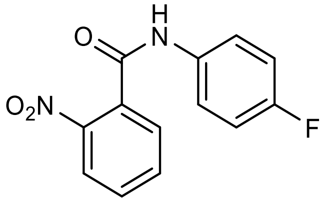

The title compound was prepared starting from $NiBr_2$ (22 mg, 0.1 mmol, 10 mol%), cucurbit[6]uril (**L**) (100 mg, 0.1 mmol, 10 mol%), Dy_2O_3 (373 mg, 1.0 mmol, 1.0 equiv.), DABCO (157 mg, 1.4 mmol, 1.4 equiv.), boronic acid **3v** (200 mg, 1.2 mmol, 1.2 equiv.) and trifluoroacetamide **1l** (207 mg, 1.0 mmol, 1.0 equiv.). The purification was accomplished by column chromatography on silica gel with Hexane/Ethyl acetate 2:1 as eluent to provide the desired amide **2k** (166 mg, 0.64 mmol, 64%).

White solid, mp 159 - 160 °C. **^{1}H NMR** (500 MHz, DMSO-d_6): δ= 10.75 (s, 1H), 8.15 (d, ^3J = 8.0 Hz, 1H), 7.88 (t, ^3J = 7.3 Hz, 1H), 7.80 – 7.75 (m, 2H), 7.73 – 7.70 (m), 7.22 (t, ^3J = 8.9 Hz, 2H).

$^{13}C\{^1H\}$ NMR (126 MHz, DMSO-d_6): δ= 164.1, 158.5 (d, $^1J_{CF}$ = 239.8 Hz), 146.5, 135.2, 134.1, 132.6, 131.1, 129.3, 124.3, 121.5 (d, J_{CF} = 7.8 Hz), 115.5 (d, J_{CF} = 22.2 Hz).

HRMS (TOF MS ES+) m/z: $[M + H]^+$: Calcd for $C_{13}H_{10}N_2O_3F$ (M+H) 261.0678. Found 261.0675.

methyl 3-(4-(trifluoromethyl)benzamido)thiophene-2-carboxylate (2l).

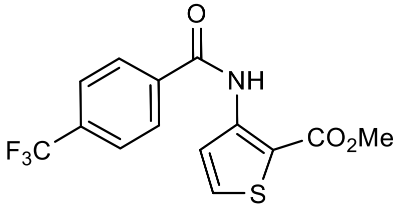

The title compound was prepared starting from $NiBr_2$ (22 mg, 0.1 mmol, 10 mol%), cucurbit[6]uril (**L**) (100 mg, 0.1 mmol, 10 mol%), Dy_2O_3 (373 mg, 1.0 mmol, 1.0 equiv.), DABCO (157 mg, 1.4 mmol, 1.4 equiv.), boronic acid **3m** (228 mg, 1.2 mmol, 1.2 equiv.) and trifluoroacetamide **1m** (253 mg, 1.0 mmol, 1.0 equiv.). The purification was accomplished by column chromatography on silica gel with Hexane/Ethyl acetate 2:1 as eluent to provide the desired amide **2l** (243 mg, 0.74 mmol, 74%).

Alternatively, the title compound was prepared starting from $NiBr_2$ (22 mg, 0.1 mmol, 10 mol%), cucurbit[6]uril (**L**) (100 mg, 0.1 mmol, 10 mol%), Dy_2O_3 (373 mg, 1.0 mmol, 1.0 equiv.), DABCO (157 mg, 1.4 mmol, 1.4 equiv.), trialkoxysilane **4h** (319 mg, 1.2 mmol, 1.2 equiv.) and trifluoroacetamide **1m** (253 mg, 1.0 mmol, 1.0 equiv.). The purification was accomplished by column chromatography on silica gel with Hexane/Ethyl acetate 2:1 as eluent to provide the desired amide **2l** (233 mg, 0.71 mmol, 71%).

White solid, mp 119 - 120 ^oC. **^1H NMR** (500 MHz, $CDCl_3$): δ= 11.26 (s, 1H), 8.26 (d, ^3J = 5.5 Hz, 1H), 8.11 (d, ^3J = 8.2 Hz, 2H), 7.77 (d, ^3J = 8.6 Hz, 2H), 7.54 (d, ^3J = 5.6 Hz, 2H), 3.93 (s, 3H).

$^{13}C\{^1H\}$ NMR (126 MHz, $CDCl_3$): δ= 165.3, 162.8, 144.7, 136.9, 133.8 (q, J_{CF} = 33.4 Hz), 132.1, 127.9, 125.9, 123.6 (q, $^1J_{CF}$ = 273.0 Hz), 122.2, 111.0, 52.2.

HRMS (TOF MS ES+) m/z: $[M + H]^+$: Calcd for $C_{14}H_{11}NO_3F_3S$ (M+H) 330.0419. Found 330.0412.

N-(4-fluoro-3-methoxyphenyl)nicotinamide (2m).

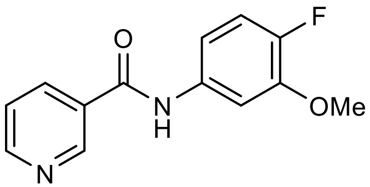

The title compound was prepared starting from $NiBr_2$ (22 mg, 0.1 mmol, 10 mol%), cucurbit[6]uril (**L**) (100 mg, 0.1 mmol, 10 mol%), Dy_2O_3 (373 mg, 1.0 mmol, 1.0 equiv.), DABCO (157 mg, 1.4 mmol, 1.4 equiv.), boronic acid **3w** (148 mg, 1.2 mmol, 1.2 equiv.) and trifluoroacetamide **1h** (237 mg, 1.0 mmol, 1.0 equiv.). The purification was accomplished by column chromatography on silica gel with Hexane/Ethyl acetate 1:1 as eluent to provide the desired amide **2m** (177 mg, 0.72 mmol, 72%).

Alternatively, the title compound was prepared starting from $NiBr_2$ (22 mg, 0.1 mmol, 10 mol%), cucurbit[6]uril (**L**) (100 mg, 0.1 mmol, 10 mol%), Dy_2O_3 (373 mg, 1.0 mmol, 1.0 equiv.), DABCO (157 mg, 1.4 mmol, 1.4 equiv.), trialkoxysilane **4l** (239 mg, 1.2 mmol, 1.2 equiv.) and trifluoroacetamide **1h** (237 mg, 1.0 mmol, 1.0 equiv.). The purification was accomplished by column chromatography on silica gel with Hexane/Ethyl acetate 1:1 as eluent to provide the desired amide **2m** (148 mg, 0.60 mmol, 60%).

White solid, mp 152 - 153 °C. **^{1}H NMR** (500 MHz, DMSO-d_6): δ = 10.42 (s, 1H), 9.06 (d, ^5J=1.7 Hz, 1H), 8.72 (dd, ^3J=4.8 Hz, ^4J=1.5 Hz, 1H), 8.27 – 8.22 (m, 1H), 7.70 (dd, ^3J=13.7 Hz, ^4J=2.4 Hz, 1H), 7.53 (dd, ^3J=7.9 Hz, ^4J=4.9 Hz, 1H), 7.46 (d, ^3J=8.9 Hz, 1H), 7.13 (t, ^3J=9.3 Hz, 1H), 3.79 (s, 3H).

^{13}C{^{1}H} NMR (126 MHz, DMSO-d_6): δ= 163.8, 152.1, 150.8 (d, $^1J_{CF}$ = 244.3 Hz), 148.6, 143.4 (d, J_{CF} = 10.8 Hz), 135.5, 132.2 (d, J_{CF} = 9.2 Hz), 130.4, 123.5, 116.3 (d, J_{CF} = 3.1 Hz), 113.9, 108.7 (d, J_{CF} = 20.8 Hz), 56.1.

HRMS (TOF MS ES+) m/z: [M + H]$^+$: Calcd for $C_{13}H_{12}N_2O_2F$ (M+H) 247.0881. Found 247.0883.

N^1,N^3-bis(3-(trifluoromethyl)phenyl)isophthalamide (2n).

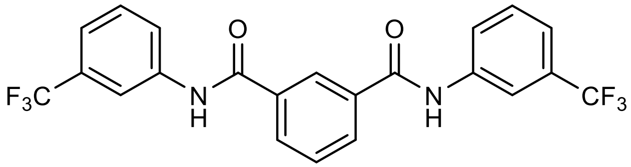

The title compound was prepared starting from $NiBr_2$ (22 mg, 0.1 mmol, 10 mol%), cucurbit[6]uril (**L**) (100 mg, 0.1 mmol, 10 mol%), Dy_2O_3 (373 mg, 1.0 mmol, 1.0 equiv.), DABCO (157 mg, 1.4 mmol, 1.4 equiv.), boronic acid **3x** (166 mg, 1.0 mmol, 1.0 equiv.) and trifluoroacetamide **1b** (591 mg, 2.3 mmol, 2.3 equiv.). The purification was accomplished by column chromatography on silica gel with Hexane/Ethyl acetate 1:1 as eluent to provide the desired amide **2n** (339 mg, 0.75 mmol, 75%).

Alternatively, the title compound was prepared starting from $NiBr_2$ (22 mg, 0.1 mmol, 10 mol%), cucurbit[6]uril (**L**) (100 mg, 0.1 mmol, 10 mol%), Dy_2O_3 (373 mg, 1.0 mmol, 1.0 equiv.), DABCO (157 mg, 1.4 mmol, 1.4 equiv.), trialkoxysilane **4m** (318 mg, 1.0 mmol, 1.0 equiv.) and trifluoroacetamide **1b** (591 mg, 2.3 mmol, 2.3 equiv.). The purification was accomplished by column chromatography on silica gel with Hexane/Ethyl acetate 1:1 as eluent to provide the desired amide **2n** (316 mg, 0.70 mmol, 70%).

White solid, mp 217 - 218 °C. **^{1}H NMR** (500 MHz, DMSO-d_6): δ = 10.72 (s, 2H), 8.59 (s, 1H), 8.26 (s, 2H), 8.18 (dd, ^3J=7.8 Hz, ^4J=1.6 Hz, 2H), 8.08 (d, ^3J=8.4 Hz, 2H), 7.70 (t, ^3J=7.8 Hz, 1H), 7.58 (t, J=8.0 Hz, 2H), 7.43 (d, ^3J=7.8 Hz, 2H).

^{13}C{^{1}H} NMR (126 MHz, DMSO-d_6): δ= 165.4, 139.9, 134.8, 131.1, 129.9, 129.5 (q, $^2J_{CF}$ = 31.6 Hz), 128.8, 127.3, 124.2 (q, $^1J_{CF}$ = 272.1 Hz), 123.8, 120.1, 116.5.

HRMS (TOF MS ES+) m/z: $[M + H]^+$: Calcd for $C_{22}H_{15}N_2O_2F_6$ (M+H) 453.1041. Found 453.1038.

N-benzyl-3-methylbenzamide (2o).

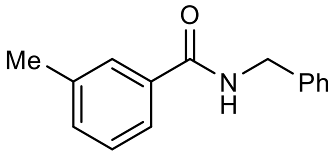

The title compound was prepared starting from $NiBr_2$ (22 mg, 0.1 mmol, 10 mol%), cucurbit[6]uril (**L**) (100 mg, 0.1 mmol, 10 mol%), Dy_2O_3 (373 mg, 1.0 mmol, 1.0 equiv.), DABCO (157 mg, 1.4 mmol, 1.4 equiv.), boronic acid **3b** (163 mg, 1.2 mmol, 1.2 equiv.) and trifluoroacetamide **1n** (203 mg, 1.0 mmol, 1.0 equiv.). The purification was accomplished by column chromatography on silica gel with Hexane/Ethyl acetate 5:1 as eluent to provide the desired amide **2o** (209 mg, 0.93 mmol, 93%).

Alternatively, the title compound was prepared starting from $NiBr_2$ (22 mg, 0.1 mmol, 10 mol%), cucurbit[6]uril (**L**) (100 mg, 0.1 mmol, 10 mol%), Dy_2O_3 (373 mg, 1.0 mmol, 1.0 equiv.), DABCO (157 mg, 1.4 mmol, 1.4 equiv.), trialkoxysilane **4b** (254 mg, 1.2 mmol, 1.2 equiv.) and trifluoroacetamide **1n** (203 mg, 1.0 mmol, 1.0 equiv.). The purification was accomplished by column chromatography on silica gel with Hexane/Ethyl acetate 5:1 as eluent to provide the desired amide **2o** (205 mg, 0.91 mmol, 91%).

White solid, mp 94 - 95 °C.**^{1}H NMR** (500 MHz, $CDCl_3$): δ = 7.62 (s, 1H), 7.60 – 7.54 (m, 1H), 7.36 – 7.32 (m, 4H), 7.31 – 7.27 (m, 3H), 6.65 (s, 1H), 4.61 (d, ^3J=5.7 Hz, 2H), 2.37 (s, 3H).

^{13}C{^{1}H} NMR (126 MHz, $CDCl_3$): δ= 167.5, 138.5, 138.4, 134.4, 132.3, 128.8, 128.5, 127.9, 127.8, 127.6, 124.0, 44.1, 21.4.

HRMS (TOF MS ES+) m/z: $[M + H]^+$: Calcd for $C_{15}H_{16}NO$ (M+H) 226.1234. Found 226.1232.

N-benzyl-4-fluoro-3-(trifluoromethyl)benzamide (2p).

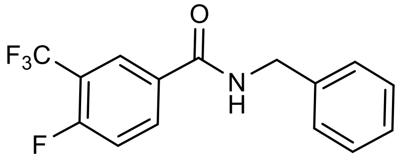

The title compound was prepared starting from $NiBr_2$ (22 mg, 0.1 mmol, 10 mol%), cucurbit[6]uril (**L**) (100 mg, 0.1 mmol, 10 mol%), Dy_2O_3 (373 mg, 1.0 mmol, 1.0 equiv.), DABCO (157 mg, 1.4 mmol, 1.4 equiv.), boronic acid **3r** (250 mg, 1.2 mmol, 1.2 equiv.) and trifluoroacetamide **1n** (203 mg, 1.0 mmol, 1.0 equiv.). The purification was accomplished by column chromatography on silica gel with Hexane/Ethyl acetate 4:1 as eluent to provide the desired amide **2p** (261 mg, 0.88 mmol, 88%).

White solid, mp 152 - 153 °C. **^{1}H NMR** (500 MHz, $CDCl_3$): δ = 8.06 (dd, ^3J=6.6 Hz, ^4J =1.9 Hz, 1H), 8.01 – 7.95 (m, 1H), 7.36 – 7.27 (m, 5H), 7.22 (t, ^3J=9.2 Hz, 1H), 6.92 – 6.58 (m, 1H), 4.59 (d, ^2J =5.6 Hz, 2H).

^{13}C{^{1}H} NMR (126 MHz, $CDCl_3$): δ= 165.1, 161.6 (d, $^1J_{CF}$ = 244.8 Hz), 137.6, 133.0 (d, J_{CF} = 8.5 Hz), 130.7 (d, J_{CF} = 3.6 Hz), 128.9, 127.9, 127.8, 126.5, 122.1 (q, $^1J_{CF}$ = 274.3 Hz, CF_3), 118.9 (q, $^2J_{CF}$ = 32.0 Hz), 117.3 (d, J_{CF} = 21.2 Hz), 44.4.

HRMS (TOF MS ES+) m/z: $[M + H]^+$: Calcd for $C_{15}H_{12}NOF_4$ (M+H) 298.0857. Found 298.0855.

***N*-phenethyl-3-(trifluoromethoxy)benzamide (2q).**

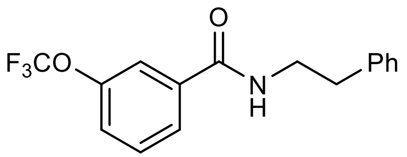

The title compound was prepared starting from $NiBr_2$ (22 mg, 0.1 mmol, 10 mol%), cucurbit[6]uril (**L**) (100 mg, 0.1 mmol, 10 mol%), Dy_2O_3 (373 mg, 1.0 mmol, 1.0 equiv.), DABCO (157 mg, 1.4 mmol, 1.4 equiv.), boronic acid **3p** (163 mg, 1.2 mmol, 1.2 equiv.) and trifluoroacetamide **1o** (217 mg, 1.0 mmol, 1.0 equiv.). The purification was accomplished by column chromatography on silica gel with Hexane/Ethyl acetate 4:1 as eluent to provide the desired amide **2q** (269 mg, 0.87 mmol, 87%).

Alternatively, the title compound was prepared starting from $NiBr_2$ (22 mg, 0.1 mmol, 10 mol%), cucurbit[6]uril (**L**) (100 mg, 0.1 mmol, 10 mol%), Dy_2O_3 (373 mg, 1.0 mmol, 1.0 equiv.), DABCO (157 mg, 1.4 mmol, 1.4 equiv.), trialkoxysilane **4j** (338 mg, 1.2 mmol, 1.2 equiv.) and trifluoroacetamide **1o** (217 mg, 1.0 mmol, 1.0 equiv.). The purification was accomplished by column chromatography on silica gel with Hexane/Ethyl acetate 4:1 as eluent to provide the desired amide **2q** (250 mg, 0.81 mmol, 81%).

White solid, mp 85 - 86 °C. **^{1}H NMR** (500 MHz, $CDCl_3$): δ = 7.59 – 7.57 (m, 2H), 7.42 (d, ^3J=8.2 Hz, 1H), 7.32 (d, ^3J=7.5 Hz, 3H), 7.24 (dd, ^3J=16.7 Hz, ^4J=6.5 Hz, 3H), 6.42 (s, 1H), 3.71 – 3.67 (m, 2H), 2.93 (t, ^3J=7.0 Hz, 2H).

^{13}C{^{1}H} NMR (126 MHz, $CDCl_3$): δ= 166.1, 149.4, 138.7, 136.8, 130.1, 128.8, 128.8, 126.7, 125.0, 123.8, 120.4 (q, $^1J_{CF}$ = 256.8 Hz), 119.8, 113.2, 41.4, 35.6.

HRMS (TOF MS ES+) m/z: $[M + H]^+$: Calcd for $C_{16}H_{15}NO_2F_3$ (M+H) 310.1065. Found 310.1055.

3,4,5-trifluoro-N-(3-phenylpropyl)benzamide (2r).

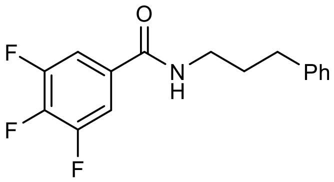

The title compound was prepared starting from $NiBr_2$ (22 mg, 0.1 mmol, 10 mol%), cucurbit[6]uril (**L**) (100 mg, 0.1 mmol, 10 mol%), Dy_2O_3 (373 mg, 1.0 mmol, 1.0 equiv.), DABCO (157 mg, 1.4 mmol, 1.4 equiv.), boronic acid **3j** (211 mg, 1.2 mmol, 1.2 equiv.) and trifluoroacetamide **1o** (231 mg, 1.0 mmol, 1.0 equiv.). The purification was accomplished by column chromatography on silica gel with Hexane/Ethyl acetate 4:1 as eluent to provide the desired amide **2r** (196 mg, 0.67 mmol, 67%).

White solid, mp 83 - 84 °C. **^{1}H NMR** (500 MHz, $CDCl_3$): δ = 7.35 – 7.15 (m, 7H), 6.32 (s, 1H), 3.47 (dd, ^3J=12.8 Hz, ^4J=6.7 Hz, 2H), 2.72 (t, ^3J=7.4 Hz, 2H), 1.96 (p, ^3J=7.1 Hz, 2H).

^{13}C{^{1}H} NMR (126 MHz, $CDCl_3$): δ= 164.3, 151.1 (d, $^1J_{CF}$ = 253.2 Hz), 150.0, 141.9 (d, $^1J_{CF}$ = 256.4 Hz), 130.4, 128.7, 128.4, 126.3, 111.6 (d, J_{CF} = 16.7 Hz), 40.4, 33.7, 30.8.

HRMS (TOF MS ES+) m/z: $[M + H]^+$: Calcd for $C_{16}H_{15}NOF_3$ (M+H) 294.1113. Found 294.1106.

4-bromo-N-(3-phenylpropyl)benzamide (2s).

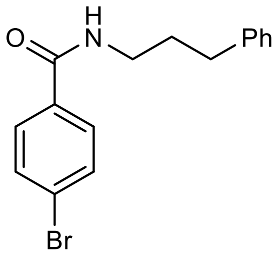

The title compound was prepared starting from $NiBr_2$ (22 mg, 0.1 mmol, 10 mol%), cucurbit[6]uril (**L**) (100 mg, 0.1 mmol, 10 mol%), Dy_2O_3 (373 mg, 1.0 mmol, 1.0 equiv.), DABCO (157 mg, 1.4 mmol, 1.4 equiv.), boronic acid **3d** (240 mg, 1.2 mmol, 1.2 equiv.) and trifluoroacetamide **1p** (231 mg,

1.0 mmol, 1.0 equiv.). The purification was accomplished by column chromatography on silica gel with Hexane/Ethyl acetate 3:1 as eluent to provide the desired amide **2s** (197 mg, 0.62 mmol, 62%).

Alternatively, the title compound was prepared starting from $NiBr_2$ (22 mg, 0.1 mmol, 10 mol%), cucurbit[6]uril (**L**) (100 mg, 0.1 mmol, 10 mol%), Dy_2O_3 (373 mg, 1.0 mmol, 1.0 equiv.), DABCO (157 mg, 1.4 mmol, 1.4 equiv.), trialkoxysilane **4d** (331 mg, 1.2 mmol, 1.2 equiv.) and trifluoroacetamide **1p** (231 mg, 1.0 mmol, 1.0 equiv.). The purification was accomplished by column chromatography on silica gel with Hexane/Ethyl acetate 3:1 as eluent to provide the desired amide **2s** (191 mg, 0.60 mmol, 60%).

White solid, mp 119 - 120 °C. **^{1}H NMR** (500 MHz, DMSO-d_6): δ= 8.58 (t, ^3J = 5.6 Hz, 1H), 7.78 (dt, ^3J = 8.5 Hz, ^4J = 1.7 Hz, 2H), 7.66 (dt, ^3J = 8.5 Hz, ^4J = 1.7 Hz, 2H), 7.29 – 7.26 (m, 2H), 7.21 (d, ^3J = 7.0 Hz, 2H), 7.17 (t, ^3J = 7.3 Hz, 1H), 3.27 (q, ^3J = 5.7 Hz, 2H), 2.62 (t, ^3J = 7.8 Hz, 2H), 1.82 (quint. ^3J = 7.4 Hz, 2H).

^{13}C{^{1}H} NMR (126 MHz, DMSO-d_6): δ= 165.2, 141.7, 133.7, 131.6, 129.3, 128.3, 128.2, 125.7, 124.7, 32.6, 30.8.

MS (GC, 70eV): m/z (%) = 319 (M^+, 37), 317 (36), 214 (100), 212 (99), 185 (78), 183 (80), 157 (37), 155 (32).

Anal. calcd. for $C_{16}H_{16}NOBr$: C, 60.39; H, 5.07; N, 4.40. Found: C, 60.49; H, 5.13, N, 4.26.

N-(4-methylbenzyl)-4-(trifluoromethoxy)benzamide (2t).

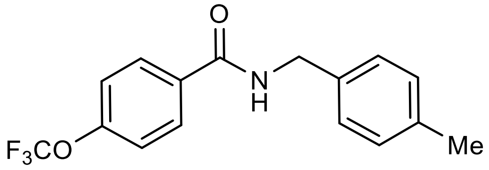

The title compound was prepared starting from $NiBr_2$ (22 mg, 0.1 mmol, 10 mol%), cucurbit[6]uril (**L**) (100 mg, 0.1 mmol, 10 mol%), Dy_2O_3 (373 mg, 1.0 mmol, 1.0 equiv.), DABCO (157 mg, 1.4 mmol, 1.4 equiv.), boronic acid **3q** (247 mg, 1.2 mmol, 1.2 equiv.) and trifluoroacetamide **1q** (217 mg, 1.0 mmol, 1.0 equiv.). The purification was accomplished by column chromatography on silica gel with Hexane/Ethyl acetate 3:1 as eluent to provide the desired amide **2t** (268 mg, 0.87 mmol, 87%).

Alternatively, the title compound was prepared starting from NiI_2 (31 mg, 0.1 mmol, 10 mol%), cucurbit[6]uril (**L**) (100 mg, 0.1 mmol, 10 mol%), Dy_2O_3 (373 mg, 1.0 mmol, 1.0 equiv.), DABCO (157 mg, 1.4 mmol, 1.4 equiv.), trialkoxysilane **4k** (338 mg, 1.2 mmol, 1.2 equiv.) and trifluoroacetamide **1q** (217 mg, 1.0 mmol, 1.0 equiv.). The purification was accomplished by column chromatography on silica gel with Hexane/Ethyl acetate 3:1 as eluent to provide the desired amide **2t** (265 mg, 0.86 mmol, 86%).

Alternatively, the title compound was prepared starting from $NiBr_2$ (22 mg, 0.1 mmol, 10 mol%), cucurbit[6]uril (**L**) (100 mg, 0.1 mmol, 10 mol%), Dy_2O_3 (373 mg, 1.0 mmol, 1.0 equiv.), DABCO (157 mg, 1.4 mmol, 1.4 equiv.), bis(pinacolato)diborane (330 mg, 1.3 mmol, 1.3 equiv.), iodonium salt **5f** (437 mg, 0.7 mmol, 0.7 equiv.) and trifluoroacetamide **1q** (217 mg, 1.0 mmol, 1.0 equiv.). The purification was accomplished by column chromatography on silica gel with Hexane/Ethyl acetate 3:1 as eluent to provide the desired amide **2t** (256 mg, 0.83 mmol, 83%).

Alternatively, the title compound was prepared starting from $NiBr_2$ (22 mg, 0.1 mmol, 10 mol%), cucurbit[6]uril (**L**) (100 mg, 0.1 mmol, 10 mol%), Dy_2O_3 (373 mg, 1.0 mmol, 1.0 equiv.), DABCO (157 mg, 1.4 mmol, 1.4 equiv.), bis(pinacolato)diborane (305 mg, 1.2 mmol, 1.2 equiv.); then appropriate sulphonium salt **6c** (446 mg, 1.2 mmol, 1.2 equiv.) and trifluoroacetamide **1q** (217 mg, 1.0 mmol, 1.0 equiv.). The purification was accomplished by column chromatography on silica gel with Hexane/Ethyl acetate 3:1 as eluent to provide the desired amide **2t** (265 mg, 0.86 mmol, 86%).

Alternatively, the title compound was prepared starting from $NiBr_2$ (22 mg, 0.1 mmol, 10 mol%), cucurbit[6]uril (**L**) (100 mg, 0.1 mmol, 10 mol%), Dy_2O_3 (373 mg, 1.0 mmol, 1.0 equiv.), DABCO (157 mg, 1.4 mmol, 1.4 equiv.), potassium trifluoro(4-(trifluoromethoxy)phenyl)borate (322 mg, 1.2 mmol, 1.2 equiv.) and trifluoroacetamide **1q** (217 mg, 1.0 mmol, 1.0 equiv.). The purification was accomplished by column chromatography on silica gel with Hexane/Ethyl acetate 3:1 as eluent to provide the desired amide **2t** (250 mg, 0.81 mmol, 81%).

Alternatively, the title compound was prepared starting from $NiBr_2$ (22 mg, 0.1 mmol, 10 mol%), cucurbit[6]uril (**L**) (100 mg, 0.1 mmol, 10 mol%), Dy_2O_3 (373 mg, 1.0 mmol, 1.0 equiv.), DABCO (157 mg, 1.4 mmol, 1.4 equiv.), 4,4,5,5-tetramethyl-2-(4-(trifluoromethoxy)phenyl)-1,3,2-dioxaborolane (346 mg, 1.2 mmol, 1.2 equiv.) and trifluoroacetamide **1q** (217 mg, 1.0 mmol, 1.0 equiv.). The purification was accomplished by column chromatography on silica gel with Hexane/Ethyl acetate 3:1 as eluent to provide the desired amide **2t** (256 mg, 0.83 mmol, 83%).

White solid, mp 158 - 159 ^{o}C. **^{1}H NMR** (500 MHz, $CDCl_3$): δ = 7.81 (d, ^3J=8.7 Hz, 2H), 7.22 (d, ^3J=8.0 Hz, 4H), 7.15 (d, ^3J=7.9 Hz, 2H), 6.57 (s, 1H), 4.57 (d, ^2J=5.5 Hz, 2H), 2.34 (s, 3H).

$^{13}C\{^1H\}$ NMR (126 MHz, $CDCl_3$): δ= 166.1, 151.5, 137.5, 134.9, 132.9, 129.5, 128.9, 128.0, 121.4, 120.3 (q, $^1J_{CF}$ = 261.7 Hz), 44.0, 21.1.

HRMS (TOF MS ES+) m/z: $[M + H]^+$: Calcd for $C_{16}H_{15}NO_2F_3$ (M+H) 310.1064. Found 310.1055.

N-(4-methylbenzyl)-1-naphthamide (2u).

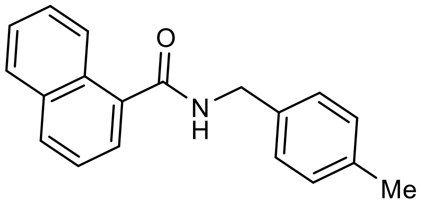

The title compound was prepared starting from $NiBr_2$ (22 mg, 0.1 mmol, 10 mol%), cucurbit[6]uril (**L**) (100 mg, 0.1 mmol, 10 mol%), Dy_2O_3 (373 mg, 1.0 mmol, 1.0 equiv.), DABCO (157 mg, 1.4 mmol, 1.4 equiv.), boronic acid **3y** (206 mg, 1.2 mmol, 1.2 equiv.) and trifluoroacetamide **1q** (217 mg, 1.0 mmol, 1.0 equiv.). The purification was accomplished by column chromatography on silica gel with Hexane/Ethyl acetate 2:1 as eluent to provide the desired amide **2u** (225 mg, 0.82 mmol, 82%).

Alternatively, the title compound was prepared starting from $NiBr_2$ (22 mg, 0.1 mmol, 10 mol%), cucurbit[6]uril (**L**) (100 mg, 0.1 mmol, 10 mol%), Dy_2O_3 (373 mg, 1.0 mmol, 1.0 equiv.), DABCO (157 mg, 1.4 mmol, 1.4 equiv.), trialkoxysilane **4n** (298 mg, 1.2 mmol, 1.2 equiv.) and trifluoroacetamide **1q** (217 mg, 1.0 mmol, 1.0 equiv.). The purification was accomplished by column chromatography on silica gel with Hexane/Ethyl acetate 2:1 as eluent to provide the desired amide **2u** (251 mg, 0.91 mmol, 91%).

Alternatively, the title compound was prepared starting from NiI_2 (31 mg, 0.1 mmol, 10 mol%), cucurbit[6]uril (**L**) (100 mg, 0.1 mmol, 10 mol%), Dy_2O_3 (373 mg, 1.0 mmol, 1.0 equiv.), DABCO (157 mg, 1.4 mmol, 1.4 equiv.), bis(pinacolato)diborane (330 mg, 1.3 mmol, 1.3 equiv.), iodonium salt **5g** (389 mg, 0.7 mmol, 0.7 equiv.) and trifluoroacetamide **1q** (217 mg, 1.0 mmol, 1.0 equiv.). The purification was accomplished by column chromatography on silica gel with Hexane/Ethyl acetate 2:1 as eluent to provide the desired amide **2u** (236 mg, 0.86 mmol, 86%).

White solid, mp 144 - 145 ^oC. **^1H NMR** (500 MHz, $CDCl_3$): δ= 8.33 (d, ^3J = 7.8 Hz, 1H), 7.96 – 7.81 (m, 2H), 7.59 – 7.49 (m, 3H), 7.43 – 7.37 (m, 1H), 7.27 (t, J = 5.3 Hz, 2H), 7.17 (d, J = 7.8 Hz, 2H), 6.37 (s, 1H), 4.65 (d, ^3J = 5.6 Hz, 2H), 2.36 (s, 3H).

$^{13}C\{^1H\}$ NMR (126 MHz, $CDCl_3$): δ= 169.3, 137.3, 135.0, 134.3, 133.6, 130.6, 130.1, 129.4, 128.2, 127.8, 127.1, 126.4, 125.4, 124.9, 124.6, 43.8, 21.1.

HRMS (TOF MS ES+) m/z: $[M + H]^+$: Calcd for $C_{16}H_{15}NO_2F_3$ (M+H) 310.1064. Found 310.1055.

4-cyano-N-(4-fluorobenzyl)benzamide (2v).

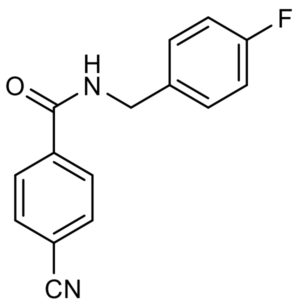

The title compound was prepared starting from $NiBr_2$ (22 mg, 0.1 mmol, 10 mol%), cucurbit[6]uril (**L**) (100 mg, 0.1 mmol, 10 mol%), Dy_2O_3 (373 mg, 1.0 mmol, 1.0 equiv.), DABCO (157 mg, 1.4 mmol, 1.4 equiv.), boronic acid **3u** (176 mg, 1.2 mmol, 1.2 equiv.) and trifluoroacetamide **1x** (221 mg, 1.0 mmol, 1.0 equiv.). The purification was accomplished by column chromatography on silica gel with Hexane/Ethyl acetate 3:1 as eluent to provide the desired amide **2v** (165 mg, 0.65 mmol, 65%).

White solid, mp 119 - 120 °C. **^1H NMR** (500 MHz, $CDCl_3$): δ= 7.87 (d, ^3J = 8.5 Hz, 2H), 7.67 (d, ^3J = 8.5 Hz, 2H), 7.29 – 7.26 (m, 2H), 7.05 (br. s, 2H), 7.00 (t, ^3J = 8.5 Hz, 2H), 4.55 (d, ^3J = 5.8 Hz, 2H).

$^{13}C\{^1H\}$ NMR (126 MHz, $CDCl_3$): δ= 165.6, 162.2 (d, $^1J_{CF}$ = 243.2 Hz), 138.0, 133.4, 132.3, 129.5 (d, J_{CF} = 8.3 Hz), 127.7, 117.9, 115.5 (d, J_{CF} = 21.0 Hz), 114.9, 43.4.

Anal. calcd. for $C_{15}H_{11}N_2OF$: C, 70.86; H, 4.36; N, 11.02. Found: C, 70.93; H, 4.39, N, 11.11.

N-(benzo[d][1,3] dioxol-5-ylmethyl)-3-(trifluoromethyl)benzamide (2w).

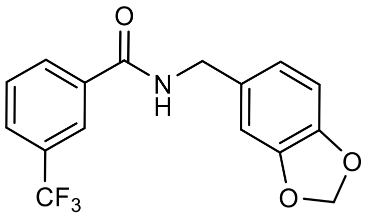

The title compound was prepared starting from $NiBr_2$ (22 mg, 0.1 mmol, 10 mol%), cucurbit[6]uril (**L**) (100 mg, 0.1 mmol, 10 mol%), Dy_2O_3 (373 mg, 1.0 mmol, 1.0 equiv.), DABCO (157 mg, 1.4 mmol, 1.4 equiv.), boronic acid **3l** (228 mg, 1.2 mmol, 1.2 equiv.) and trifluoroacetamide **1y** (247 mg, 1.0 mmol, 1.0 equiv.). The purification was accomplished by column chromatography on silica gel with Hexane/Ethyl acetate 3:1 as eluent to provide the desired amide **2w** (265 mg, 0.82 mmol, 82%). The gram scale synthesis was performed on 10 mmol of the starting **1y** and the aromatic Amide **2w** was prepared in 80% yield (2.58 g, 8.0 mmol).

Alternatively, the title compound was prepared starting from $NiBr_2$ (22 mg, 0.1 mmol, 10 mol%), cucurbit[6]uril (**L**) (100 mg, 0.1 mmol, 10 mol%), Dy_2O_3 (373 mg, 1.0 mmol, 1.0 equiv.), DABCO (157 mg, 1.4 mmol, 1.4 equiv.), trialkoxysilane **4g** (319 mg, 1.2 mmol, 1.2 equiv.) and trifluoroacetamide **1y** (247 mg, 1.0 mmol, 1.0 equiv.). The purification was accomplished by column chromatography on silica gel with Hexane/Ethyl acetate 3:1 as eluent to provide the desired amide **2w** (258 mg, 0.80 mmol, 80%). The gram scale synthesis was performed on 10 mmol of the starting **1y** and the aromatic Amide **2w** was prepared in 71% yield (2.29 g, 7.1 mmol).

Alternatively, the title compound was prepared starting from NiI_2 (31 mg, 0.1 mmol, 10 mol%), cucurbit[6]uril (**L**) (100 mg, 0.1 mmol, 10 mol%), Dy_2O_3 (373 mg, 1.0 mmol, 1.0 equiv.), DABCO (157 mg, 1.4 mmol, 1.4 equiv.), bis(pinacolato)diborane (330 mg, 1.3 mmol, 1.3 equiv.), iodonium salt **5d** (414 mg, 0.7 mmol, 0.7 equiv) and trifluoroacetamide **1y** (247 mg, 1.0 mmol, 1.0 equiv.). The purification was accomplished by column chromatography on silica gel with Hexane/Ethyl acetate 3:1 as eluent to provide the desired amide **2w** (262 mg, 0.81 mmol, 81%). The gram scale synthesis was performed on 10 mmol of the starting **1y** and the aromatic Amide **2w** was prepared in 74% yield (2.39 g, 7.4 mmol).

Alternatively, the title compound was prepared starting from NiI_2 (31 mg, 0.1 mmol, 10 mol%), cucurbit[6]uril (**L**) (100 mg, 0.1 mmol, 10 mol%), Dy_2O_3 (373 mg, 1.0 mmol, 1.0 equiv.), DABCO (157 mg, 1.4 mmol, 1.4 equiv.), bis(pinacolato)diborane (305 mg, 1.2 mmol, 1.2 equiv.); then appropriate sulphonium salt **6b** (427 mg, 1.2 mmol, 1.2 equiv.) and trifluoroacetamide **1y** (247 mg, 1.0 mmol, 1.0 equiv.). The purification was

accomplished by column chromatography on silica gel with Hexane/Ethyl acetate 3:1 as eluent to provide the desired amide **2w** (236 mg, 0.73 mmol, 73%). The gram scale synthesis was performed on 10 mmol of the starting **1y** and the aromatic Amide **2w** was prepared in 65% yield (2.10 g, 6.5 mmol).

Alternatively, the title compound was prepared starting from $NiBr_2$ (22 mg, 0.1 mmol, 10 mol%), cucurbit[6]uril (**L**) (100 mg, 0.1 mmol, 10 mol%), Dy_2O_3 (373 mg, 1.0 mmol, 1.0 equiv.), DABCO (157 mg, 1.4 mmol, 1.4 equiv.), potassium trifluoro(3-(trifluoromethyl)phenyl)borate (302 mg, 1.2 mmol, 1.2 equiv.) and trifluoroacetamide **1y** (247 mg, 1.0 mmol, 1.0 equiv.). The purification was accomplished by column chromatography on silica gel with Hexane/Ethyl acetate 3:1 as eluent to provide the desired amide **2w** (258 mg, 0.80 mmol, 80%). The gram scale synthesis was performed on 10 mmol of the starting **1y** and the aromatic Amide **2w** was prepared in 71% yield (2.29 g, 7.1 mmol).

Alternatively, the title compound was prepared starting from $NiBr_2$ (22 mg, 0.1 mmol, 10 mol%), cucurbit[6]uril (**L**) (100 mg, 0.1 mmol, 10 mol%), Dy_2O_3 (373 mg, 1.0 mmol, 1.0 equiv.), DABCO (157 mg, 1.4 mmol, 1.4 equiv.), 4,4,5,5-tetramethyl-2-(3-(trifluoromethyl)phenyl)-1,3,2-dioxaborolane (326 mg, 1.2 mmol, 1.2 equiv.) and trifluoroacetamide **1y** (247 mg, 1.0 mmol, 1.0 equiv.). The purification was accomplished by column chromatography on silica gel with Hexane/Ethyl acetate 3:1 as eluent to provide the desired amide **2w** (239 mg, 0.74 mmol, 74%). The gram scale synthesis was performed on 10 mmol of the starting **1y** and the aromatic Amide **2w** was prepared in 61% yield (1.97 g, 6.1 mmol).

White solid, mp 102 - 103 °C. **^{1}H NMR** (500 MHz, $CDCl_3$): δ = 8.03 (s, 1H), 7.93 (d, ^3J=7.8 Hz, 1H), 7.70 (d, ^3J=7.7 Hz, 1H), 7.48 (t, ^3J=7.7 Hz, 1H), 7.15 (s, 1H), 6.77 (s, 1H), 6.75 – 6.68 (m, 2H), 5.89 (d, ^2J=0.8 Hz, 2H), 4.50 – 4.42 (m, 2H).

^{13}C{^{1}H} NMR (126 MHz, $CDCl_3$): δ= 166.0, 147.9, 147.0, 135.0, 131.6, 130.9 (q, $^2J_{CF}$ = 34.1 Hz), 130.3, 129.1, 128.0 (m), 124.0, 123.6 (q, $^1J_{CF}$ = 272.8 Hz, CF_3), 121.1, 108.3, 108.2, 101.0, 43.9.

MS (GC, 70eV): m/z (%) = 323 (M^+, 83), 173 (100), 145 (50), 135 (42).

Anal. calcd. for $C_{16}H_{12}NO_3F_3$: C, 59.45; H, 3.74; N, 4.33. Found: C, 59.62; H, 3.69, N, 4.30.

N-(1-phenylethyl)-4-(trifluoromethyl)benzamide (2x).

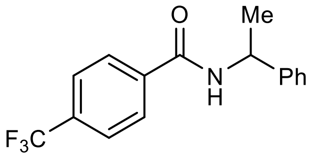

The title compound was prepared starting from $NiBr_2$ (22 mg, 0.1 mmol, 10 mol%), cucurbit[6]uril (**L**) (100 mg, 0.1 mmol, 10 mol%), Dy_2O_3 (373 mg, 1.0 mmol, 1.0 equiv.), DABCO (157 mg, 1.4 mmol, 1.4 equiv.), boronic acid **3m** (228 mg, 1.2 mmol, 1.2 equiv.) and trifluoroacetamide **1v** (217 mg, 1.0 mmol, 1.0 equiv.). The purification was accomplished by column chromatography on silica gel with Hexane/Ethyl acetate 4:1 as eluent to provide the desired amide **2x** (264 mg, 0.90 mmol, 90%).

Alternatively, the title compound was prepared starting from $NiBr_2$ (22 mg, 0.1 mmol, 10 mol%), cucurbit[6]uril (**L**) (100 mg, 0.1 mmol, 10 mol%), Dy_2O_3 (373 mg, 1.0 mmol, 1.0 equiv.), DABCO (157 mg, 1.4 mmol, 1.4 equiv.), trialkoxysilane **4h** (319 mg, 1.2 mmol, 1.2 equiv.) and trifluoroacetamide **1v** (217 mg, 1.0 mmol, 1.0 equiv.). The purification was accomplished by column chromatography on silica gel with Hexane/Ethyl acetate 4:1 as eluent to provide the desired amide **2x** (270 mg, 0.92 mmol, 92%).

White solid, mp 133 - 135 °C. **^{1}H NMR** (500 MHz, $CDCl_3$): δ = 7.83 (d, ^3J=8.1 Hz, 2H), 7.61 (d, ^3J=8.1 Hz, 2H), 7.39 – 7.32 (m, 4H), 7.31 – 7.25 (m, 1H), 6.76 (s, 1H), 5.30 (p, ^3J=7.0 Hz, 1H), 1.59 (d, ^3J=6.9 Hz, 3H).

^{13}C{^{1}H} NMR (126 MHz, $CDCl_3$): δ= 165.4, 142.8, 137.8, 133.1 (q, J_{CF} = 32.8 Hz), 128.7, 127.6, 127.4, 126.2, 125.5 (m), 123.6 (q, $^1J_{CF}$ = 272.5 Hz), 49.5, 21.6.

HRMS (TOF MS ES+) m/z: $[M + H]^+$: Calcd for $C_{16}H_{15}NOF_3$ (M+H) 294.1110. Found 294.1106.

2-fluoro-N-phenethylbenzamide (2y).

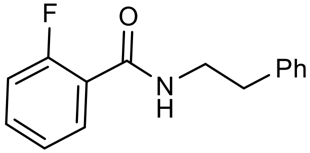

The title compound was prepared starting from $NiBr_2$ (22 mg, 0.1 mmol, 10 mol%), cucurbit[6]uril (**L**) (100 mg, 0.1 mmol, 10 mol%), Dy_2O_3 (373 mg, 1.0 mmol, 1.0 equiv.), DABCO (157 mg, 1.4 mmol, 1.4 equiv.), boronic acid **3f** (168 mg, 1.2 mmol, 1.2 equiv.) and trifluoroacetamide **1o** (217 mg, 1.0 mmol, 1.0 equiv.). The purification was accomplished by column chromatography on silica gel with Hexane/Ethyl acetate 4:1 as eluent to provide the desired amide **2y** (201 mg, 0.83 mmol, 83%).

Alternatively, the title compound was prepared starting from NiI_2 (31 mg, 0.1 mmol, 10 mol%), cucurbit[6]uril (**L**) (100 mg, 0.1 mmol, 10 mol%), Dy_2O_3 (373 mg, 1.0 mmol, 1.0 equiv.), DABCO (157 mg, 1.4 mmol, 1.4 equiv.), bis(pinacolato)diborane (330 mg, 1.3 mmol, 1.3 equiv.), iodonium salt **5d** (344 mg, 0.7 mmol, 0.7 equiv.) and trifluoroacetamide **1o** (217 mg, 1.0 mmol, 1.0 equiv.). The purification was accomplished by column chromatography on silica gel with Hexane/Ethyl acetate 4:1 as eluent to provide the desired amide **2y** (197 mg, 0.81 mmol, 81%).

White solid, mp 63 - 64 ^oC. **^{1}H NMR** (500 MHz, $CDCl_3$): δ = 8.08 (td, ^3J=7.9 Hz, ^4J=1.8 Hz, 1H), 7.49 – 7.40 (m, 1H), 7.36 – 7.29 (m, 2H), 7.24 (ddd, ^3J=5.2 Hz, ^4J=4.4 Hz, ^5J=1.4 Hz, 4H), 7.07 (ddd, ^3J=12.1 Hz, ^4J=8.3 Hz, ^5J=0.8 Hz, 1H), 6.80 (s, 1H), 3.76 – 3.71 (m, 2H), 2.94 (t, ^2J=7.0 Hz, 2H).

^{13}C{^{1}H} NMR (126 MHz, $CDCl_3$): δ= 163.1, 160.5 (q, $^1J_{CF}$ = 242.3 Hz), 138.8, 133.1 (d, J_{CF} = 8.5 Hz,), 131.9, 128.6 (d, J_{CF} = 15.4 Hz), 126.5, 126.6, 124.6 (m), 121.0(d, J_{CF} = 11.8 Hz), 115.8 (d, J_{CF} = 23.8 Hz), 41.2, 35.6.

HRMS (TOF MS ES+) m/z: $[M + H]^+$: Calcd for $C_{15}H_{15}NOF$ (M+H) 244.1144. Found 244.1138.

N-propyl-2-(trifluoromethyl)benzamide (2z).

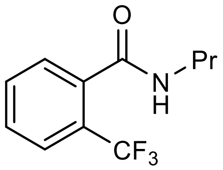

The title compound was prepared starting from $NiBr_2$ (22 mg, 0.1 mmol, 10 mol%), cucurbit[6]uril (**L**) (100 mg, 0.1 mmol, 10 mol%), Dy_2O_3 (373 mg, 1.0 mmol, 1.0 equiv.), DABCO (157 mg, 1.4 mmol, 1.4 equiv.), boronic acid **3k** (228 mg, 1.2 mmol, 1.2 equiv.) and trifluoroacetamide **1r** (155 mg, 1.0 mmol, 1.0 equiv.). The purification was accomplished by column chromatography on silica gel with Hexane/Ethyl acetate 4:1 as eluent to provide the desired amide **2z** (166 mg, 0.72 mmol, 72%).

Alternatively, the title compound was prepared starting from $NiBr_2$ (22 mg, 0.1 mmol, 10 mol%), cucurbit[6]uril (**L**) (100 mg, 0.1 mmol, 10 mol%), Dy_2O_3 (373 mg, 1.0 mmol, 1.0 equiv.), DABCO (157 mg, 1.4 mmol, 1.4 equiv.), trialkoxysilane **4f** (319 mg, 1.2 mmol, 1.2 equiv.) and trifluoroacetamide **1r** (155 mg, 1.0 mmol, 1.0 equiv.). The purification was accomplished by column chromatography on silica gel with Hexane/Ethyl acetate 4:1 as eluent to provide the desired amide **2z** (164 mg, 0.72 mmol, 71%).

White solid, mp 58 - 60 °C. **^{1}H NMR** (500 MHz, $CDCl_3$): δ = 7.62 (d, ^3J=7.5 Hz, 1H), 7.54 – 7.44 (m, 2H), 7.43 (s, 1H), 6.12 (s, 1H), 3.36 – 3.24 (m, 2H), 1.63 – 1.49 (m, 2H), 0.92 (td, ^3J=7.4 Hz, ^2J=1.1 Hz, 3H).

^{13}C{^{1}H} NMR (126 MHz, $CDCl_3$): δ= 167.8, 136.1, 131.8, 129.5, 128.5, 127.0 (q, $^2J_{CF}$ = 31.9 Hz), 126.1 (m), 123.5 (q, $^1J_{CF}$ = 273.3 Hz), 41.8, 22.4, 11.2.

HRMS (TOF MS ES+) m/z: $[M + H]^+$: Calcd for $C_{11}H_{13}NOF_3$ (M+H) 232.0952. Found 232.0949.

***N*-isopropyl-4-((trifluoromethyl)thio)benzamide (2aa).**

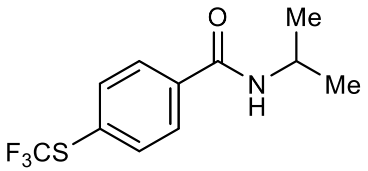

The title compound was prepared starting from $NiBr_2$ (22 mg, 0.1 mmol, 10 mol%), cucurbit[6]uril (**L**) (100 mg, 0.1 mmol, 10 mol%), Dy_2O_3 (373 mg, 1.0 mmol, 1.0 equiv.), DABCO (157 mg, 1.4 mmol, 1.4 equiv.), boronic acid **3t** (266 mg, 1.2 mmol, 1.2 equiv.) and trifluoroacetamide **1s** (155 mg, 1.0 mmol, 1.0 equiv.). The purification was accomplished by column chromatography on silica gel with Hexane/Ethyl acetate 6:1 as eluent to provide the desired amide **2aa** (234 mg, 0.89 mmol, 89%).

White solid, mp 88 - 89 °C. **^{1}H NMR** (500 MHz, $CDCl_3$): δ = 7.77 (d, ^3J=8.2 Hz, 2H), 7.65 (d, ^3J=7.3 Hz, 2H), 6.29 (s, 1H), 4.24 (dq, ^3J=13.1 Hz, ^4J=6.6 Hz, 1H), 1.23 (d, ^3J=6.6 Hz, 6H).

^{13}C{^{1}H} NMR (126 MHz, $CDCl_3$): δ= 165.5, 137.2, 136.0, 129.3 (q, $^1J_{CF}$ = 306.0 Hz), 127.9, 127.7, 42.1, 22.6.

HRMS (TOF MS ES+) m/z: $[M + H]^+$: Calcd for $C_{11}H_{13}NOF_3S$ (M+H) 264.0674. Found 264.0670.

2,4,5-trifluoro-N,N-diisopropylbenzamide (2ab).

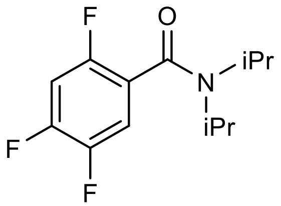

The title compound was prepared starting from $NiBr_2$ (22 mg, 0.1 mmol, 10 mol%), cucurbit[6]uril (**L**) (100 mg, 0.1 mmol, 10 mol%), Dy_2O_3 (373 mg, 1.0 mmol, 1.0 equiv.), DABCO (157 mg, 1.4 mmol, 1.4 equiv.), boronic acid **3i** (211 mg, 1.2 mmol, 1.2 equiv.) and trifluoroacetamide **1t** (197 mg, 1.0 mmol, 1.0 equiv.). The purification was accomplished by column chromatography on silica gel with Hexane/Ethyl acetate 8:1 as eluent to provide the desired amide **2ab** (199 mg, 0.77 mmol, 77%).

White solid, mp 62 - 63 ^oC. **^{1}H NMR** (500 MHz, $CDCl_3$): δ = 7.08 (td, ^3J=9.0 Hz, ^4J=6.0 Hz, 1H), 6.93 (ddd, ^3J=9.8 Hz, ^4J=8.5 Hz, ^5J=6.3 Hz, 1H), 3.66 (dd, ^3J=13.2 Hz, ^4J=6.6 Hz, 1H), 3.50 (dt, ^3J=13.6 Hz, ^4J=6.8 Hz, 1H), 1.49 (d, ^3J=6.9 Hz, 6H), 1.13 (d, ^3J=36.8 Hz, 6H).

^{13}C{^{1}H} NMR (126 MHz, $CDCl_3$): δ= 163.4, 154.1 (ddd, $^1J_{CF}$ = 245.4 Hz, J_{CF} = 8.9, J_{CF} = 2.0 Hz), 150.0 (dt, $^1J_{CF}$ = 253.1 Hz, J_{CF} = 14.3 Hz), 147.0 (ddd, $^1J_{CF}$ = 246.9 Hz, J_{CF} = 12.9 Hz, J_{CF} = 3.7 Hz), 122.9 (dt, J_{CF} = 22.2 Hz, J_{CF} = 4.5 Hz), 115.9 (dd, J_{CF} = 20.3 Hz, J_{CF} = 5.7 Hz), 105.9 (m), 51.3, 46.2, 20.6 (m).

HRMS (TOF MS ES+) m/z: $[M + H]^+$: Calcd for $C_{13}H_{17}NOF_3$ (M+H) 260.1264. Found 260.1262.

N-cyclohexyl-2,5-difluorobenzamide (2ac).

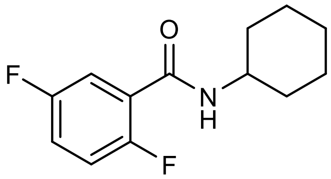

The title compound was prepared starting from $NiBr_2$ (22 mg, 0.1 mmol, 10 mol%), cucurbit[6]uril (**L**) (100 mg, 0.1 mmol, 10 mol%), Dy_2O_3 (373 mg, 1.0 mmol, 1.0 equiv.), DABCO (157 mg, 1.4 mmol, 1.4 equiv.), boronic acid **3h** (190 mg, 1.2 mmol, 1.2 equiv.) and trifluoroacetamide **1w** (195 mg,

1.0 mmol, 1.0 equiv.). The purification was accomplished by column chromatography on silica gel with Hexane/Ethyl acetate 4:1 as eluent to provide the desired amide **2ac** (189 mg, 0.79 mmol, 79 %).

White solid, mp 110 - 112 °C. **^{1}H NMR** (500 MHz, $CDCl_3$): δ = 7.74 (ddd, ^3J=8.9 Hz, ^4J=6.0 Hz, ^5J=3.2 Hz, 1H), 7.12 – 7.02 (m, 2H), 6.62 (s, 1H), 3.98 (dd, ^3J=7.2 Hz, ^4J=3.2 Hz, 1H), 2.03 – 1.96 (m, 2H), 1.75 – 1.69 (m, 2H), 1.65 – 1.58 (m), 1.41 (ddd, ^3J=25.0 Hz, ^4J=9.3 Hz, ^5J=3.3 Hz, 2H), 1.29 – 1.18 (m, 3H).

^{13}C{^{1}H} NMR (126 MHz, $CDCl_3$): δ= 160.9, 158.8 (d, $^1J_{CF}$ = 244.8 Hz), 156.6 (d, $^1J_{CF}$ = 242.1 Hz), 123.0 (m), 119.5 (dd, J_{CF} = 24.7 Hz, J_{CF} = 10.6 Hz), 118.1 (d, J_{CF} = 25.8 Hz), 117.3 (dd, J_{CF} = 28.2 Hz, J_{CF} = 8.2 Hz), 48.8, 32.9, 25.5, 24.7.

HRMS (TOF MS ES+) m/z: $[M + H]^+$: Calcd for $C_{13}H_{16}NOF_2$ (M+H) 240.1203. Found 240.1200.

N-(tert-butyl)-3,5-difluorobenzamide (2ad).

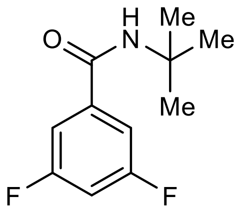

The title compound was prepared starting from $NiBr_2$ (22 mg, 0.1 mmol, 10 mol%), cucurbit[6]uril (**L**) (100 mg, 0.1 mmol, 10 mol%), Dy_2O_3 (373 mg, 1.0 mmol, 1.0 equiv.), DABCO (157 mg, 1.4 mmol, 1.4 equiv.), boronic acid **3g** (190 mg, 1.2 mmol, 1.2 equiv.) and trifluoroacetamide **1u** (169 mg, 1.0 mmol, 1.0 equiv.). The purification was accomplished by column chromatography on silica gel with Hexane/Ethyl acetate 5:1 as eluent to provide the desired amide **2ad** (179 mg, 0.84 mmol, 84%).

White solid, mp 115 - 116 °C. **^{1}H NMR** (500 MHz, $CDCl_3$): δ = 7.20 (dd, ^3J=7.8 Hz, ^4J=2.2 Hz, 2H), 6.88 (tt, ^3J=8.6 Hz, ^4J=2.3 Hz, 1H), 6.00 (s, 1H), 1.44 (s, 9H).

^{13}C{^{1}H} NMR (126 MHz, $CDCl_3$): δ= 164.4, 162.8 (dd, $^1J_{CF}$ = 250.5 Hz, J_{CF} = 12.5 Hz), 139.3, 110.0 (dd, $^1J_{CF}$ = 19.7 Hz, J_{CF} = 6.6 Hz), 106.3 (t, J_{CF} = 25.8 Hz), 52.0, 28.7.

HRMS (TOF MS ES+) m/z: $[M + H]^+$: Calcd for $C_{11}H_{14}NOF_2$ (M+H) 214.1043. Found 214.1043.

***N*-(adamantan-1-yl)-4-(trifluoromethyl)benzamide (2ae).**

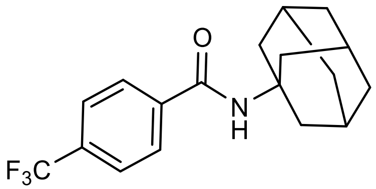

The title compound was prepared starting from $NiBr_2$ (22 mg, 0.1 mmol, 10 mol%), cucurbit[6]uril (**L**) (100 mg, 0.1 mmol, 10 mol%), Dy_2O_3 (373 mg, 1.0 mmol, 1.0 equiv.), DABCO (157 mg, 1.4 mmol, 1.4 equiv.), boronic acid **3m** (228 mg, 1.2 mmol, 1.2 equiv.) and trifluoroacetamide **1z** (247 mg, 1.0 mmol, 1.0 equiv.). The purification was accomplished by column chromatography on silica gel with Hexane/Ethyl acetate 5:1 as eluent to provide the desired amide **2ae** (268 mg, 0.83 mmol, 83%).

Alternatively, the title compound was prepared starting from $NiBr_2$ (22 mg, 0.1 mmol, 10 mol%), cucurbit[6]uril (**L**) (100 mg, 0.1 mmol, 10 mol%), Dy_2O_3 (373 mg, 1.0 mmol, 1.0 equiv.), DABCO (157 mg, 1.4 mmol, 1.4 equiv.), trialkoxysilane **4h** (319 mg, 1.2 mmol, 1.2 equiv.) and trifluoroacetamide **1z** (247 mg, 1.0 mmol, 1.0 equiv.). The purification was accomplished by column chromatography on silica gel with Hexane/Ethyl acetate 5:1 as eluent to provide the desired amide **2ae** (284 mg, 0.88 mmol, 88%).

Alternatively, the title compound was prepared starting from $NiBr_2$ (22 mg, 0.1 mmol, 10 mol%), cucurbit[6]uril (**L**) (100 mg, 0.1 mmol, 10 mol%), Dy_2O_3 (373 mg, 1.0 mmol, 1.0 equiv.), DABCO (157 mg, 1.4 mmol, 1.4 equiv.), potassium trifluoro(4-(trifluoromethyl)phenyl)borate (302 mg, 1.2 mmol, 1.2 equiv.) and trifluoroacetamide **1z** (247 mg, 1.0 mmol, 1.0 equiv.). The purification was accomplished by column chromatography on silica gel with Hexane/Ethyl acetate 5:1 as eluent to provide the desired amide **2ae** (239 mg, 0.74 mmol, 74%).

Alternatively, the title compound was prepared starting from $NiBr_2$ (22 mg, 0.1 mmol, 10 mol%), cucurbit[6]uril (**L**) (100 mg, 0.1 mmol, 10 mol%), Dy_2O_3 (373 mg, 1.0 mmol, 1.0 equiv.), DABCO (157 mg, 1.4 mmol, 1.4 equiv.), 4,4,5,5-tetramethyl-2-(4-(trifluoromethyl)phenyl)-1,3,2-dioxaborolane (326 mg, 1.2 mmol, 1.2 equiv.) and trifluoroacetamide **1z** (247 mg, 1.0 mmol, 1.0 equiv.). The purification was accomplished by column chromatography on silica gel with Hexane/Ethyl acetate 5:1 as eluent to provide the desired amide **2ae** (255 mg, 0.79 mmol, 79%).

White solid, mp 155 - 156 ^{o}C. **^{1}H NMR** (500 MHz, DMSO-d_6): δ = 7.92 (d, ^{3}J=8.1 Hz, 2H), 7.83 (s, 1H), 7.73 (d, ^{3}J=8.3 Hz, 2H), 2.04 (s, 6H), 2.01 (s, 3H), 1.61 (s, 6H).

^{13}C{^{1}H} NMR (126 MHz, DMSO-d_6): δ= 164.9, 139.8, 130.7 (q, $^{2}J_{CF}$ = 31.8 Hz), 128.3, 124.9 (m), 124.0 (q, $^{1}J_{CF}$ = 274.6 Hz), 51.8, 40.8, 36.1, 28.9.

MS (GC, 70eV): m/z (%) = 323 (M^+, 47), 266 (100), 173 (72), 145 (38).

Anal. calcd. for $C_{18}H_{20}F_3NO$: C, 66.86; H, 6.23; N, 4.33. Found: C, 66.72; H, 6.31; N, 4.35.

(D) Copies ^{1}H and ^{13}C NMR spectra

Compound **2a**

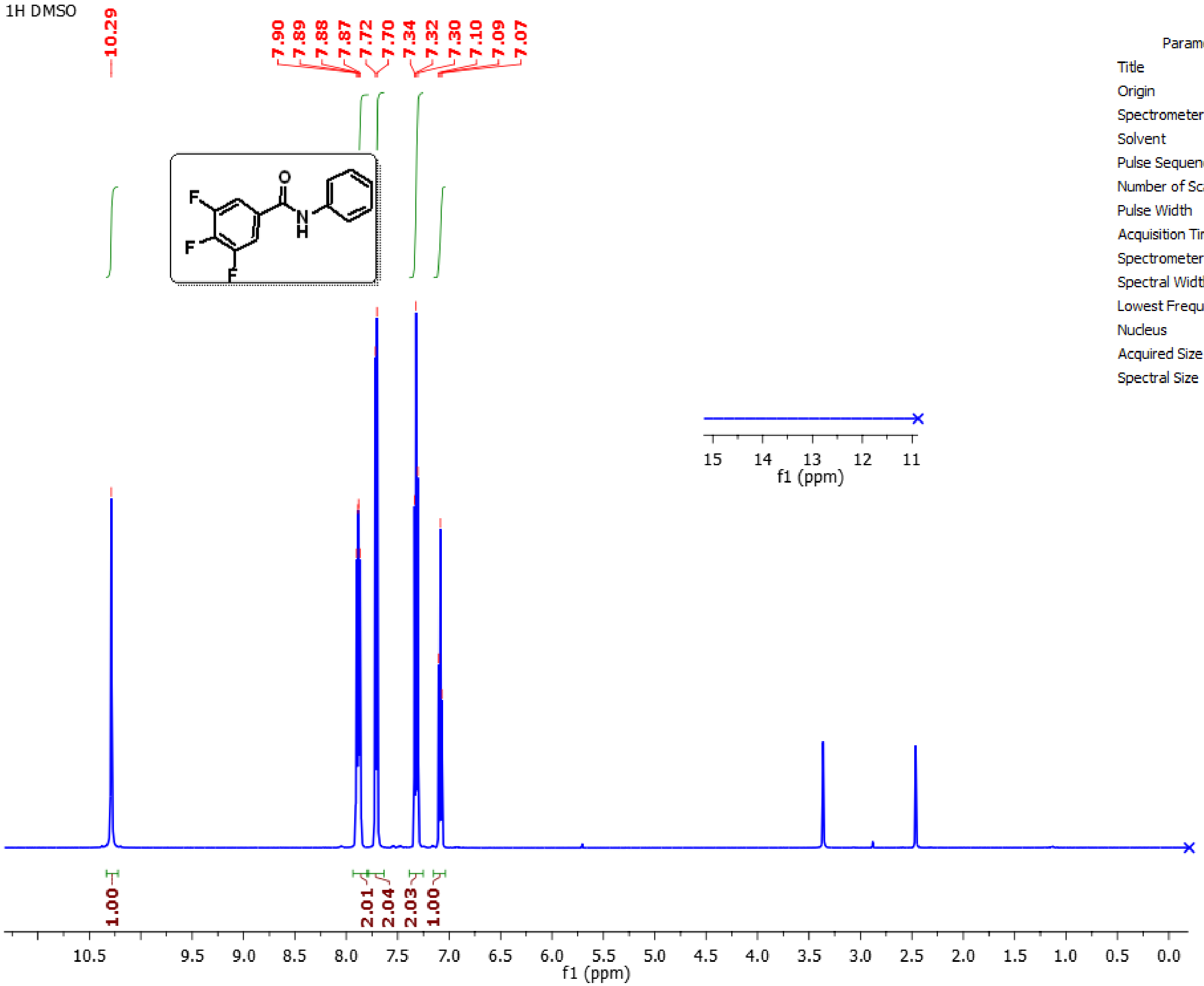

Parameters

Parameter	Value
Title	IVA 2378
Origin	Bruker BioSpin GmbH
Spectrometer	AV_III_500
Solvent	DMSO
Pulse Sequence	zg30
Number of Scans	24
Pulse Width	9.9500
Acquisition Time	2.6564
Spectrometer Frequency	500.13
Spectral Width	12335.5
Lowest Frequency	-3190.6
Nucleus	1H
Acquired Size	32768
Spectral Size	65536

Compound **2a**

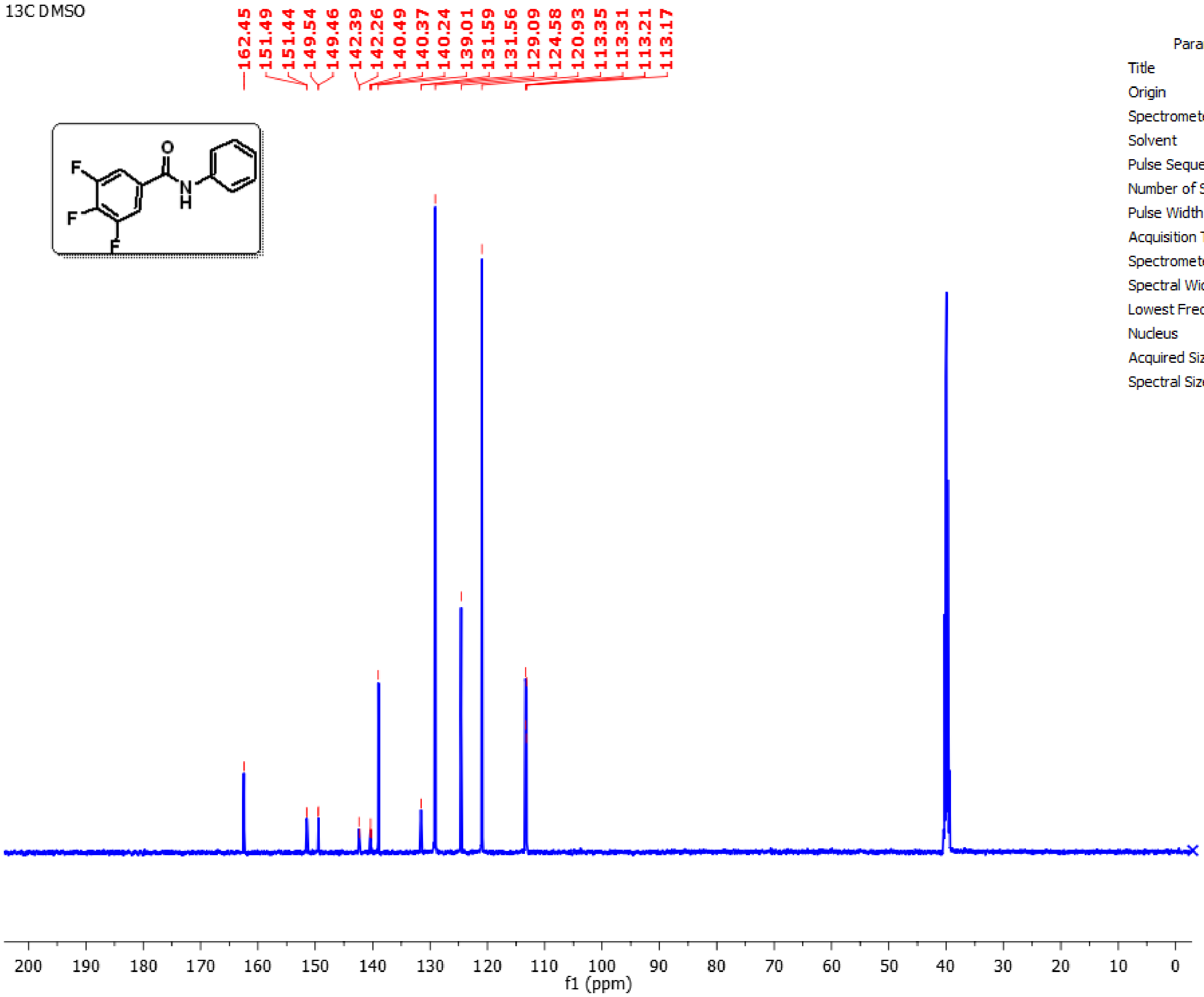

Parameters

Parameter	Value
Title	IVA 2378
Origin	Bruker BioSpin GmbH
Spectrometer	AV_III_500
Solvent	DMSO
Pulse Sequence	zgpg30
Number of Scans	512
Pulse Width	11.0000
Acquisition Time	0.9088
Spectrometer Frequency	125.76
Spectral Width	36057.7
Lowest Frequency	-2939.0
Nucleus	13C
Acquired Size	32768
Spectral Size	65536

Compound **2b**

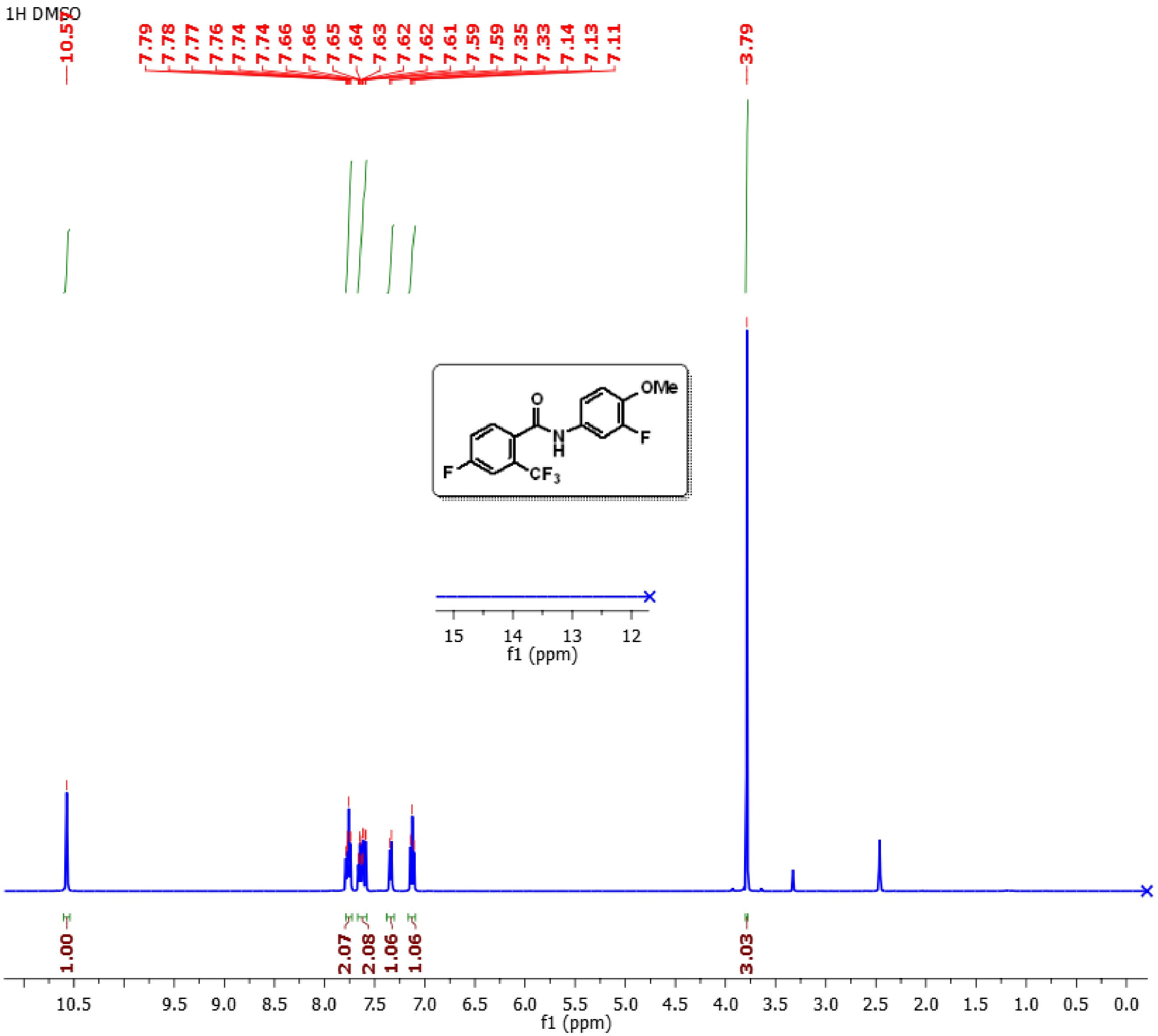

Parameters

Parameter	Value
Title	IVA 1751
Origin	Bruker BioSpin GmbH
Spectrometer	AV_III_500
Solvent	DMSO
Pulse Sequence	zg30
Number of Scans	24
Pulse Width	9.9500
Acquisition Time	2.6564
Spectrometer Frequency	500.13
Spectral Width	12335.5
Lowest Frequency	-3190.6
Nucleus	1H
Acquired Size	32768
Spectral Size	65536

Compound **2b**

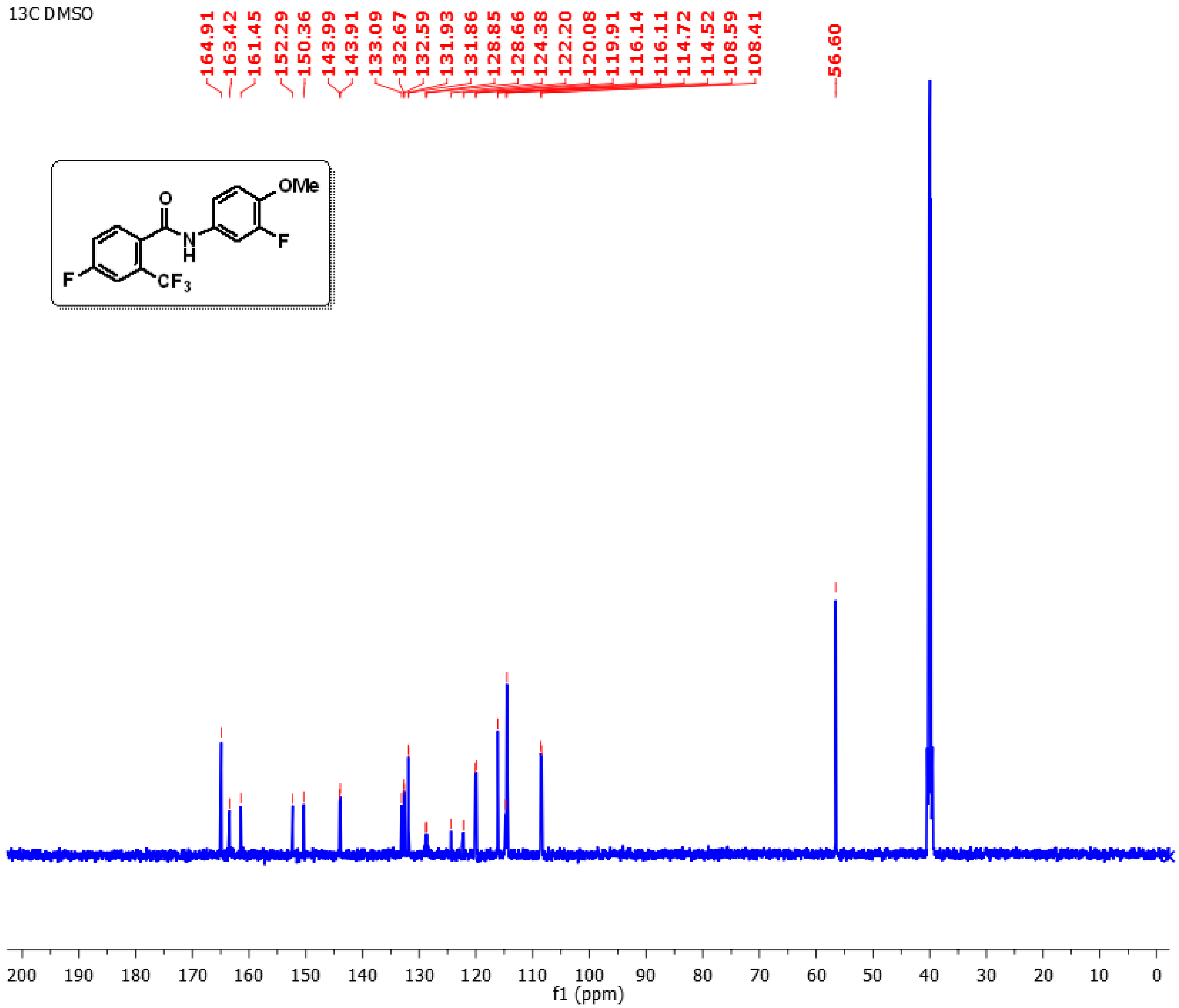

Parameters

Parameter	Value
Title	IVA 1751
Origin	Bruker BioSpin GmbH
Spectrometer	AV_III_500
Solvent	DMSO
Pulse Sequence	zgpg30
Number of Scans	256
Pulse Width	11.0000
Acquisition Time	0.9088
Spectrometer Frequency	125.76
Spectral Width	36057.7
Lowest Frequency	-2939.0
Nucleus	13C
Acquired Size	32768
Spectral Size	65536

Compound **2c**

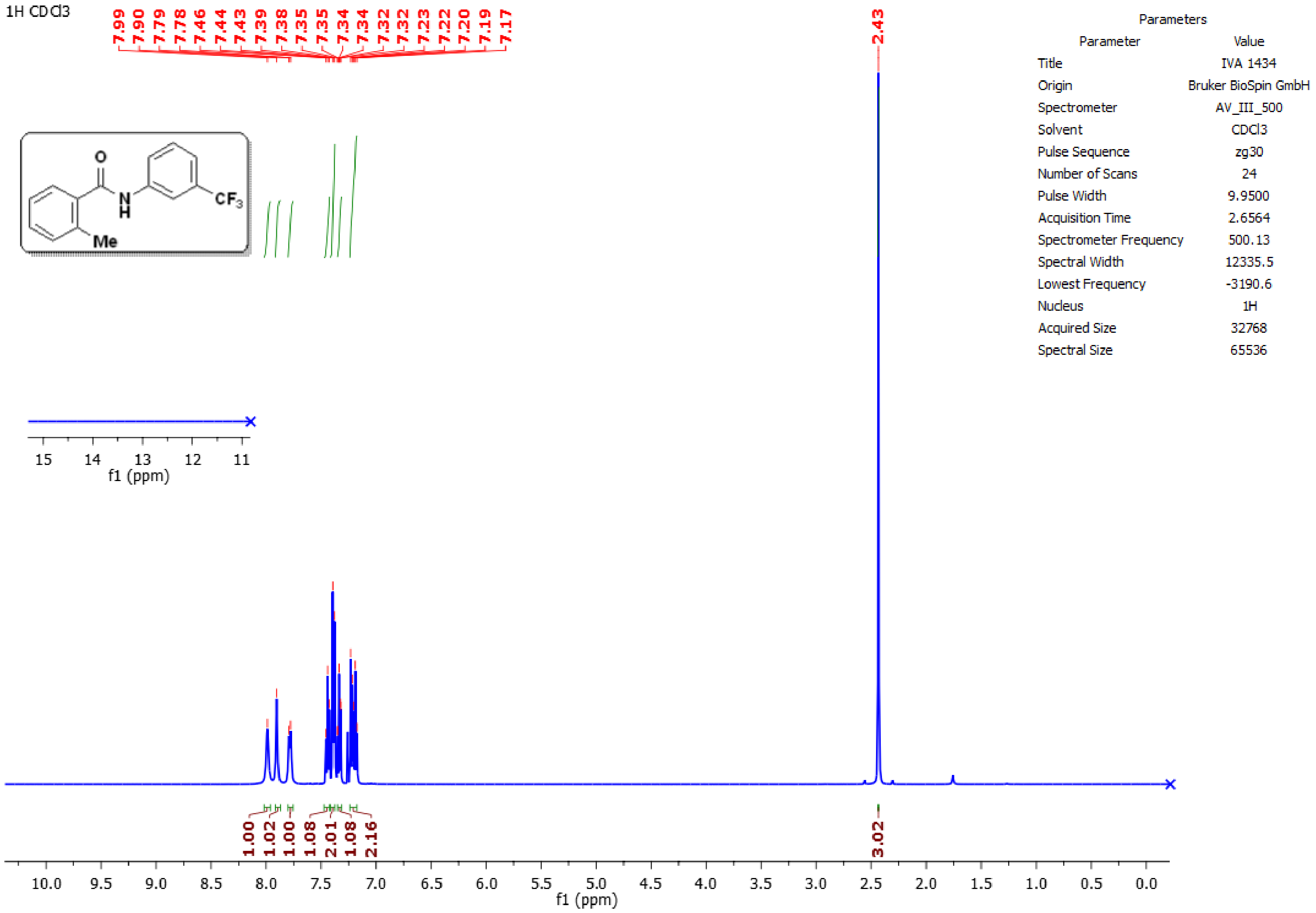

1H CDCl3
7.99 7.90 7.79 7.78 7.46 7.44 7.43 7.39 7.38 7.35 7.35 7.34 7.34 7.32 7.32 7.23 7.22 7.20 7.19 7.17
2.43
O
N
H
CF3
Me
15 14 13 12 11
f1 (ppm)
1.00 1.02 1.00 1.08 2.01 1.08 2.16
3.02
10.0 9.5 9.0 8.5 8.0 7.5 7.0 6.5 6.0 5.5 5.0 4.5 4.0 3.5 3.0 2.5 2.0 1.5 1.0 0.5 0.0
f1 (ppm)
Parameters
Parameter Value
Title IVA 1434
Origin Bruker BioSpin GmbH
Spectrometer AV_III_500
Solvent CDCl3
Pulse Sequence zg30
Number of Scans 24
Pulse Width 9.9500
Acquisition Time 2.6564
Spectrometer Frequency 500.13
Spectral Width 12335.5
Lowest Frequency -3190.6
Nucleus 1H
Acquired Size 32768
Spectral Size 65536

Compound **2c**

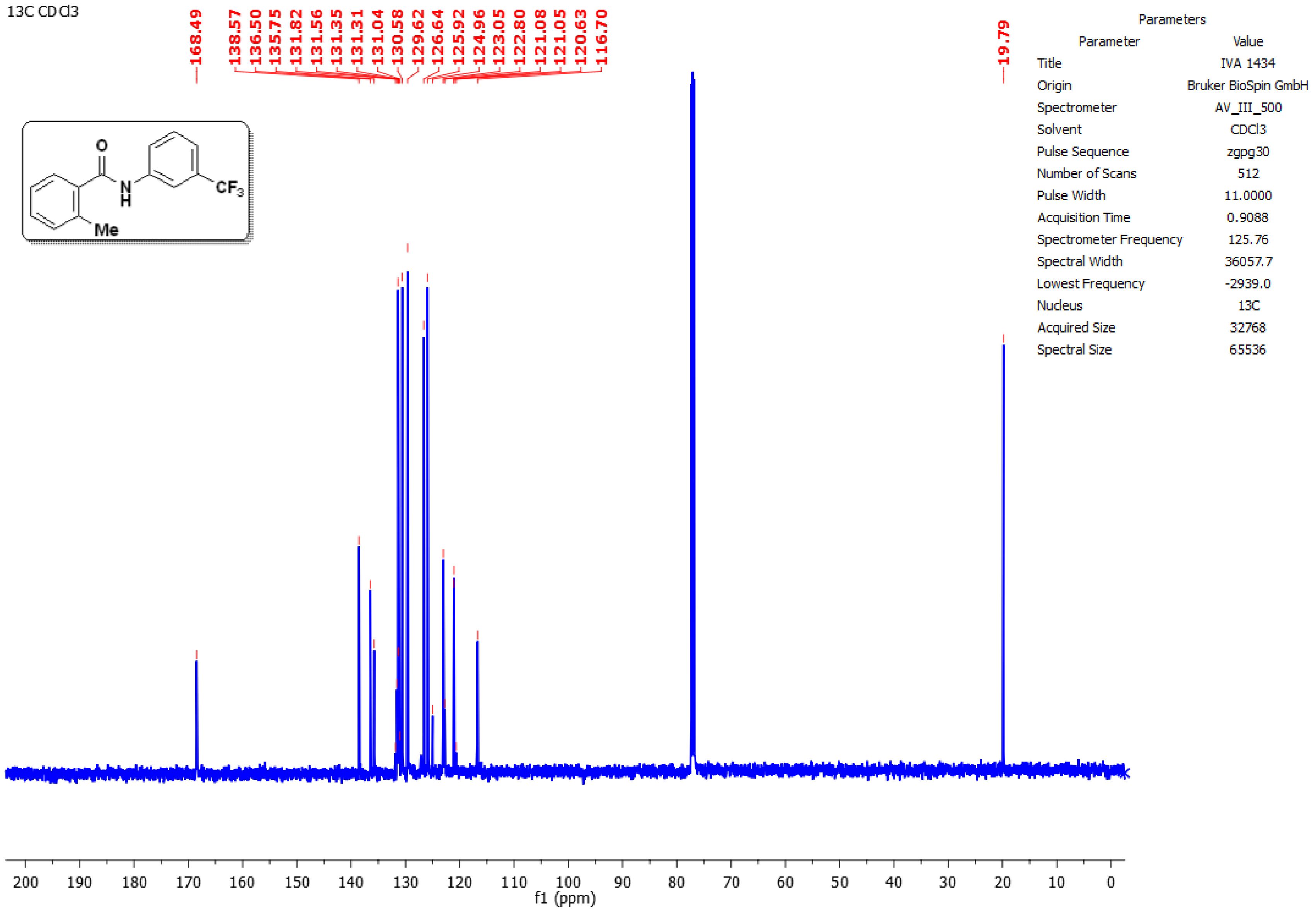

13C CDCl3
168.49
138.57
136.50
135.75
131.82
131.56
131.35
131.31
131.04
130.58
129.62
126.64
125.92
124.96
123.05
122.80
121.08
121.05
120.63
116.70
19.79
O
N
H
CF3
Me
Parameters
Parameter
Value
Title
IVA 1434
Origin
Bruker BioSpin GmbH
Spectrometer
AV_III_500
Solvent
CDCl3
Pulse Sequence
zgpg30
Number of Scans
512
Pulse Width
11.0000
Acquisition Time
0.9088
Spectrometer Frequency
125.76
Spectral Width
36057.7
Lowest Frequency
-2939.0
Nucleus
13C
Acquired Size
32768
Spectral Size
65536
200 190 180 170 160 150 140 130 120 110 100 90 80 70 60 50 40 30 20 10 0
f1 (ppm)

Compound **2d**

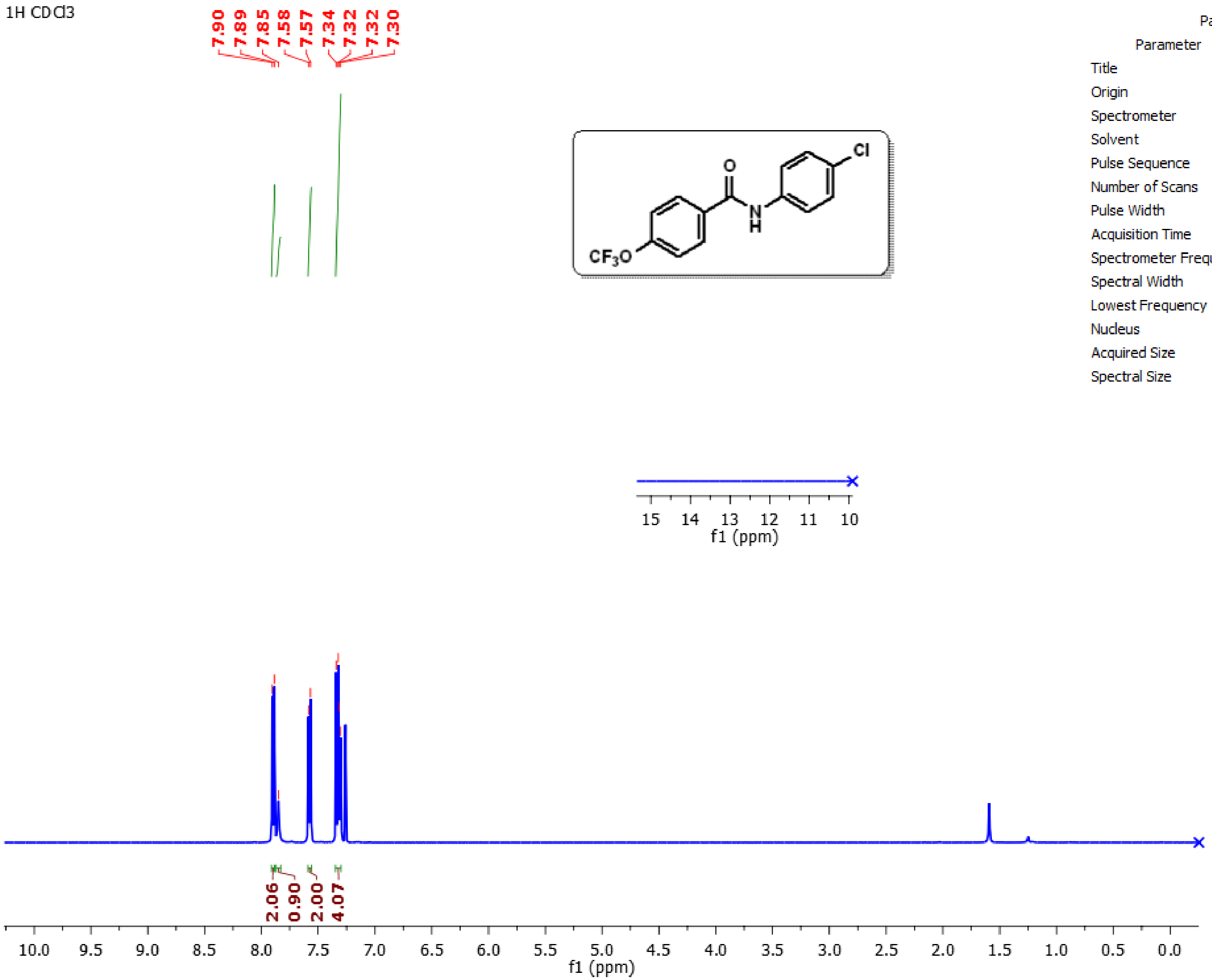

Parameters

Parameter	Value
Title	IVA 935
Origin	Bruker BioSpin GmbH
Spectrometer	AV_III_500
Solvent	CDCl3
Pulse Sequence	zg30
Number of Scans	24
Pulse Width	9.9500
Acquisition Time	2.6564
Spectrometer Frequency	500.13
Spectral Width	12335.5
Lowest Frequency	-3190.6
Nucleus	1H
Acquired Size	32768
Spectral Size	65536

Compound **2d**

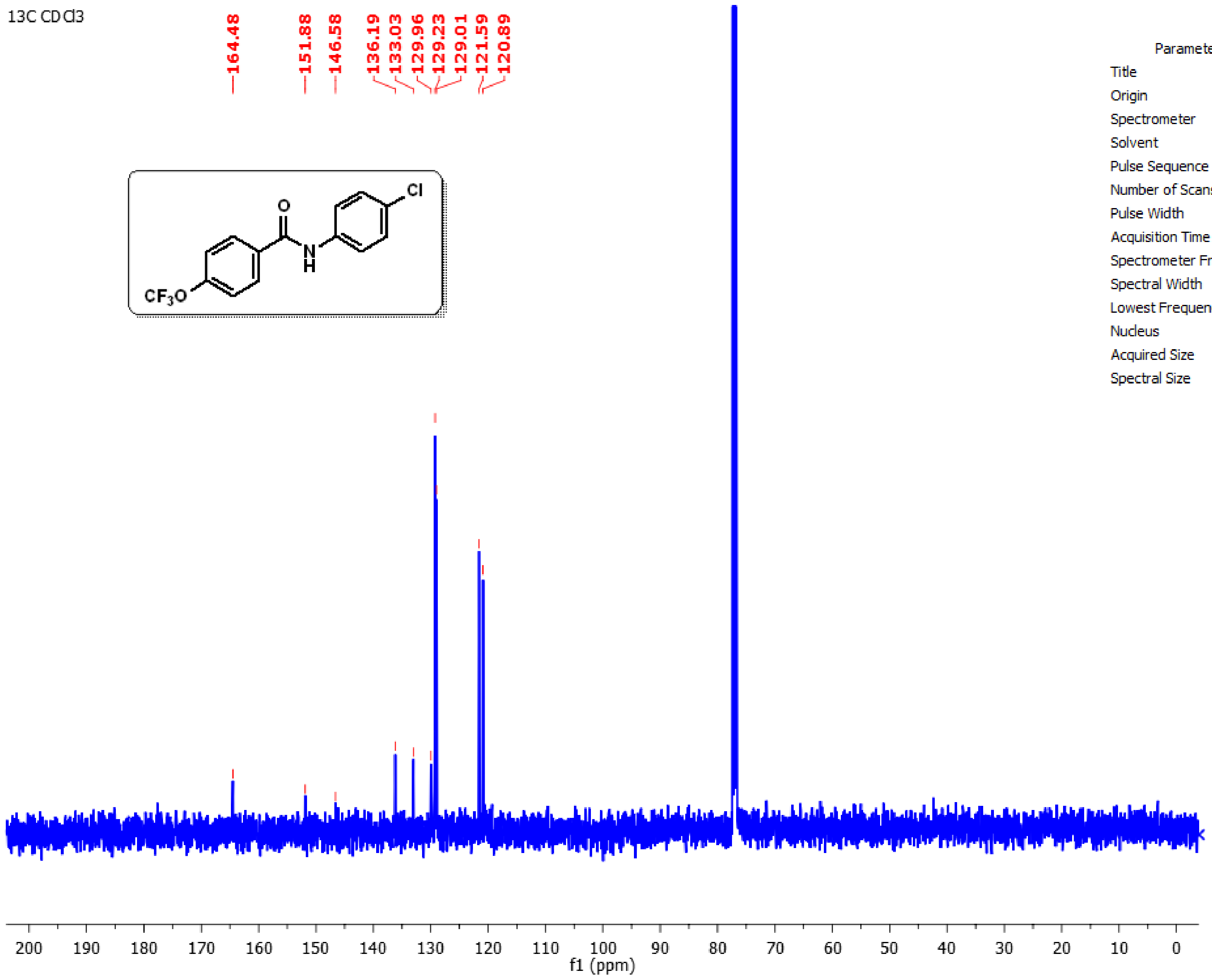

Parameters

Parameter	Value
Title	IVA 935
Origin	Bruker BioSpin GmbH
Spectrometer	AV_III_500
Solvent	CDCl3
Pulse Sequence	zgpg30
Number of Scans	236
Pulse Width	11.0000
Acquisition Time	0.9088
Spectrometer Frequency	125.76
Spectral Width	36057.7
Lowest Frequency	-2939.0
Nucleus	13C
Acquired Size	32768
Spectral Size	65536

Compound **2e**

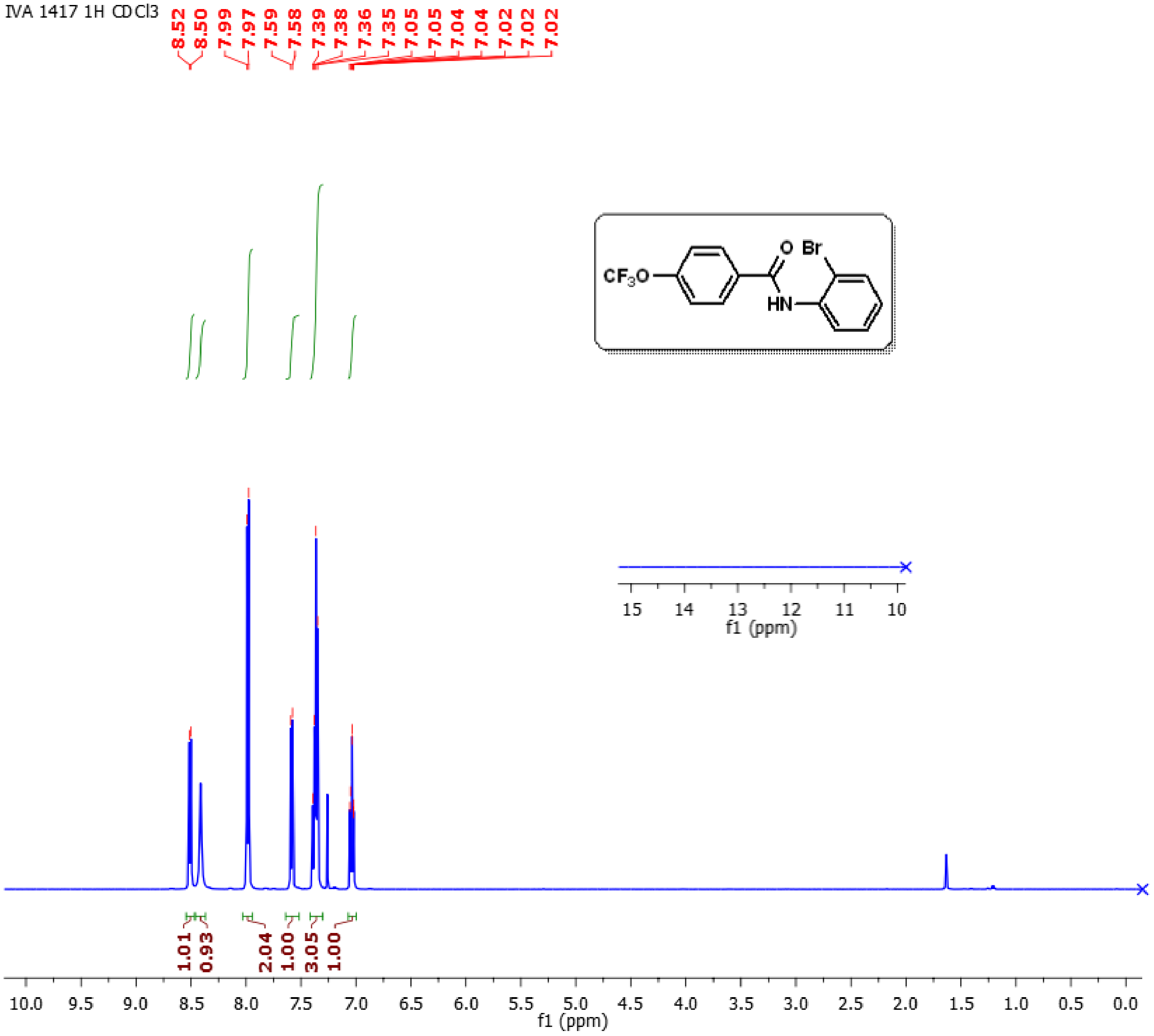

Parameters

Parameter	Value
Title	IVA 1417
Origin	Bruker BioSpin GmbH
Spectrometer	AV_III_500
Solvent	CDCl3
Pulse Sequence	zg30
Number of Scans	24
Pulse Width	9.9500
Acquisition Time	2.6564
Spectrometer Frequency	500.13
Spectral Width	12335.5
Lowest Frequency	-3190.6
Nucleus	1H
Acquired Size	32768
Spectral Size	65536

Compound **2e**

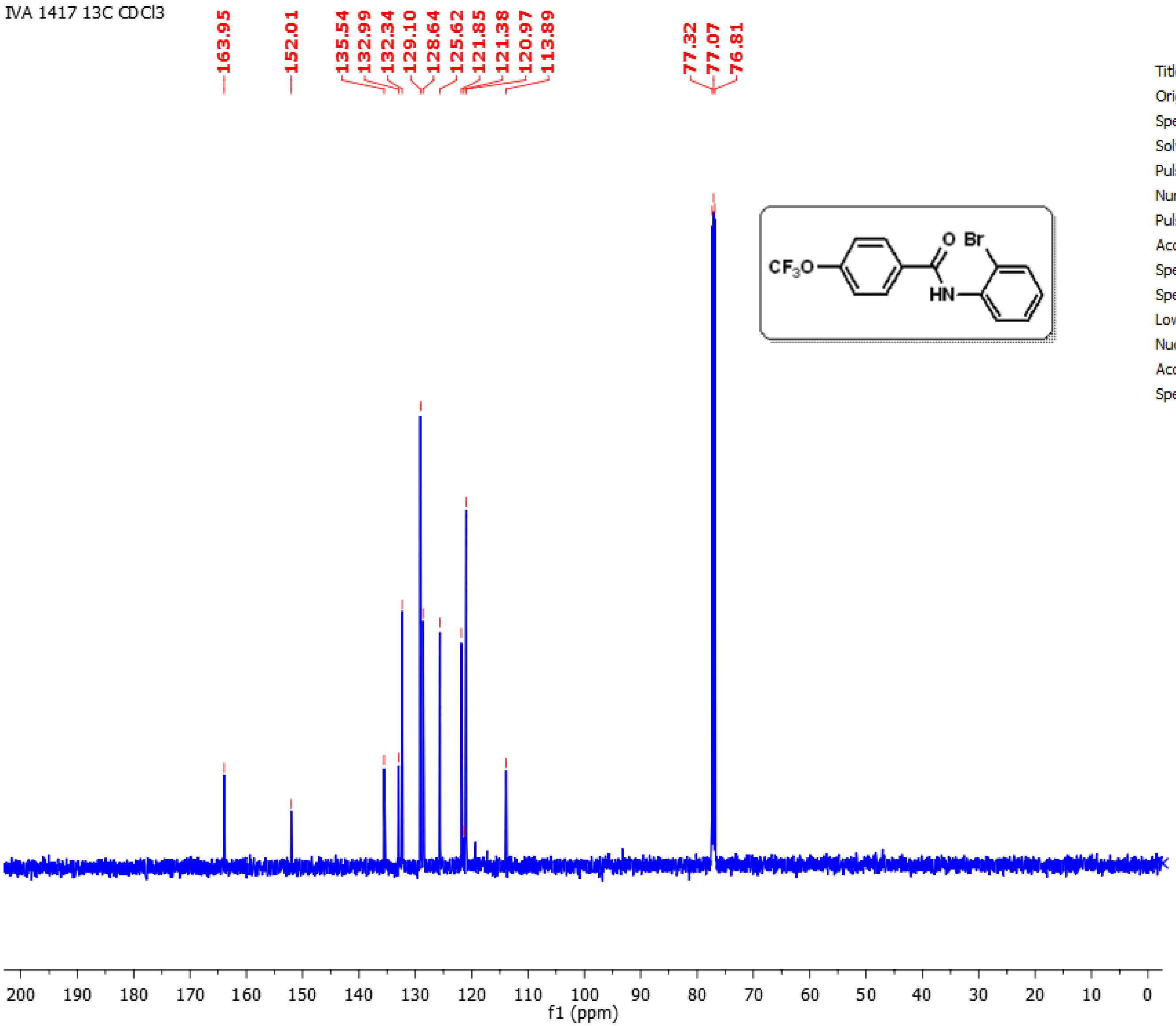

Parameters

Parameter	Value
Title	IVA 1417
Origin	Bruker BioSpin GmbH
Spectrometer	AV_III_500
Solvent	CDCl3
Pulse Sequence	zgpg30
Number of Scans	256
Pulse Width	11.0000
Acquisition Time	0.9088
Spectrometer Frequency	125.76
Spectral Width	36057.7
Lowest Frequency	-2939.0
Nucleus	13C
Acquired Size	32768
Spectral Size	65536

Compound **2f**

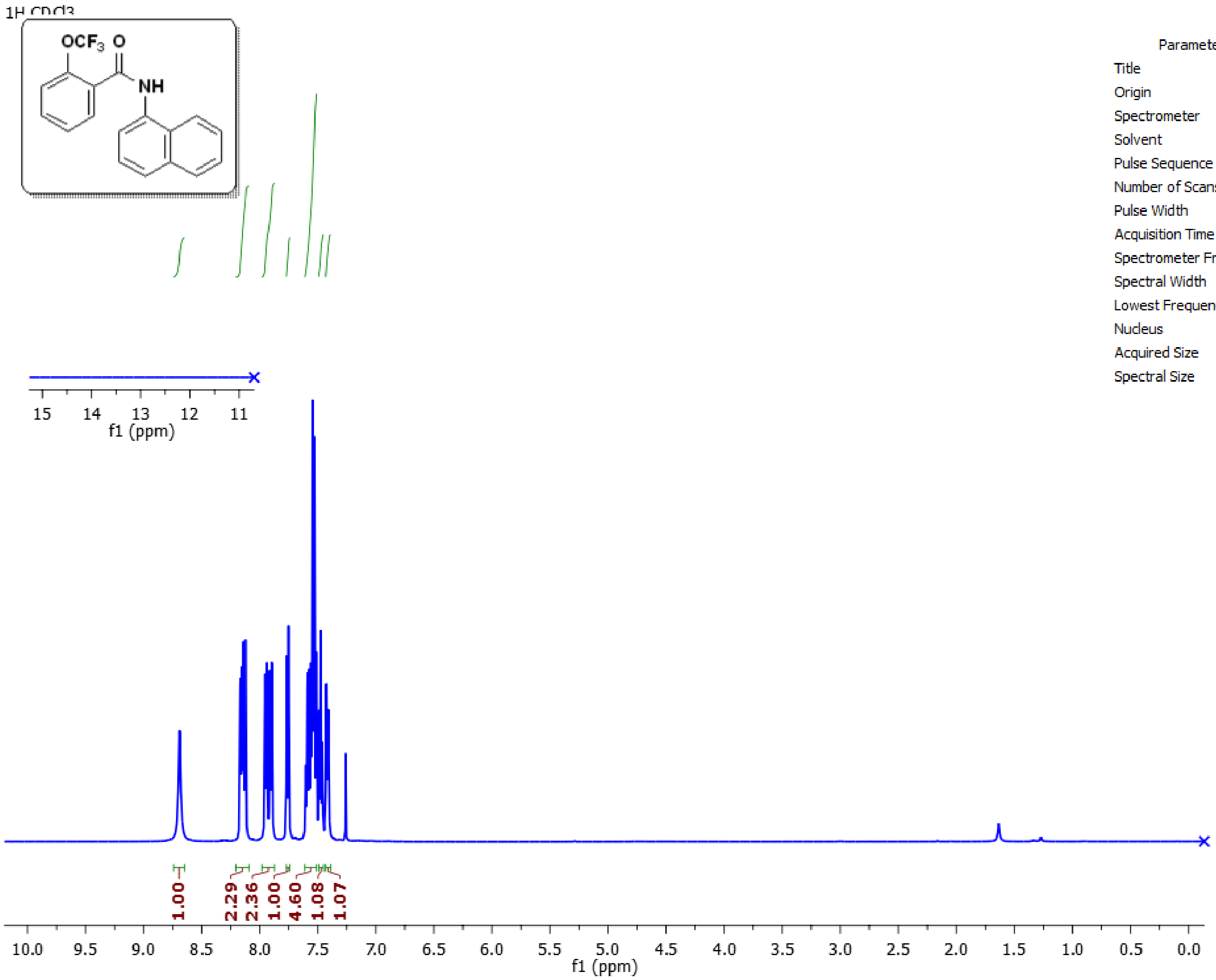

Parameters

Parameter	Value
Title	IVA 1439
Origin	Bruker BioSpin GmbH
Spectrometer	AV_III_500
Solvent	CDCl3
Pulse Sequence	zg30
Number of Scans	24
Pulse Width	9.9500
Acquisition Time	2.6564
Spectrometer Frequency	500.13
Spectral Width	12335.5
Lowest Frequency	-3190.6
Nucleus	1H
Acquired Size	32768
Spectral Size	65536

Compound **2f**

13C CDCl3

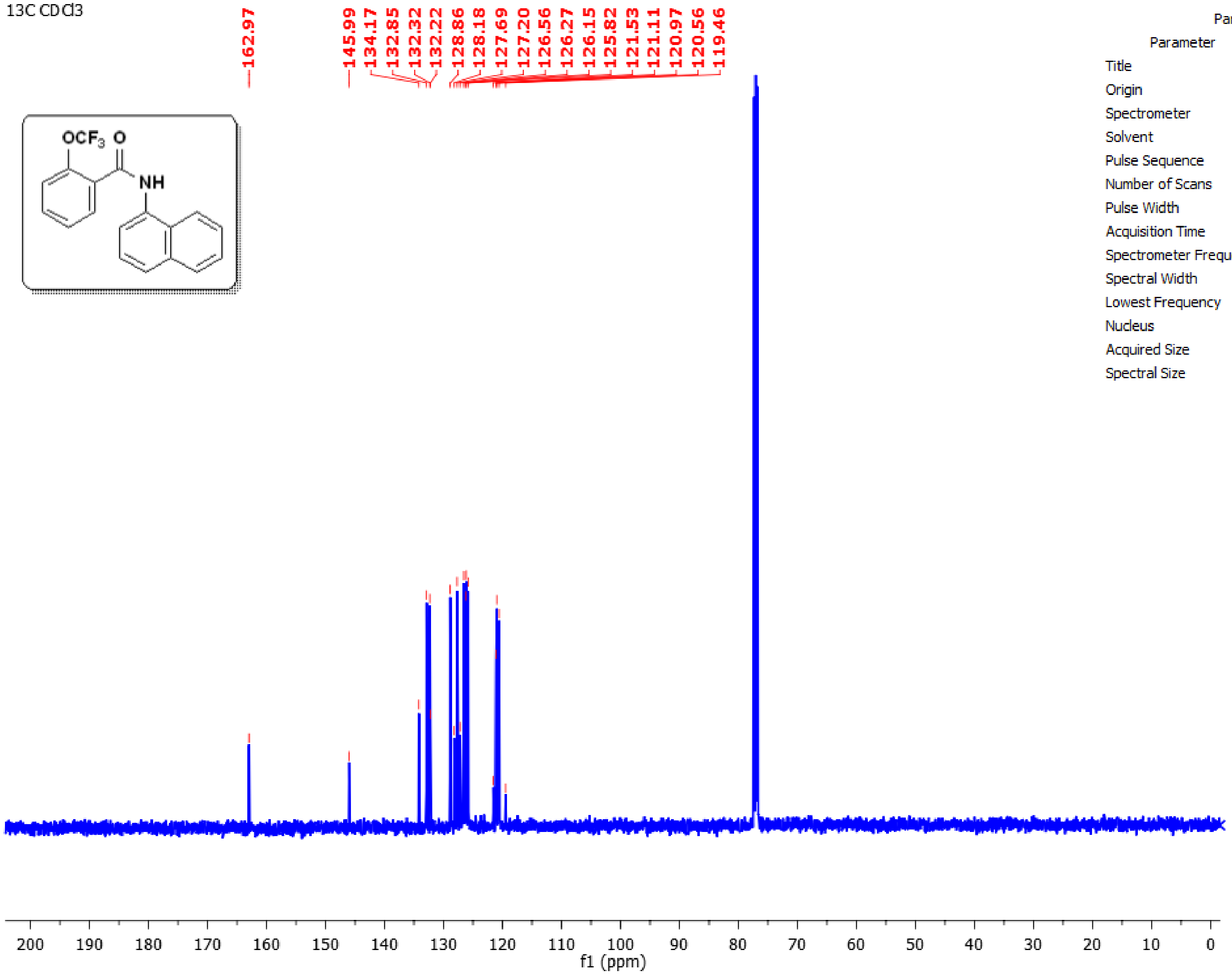

Parameters

Parameter	Value
Title	IVA 1439
Origin	Bruker BioSpin GmbH
Spectrometer	AV_III_500
Solvent	CDCl3
Pulse Sequence	zgpg30
Number of Scans	512
Pulse Width	11.0000
Acquisition Time	0.9088
Spectrometer Frequency	125.76
Spectral Width	36057.7
Lowest Frequency	-2939.0
Nucleus	13C
Acquired Size	32768
Spectral Size	65536

Compound **2g**

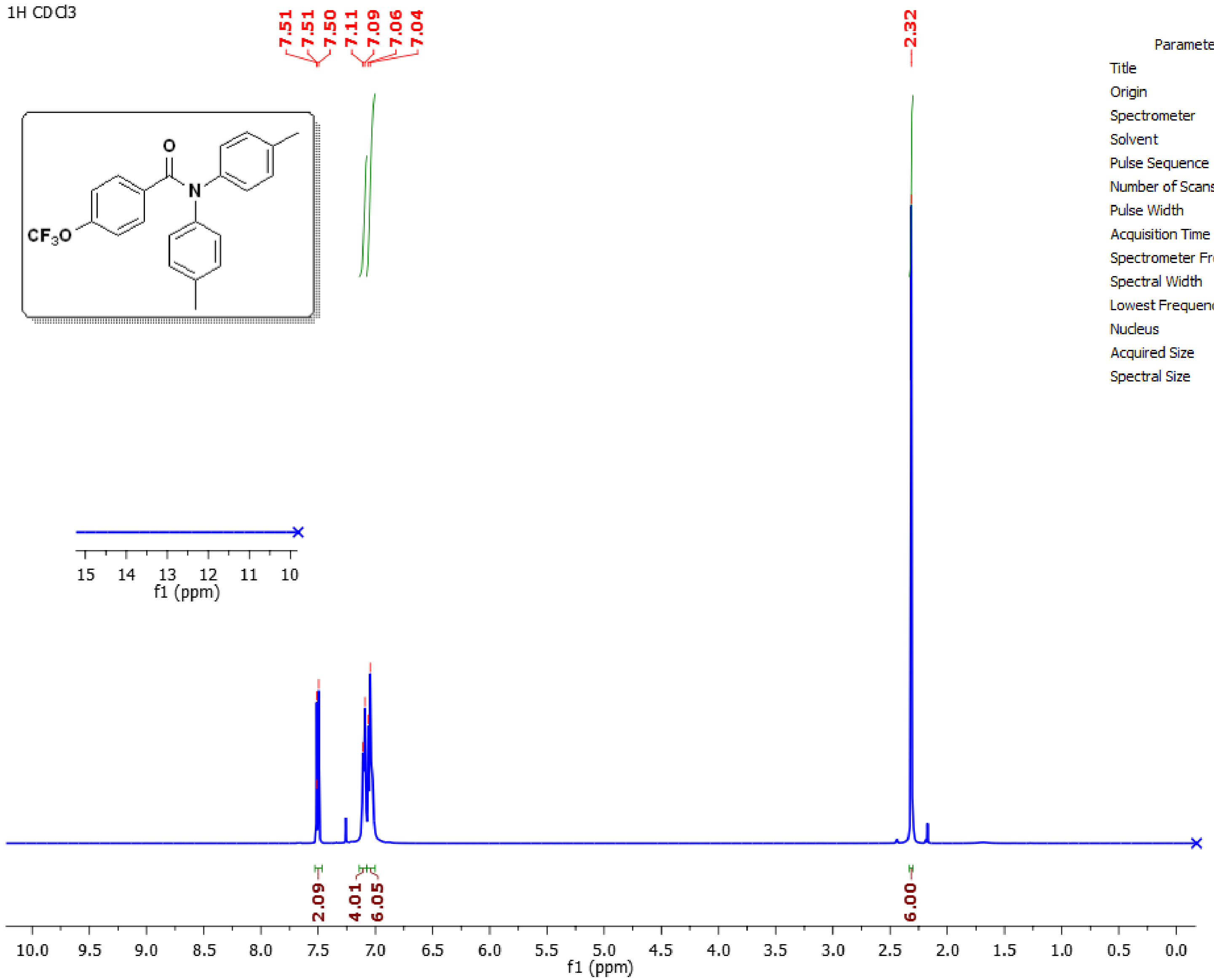

Parameters

Parameter	Value
Title	IVA 2837
Origin	Bruker BioSpin GmbH
Spectrometer	AV_III_500
Solvent	CDCl3
Pulse Sequence	zg30
Number of Scans	24
Pulse Width	9.9500
Acquisition Time	2.6564
Spectrometer Frequency	500.13
Spectral Width	12335.5
Lowest Frequency	-3190.6
Nucleus	1H
Acquired Size	32768
Spectral Size	65536

Compound **2g**

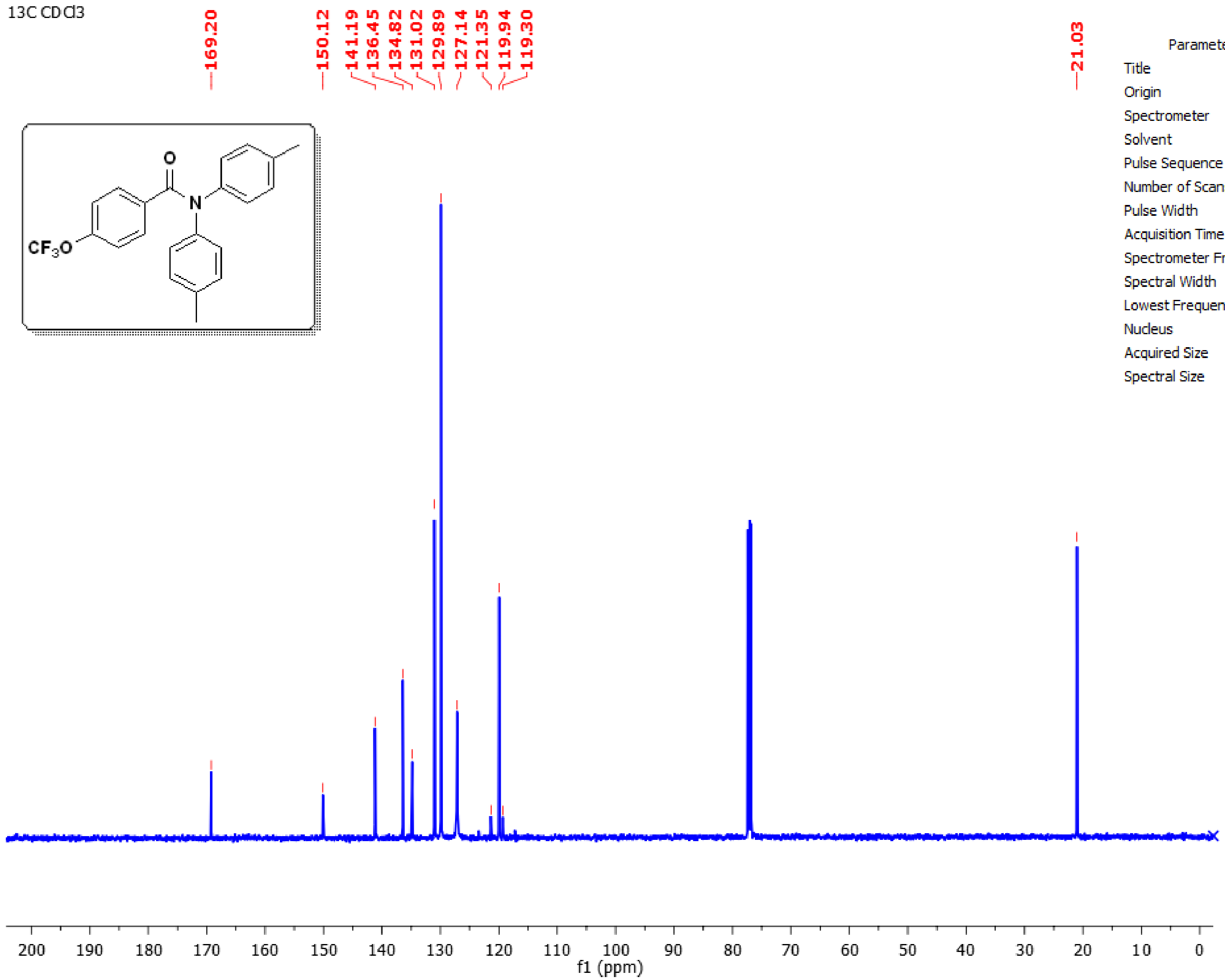

Parameter	Value
Title	IVA 2837
Origin	Bruker BioSpin GmbH
Spectrometer	AV_III_500
Solvent	CDCl3
Pulse Sequence	zgpg30
Number of Scans	512
Pulse Width	11.0000
Acquisition Time	0.9088
Spectrometer Frequency	125.76
Spectral Width	36057.7
Lowest Frequency	-2939.0
Nucleus	13C
Acquired Size	32768
Spectral Size	65536

Compound **2h**

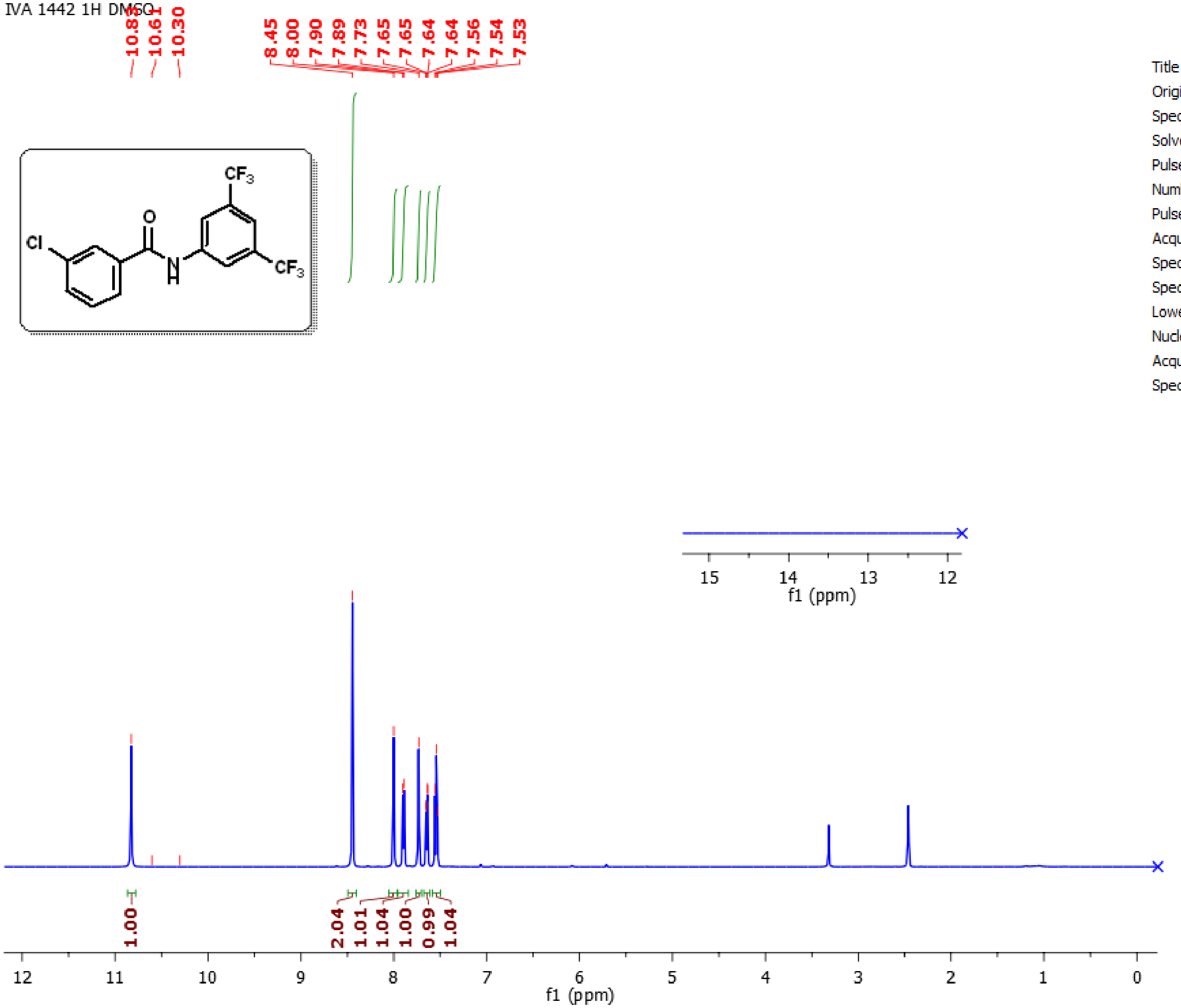

Parameters

Parameter	Value
Title	IVA 1442
Origin	Bruker BioSpin GmbH
Spectrometer	AV_III_500
Solvent	DMSO
Pulse Sequence	zg30
Number of Scans	24
Pulse Width	9.9500
Acquisition Time	2.6564
Spectrometer Frequency	500.13
Spectral Width	12335.5
Lowest Frequency	-3190.6
Nucleus	1H
Acquired Size	32768
Spectral Size	65536

Compound **2h**

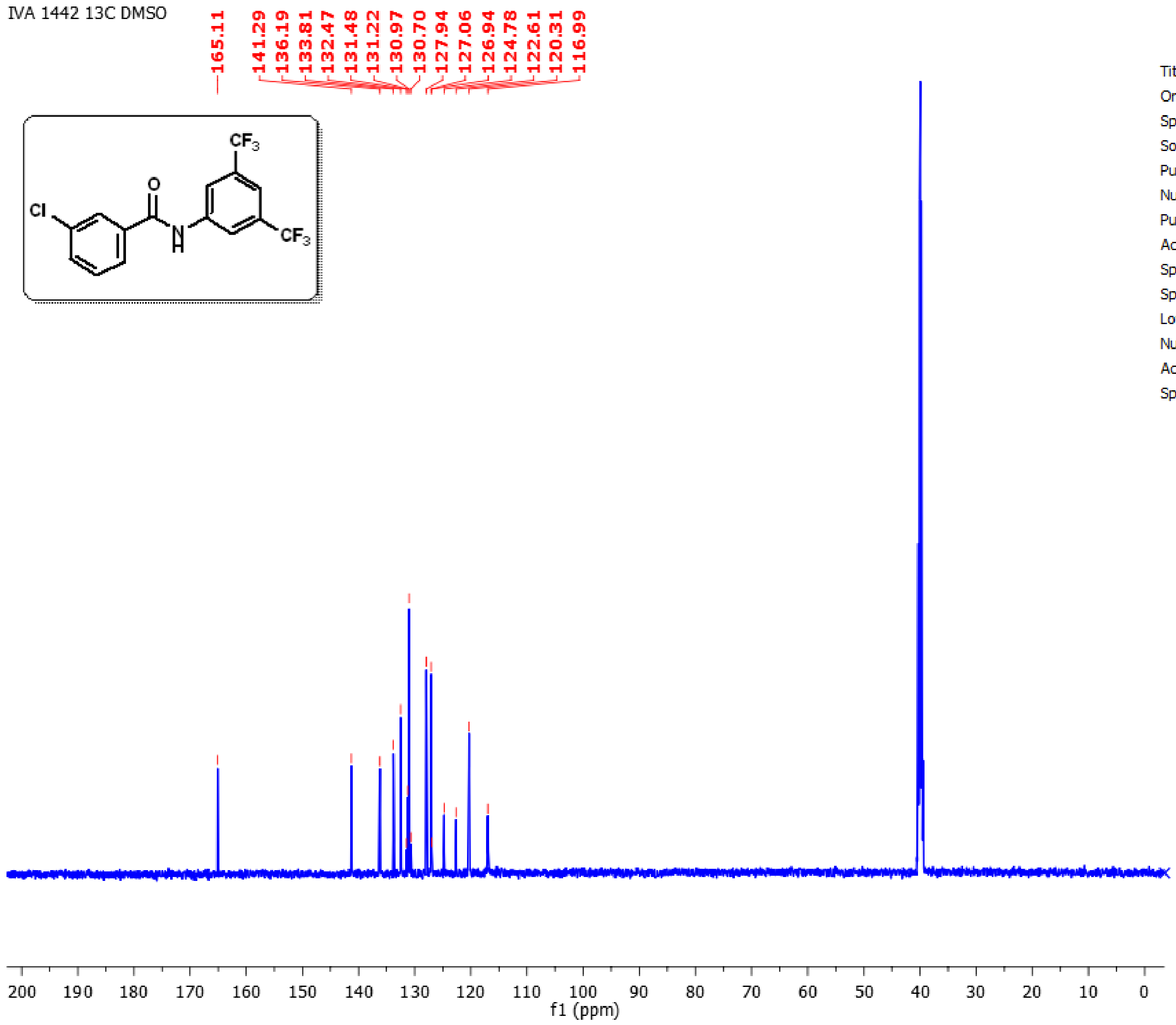

Parameter	Value
Title	IVA 1442
Origin	Bruker BioSpin GmbH
Spectrometer	AV_III_500
Solvent	DMSO
Pulse Sequence	zgpg30
Number of Scans	512
Pulse Width	11.0000
Acquisition Time	0.9088
Spectrometer Frequency	125.76
Spectral Width	36057.7
Lowest Frequency	-2939.0
Nucleus	13C
Acquired Size	32768
Spectral Size	65536

Compound **2i**

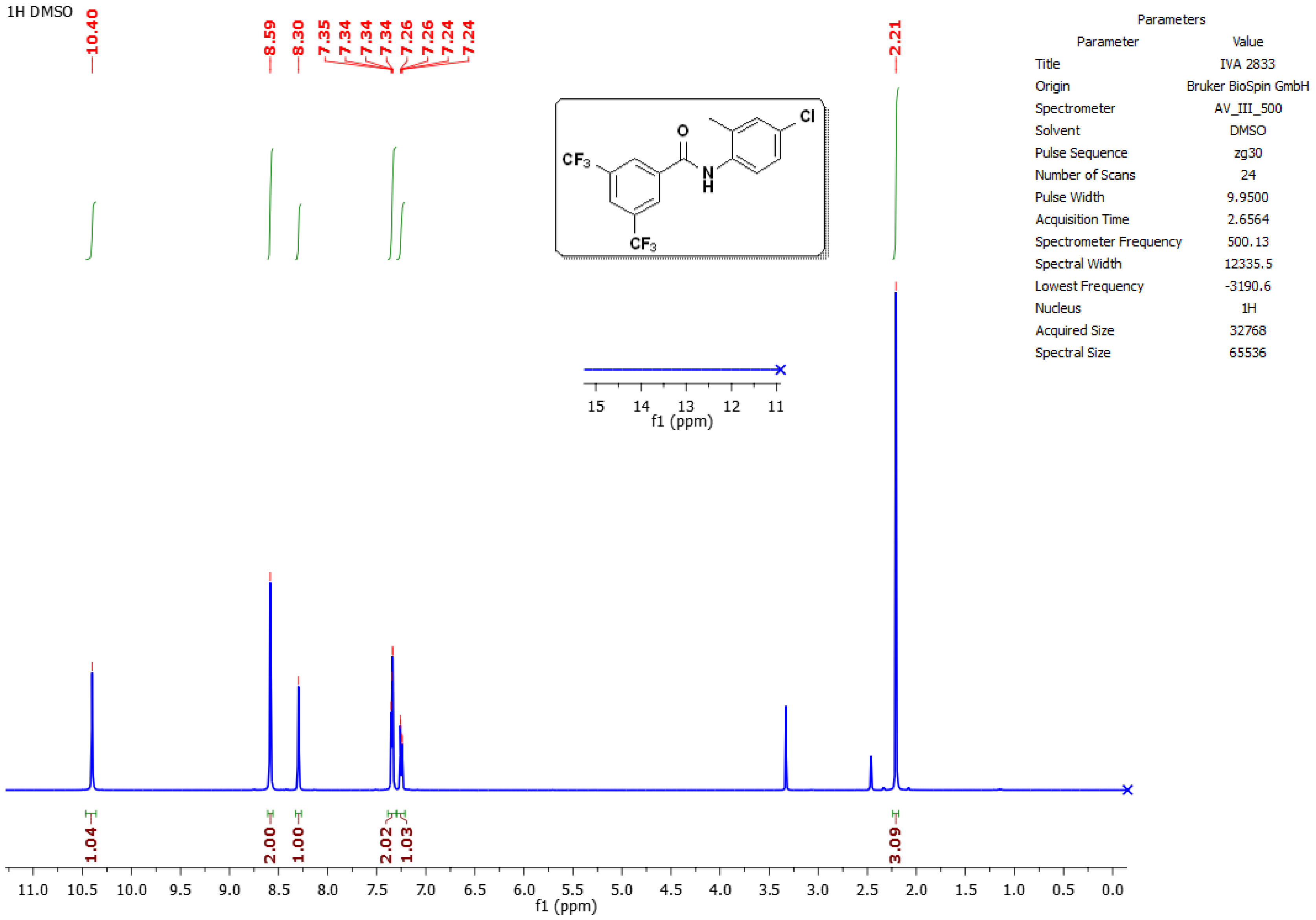

1H DMSO
10.40
8.59
8.30
7.35
7.34
7.34
7.34
7.26
7.26
7.24
7.24
2.21
CF3
CF3
O
N
H
Cl
15
14
13
12
11
f1 (ppm)
1.04
2.00
1.00
2.02
1.03
3.09
11.0
10.5
10.0
9.5
9.0
8.5
8.0
7.5
7.0
6.5
6.0
5.5
5.0
4.5
4.0
3.5
3.0
2.5
2.0
1.5
1.0
0.5
0.0
f1 (ppm)
Parameters
Parameter
Value
Title
IVA 2833
Origin
Bruker BioSpin GmbH
Spectrometer
AV_III_500
Solvent
DMSO
Pulse Sequence
zg30
Number of Scans
24
Pulse Width
9.9500
Acquisition Time
2.6564
Spectrometer Frequency
500.13
Spectral Width
12335.5
Lowest Frequency
-3190.6
Nucleus
1H
Acquired Size
32768
Spectral Size
65536

Compound **2i**

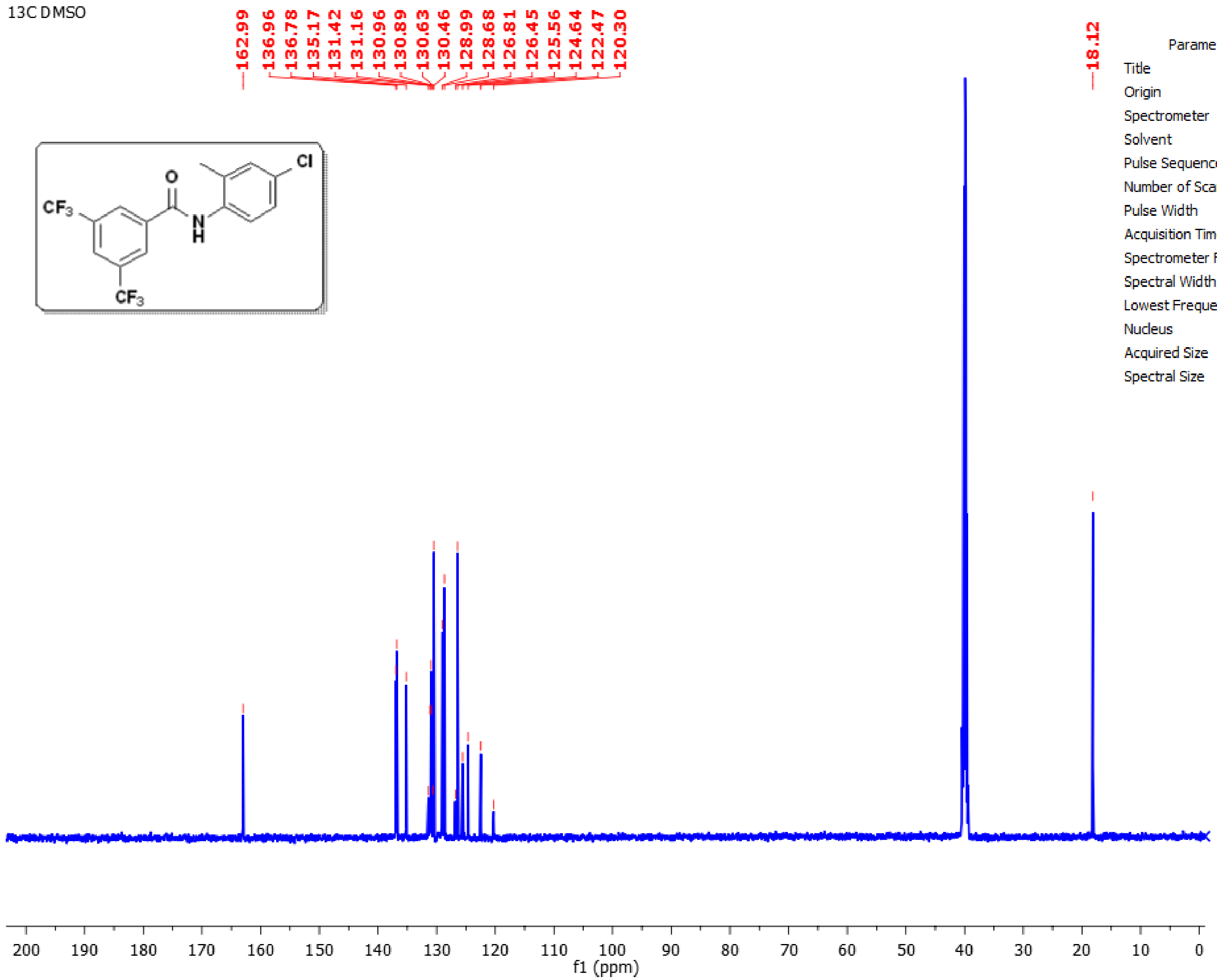

Parameters

Parameter	Value
Title	IVA 2833
Origin	Bruker BioSpin GmbH
Spectrometer	AV_III_500
Solvent	DMSO
Pulse Sequence	zgpg30
Number of Scans	512
Pulse Width	11.0000
Acquisition Time	0.9088
Spectrometer Frequency	125.76
Spectral Width	36057.7
Lowest Frequency	-2939.0
Nucleus	13C
Acquired Size	32768
Spectral Size	65536

Compound **2j**

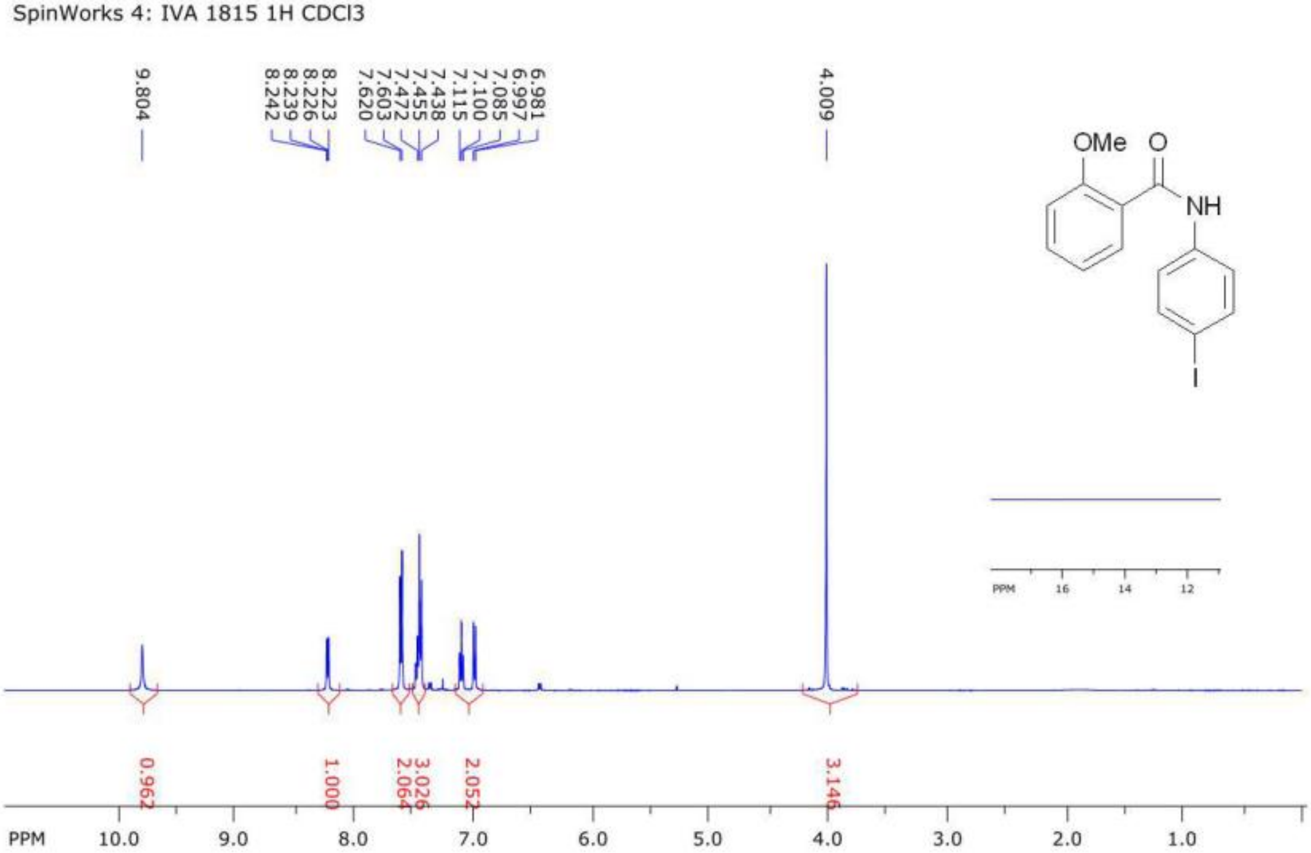

file: ...APO\NMR\500-2\mkr11706\21 1815\fid expt: <zg30>
transmitter freq.: 500.133001 MHz
time domain size: 65536 points
width: 12335.53 Hz = 24.6645 ppm = 0.188225 Hz/pt
number of scans: 24

freq. of 0 ppm: 500.130023 MHz
processed size: 65536 complex points
LB: 0.300 GF: 0.0000
Hz/cm: 219.965 ppm/cm: 0.43981

Compound **2j**

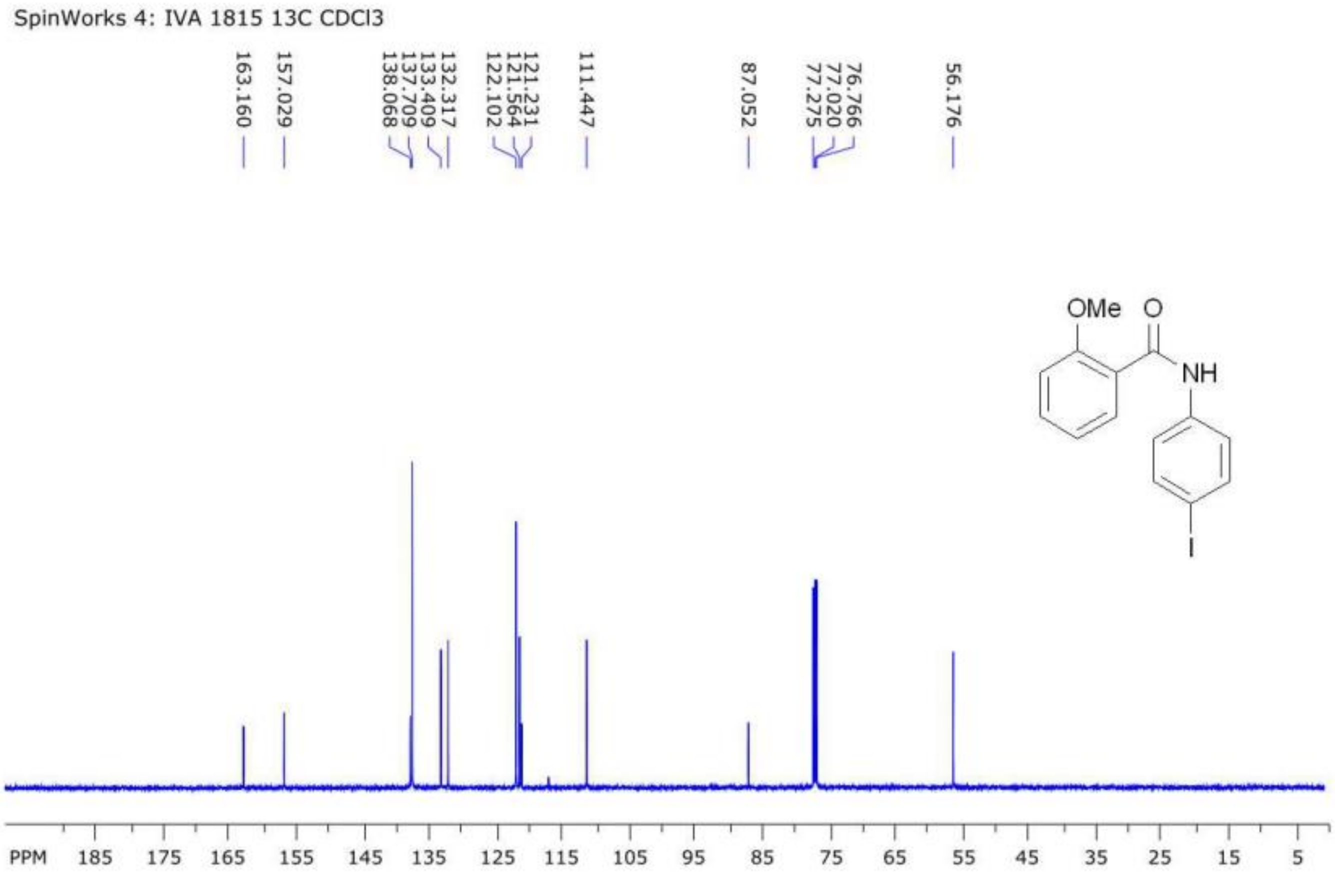

file: D:\NAPO\NMR\500-2\mkr11706\22\fid expt: <zgpg30>
transmitter freq.: 125.772879 MHz
time domain size: 65536 points
width: 36057.69 Hz = 286.6889 ppm = 0.550197 Hz/pt
number of scans: 512

freq. of 0 ppm: 125.757807 MHz
processed size: 32768 complex points
LB: 2.000 GF: 0.0000
Hz/cm: 1000.362 ppm/cm: 7.95372

Compound **2k**

SpinWorks 4: IVA 1748 1H DMSO

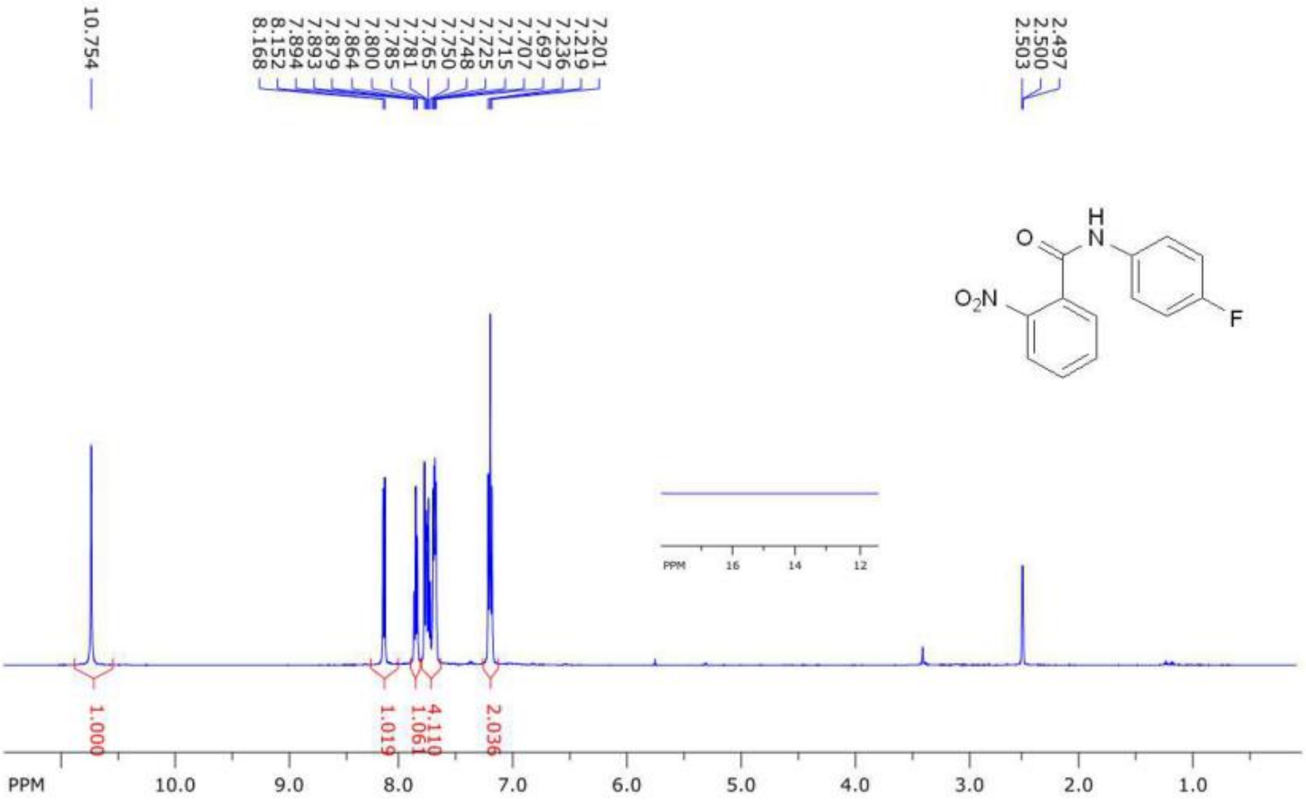

file: D:\NAPO\NMR\500-2\mkr10103\7\fid expt: <zg30>
transmitter freq.: 500.133001 MHz
time domain size: 65536 points
width: 12335.53 Hz = 24.6645 ppm = 0.188225 Hz/pt
number of scans: 24

freq. of 0 ppm: 500.130005 MHz
processed size: 65536 complex points
LB: 0.300 GF: 0.0000
Hz/cm: 229.244 ppm/cm: 0.45837

Compound **2k**

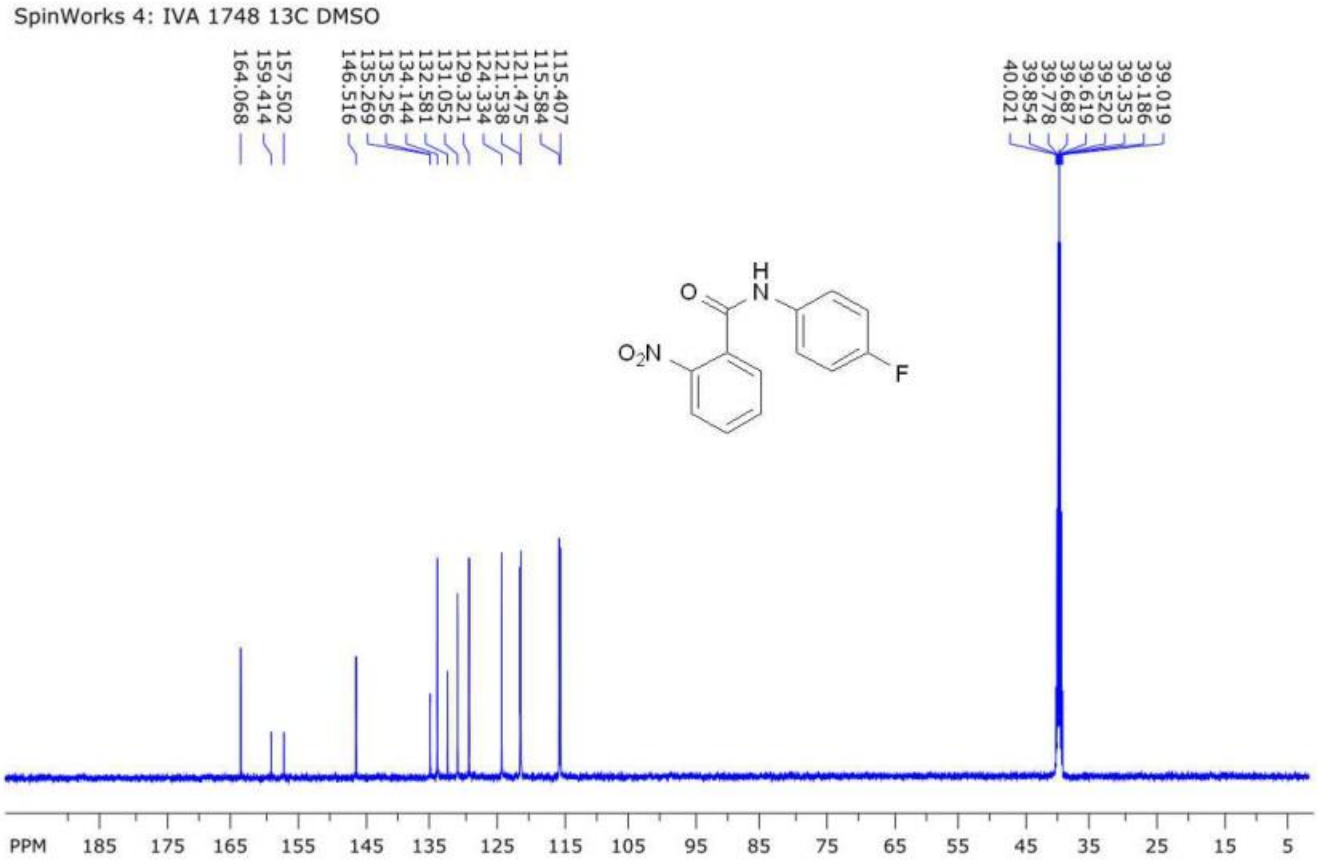

file: D:\NAPO\NMR\500-2\mkr10103\8\fid expt: <zgpg30>
transmitter freq.: 125.772879 MHz
time domain size: 65536 points
width: 36057.69 Hz = 286.6889 ppm = 0.550197 Hz/pt
number of scans: 512

freq. of 0 ppm: 125.757842 MHz
processed size: 32768 complex points
LB: 2.000 GF: 0.0000
Hz/cm: 997.742 ppm/cm: 7.93288

Compound **2l**

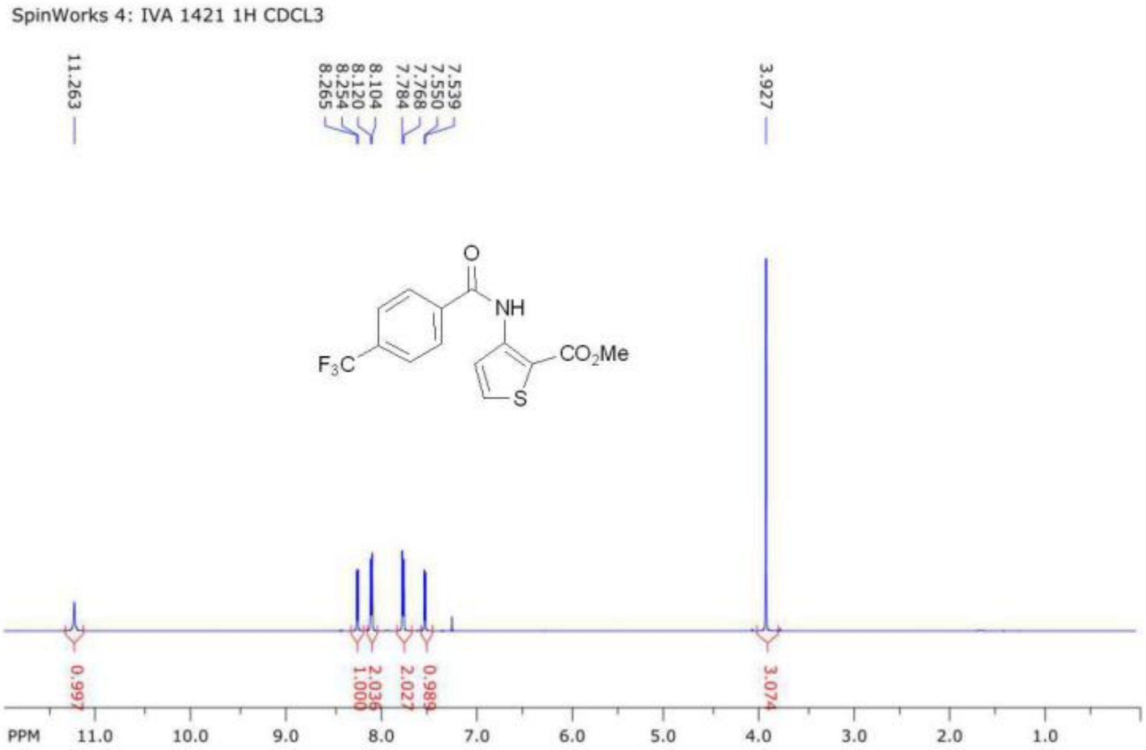

file: D:\NAPO\NMR\500-2\mkr10701\21\fid expt: <zg30>
transmitter freq.: 500.133001 MHz
time domain size: 65536 points
width: 12335.53 Hz = 24.6645 ppm = 0.188225 Hz/pt
number of scans: 24

freq. of 0 ppm: 500.130025 MHz
processed size: 65536 complex points
LB: 0.300 GF: 0.0000
Hz/cm: 240.161 ppm/cm: 0.48019

Compound **2l**

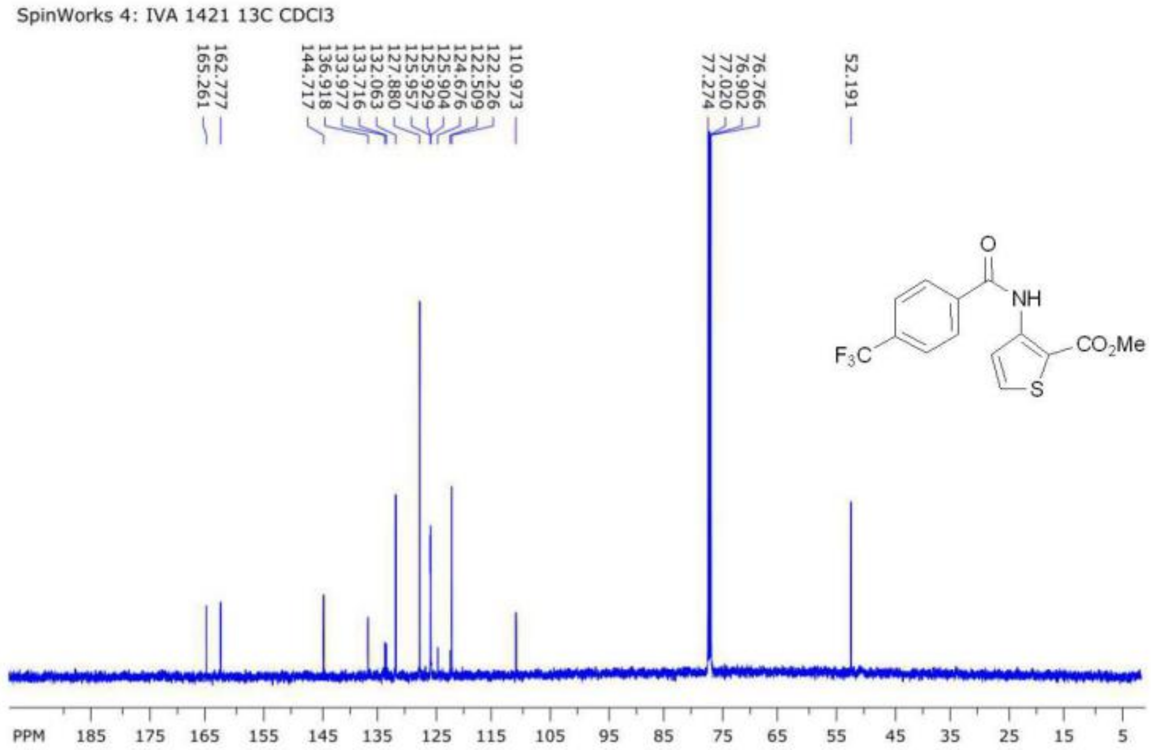

file: D:\NAPO\NMR\500-2\mkr10701\22\fid expt: <zgpg30>
transmitter freq.: 125.772879 MHz
time domain size: 65536 points
width: 36057.69 Hz = 286.6889 ppm = 0.550197 Hz/pt
number of scans: 512

freq. of 0 ppm: 125.757793 MHz
processed size: 32768 complex points
LB: 2.000 GF: 0.0000
Hz/cm: 998.766 ppm/cm: 7.94103

Compound **2m**

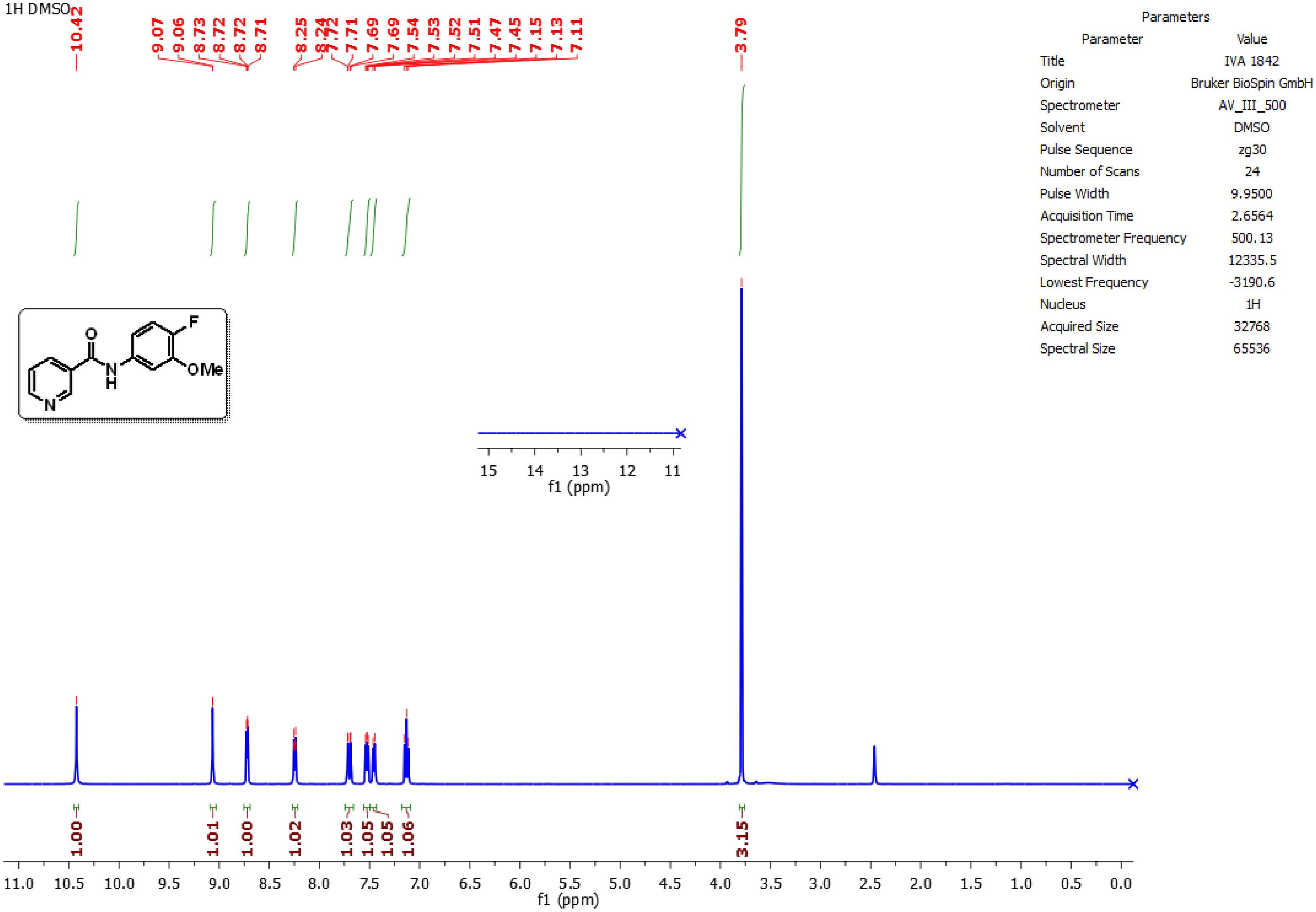

Parameter	Value
Title	IVA 1842
Origin	Bruker BioSpin GmbH
Spectrometer	AV_III_500
Solvent	DMSO
Pulse Sequence	zg30
Number of Scans	24
Pulse Width	9.9500
Acquisition Time	2.6564
Spectrometer Frequency	500.13
Spectral Width	12335.5
Lowest Frequency	-3190.6
Nucleus	1H
Acquired Size	32768
Spectral Size	65536

Compound **2m**

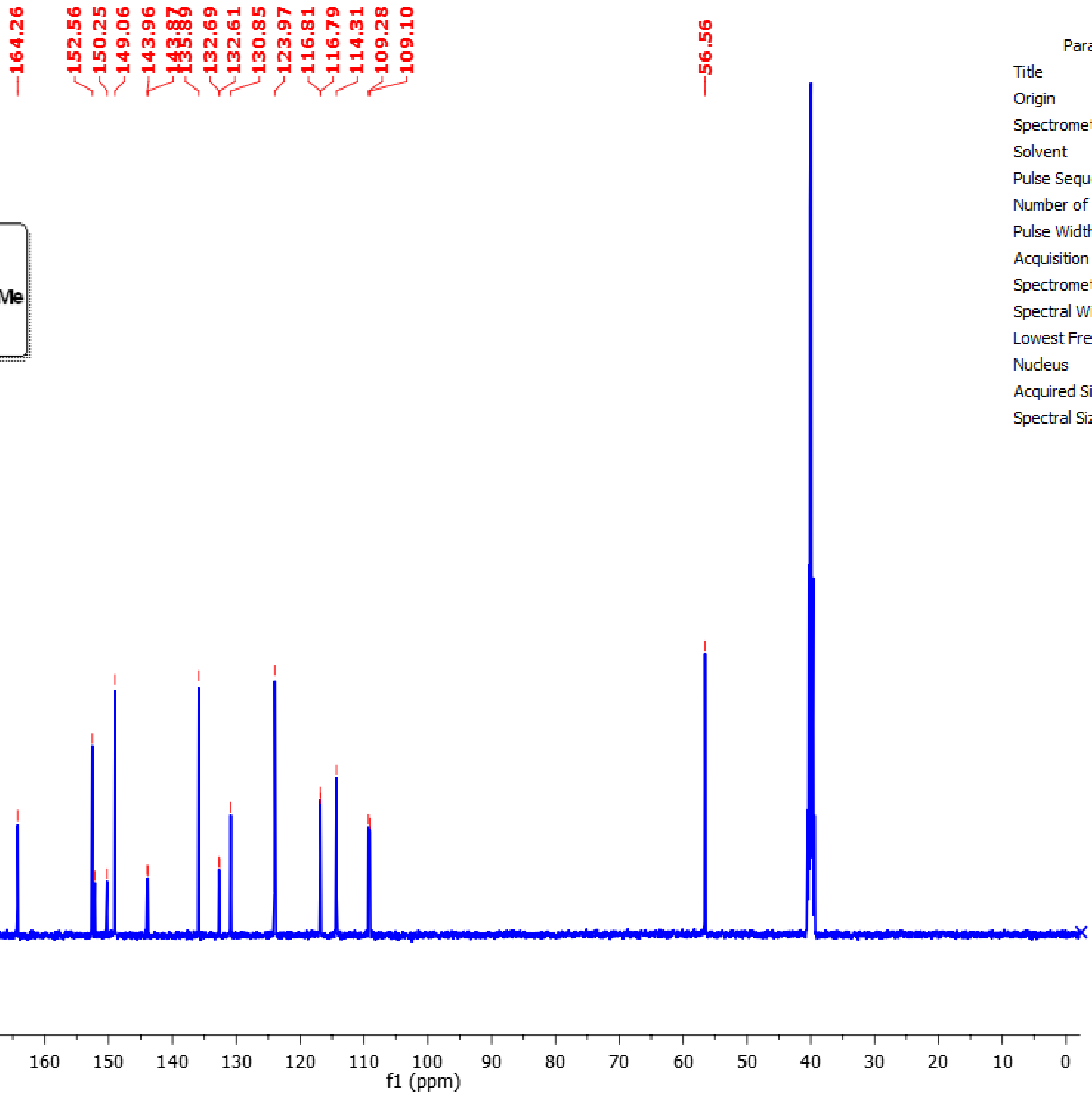

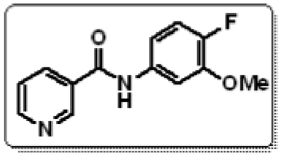

Parameters

Parameter	Value
Title	IVA 1842
Origin	Bruker BioSpin GmbH
Spectrometer	AV_III_500
Solvent	DMSO
Pulse Sequence	zgpg30
Number of Scans	512
Pulse Width	11.0000
Acquisition Time	0.9088
Spectrometer Frequency	125.76
Spectral Width	36057.7
Lowest Frequency	-2939.0
Nucleus	13C
Acquired Size	32768
Spectral Size	65536

Compound **2n**

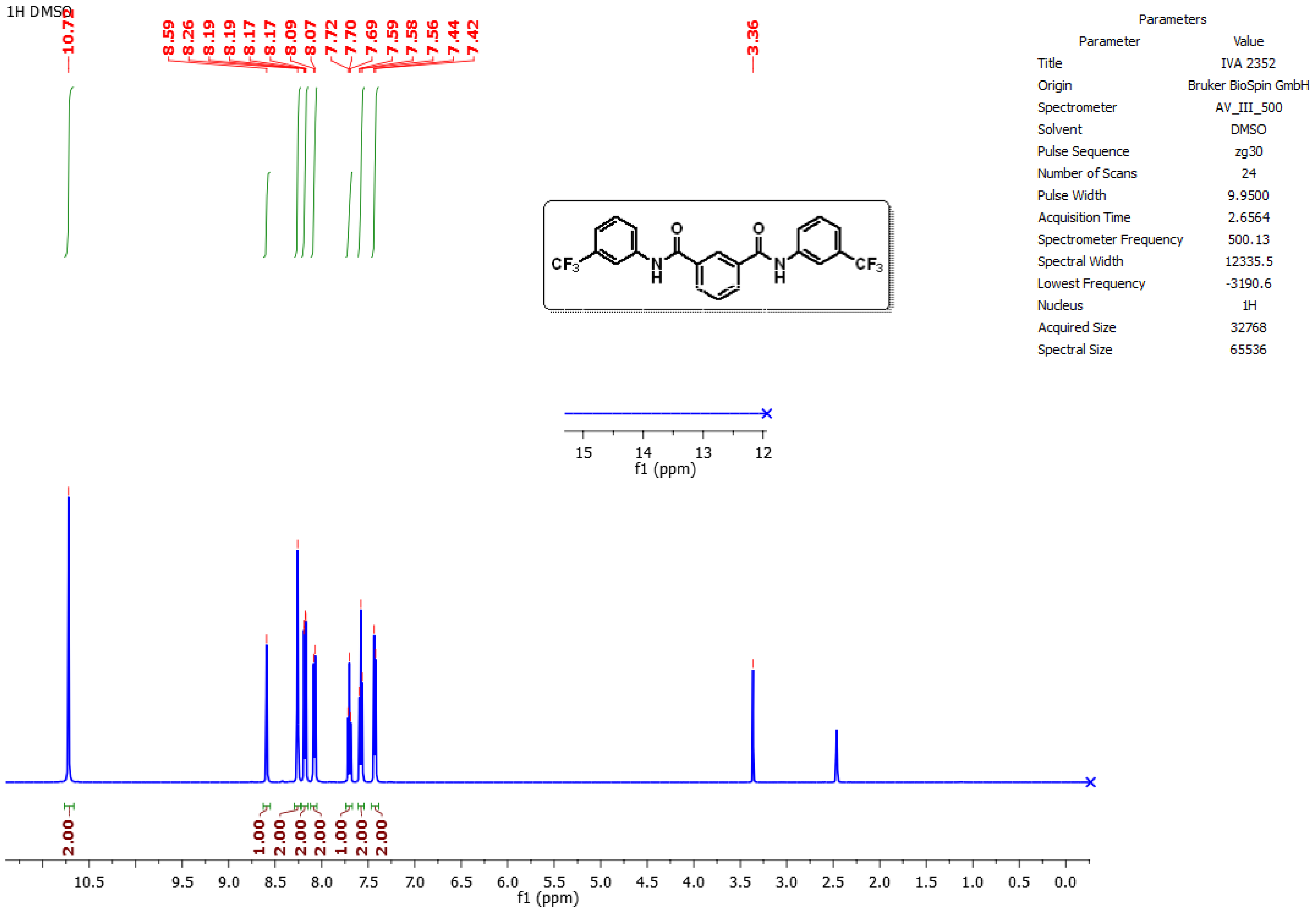

1H DMSO
10.72
8.59
8.26
8.19
8.19
8.17
8.17
8.09
8.07
7.72
7.70
7.69
7.59
7.58
7.56
7.44
7.42
3.36
CF3
N
H
O
O
N
H
CF3
Parameters
Parameter
Value
Title
IVA 2352
Origin
Bruker BioSpin GmbH
Spectrometer
AV_III_500
Solvent
DMSO
Pulse Sequence
zg30
Number of Scans
24
Pulse Width
9.9500
Acquisition Time
2.6564
Spectrometer Frequency
500.13
Spectral Width
12335.5
Lowest Frequency
-3190.6
Nucleus
1H
Acquired Size
32768
Spectral Size
65536
15
14
13
12
f1 (ppm)
2.00
1.00
2.00
2.00
2.00
1.00
2.00
2.00
10.5
9.5
9.0
8.5
8.0
7.5
7.0
6.5
6.0
5.5
5.0
4.5
4.0
3.5
3.0
2.5
2.0
1.5
1.0
0.5
0.0
f1 (ppm)

Compound **2n**

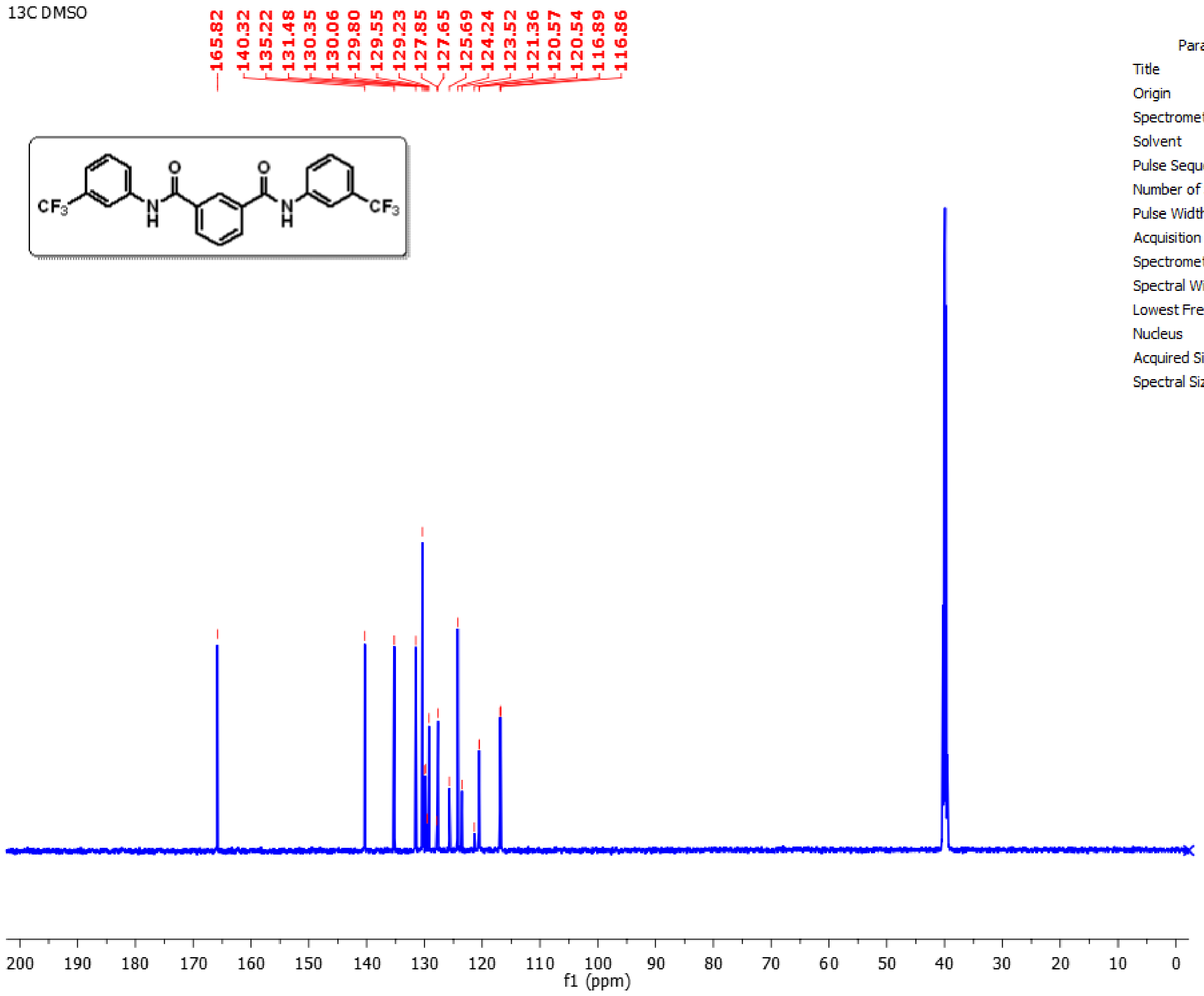

Parameters

Parameter	Value
Title	IVA 2352
Origin	Bruker BioSpin GmbH
Spectrometer	AV_III_500
Solvent	DMSO
Pulse Sequence	zgpg30
Number of Scans	512
Pulse Width	11.0000
Acquisition Time	0.9088
Spectrometer Frequency	125.76
Spectral Width	36057.7
Lowest Frequency	-2939.0
Nucleus	13C
Acquired Size	32768
Spectral Size	65536

Compound **2o**

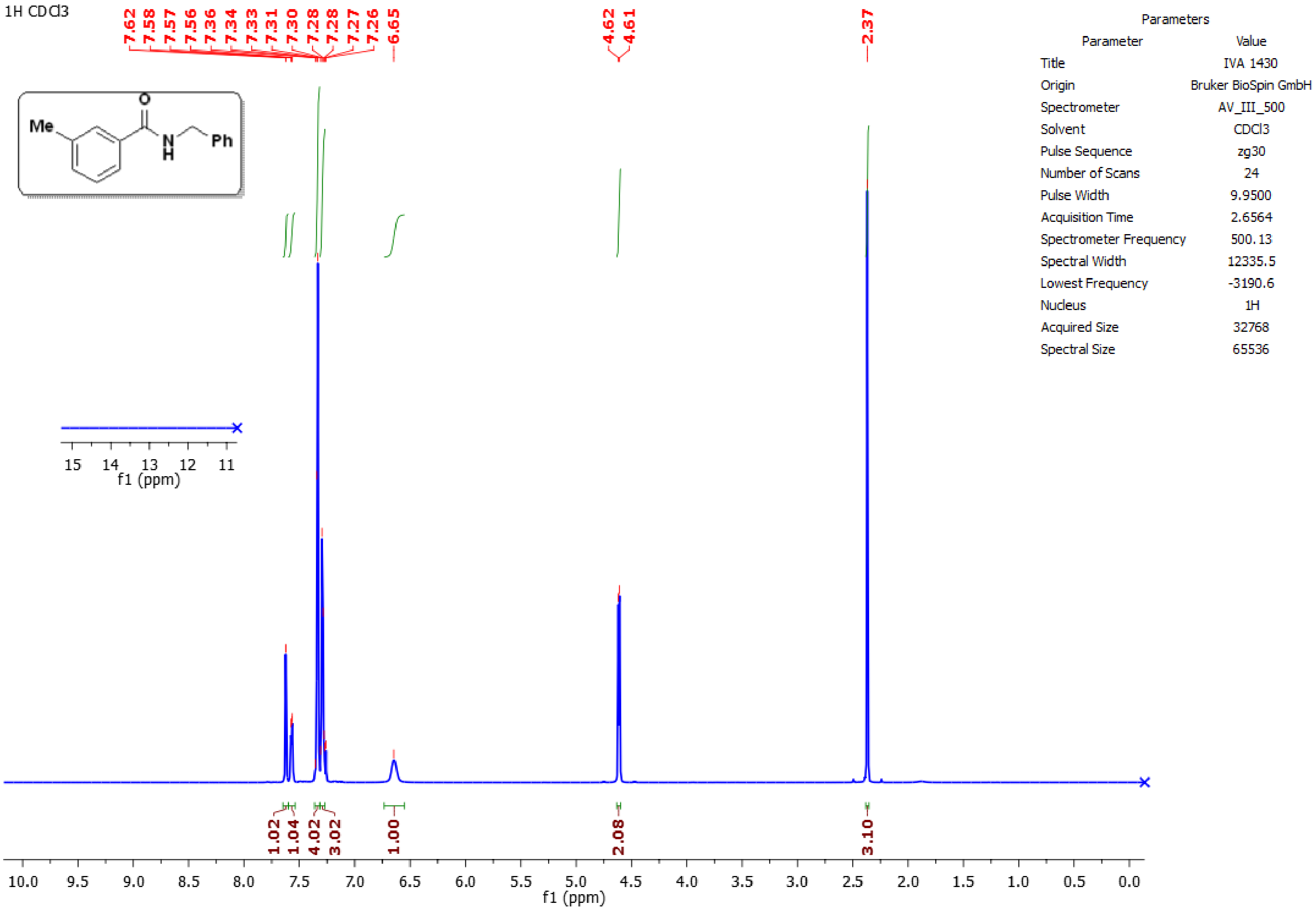
1H CDCl3
7.62
7.58
7.57
7.56
7.36
7.34
7.33
7.31
7.30
7.28
7.28
7.27
7.26
6.65
4.62
4.61
2.37
Me
O
N
H
Ph
15 14 13 12 11
f1 (ppm)
1.02
1.04
4.02
3.02
1.00
2.08
3.10
10.0 9.5 9.0 8.5 8.0 7.5 7.0 6.5 6.0 5.5 5.0 4.5 4.0 3.5 3.0 2.5 2.0 1.5 1.0 0.5 0.0
f1 (ppm)
Parameters
Parameter Value
Title IVA 1430
Origin Bruker BioSpin GmbH
Spectrometer AV_III_500
Solvent CDCl3
Pulse Sequence zg30
Number of Scans 24
Pulse Width 9.9500
Acquisition Time 2.6564
Spectrometer Frequency 500.13
Spectral Width 12335.5
Lowest Frequency -3190.6
Nucleus 1H
Acquired Size 32768
Spectral Size 65536

Compound **2o**

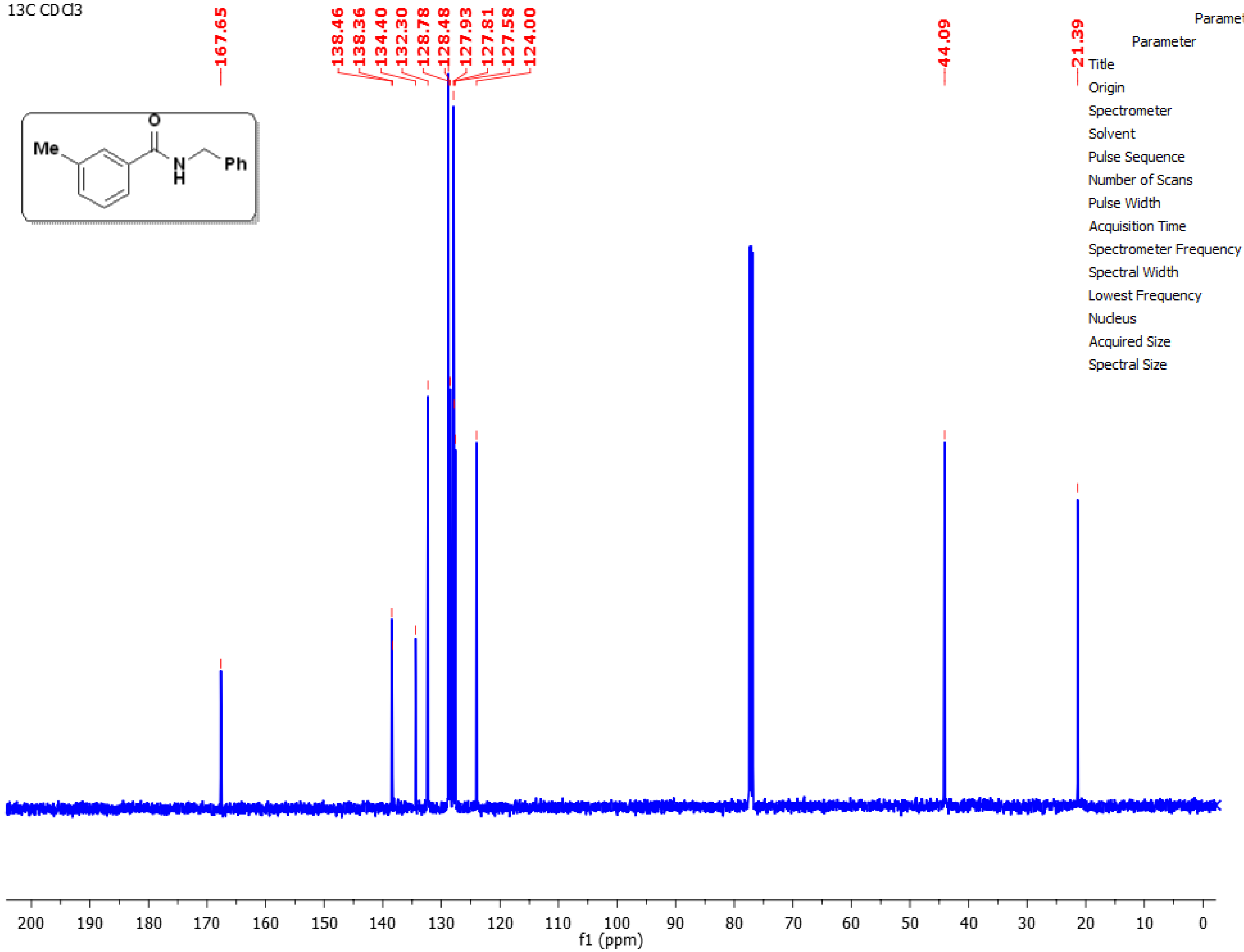

Parameters

Parameter	Value
Title	IVA 1430
Origin	Bruker BioSpin GmbH
Spectrometer	AV_III_500
Solvent	CDCl3
Pulse Sequence	zgpg30
Number of Scans	512
Pulse Width	11.0000
Acquisition Time	0.9088
Spectrometer Frequency	125.76
Spectral Width	36057.7
Lowest Frequency	-2939.0
Nucleus	13C
Acquired Size	32768
Spectral Size	65536

Compound **2p**

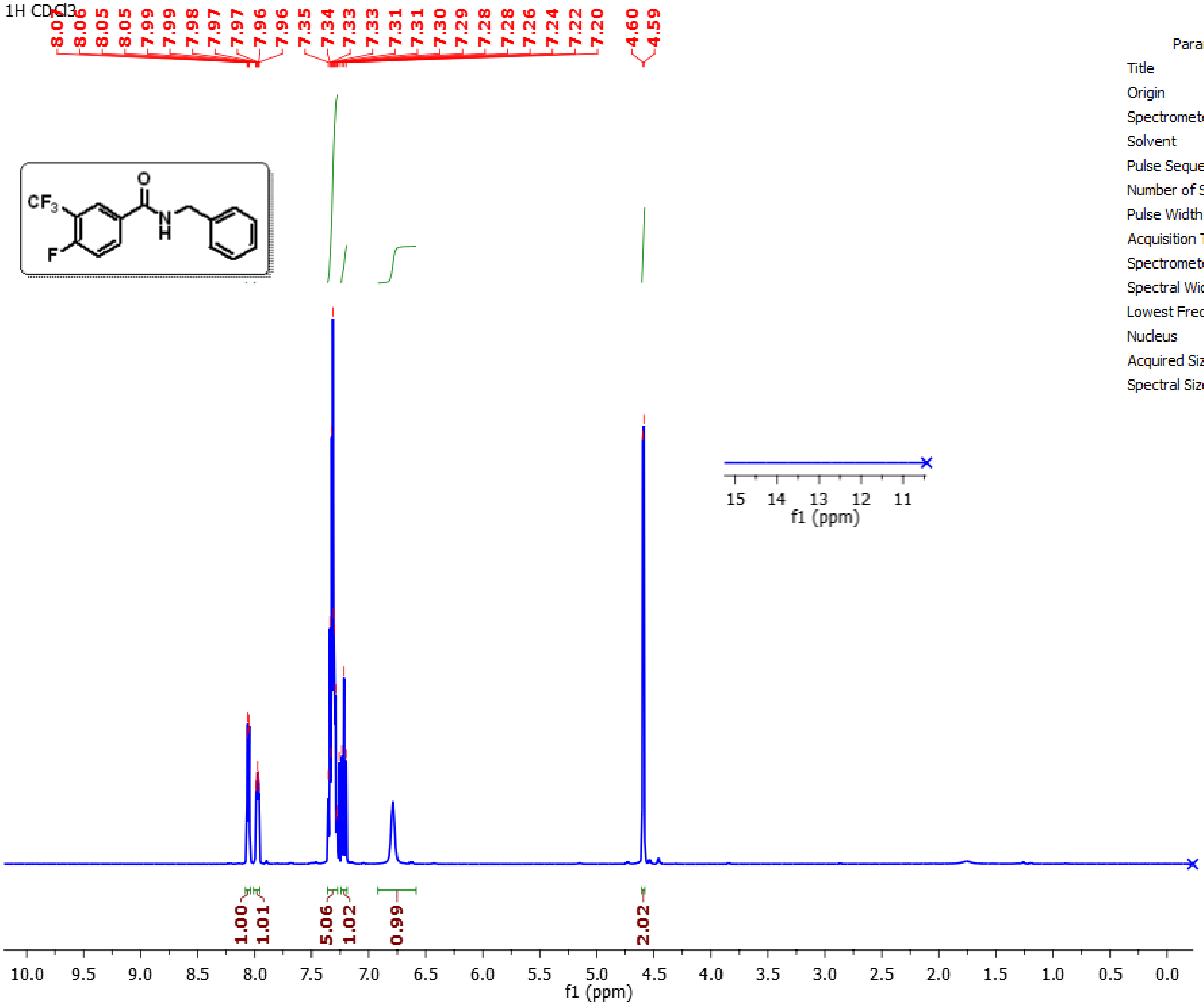

Parameters

Parameter	Value
Title	IVA 1754
Origin	Bruker BioSpin GmbH
Spectrometer	AV_III_500
Solvent	CDCl3
Pulse Sequence	zg30
Number of Scans	24
Pulse Width	9.6600
Acquisition Time	2.6564
Spectrometer Frequency	500.13
Spectral Width	12335.5
Lowest Frequency	-3190.6
Nucleus	1H
Acquired Size	32768
Spectral Size	65536

Compound **2p**

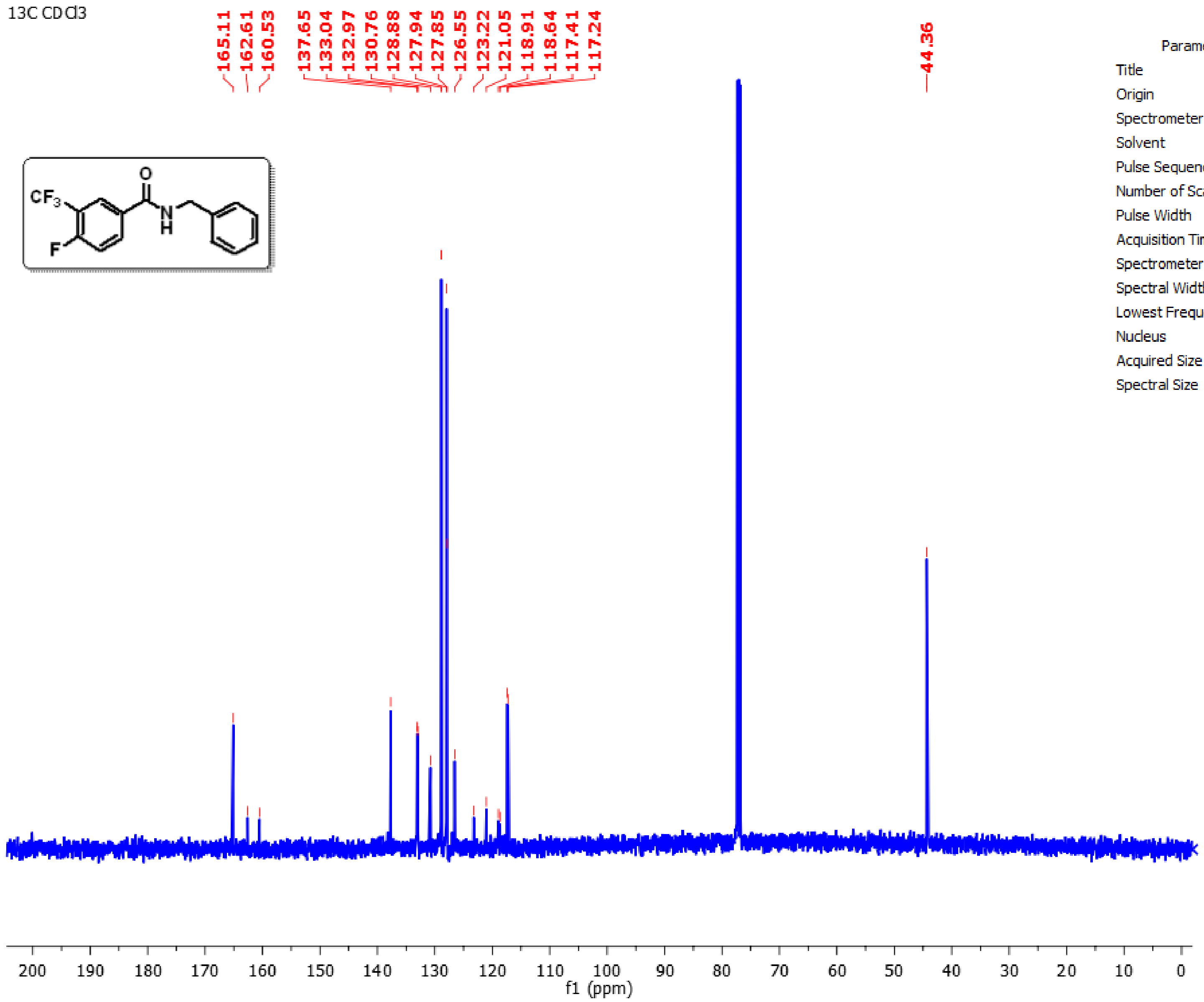

Parameter	Value
Title	IVA 1754
Origin	Bruker BioSpin GmbH
Spectrometer	AV_III_500
Solvent	CDCl3
Pulse Sequence	zgpg30
Number of Scans	512
Pulse Width	11.5000
Acquisition Time	0.9088
Spectrometer Frequency	125.76
Spectral Width	36057.7
Lowest Frequency	-2939.0
Nucleus	13C
Acquired Size	32768
Spectral Size	65536

Compound **2q**

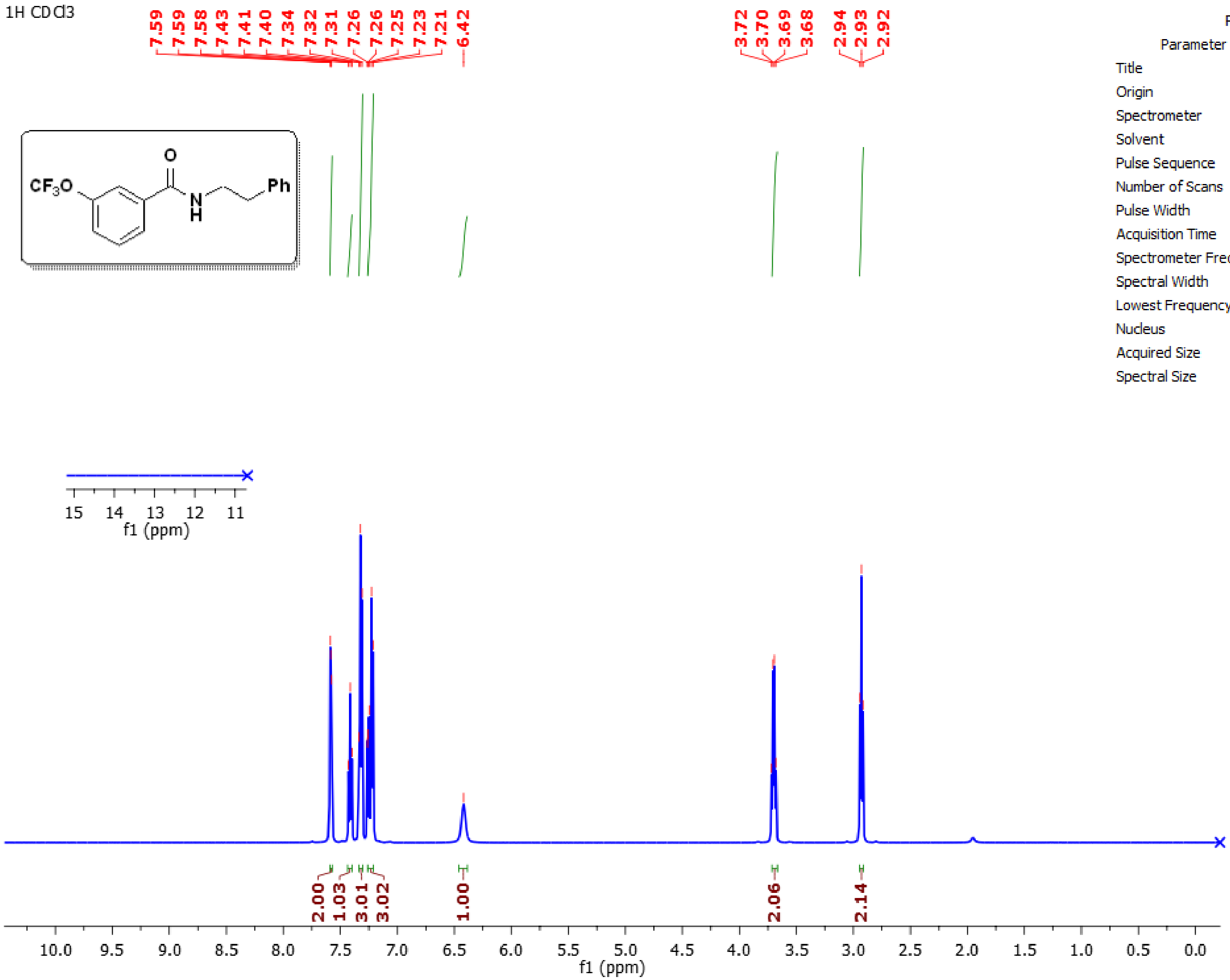

Parameters

Parameter	Value
Title	IVA 1427
Origin	Bruker BioSpin GmbH
Spectrometer	AV_III_500
Solvent	CDCl3
Pulse Sequence	zg30
Number of Scans	24
Pulse Width	9.9500
Acquisition Time	2.6564
Spectrometer Frequency	500.13
Spectral Width	12335.5
Lowest Frequency	-3190.6
Nucleus	1H
Acquired Size	32768
Spectral Size	65536

Compound **2q**

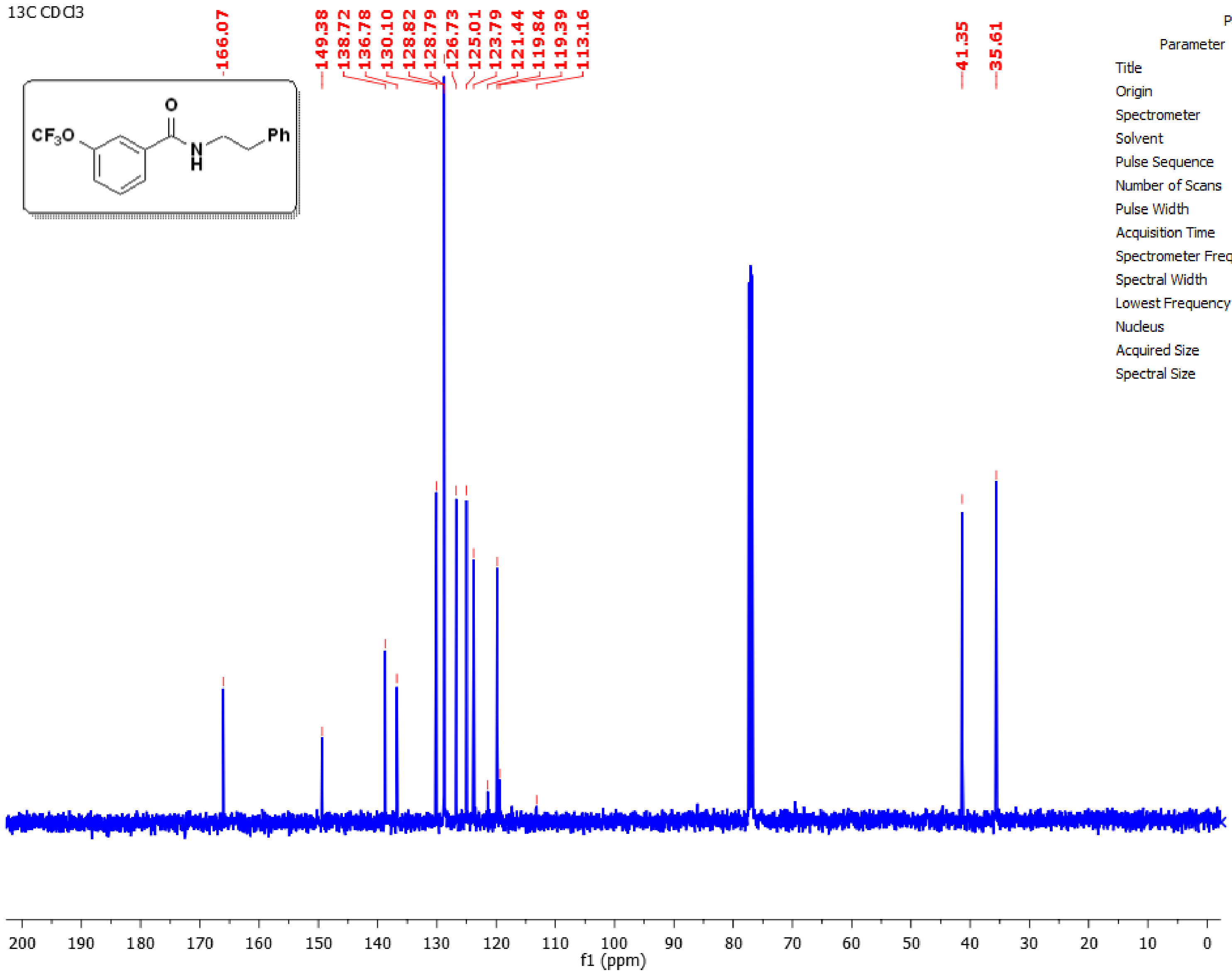

Parameters

Parameter	Value
Title	IVA 1427
Origin	Bruker BioSpin GmbH
Spectrometer	AV_III_500
Solvent	CDCl3
Pulse Sequence	zgpg30
Number of Scans	148
Pulse Width	11.0000
Acquisition Time	0.9088
Spectrometer Frequency	125.76
Spectral Width	36057.7
Lowest Frequency	-2939.0
Nucleus	13C
Acquired Size	32768
Spectral Size	65536

Compound **2r**

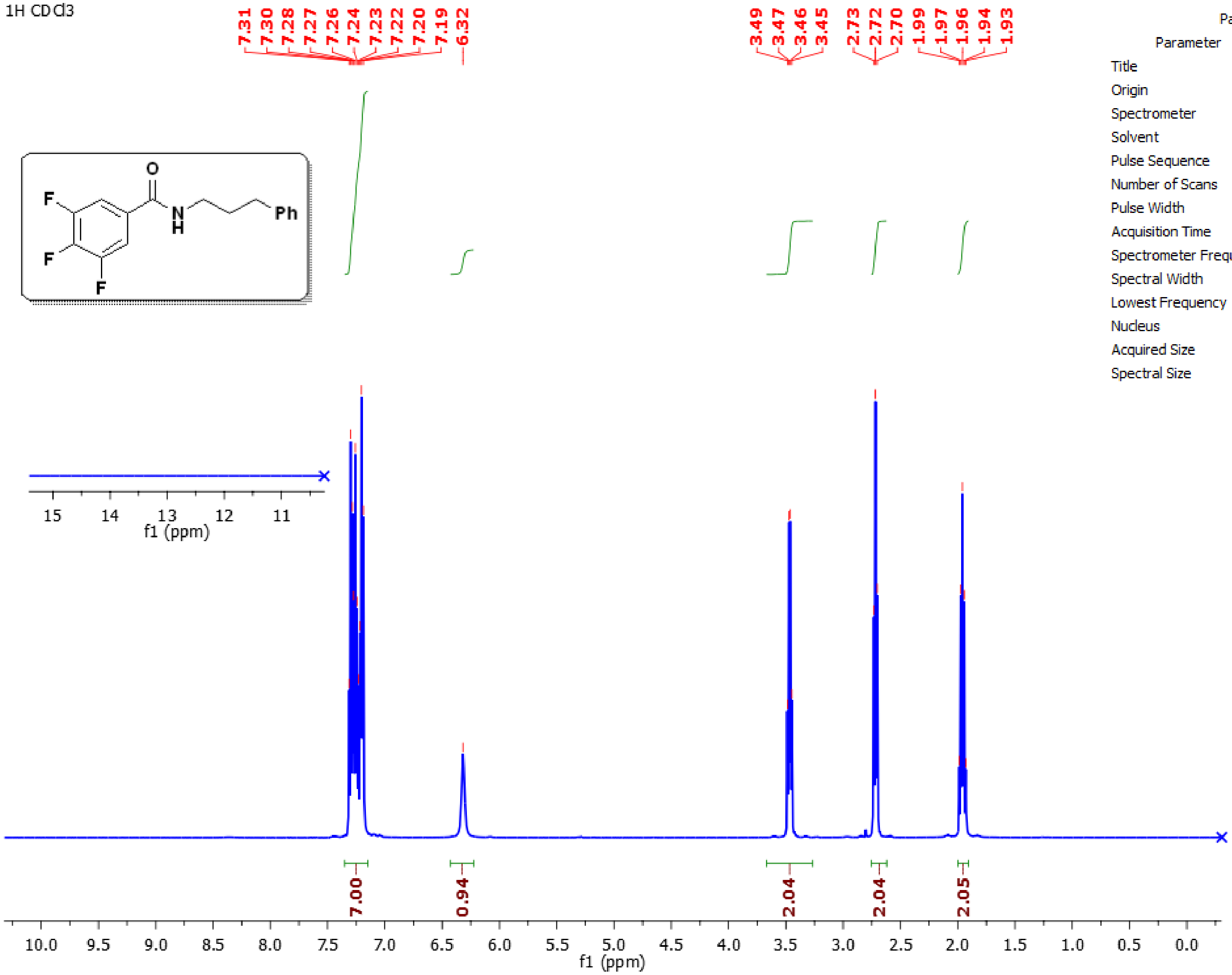

Parameters

Parameter	Value
Title	IVA 1450
Origin	Bruker BioSpin GmbH
Spectrometer	AV_III_500
Solvent	CDCl3
Pulse Sequence	zg30
Number of Scans	24
Pulse Width	9.9500
Acquisition Time	2.6564
Spectrometer Frequency	500.13
Spectral Width	12335.5
Lowest Frequency	-3190.6
Nucleus	1H
Acquired Size	32768
Spectral Size	65536

Compound **2r**

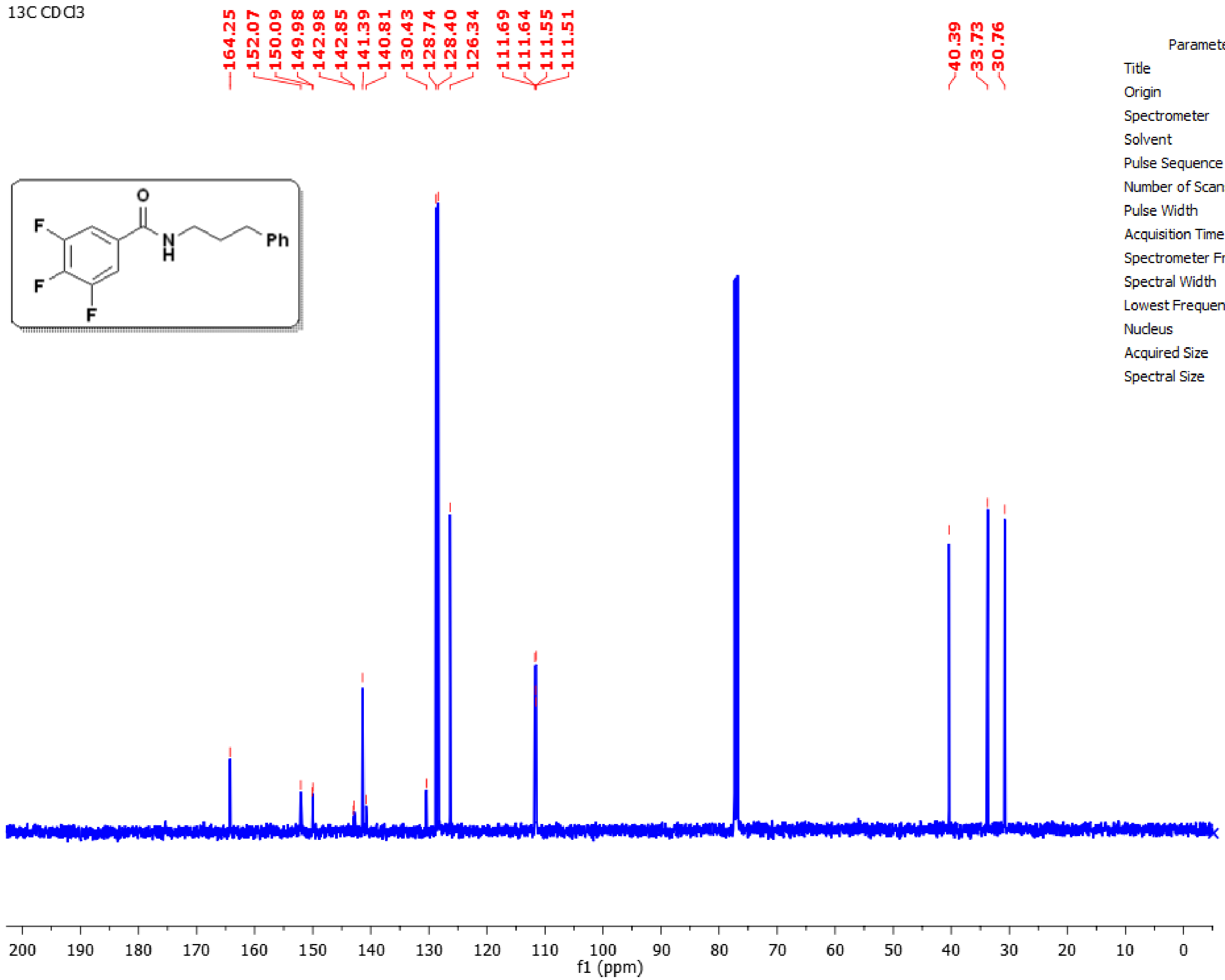

Parameters

Parameter	Value
Title	IVA 1450
Origin	Bruker BioSpin GmbH
Spectrometer	AV_III_500
Solvent	CDCl3
Pulse Sequence	zgpg30
Number of Scans	256
Pulse Width	11.0000
Acquisition Time	0.9088
Spectrometer Frequency	125.76
Spectral Width	36057.7
Lowest Frequency	-2939.0
Nucleus	13C
Acquired Size	32768
Spectral Size	65536

Compound **2s**

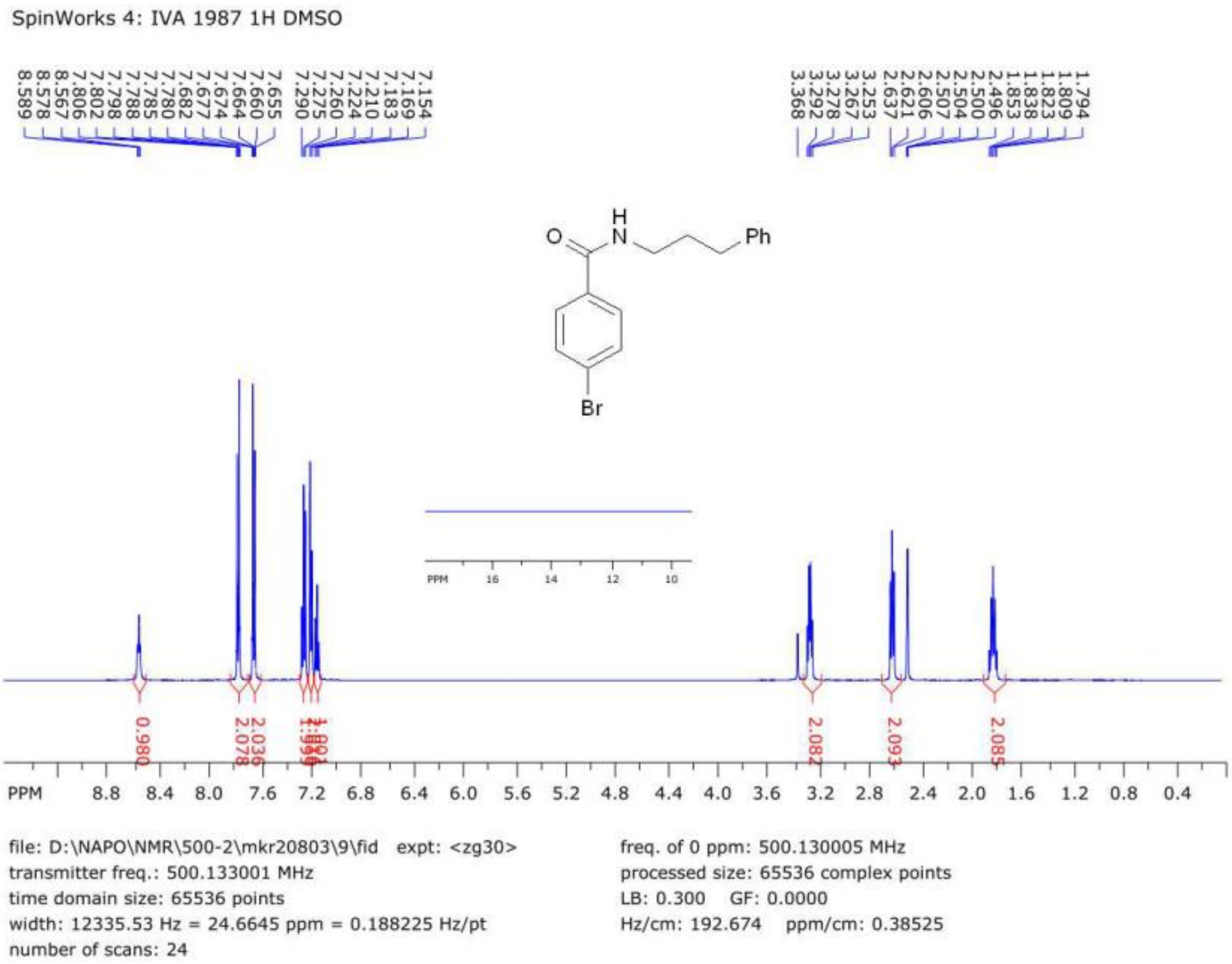
SpinWorks 4: IVA 1987 1H DMSO
8.589
8.578
8.567
7.806
7.802
7.798
7.788
7.785
7.780
7.682
7.677
7.674
7.664
7.660
7.655
7.290
7.275
7.260
7.224
7.210
7.183
7.169
7.154
3.368
3.292
3.278
3.267
3.253
2.637
2.621
2.606
2.507
2.504
2.500
2.496
1.853
1.838
1.823
1.809
1.794
O
H
N
Ph
Br
PPM 16 14 12 10
0.980
2.078
2.036
2.082
2.093
2.085
PPM 8.8 8.4 8.0 7.6 7.2 6.8 6.4 6.0 5.6 5.2 4.8 4.4 4.0 3.6 3.2 2.8 2.4 2.0 1.6 1.2 0.8 0.4
file: D:\NAPO\NMR\500-2\mkr20803\9\fid expt: <zg30>
transmitter freq.: 500.133001 MHz
time domain size: 65536 points
width: 12335.53 Hz = 24.6645 ppm = 0.188225 Hz/pt
number of scans: 24
freq. of 0 ppm: 500.130005 MHz
processed size: 65536 complex points
LB: 0.300 GF: 0.0000
Hz/cm: 192.674 ppm/cm: 0.38525

Compound **2s**

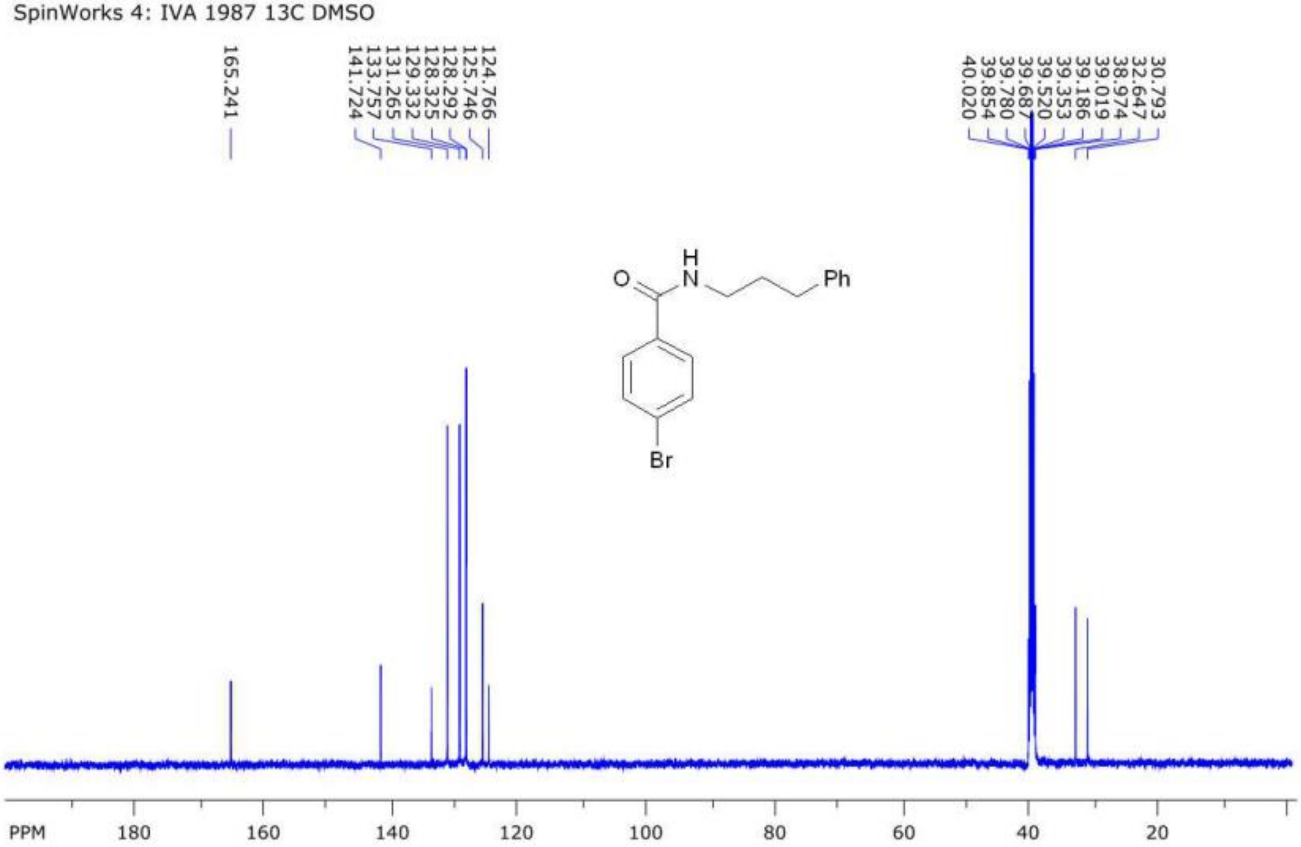

file: D:\NAPO\NMR\500-2\mkr20803\10\fid expt: <zgpg30>
transmitter freq.: 125.772879 MHz
time domain size: 65536 points
width: 36057.69 Hz = 286.6889 ppm = 0.550197 Hz/pt
number of scans: 512

freq. of 0 ppm: 125.757845 MHz
processed size: 32768 complex points
LB: 2.000 GF: 0.0000
Hz/cm: 1016.316 ppm/cm: 8.08057

Compound **2t**

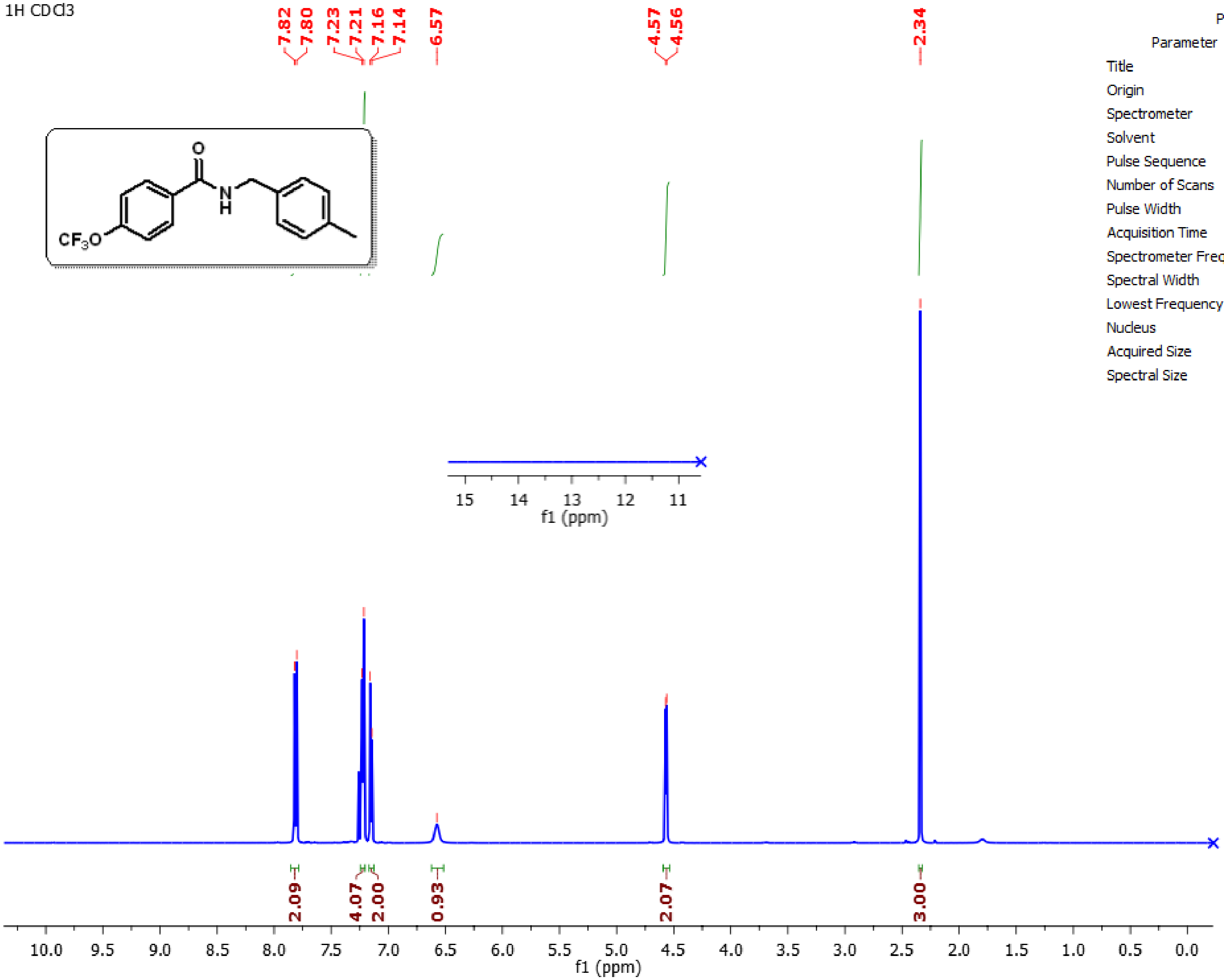

Parameters

Parameter	Value
Title	IVA 1382
Origin	Bruker BioSpin GmbH
Spectrometer	AV_III_500
Solvent	CDCl3
Pulse Sequence	zg30
Number of Scans	24
Pulse Width	9.9500
Acquisition Time	2.6564
Spectrometer Frequency	500.13
Spectral Width	12335.5
Lowest Frequency	-3190.6
Nucleus	1H
Acquired Size	32768
Spectral Size	65536

Compound **2t**

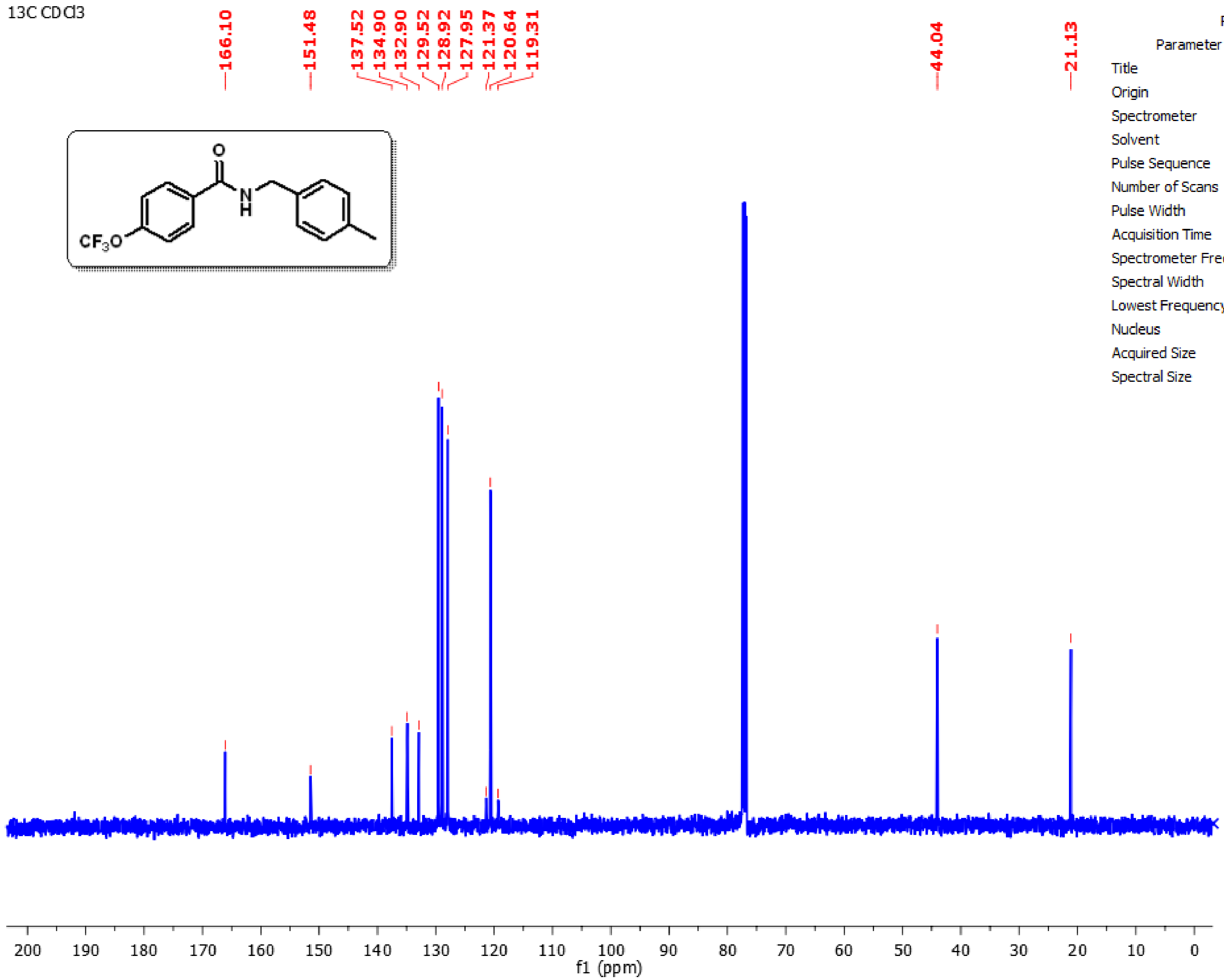

Parameters

Parameter	Value
Title	IVA 1382
Origin	Bruker BioSpin GmbH
Spectrometer	AV_III_500
Solvent	CDCl3
Pulse Sequence	zgpg30
Number of Scans	256
Pulse Width	11.0000
Acquisition Time	0.9088
Spectrometer Frequency	125.76
Spectral Width	36057.7
Lowest Frequency	-2939.0
Nucleus	13C
Acquired Size	32768
Spectral Size	65536

Compound **2u**

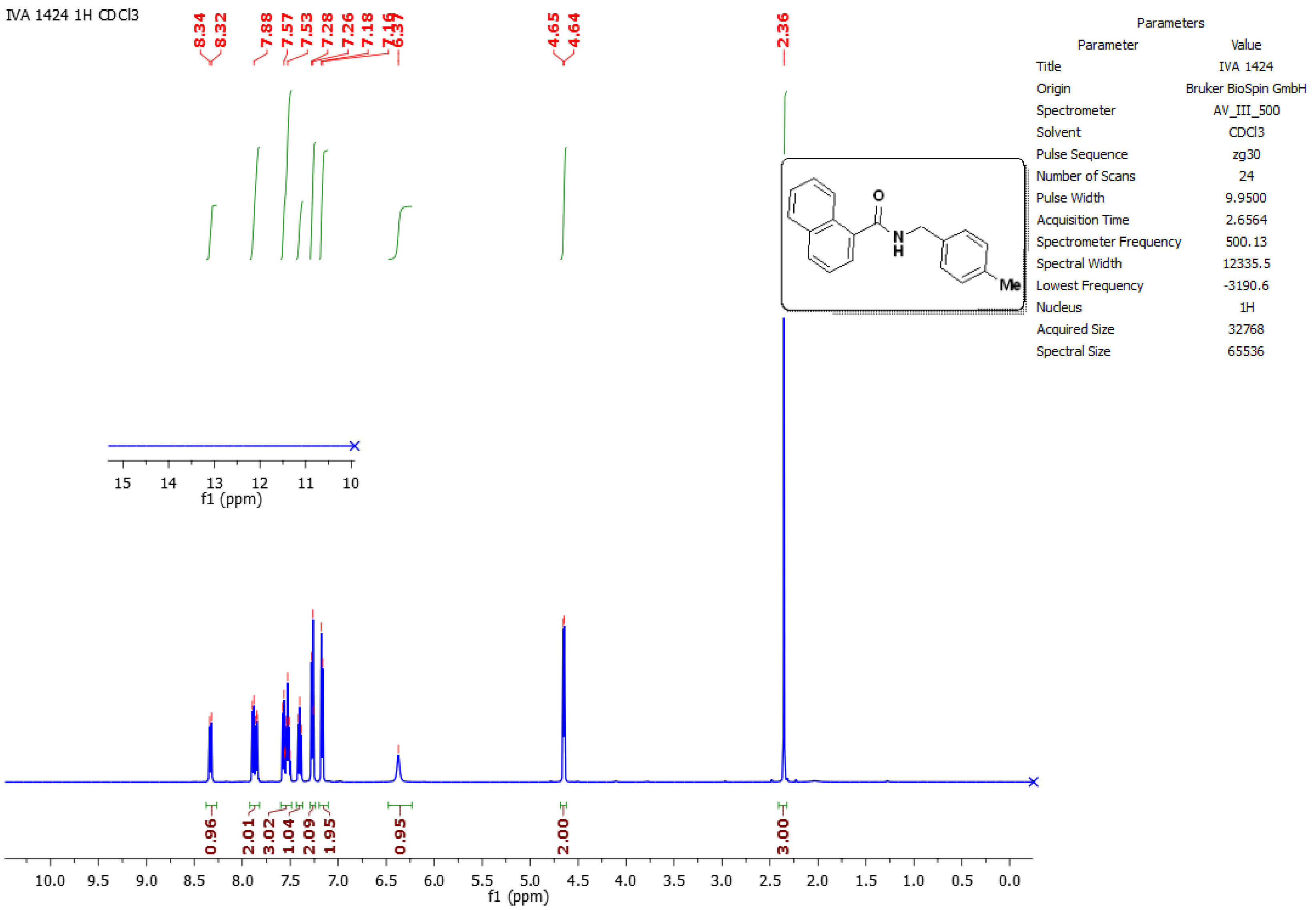
IVA 1424 1H CDCl3
8.34
8.32
7.88
7.57
7.53
7.28
7.26
7.18
7.16
6.37
4.65
4.64
2.36
O
N
H
Me
Parameters
Parameter Value
Title IVA 1424
Origin Bruker BioSpin GmbH
Spectrometer AV_III_500
Solvent CDCl3
Pulse Sequence zg30
Number of Scans 24
Pulse Width 9.9500
Acquisition Time 2.6564
Spectrometer Frequency 500.13
Spectral Width 12335.5
Lowest Frequency -3190.6
Nucleus 1H
Acquired Size 32768
Spectral Size 65536
15 14 13 12 11 10
f1 (ppm)
0.96
2.01
3.02
1.04
2.09
1.95
0.95
2.00
3.00
10.0 9.5 9.0 8.5 8.0 7.5 7.0 6.5 6.0 5.5 5.0 4.5 4.0 3.5 3.0 2.5 2.0 1.5 1.0 0.5 0.0
f1 (ppm)

Compound **2u**

SpinWorks 4: IVA 1424 13C CDCl3

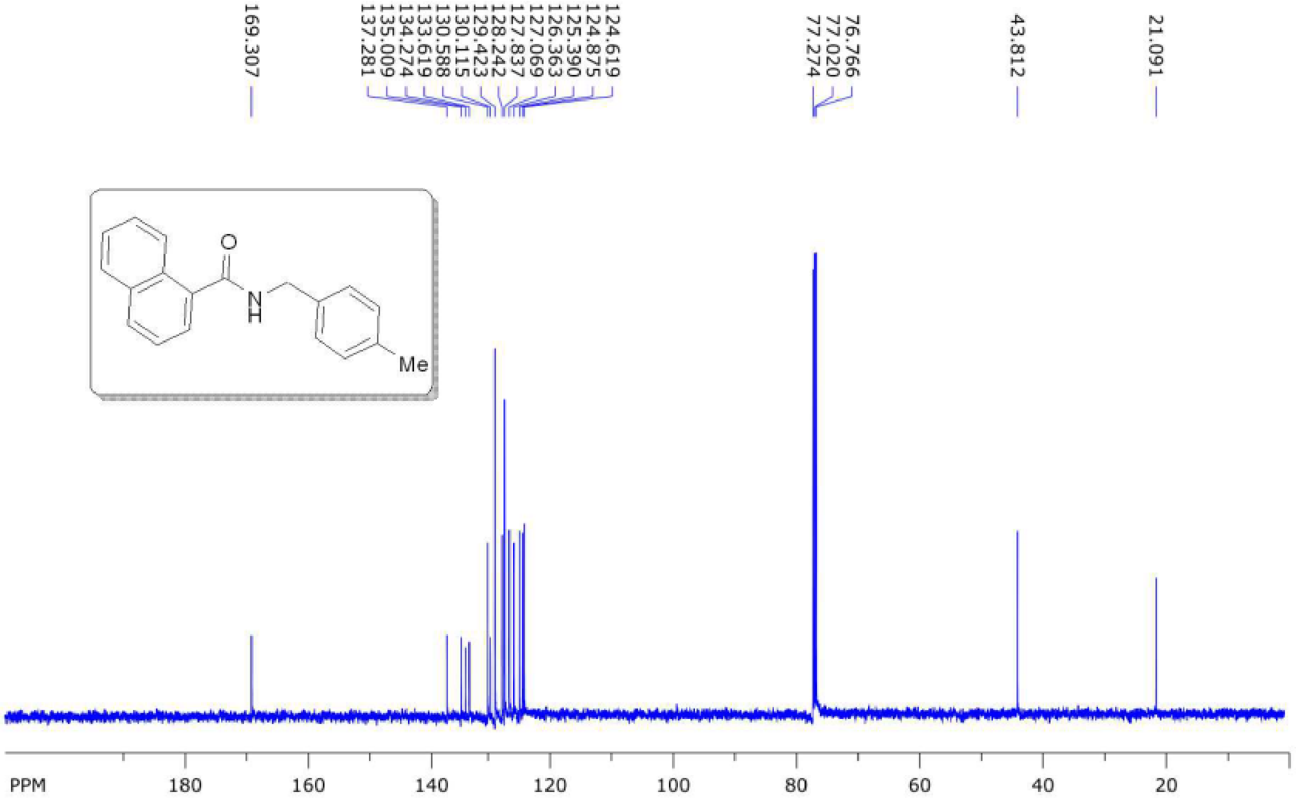

file: D:\NAPO\NMR\500-2\mkr11712\30\fid expt: <zgpg30>
transmitter freq.: 125.772879 MHz
time domain size: 65536 points
width: 36057.69 Hz = 286.6889 ppm = 0.550197 Hz/pt
number of scans: 256

freq. of 0 ppm: 125.757799 MHz
processed size: 32768 complex points
LB: 2.000 GF: 0.0000
Hz/cm: 1054.608 ppm/cm: 8.38502

Compound **2v**

SpinWorks 4: IVA 1987 1H CDCl3

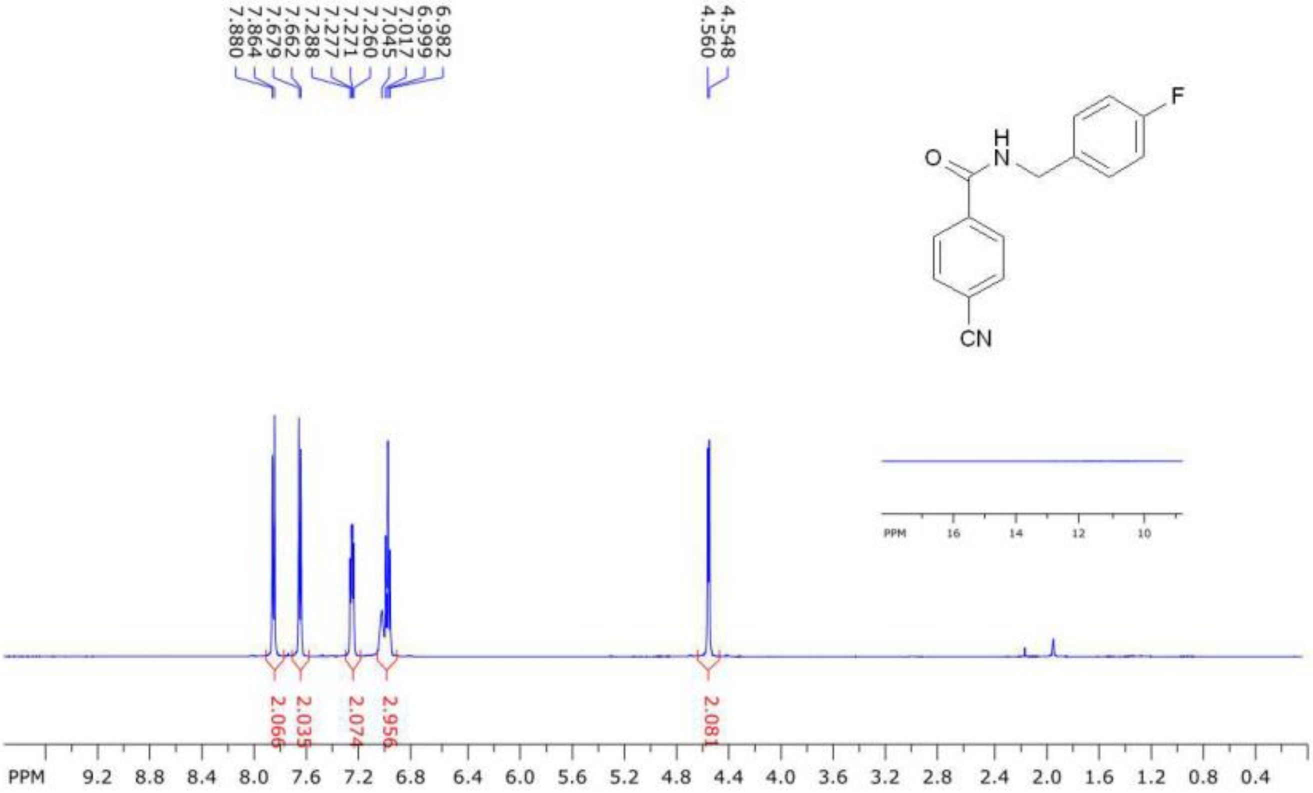

file: D:\NAPO\NMR\500-2\mkr23004\13\fid expt: <zg30>
transmitter freq.: 500.133001 MHz
time domain size: 65536 points
width: 12335.53 Hz = 24.6645 ppm = 0.188225 Hz/pt
number of scans: 24

freq. of 0 ppm: 500.130014 MHz
processed size: 65536 complex points
LB: 0.300 GF: 0.0000
Hz/cm: 198.133 ppm/cm: 0.39616

Compound **2v**

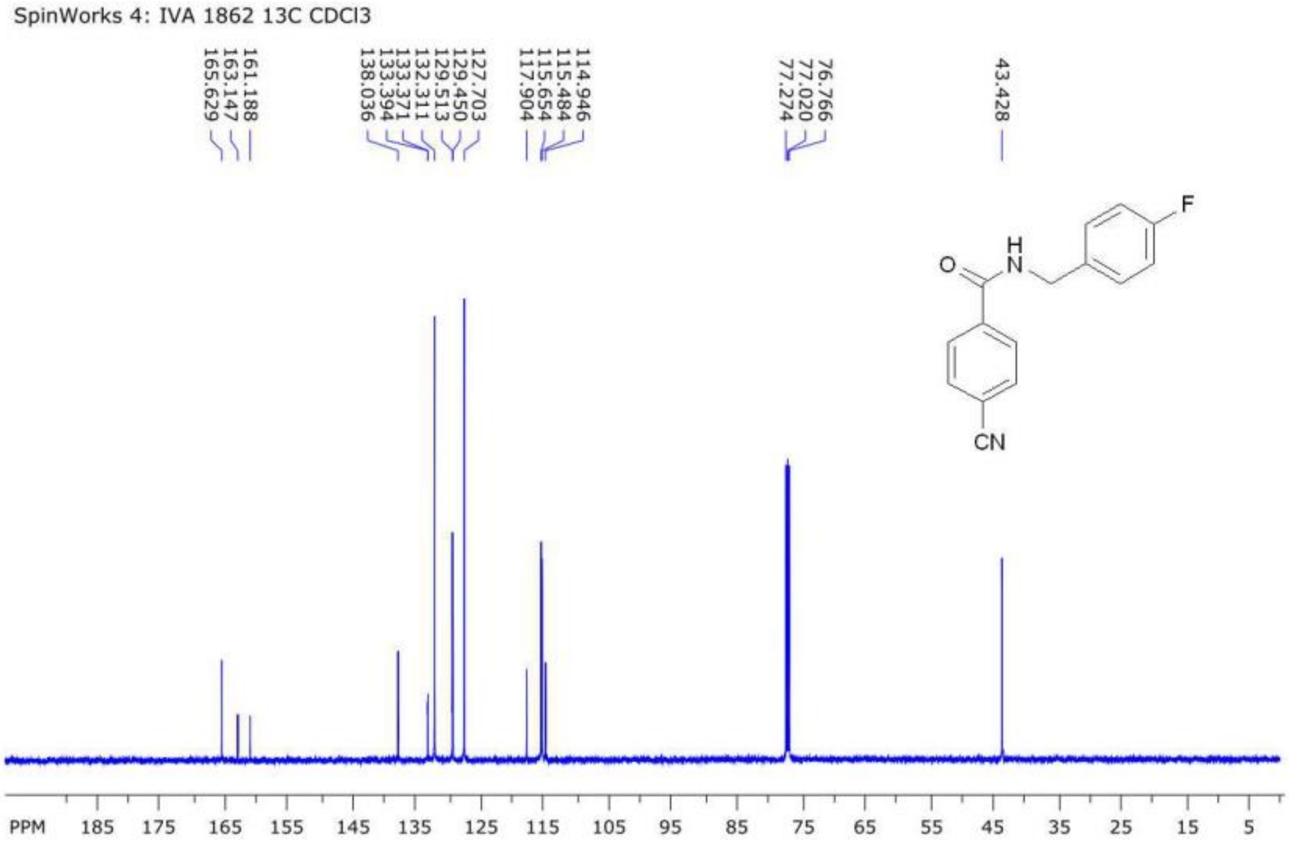

file: D:\NAPO\NMR\500-2\mkr23004\14\fid expt: <zgpg30>
transmitter freq.: 125.772879 MHz
time domain size: 65536 points
width: 36057.69 Hz = 286.6889 ppm = 0.550197 Hz/pt
number of scans: 512

freq. of 0 ppm: 125.757802 MHz
processed size: 32768 complex points
LB: 2.000 GF: 0.0000
Hz/cm: 1004.298 ppm/cm: 7.98501

Compound **2w**

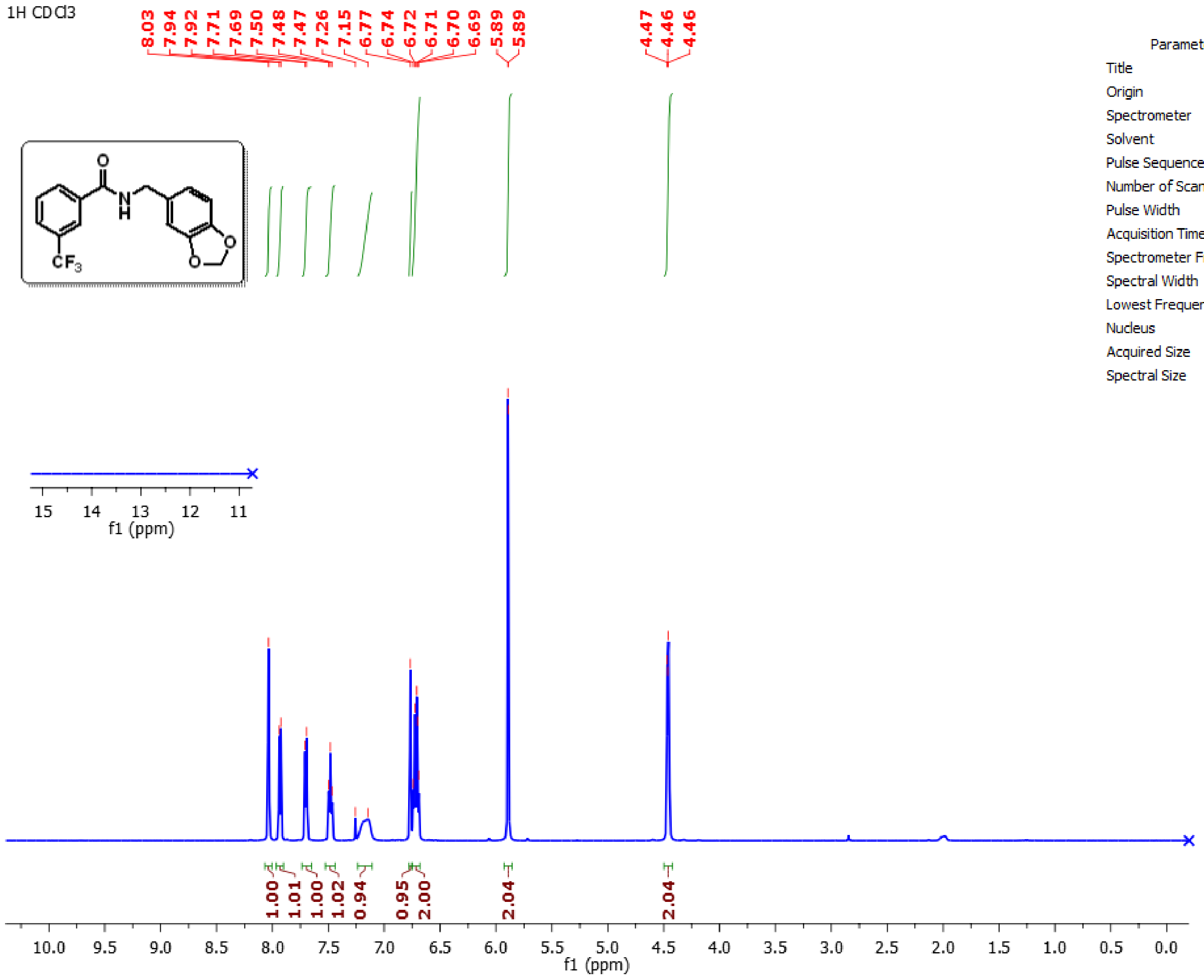

Parameters

Parameter	Value
Title	IVA 1858
Origin	Bruker BioSpin GmbH
Spectrometer	AV_III_500
Solvent	CDCl3
Pulse Sequence	zg30
Number of Scans	24
Pulse Width	9.9500
Acquisition Time	2.6564
Spectrometer Frequency	500.13
Spectral Width	12335.5
Lowest Frequency	-3190.6
Nucleus	1H
Acquired Size	32768
Spectral Size	65536

Compound **2w**

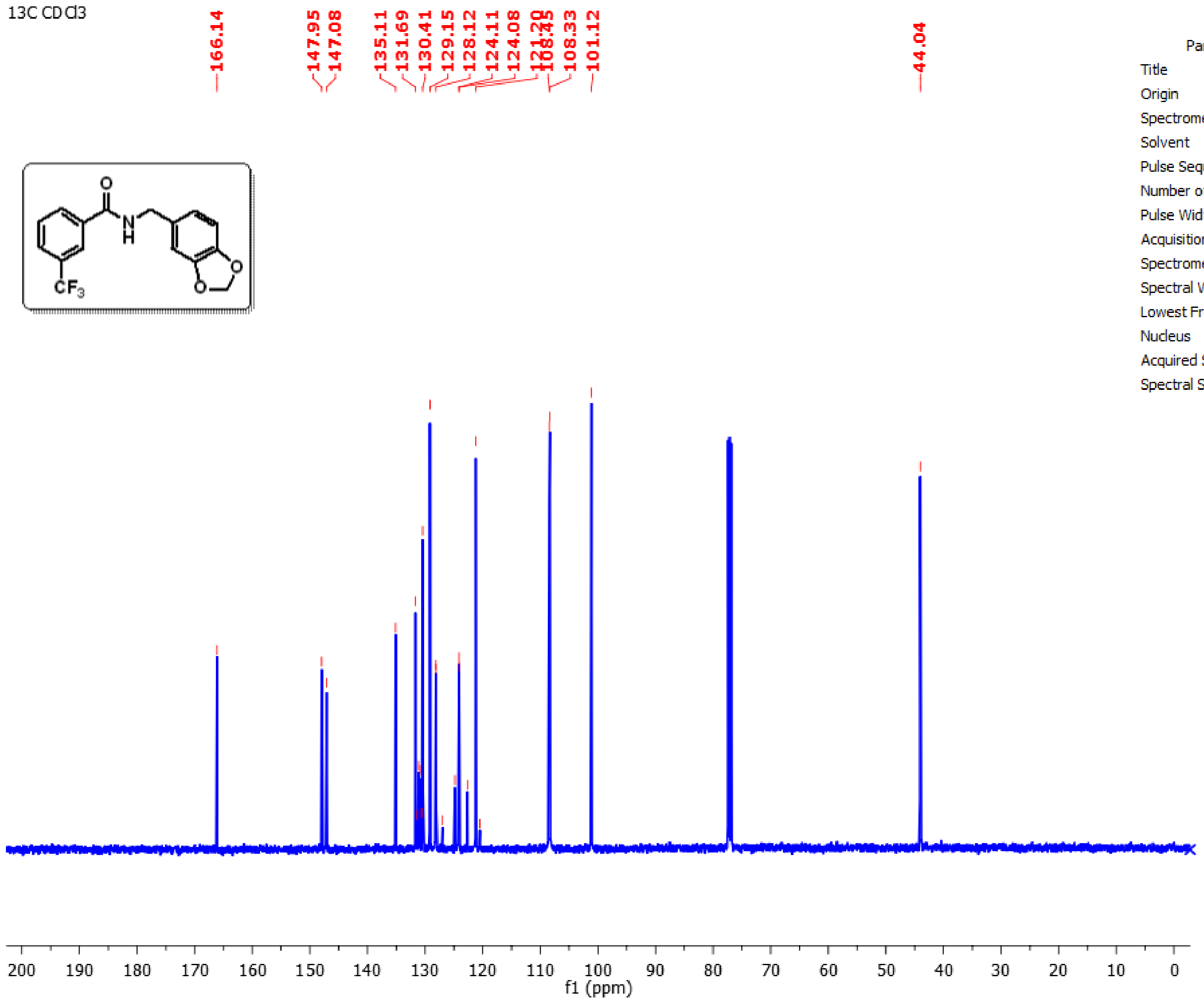

Parameters

Parameter	Value
Title	IVA 1858
Origin	Bruker BioSpin GmbH
Spectrometer	AV_III_500
Solvent	CDCl3
Pulse Sequence	zgpg30
Number of Scans	512
Pulse Width	11.0000
Acquisition Time	0.9088
Spectrometer Frequency	125.76
Spectral Width	36057.7
Lowest Frequency	-2939.0
Nucleus	13C
Acquired Size	32768
Spectral Size	65536

Compound **2x**

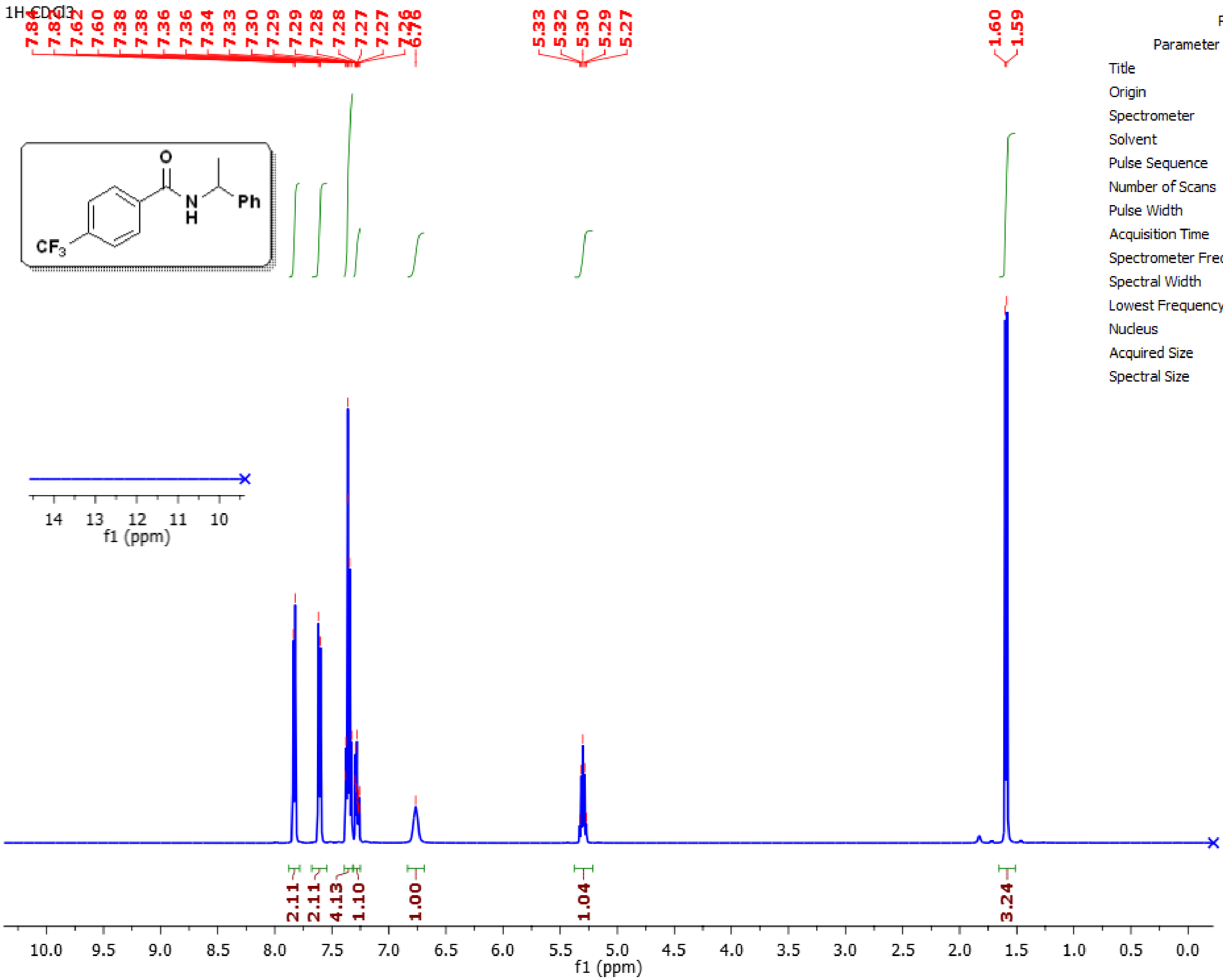

Parameters

Parameter	Value
Title	IVA 2336
Origin	Bruker BioSpin GmbH
Spectrometer	AV_III_500
Solvent	CDCl3
Pulse Sequence	zg30
Number of Scans	24
Pulse Width	9.9500
Acquisition Time	2.6564
Spectrometer Frequency	500.13
Spectral Width	12335.5
Lowest Frequency	-3190.6
Nucleus	1H
Acquired Size	32768
Spectral Size	65536

Compound **2x**

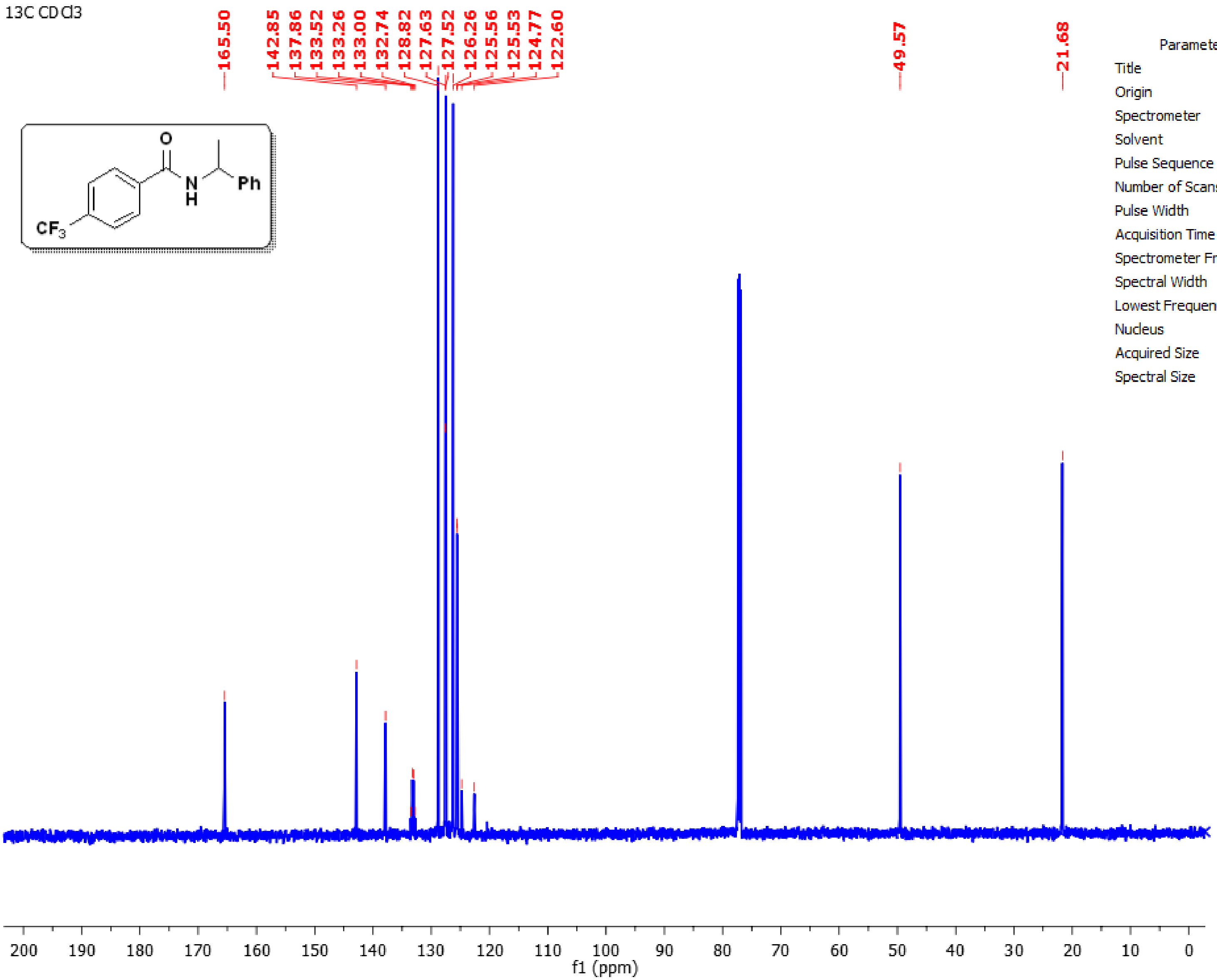

Parameters

Parameter	Value
Title	IVA 2336
Origin	Bruker BioSpin GmbH
Spectrometer	AV_III_500
Solvent	CDCl3
Pulse Sequence	zgpg30
Number of Scans	512
Pulse Width	11.0000
Acquisition Time	0.9088
Spectrometer Frequency	125.76
Spectral Width	36057.7
Lowest Frequency	-2939.0
Nucleus	13C
Acquired Size	32768
Spectral Size	65536

Compound **2y**

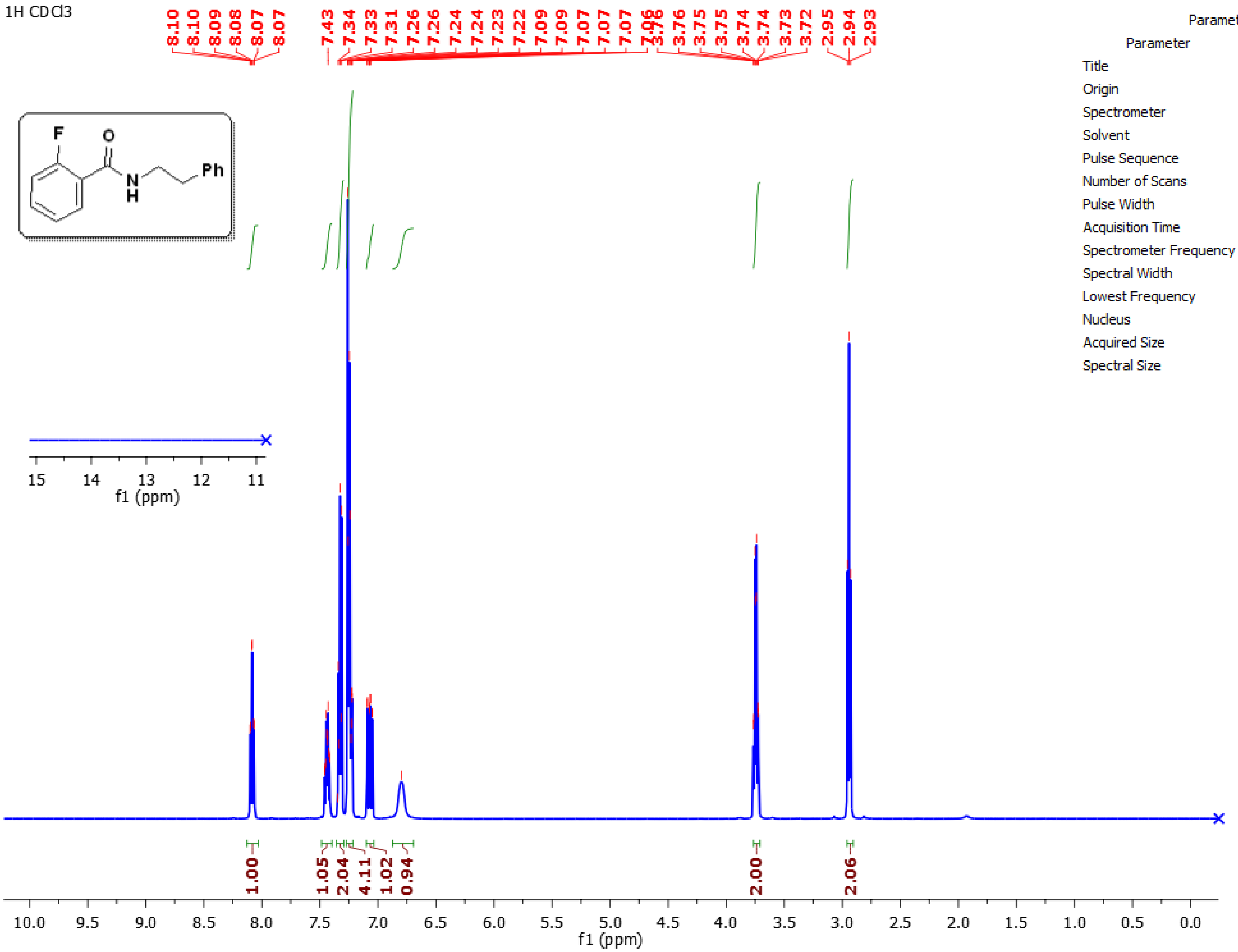

Parameters

Parameter	Value
Title	IVA 1445
Origin	Bruker BioSpin GmbH
Spectrometer	AV_III_500
Solvent	CDCl3
Pulse Sequence	zg30
Number of Scans	24
Pulse Width	9.9500
Acquisition Time	2.6564
Spectrometer Frequency	500.13
Spectral Width	12335.5
Lowest Frequency	-3190.6
Nucleus	1H
Acquired Size	32768
Spectral Size	65536

Compound **2y**

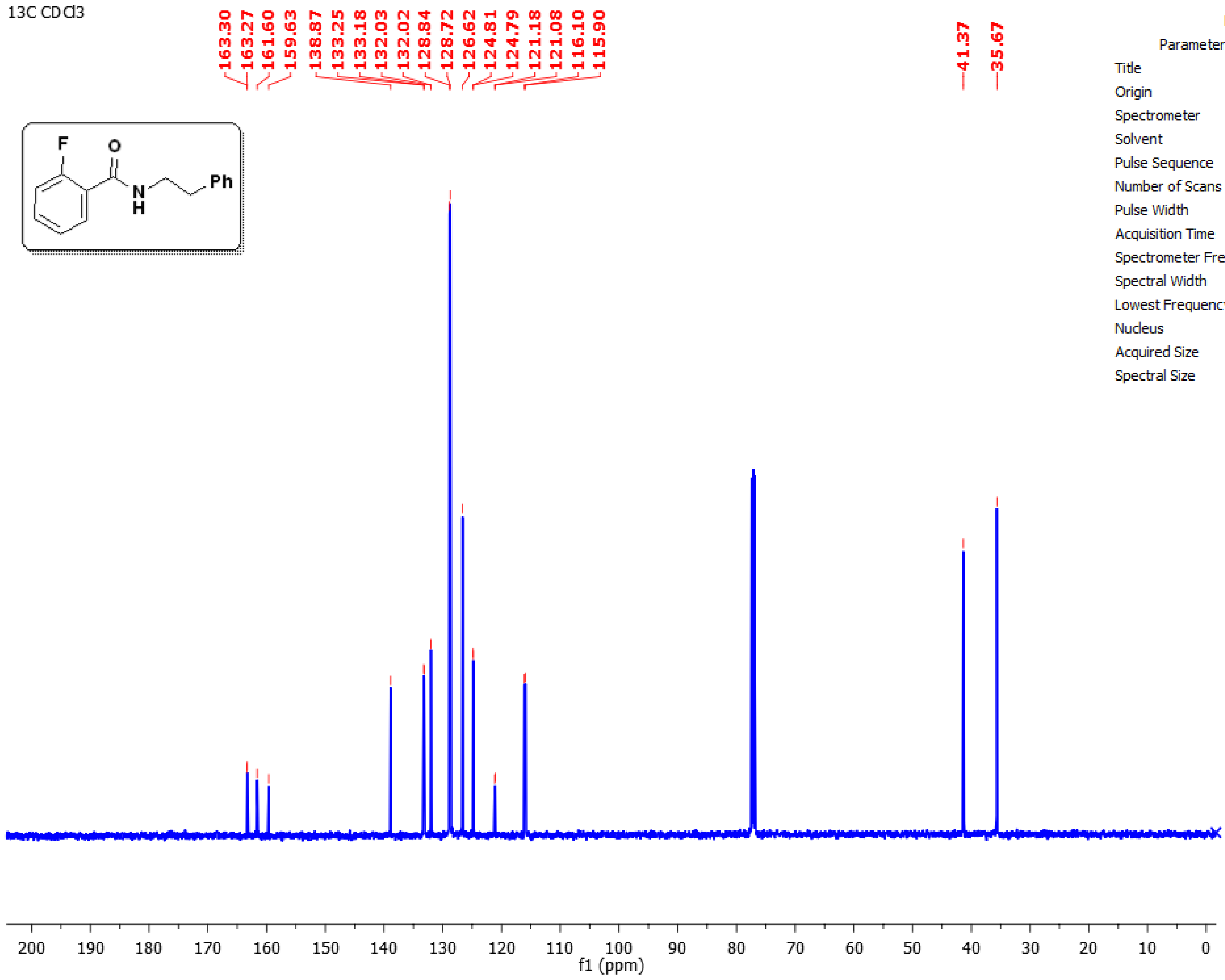

Parameters	
Parameter	Value
Title	IVA 1445
Origin	Bruker BioSpin GmbH
Spectrometer	AV_III_500
Solvent	CDCl3
Pulse Sequence	zgpg30
Number of Scans	512
Pulse Width	11.0000
Acquisition Time	0.9088
Spectrometer Frequency	125.76
Spectral Width	36057.7
Lowest Frequency	-2939.0
Nucleus	13C
Acquired Size	32768
Spectral Size	65536

Compound **2z**

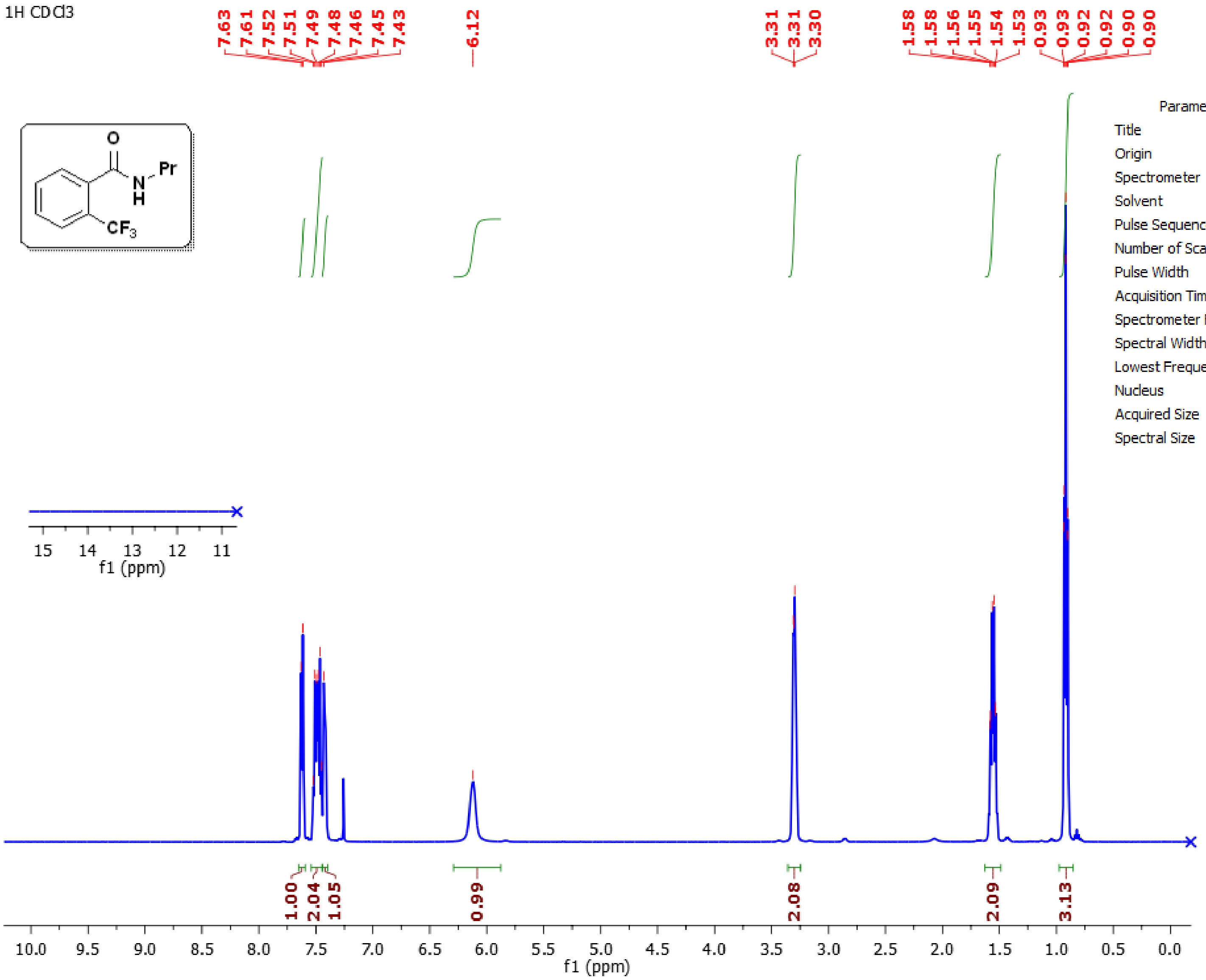

Parameters

Parameter	Value
Title	IVA 1436
Origin	Bruker BioSpin GmbH
Spectrometer	AV_III_500
Solvent	CDCl3
Pulse Sequence	zg30
Number of Scans	16
Pulse Width	9.9500
Acquisition Time	2.6564
Spectrometer Frequency	500.13
Spectral Width	12335.5
Lowest Frequency	-3190.6
Nucleus	1H
Acquired Size	32768
Spectral Size	65536

Compound **2z**

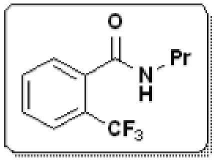

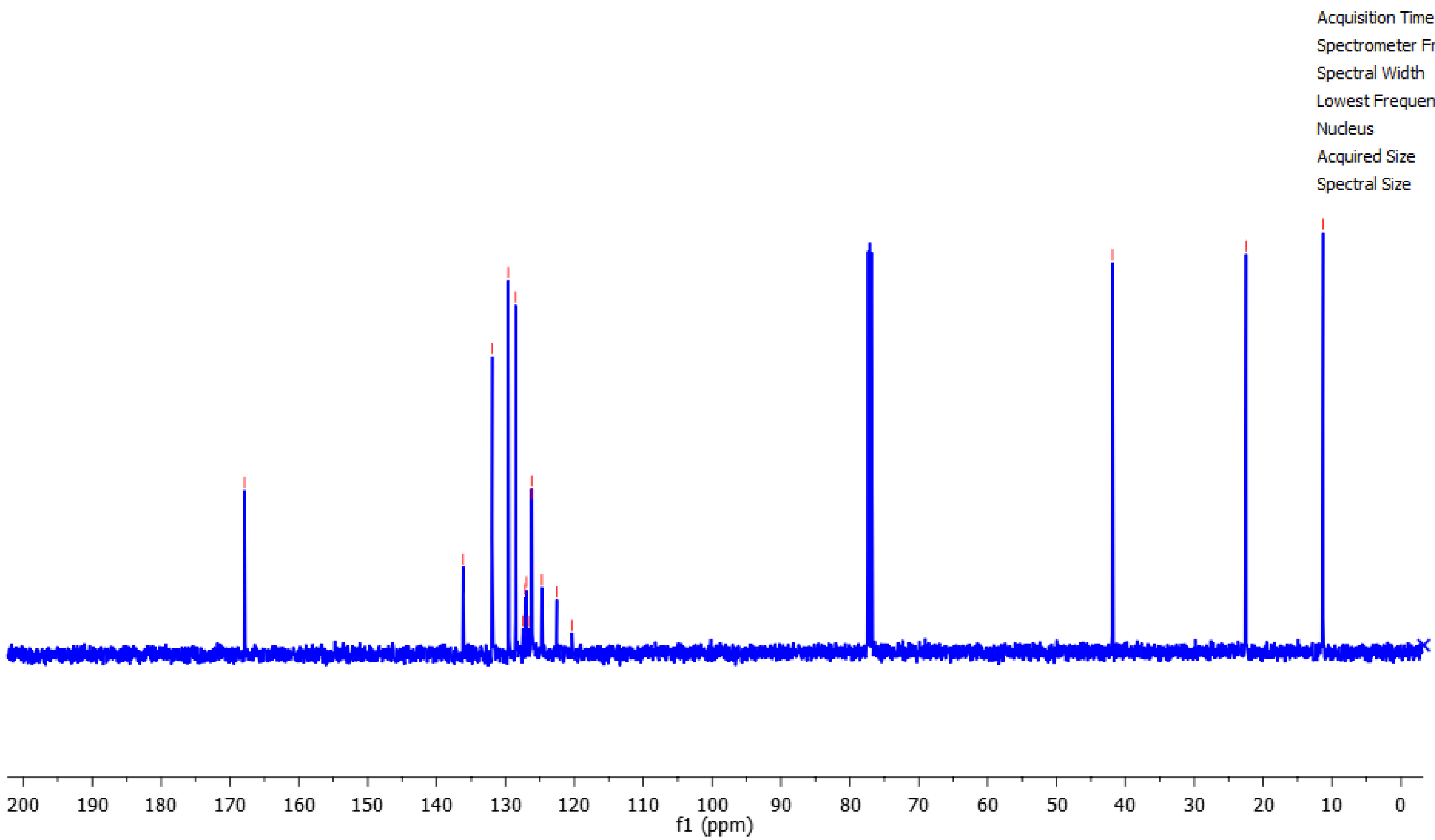

Parameters

Parameter	Value
Title	IVA 1436
Origin	Bruker BioSpin GmbH
Spectrometer	AV_III_500
Solvent	CDCl3
Pulse Sequence	zgpg30
Number of Scans	128
Pulse Width	11.0000
Acquisition Time	0.9088
Spectrometer Frequency	125.76
Spectral Width	36057.7
Lowest Frequency	-2939.0
Nucleus	13C
Acquired Size	32768
Spectral Size	65536

Compound **2aa**

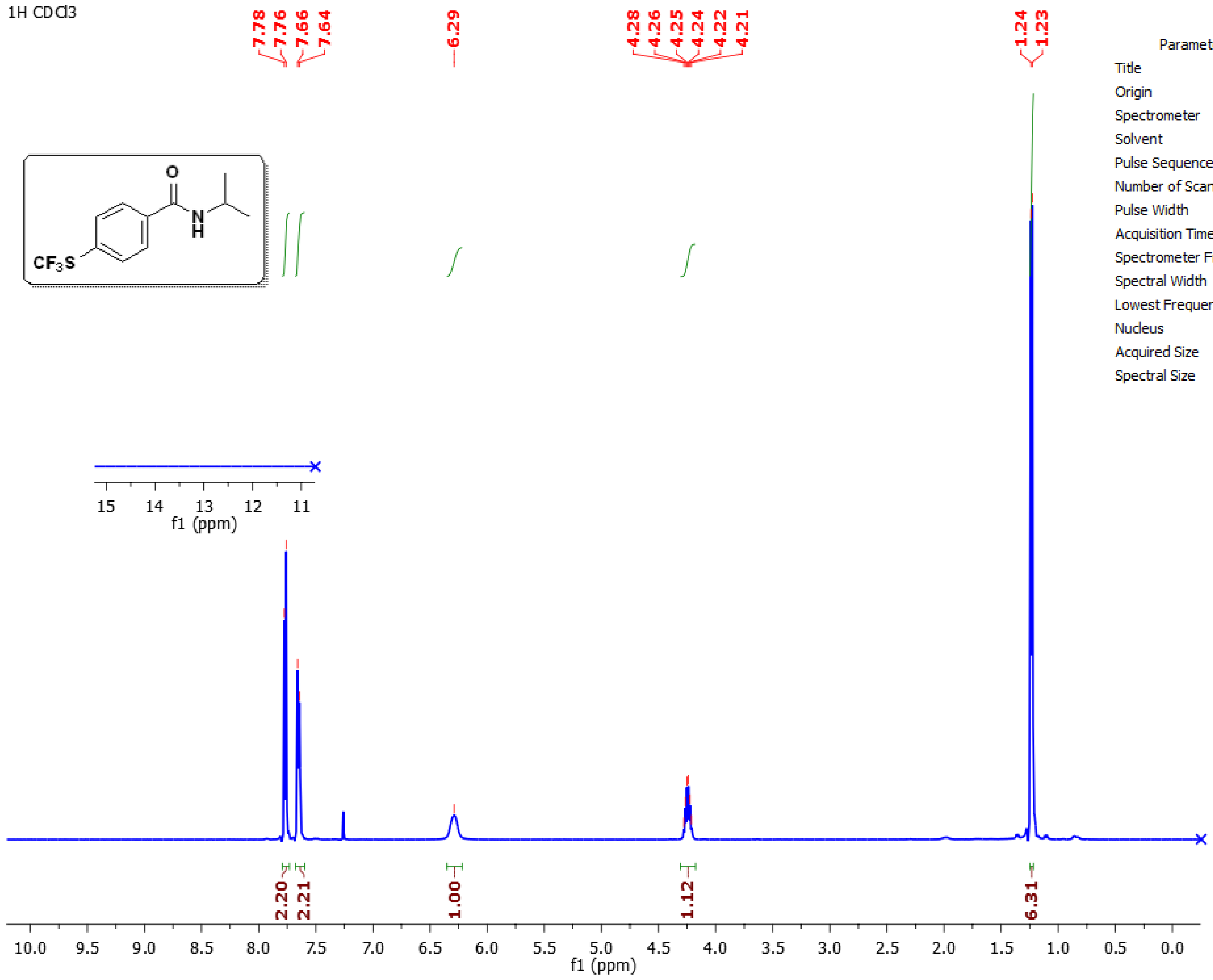

Parameters

Parameter	Value
Title	IVA 1463
Origin	Bruker BioSpin GmbH
Spectrometer	AV_III_500
Solvent	CDCl3
Pulse Sequence	zg30
Number of Scans	12
Pulse Width	9.9500
Acquisition Time	2.6564
Spectrometer Frequency	500.13
Spectral Width	12335.5
Lowest Frequency	-3190.6
Nucleus	1H
Acquired Size	32768
Spectral Size	65536

Compound **2aa**

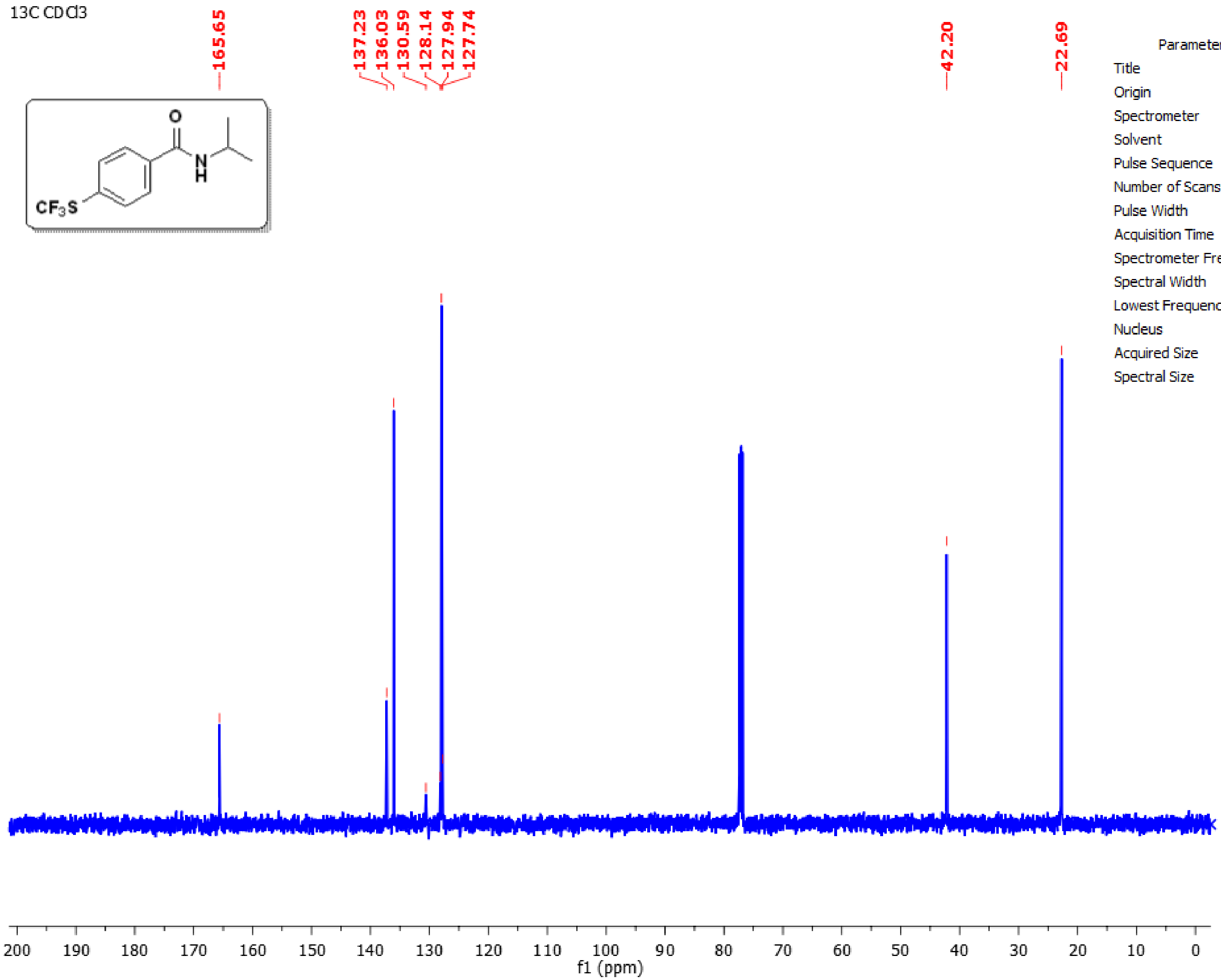

Parameters

Parameter	Value
Title	IVA 1463
Origin	Bruker BioSpin GmbH
Spectrometer	AV_III_500
Solvent	CDCl3
Pulse Sequence	zgpg30
Number of Scans	80
Pulse Width	11.0000
Acquisition Time	0.9088
Spectrometer Frequency	125.76
Spectral Width	36057.7
Lowest Frequency	-2939.0
Nucleus	13C
Acquired Size	32768
Spectral Size	65536

Compound **2ab**

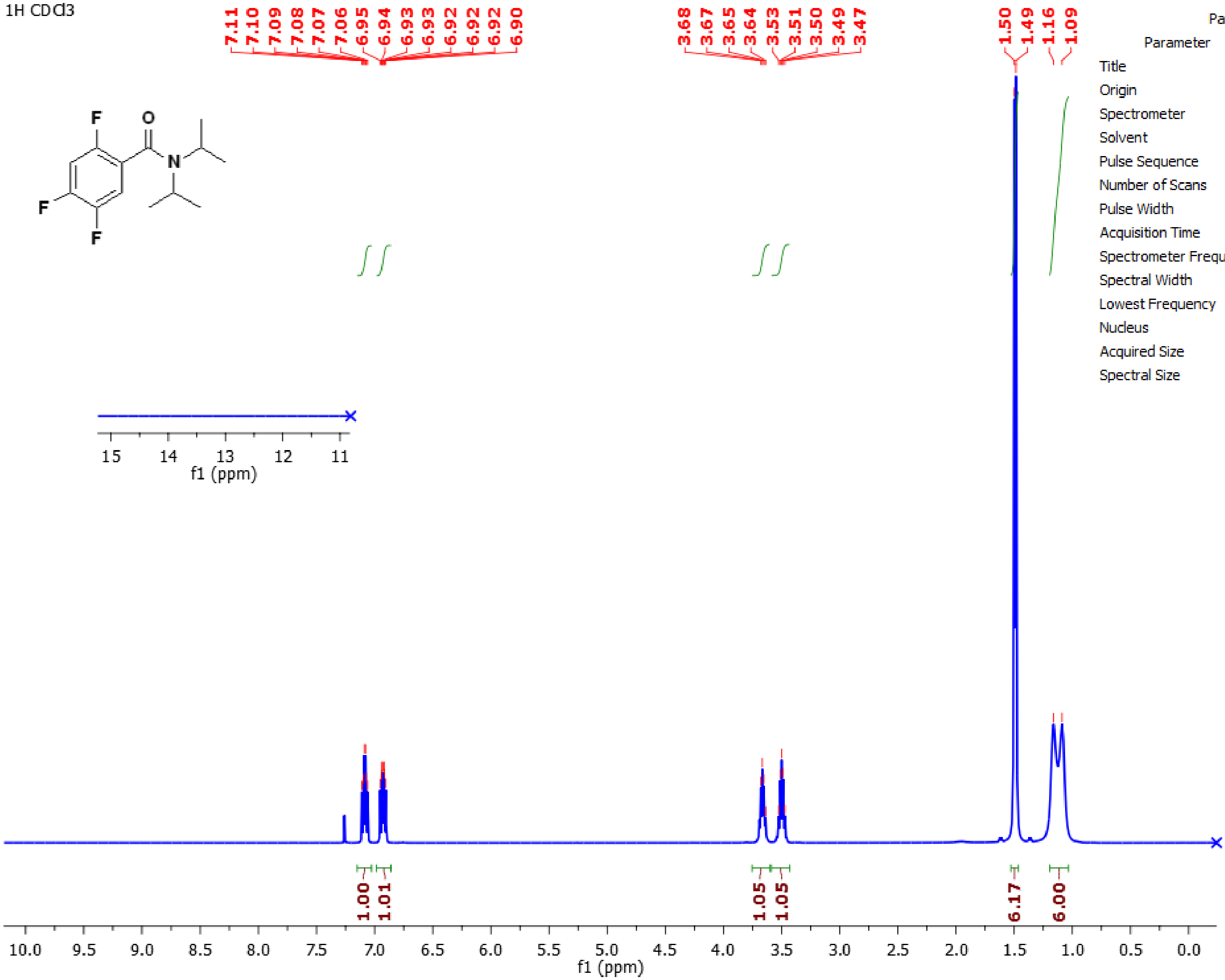

Parameter	Value
Title	IVA 1451
Origin	Bruker BioSpin GmbH
Spectrometer	AV_III_500
Solvent	CDCl3
Pulse Sequence	zg30
Number of Scans	24
Pulse Width	9.9500
Acquisition Time	2.6564
Spectrometer Frequency	500.13
Spectral Width	12335.5
Lowest Frequency	-3190.6
Nucleus	1H
Acquired Size	32768
Spectral Size	65536

Compound **2ab**

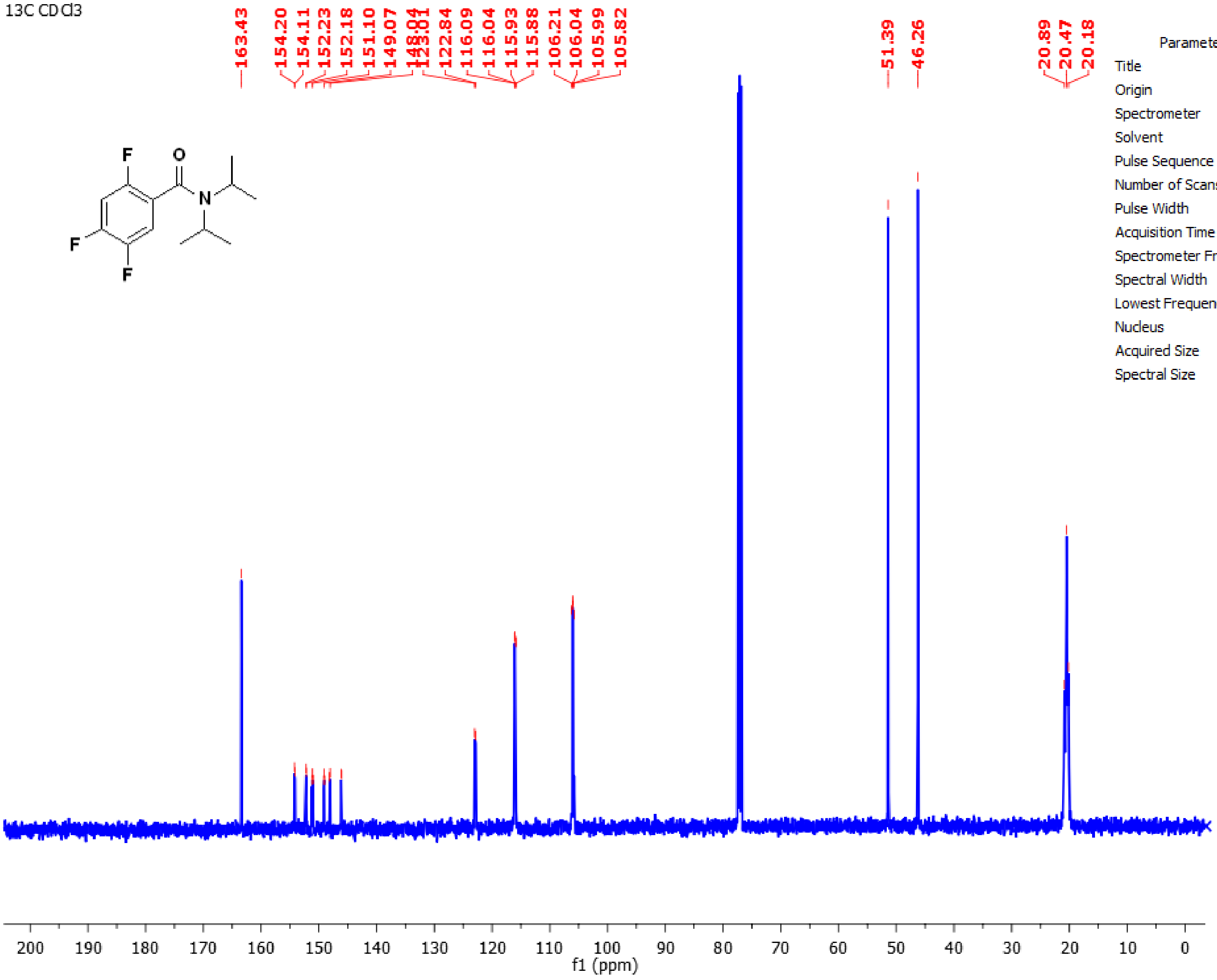

Parameters

Parameter	Value
Title	IVA 1451
Origin	Bruker BioSpin GmbH
Spectrometer	AV_III_500
Solvent	CDCl3
Pulse Sequence	zgpg30
Number of Scans	512
Pulse Width	11.0000
Acquisition Time	0.9088
Spectrometer Frequency	125.76
Spectral Width	36057.7
Lowest Frequency	-2939.0
Nucleus	13C
Acquired Size	32768
Spectral Size	65536

Compound **2ac**

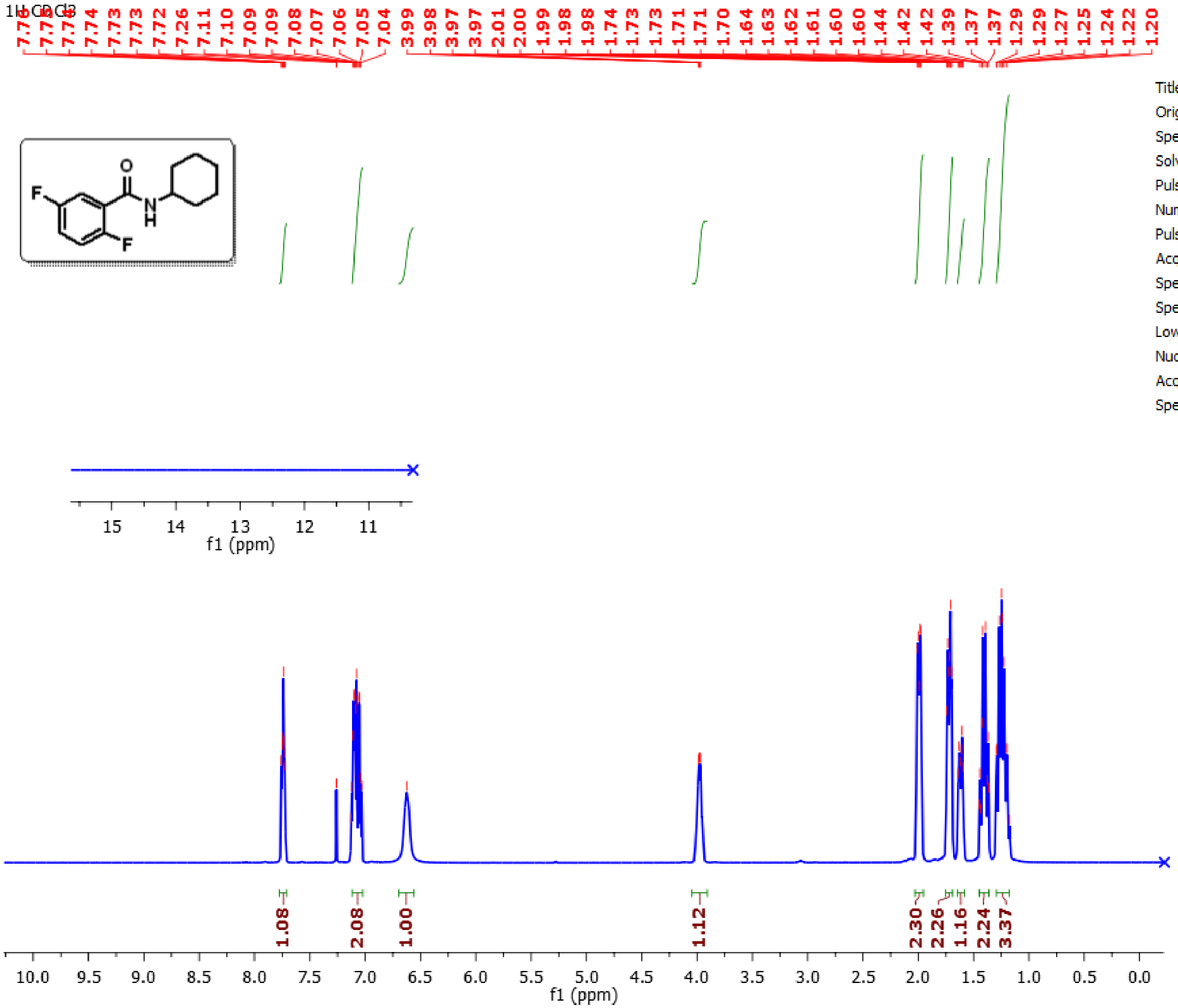

Parameter	Value
Title	IVA 1757
Origin	Bruker BioSpin GmbH
Spectrometer	AV_III_500
Solvent	CDCl3
Pulse Sequence	zg30
Number of Scans	24
Pulse Width	9.6600
Acquisition Time	2.6564
Spectrometer Frequency	500.13
Spectral Width	12335.5
Lowest Frequency	-3190.6
Nucleus	1H
Acquired Size	32768
Spectral Size	65536

Compound **2ac**

13C CDCl3

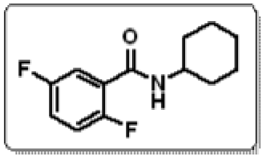

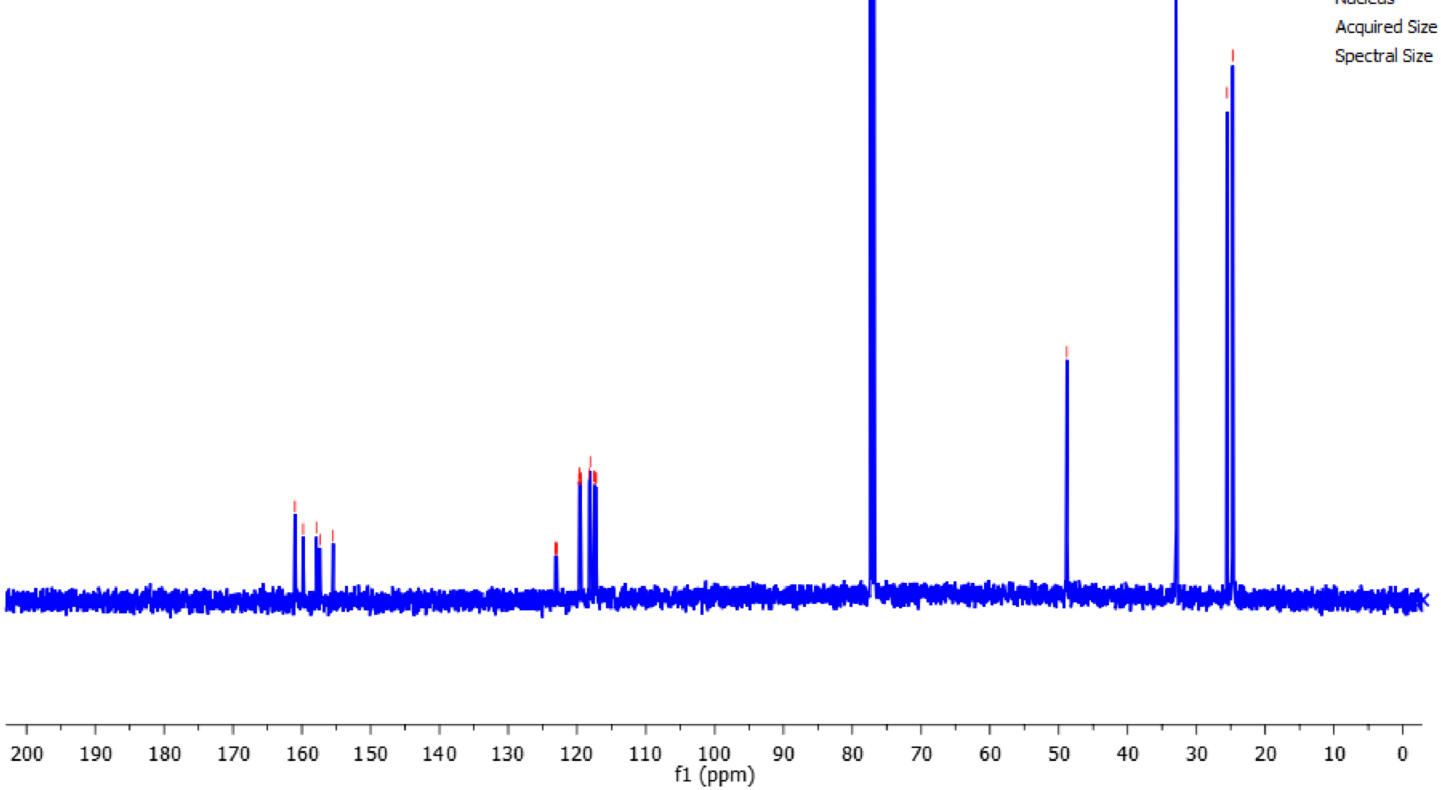

Parameters

Parameter	Value
Title	IVA 1757
Origin	Bruker BioSpin GmbH
Spectrometer	AV_III_500
Solvent	CDCl3
Pulse Sequence	zgpg30
Number of Scans	256
Pulse Width	11.5000
Acquisition Time	0.9088
Spectrometer Frequency	125.76
Spectral Width	36057.7
Lowest Frequency	-2939.0
Nucleus	13C
Acquired Size	32768
Spectral Size	65536

Compound **2ad**

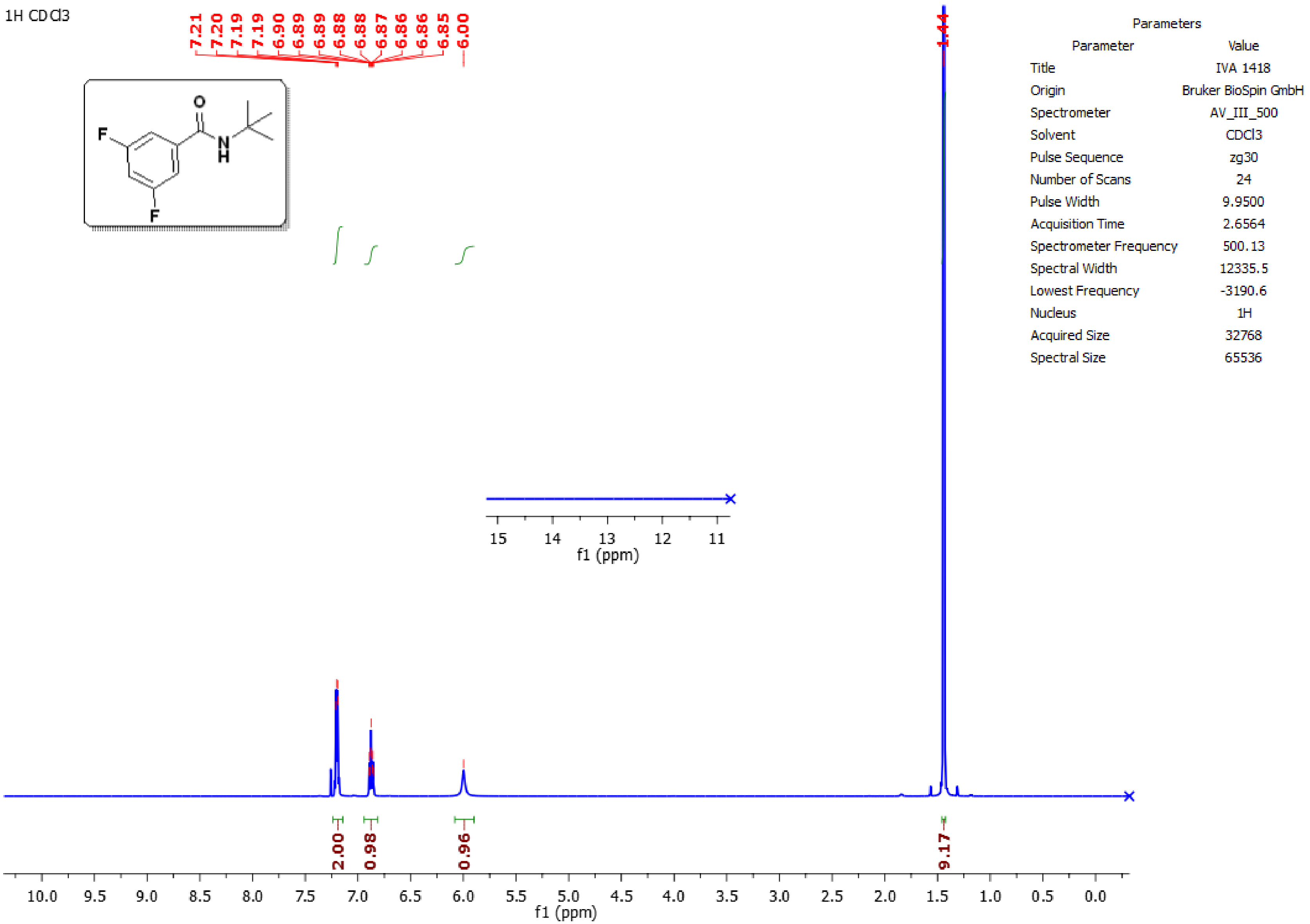

Compound **2ad**

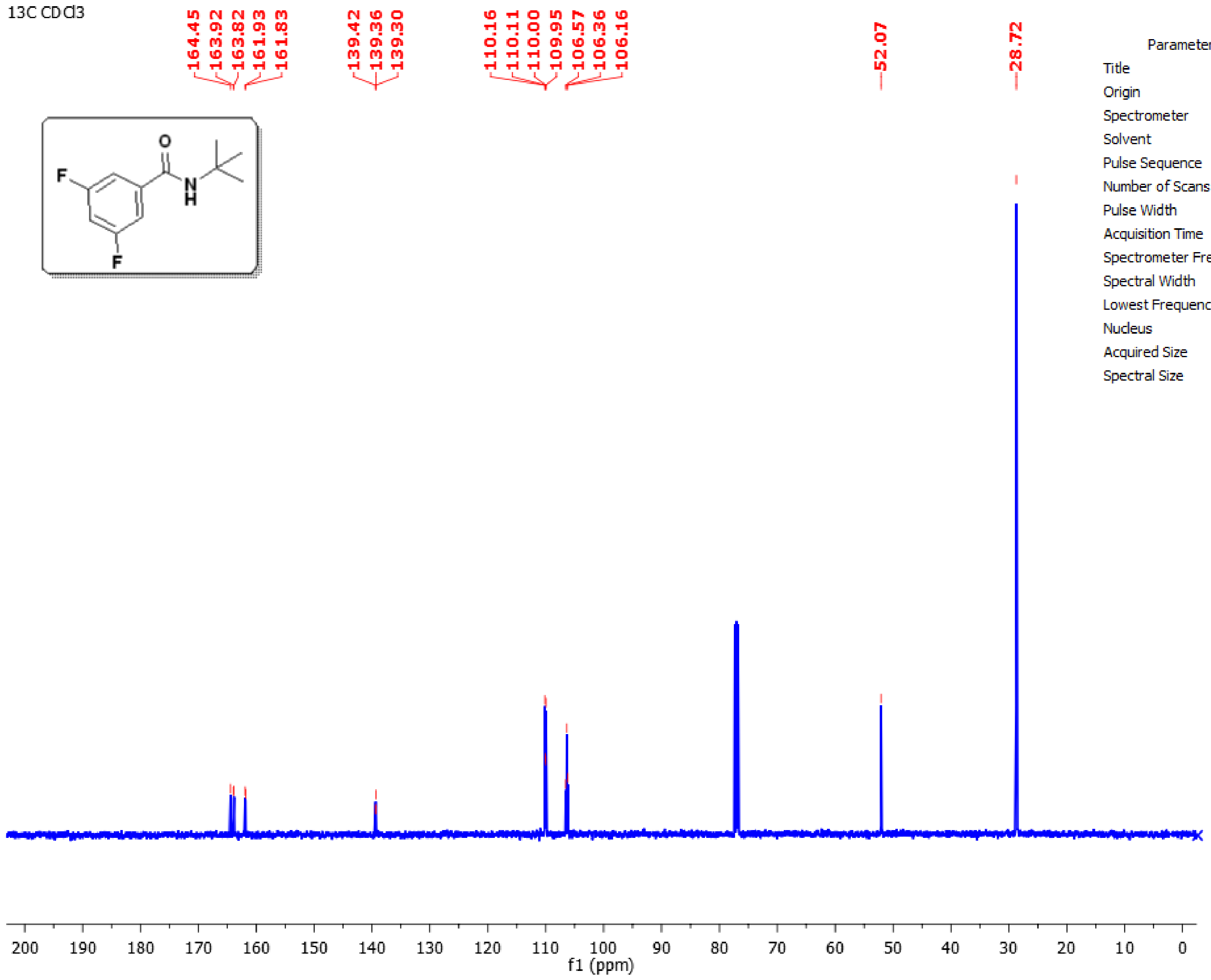

Parameters

Parameter	Value
Title	IVA 1418
Origin	Bruker BioSpin GmbH
Spectrometer	AV_III_500
Solvent	CDCl3
Pulse Sequence	zgpg30
Number of Scans	196
Pulse Width	11.0000
Acquisition Time	0.9088
Spectrometer Frequency	125.76
Spectral Width	36057.7
Lowest Frequency	-2939.0
Nucleus	13C
Acquired Size	32768
Spectral Size	65536

Compound **2ae**

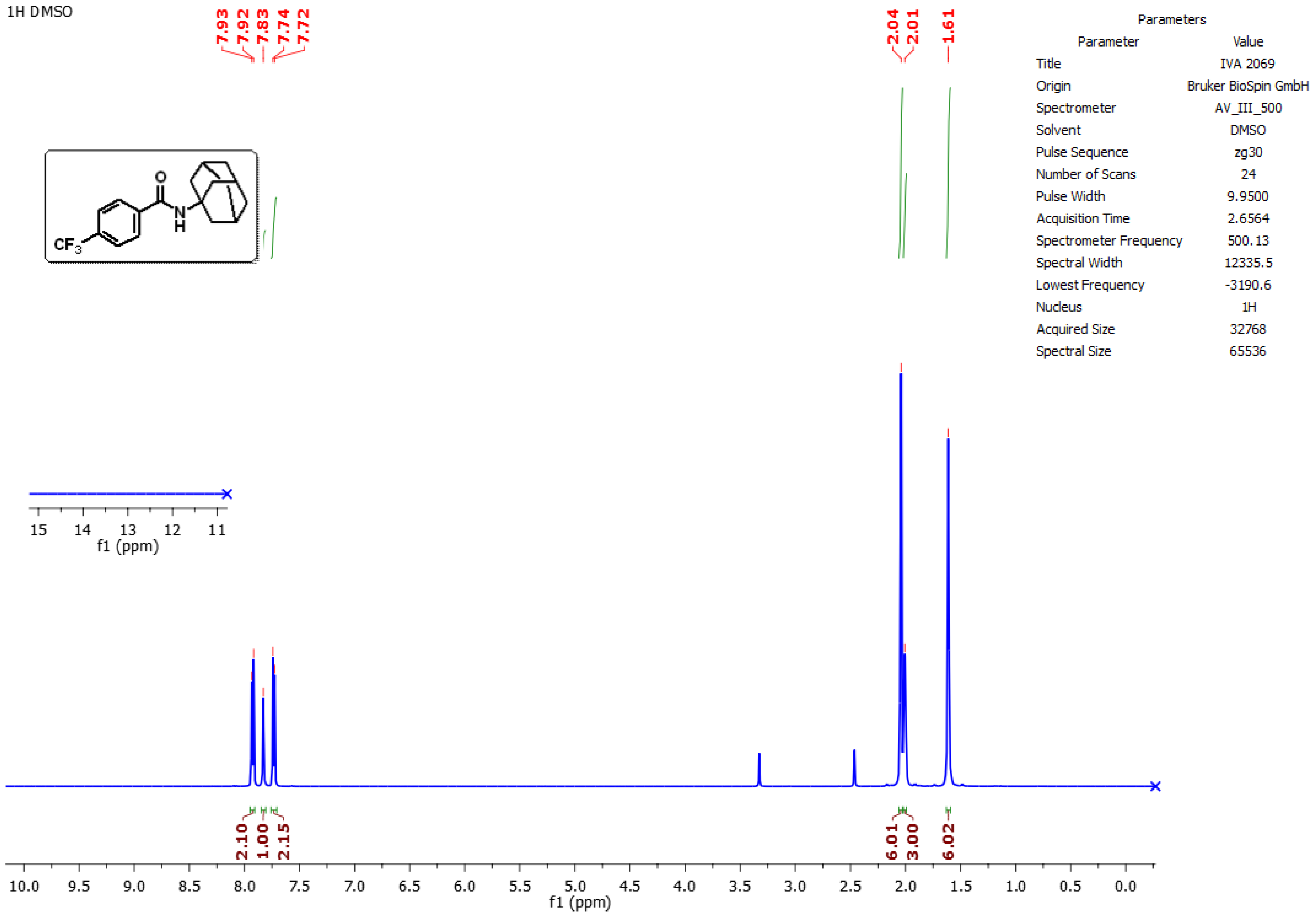

Compound **2ae**

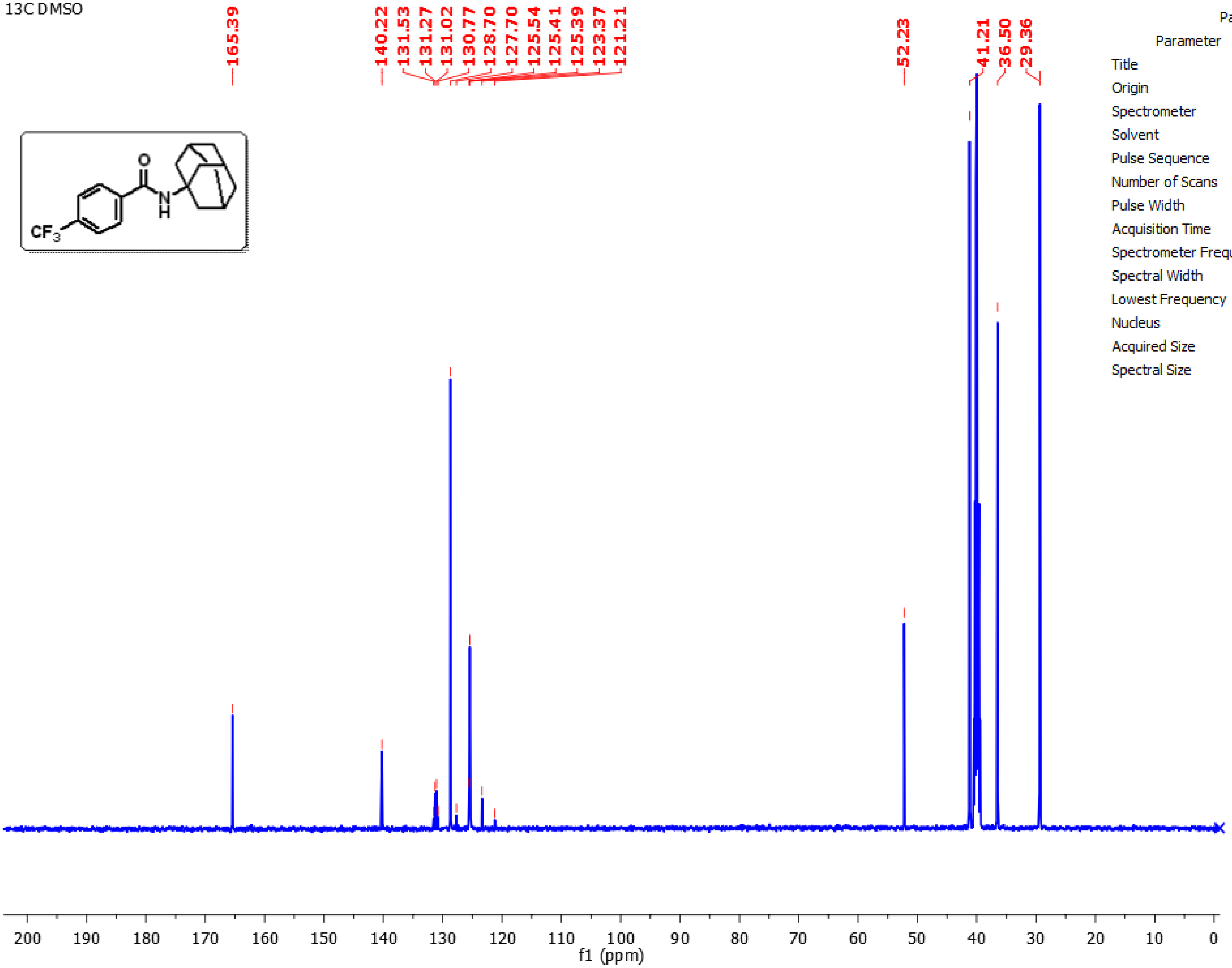

Parameters

Parameter	Value
Title	IVA 2069
Origin	Bruker BioSpin GmbH
Spectrometer	AV_III_500
Solvent	DMSO
Pulse Sequence	zgpg30
Number of Scans	512
Pulse Width	11.0000
Acquisition Time	0.9088
Spectrometer Frequency	125.76
Spectral Width	36057.7
Lowest Frequency	-2939.0
Nucleus	13C
Acquired Size	32768
Spectral Size	65536

(E) DFT Studies

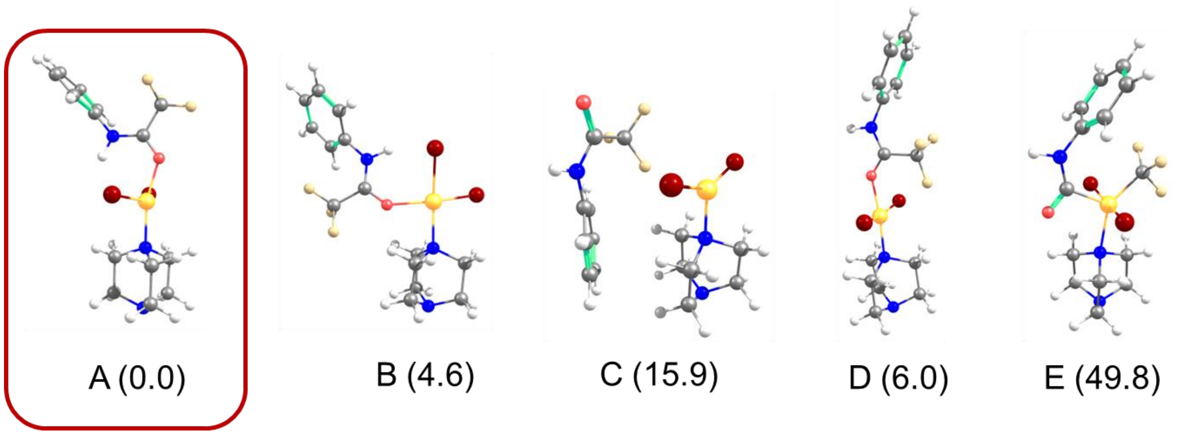

Figure S1. Relative electronic energies (in kcal/mol) of Ni^{2+} cations coordinated by DABCO and two Br^{-} anions. The structures were optimized at the ωB97X-D/def2-SVP level of theory (color coding: H/C/N/O/F/Ni/Br – white/ gray/blue/red/beige/yellow/dark red).

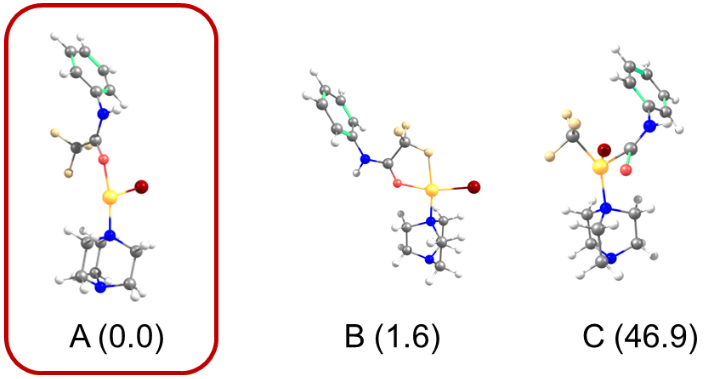

Figure S2. Relative electronic energies (in kcal/mol) of Ni^{2+} cations coordinated by DABCO and a single Br^{-} anion. The structures were optimized at the ωB97X-D/def2-SVP level of theory (color coding: H/C/N/O/F/Ni/Br – white/ gray/blue/red/beige/yellow/dark red).

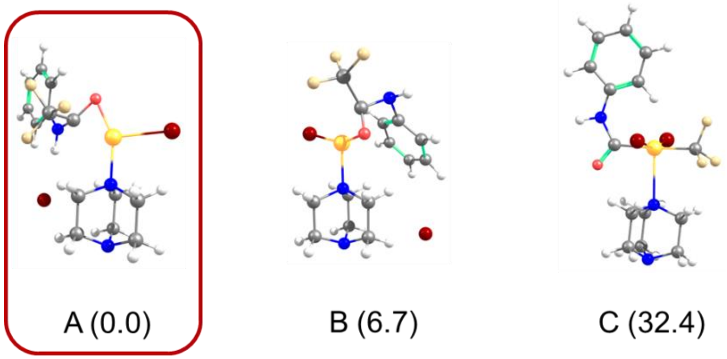

Figure S3. Relative electronic energies (in kcal/mol) of Ni^0 atoms coordinated by DABCO and two Br^- anions. The structures were optimized at the ωB97X-D/def2-SVP level of theory (color coding: H/C/N/O/F/Ni/Br – white/ gray/blue/red/beige/yellow/dark red).

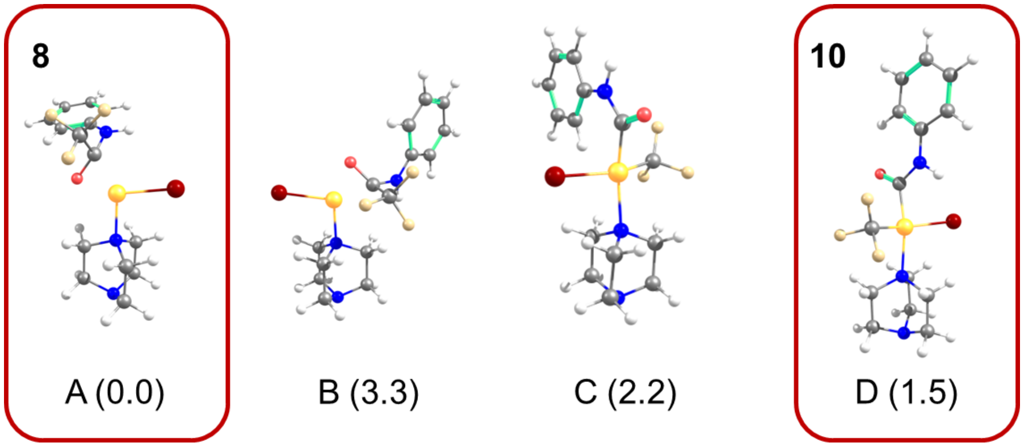

Figure S4. Relative electronic energies (in kcal/mol) of Ni^0 atoms coordinated by DABCO and a single Br^- anion. The structures were optimized at the ωB97X-D/def2-SVP level of theory (color coding: H/C/N/O/F/Ni/Br – white/ gray/blue/red/beige/yellow/dark red).

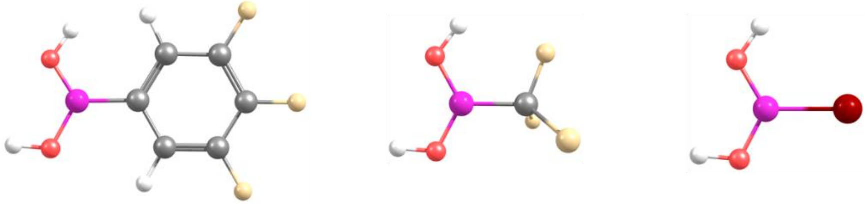

Figure S5. The molecular structures of compounds appearing in figure 1 optimized at the ωB97X-D/def2-SVP level of theory (color coding: H/C/N/O/F/Ni/Br – white/ gray/blue/red/beige/yellow/dark red).

Cartesian coordinates

Structure 7

E(RwB97XD) = -5164.43557390 a.u.

41

**C**	**-2.572175000**	**1.232605000**	**-1.170516000**
**N**	**-4.367094000**	**2.069914000**	**0.312861000**
**C**	**-3.862890000**	**2.089733000**	**-1.057956000**
**H**	**-1.703712000**	**1.831702000**	**-1.480402000**
**H**	**-2.664279000**	**0.401477000**	**-1.883960000**
**H**	**-3.675265000**	**3.136859000**	**-1.345107000**
**H**	**-4.653442000**	**1.708422000**	**-1.723860000**
**C**	**-4.668734000**	**0.690312000**	**0.686081000**
**H**	**-5.077037000**	**0.688233000**	**1.709612000**
**H**	**-5.462979000**	**0.316076000**	**0.020545000**

C	-2.049721000	1.732092000	1.120149000
H	-1.776112000	1.269600000	2.078212000
H	-1.166855000	2.301066000	0.799461000
C	-3.338732000	2.594650000	1.208468000
H	-3.743252000	2.605572000	2.233389000
H	-3.138002000	3.641762000	0.930482000
C	-3.391281000	-0.188038000	0.584435000
H	-3.118340000	-0.635369000	1.551210000
H	-3.487587000	-1.011904000	-0.136730000
N	-2.260894000	0.647772000	0.145280000
Ni	-0.547238000	-0.446336000	0.027750000
Br	-1.223500000	-2.066993000	-1.541525000
C	3.164158000	0.598048000	-0.566871000
C	3.457429000	1.408113000	0.553960000
C	3.903187000	0.825165000	-1.749509000
C	4.450637000	2.376048000	0.480250000
C	4.894907000	1.798533000	-1.803606000
C	5.185490000	2.587734000	-0.690230000
H	2.878403000	1.268013000	1.465321000
H	3.685891000	0.216502000	-2.632269000
H	4.654145000	2.986481000	1.365303000
H	5.448653000	1.941916000	-2.736428000
H	5.963397000	3.353583000	-0.733498000
N	2.211209000	-0.381509000	-0.531938000

H	1.982460000	-0.828166000	-1.409957000
C	1.230196000	-0.578327000	0.514899000
O	0.658202000	0.484866000	1.068617000
C	1.695048000	-1.633746000	1.494147000
F	2.013666000	-2.786678000	0.885336000
F	2.804402000	-1.269705000	2.189964000
F	0.767021000	-1.919103000	2.419197000

========================

Structure 8

E(RwB97XD) = -5164.43310923 a.u.

41

C	2.947093000	-0.944489000	1.121186000
N	5.055682000	-0.258057000	0.020387000
C	4.478827000	-1.164158000	1.007972000
H	2.656869000	-0.557139000	2.109182000
H	2.381196000	-1.868770000	0.954848000
H	4.981480000	-0.996328000	1.974354000
H	4.704697000	-2.198037000	0.701808000
C	4.465800000	-0.540286000	-1.286160000
H	4.902225000	0.157666000	-2.018802000
H	4.763317000	-1.556553000	-1.590298000
C	3.190746000	1.323170000	0.414327000
H	2.881848000	2.056572000	-0.335461000

H	2.822101000	1.683501000	1.383697000
C	4.728943000	1.110429000	0.403285000
H	5.214499000	1.799734000	-0.306131000
H	5.168483000	1.306239000	1.394661000
C	2.920076000	-0.405538000	-1.230508000
H	2.537913000	0.330782000	-1.949969000
H	2.407645000	-1.358381000	-1.422156000
N	2.507709000	0.046735000	0.113787000
Ni	0.355825000	0.134410000	0.132807000
Br	0.107865000	-2.274121000	-0.114998000
C	-3.795116000	-0.070272000	-0.118741000
C	-4.463386000	0.354922000	1.046553000
C	-4.573051000	-0.505099000	-1.210308000
C	-5.854563000	0.341479000	1.094566000
C	-5.961600000	-0.513479000	-1.144451000
C	-6.620220000	-0.088746000	0.010088000
H	-3.861491000	0.681737000	1.891820000
H	-4.067755000	-0.841240000	-2.120164000
H	-6.350727000	0.676490000	2.010019000
H	-6.535453000	-0.857857000	-2.009439000
H	-7.711483000	-0.094741000	0.063515000
N	-2.417472000	-0.074077000	-0.247426000
H	-2.050017000	-0.443106000	-1.114807000
C	-1.424525000	0.256530000	0.675475000

O	-1.701120000	0.588427000	1.815521000
C	0.249823000	1.977346000	-0.371108000
F	1.046827000	2.215895000	-1.496269000
F	-0.949226000	2.485838000	-0.760184000
F	0.710258000	2.899349000	0.532586000

========================

Structure of the TS (8-to-10)

E(RwB97XD) = -5164.36721913 a.u.

41

C	3.220985000	0.624134000	-1.255860000
N	4.959714000	-0.910992000	-0.393921000
C	4.719984000	0.217135000	-1.290661000
H	2.740983000	0.523721000	-2.240316000
H	3.058549000	1.660293000	-0.926930000
H	5.034783000	-0.072484000	-2.306344000
H	5.366971000	1.052256000	-0.977930000
C	4.593557000	-0.522179000	0.966564000
H	4.805432000	-1.368990000	1.638915000
H	5.246380000	0.309167000	1.277223000
C	2.626669000	-1.650497000	-0.768467000
H	2.046294000	-2.275762000	-0.078855000
H	2.144331000	-1.727270000	-1.753512000
C	4.131578000	-2.039490000	-0.810770000

H	4.341141000	-2.890234000	-0.142893000
H	4.439542000	-2.341109000	-1.824906000
C	3.095185000	-0.116547000	1.025346000
H	2.515567000	-0.752499000	1.707430000
H	2.944057000	0.925988000	1.340593000
N	2.503969000	-0.253842000	-0.317044000
Ni	0.508667000	0.246449000	-0.202667000
Br	0.577967000	2.565704000	0.182701000
C	-3.661520000	0.113988000	-0.258399000
C	-3.988529000	-1.022323000	-1.025944000
C	-4.713976000	0.900200000	0.250005000
C	-5.320596000	-1.345974000	-1.255893000
C	-6.041443000	0.563700000	0.005167000
C	-6.362261000	-0.564021000	-0.750191000
H	-3.172911000	-1.622767000	-1.426630000
H	-4.474130000	1.784690000	0.846838000
H	-5.549654000	-2.234971000	-1.850789000
H	-6.835643000	1.194558000	0.414693000
H	-7.404612000	-0.829162000	-0.942323000
N	-2.360091000	0.477931000	0.011394000
H	-2.206844000	1.283002000	0.606300000
C	-1.178771000	-0.179702000	-0.340346000
O	-1.077953000	-0.949463000	-1.324113000
C	-0.695824000	-1.163012000	1.266080000

F	-0.820591000	-0.412578000	2.385001000
F	-1.720560000	-2.049512000	1.317239000
F	0.411874000	-1.960922000	1.505504000

========================

Structure 9

E(RwB97XD) = -5355.93810790 a.u.

48

C	-2.952252000	1.460628000	-0.515957000
N	-3.349162000	2.632866000	1.629167000
C	-3.910065000	2.360851000	0.309207000
H	-2.552233000	1.980704000	-1.399059000
H	-3.440311000	0.543412000	-0.868404000
H	-4.094967000	3.321715000	-0.197688000
H	-4.888304000	1.872691000	0.443664000
C	-3.186382000	1.372186000	2.347759000
H	-2.760317000	1.592539000	3.339788000
H	-4.183140000	0.933620000	2.513501000
C	-1.076702000	2.291451000	0.710515000
H	-0.224864000	1.999377000	1.336037000
H	-0.654228000	2.745232000	-0.195941000
C	-2.039850000	3.251173000	1.459986000
H	-1.641455000	3.511995000	2.453491000
H	-2.170450000	4.195619000	0.907899000

C	-2.280615000	0.398979000	1.545176000
H	-1.396592000	0.084196000	2.120260000
H	-2.812707000	-0.510162000	1.234628000
N	-1.796943000	1.064251000	0.317550000
Ni	-0.638015000	-0.386789000	-0.749884000
Br	-2.556433000	-1.881649000	-1.058265000
C	1.474427000	-2.669404000	0.138634000
C	0.417427000	-2.636328000	1.065250000
C	2.779151000	-2.869980000	0.616864000
C	0.680955000	-2.733729000	2.428444000
C	3.028257000	-2.990805000	1.981120000
C	1.983570000	-2.904904000	2.901273000
H	-0.606010000	-2.552544000	0.696179000
H	3.608042000	-2.889112000	-0.095717000
H	-0.154623000	-2.693772000	3.132851000
H	4.055458000	-3.128630000	2.328846000
H	2.181295000	-2.980860000	3.973032000
N	1.244734000	-2.489859000	-1.219154000
H	1.953430000	-2.835387000	-1.859452000
C	0.427305000	-1.499021000	-1.801531000
O	0.505750000	-1.369268000	-3.010837000
C	2.890019000	1.345831000	0.755177000
C	3.295942000	2.201302000	-0.264999000
C	1.762483000	0.547027000	0.620883000

H	1.514216000	-0.104948000	1.463382000
C	2.544818000	2.218270000	-1.438243000
C	0.983230000	0.568020000	-0.552408000
C	1.421862000	1.417527000	-1.585887000
H	0.904978000	1.436827000	-2.549205000
F	3.618440000	1.317222000	1.880289000
F	4.381138000	2.973587000	-0.128584000
F	2.945360000	3.030771000	-2.426806000

========================

Structure 10

E(RwB97XD) = -3119.26384340 a.u.

51

C	2.303191000	1.613981000	0.191326000
N	4.205709000	0.901725000	1.602431000
C	3.545227000	2.057719000	1.009196000
H	2.357773000	1.921507000	-0.862452000
H	1.383824000	2.047690000	0.597410000
H	4.274526000	2.588414000	0.376631000
H	3.255068000	2.749422000	1.815904000
C	3.256170000	0.194533000	2.456766000
H	3.774714000	-0.659403000	2.920571000
H	2.952341000	0.870899000	3.271419000
C	3.418109000	-0.436810000	-0.325287000

H	3.321400000	-1.524198000	-0.329926000
H	3.495133000	-0.115190000	-1.373093000
C	4.634271000	0.015882000	0.526786000
H	5.143125000	-0.853201000	0.973044000
H	5.380087000	0.547043000	-0.086061000
C	2.026444000	-0.281782000	1.634182000
H	1.922039000	-1.373685000	1.637455000
H	1.089424000	0.150202000	2.013014000
N	2.172687000	0.141311000	0.226955000
Ni	0.411147000	-0.568620000	-0.763016000
C	-2.586083000	-1.898731000	0.007347000
C	-1.648994000	-2.001482000	1.053272000
C	-3.927956000	-2.212782000	0.299067000
C	-2.051757000	-2.381755000	2.327713000
C	-4.317445000	-2.599609000	1.577161000
C	-3.384985000	-2.683764000	2.610452000
H	-0.600347000	-1.786690000	0.845508000
H	-4.672926000	-2.140381000	-0.498522000
H	-1.296894000	-2.452328000	3.115732000
H	-5.369060000	-2.832387000	1.766270000
H	-3.689835000	-2.983674000	3.615493000
N	-2.255669000	-1.517147000	-1.275850000
H	-2.984767000	-1.596121000	-1.978706000
C	-1.071501000	-0.973004000	-1.801756000

**O**	**-1.089109000**	**-0.733122000**	**-3.000664000**
**C**	**1.098662000**	**-2.393721000**	**-1.062651000**
**F**	**1.731257000**	**-2.963972000**	**0.059547000**
**F**	**0.201918000**	**-3.365452000**	**-1.390638000**
**F**	**2.066330000**	**-2.515745000**	**-2.033980000**
**C**	**-1.168617000**	**3.307516000**	**-1.258858000**
**C**	**-1.739849000**	**3.636167000**	**-0.030077000**
**C**	**-0.597990000**	**2.063546000**	**-1.476806000**
**H**	**-0.206657000**	**1.849994000**	**-2.475794000**
**C**	**-1.734240000**	**2.667652000**	**0.968399000**
**C**	**-0.546932000**	**1.083389000**	**-0.465834000**
**C**	**-1.158043000**	**1.420325000**	**0.758332000**
**H**	**-1.213034000**	**0.708362000**	**1.587640000**
**F**	**-1.191896000**	**4.236170000**	**-2.226186000**
**F**	**-2.290747000**	**4.838994000**	**0.175959000**
**F**	**-2.301346000**	**2.970589000**	**2.145418000**

========================

Structure of Ph-B$(OH)_2$

E(RwB97XD) = -705.229280051 a.u.

16

**B**	**2.593117000**	**0.004408000**	**-0.004723000**
**O**	**3.208848000**	**-1.191493000**	**-0.184578000**
**H**	**4.167530000**	**-1.117253000**	**-0.153044000**

O	3.364580000	1.114440000	0.172083000
H	2.878469000	1.921487000	0.352642000
C	1.013708000	0.023859000	-0.004848000
C	0.280911000	1.218161000	-0.037888000
C	0.314148000	-1.190635000	0.026949000
C	-1.106365000	1.195925000	-0.035037000
C	-1.072296000	-1.202550000	0.033557000
C	-1.799596000	-0.012559000	0.002784000
H	0.758720000	2.200344000	-0.080169000
H	0.848105000	-2.142091000	0.045826000
F	-1.802013000	2.325859000	-0.070147000
F	-3.121488000	-0.029901000	0.007322000
F	-1.741930000	-2.347215000	0.067849000

========================

Structure of CF_3-B(OH)$_2$

E(RwB97XD) = -513.721530462 a.u.

9

C	0.965632000	-0.434173000	2.484876000
F	2.147104000	-1.035138000	2.304810000
F	1.211465000	0.720338000	3.151907000
F	0.239289000	-1.205041000	3.302144000
B	0.207033000	-0.125563000	1.086685000
O	-0.112284000	-1.172228000	0.307010000

H -0.559541000 -0.917044000 -0.506434000

O -0.079096000 1.149873000 0.747220000

H 0.211297000 1.791119000 1.404141000

=======================

Structure of CF_3-B(OH)$_2$

E(RwB97XD) = -2750.36822745 a.u.

6

B 0.960761000 -0.004856000 -0.000039000

O 1.593931000 -1.191199000 -0.000027000

H 2.551899000 -1.091867000 0.000790000

O 1.645417000 1.162100000 -0.000226000

H 1.088456000 1.944939000 0.001395000

Br -0.981684000 -0.017029000 0.000001000
